# N_2_ Dissociation vs Reversible 1,2-Methyl Migration in PC_NHC_P Cobalt(I) Complexes in the Stereoselective Isomerization (*E/Z*) of Allyl Ethers

**DOI:** 10.1021/jacsau.4c00529

**Published:** 2024-09-18

**Authors:** Sakthi Raje, Subhash Garhwal, Katarzyna Młodzikowska-Pieńko, Tofayel Sheikh Mohammad, Ron Raphaeli, Natalia Fridman, Linda J. W. Shimon, Renana Gershoni-Poranne, Graham de Ruiter

**Affiliations:** † Schulich Faculty of Chemistry and the Resnick Sustainability Center for Catalysis, Technion − Israel Institute of Technology, Technion City, Haifa 3200008, Israel; ‡ Department of Chemical Research Support, Weizmann Institute of Science, Rehovot 7610001, Israel

**Keywords:** isomerization, cobalt, catalysis, 1, 2-methyl migration, stereoselective, allyl ethers

## Abstract

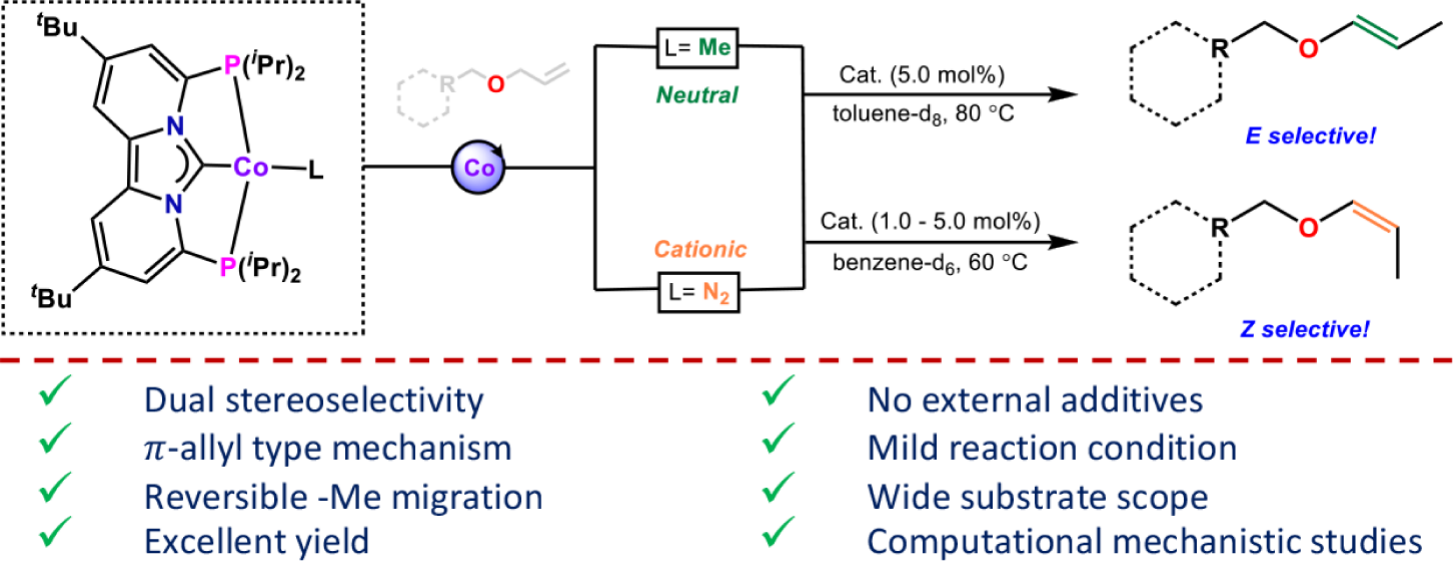

With growing efforts pushing toward sustainable catalysis, using earth-abundant metals has become increasingly important. Here, we present the first examples of cobalt PC_NHC_P pincer complexes that demonstrate dual stereoselectivity for allyl ether isomerization. While the cationic cobalt complex [((PC_NHC_P)Co)_2_-μ-N_2_][BAr_4_^F^]_2_ (**3**) mainly favors the *Z*-isomer of the enol ether, the corresponding methyl complex [(PC_NHC_P)CoMe] (**4**) mostly gives the *E*-isomer. The dichotomy in selectivity was investigated computationally, revealing important contributions from the substituents on the metal (N_2_ vs Me), including a 1,2-alkyl migration from cobalt to the N-heterocyclic carbene (NHC) of the methyl substituent, which is further explored in this report.

## Introduction

The carbon–carbon double bond is an important constituent of many organic compounds that are relevant in natural and industrial products.^1,2^ Among the variety of methods available to install it,^3−9^ the selective transposition of a carbon–carbon double bond has become an attractive choice for the synthesis of these important skeletal fragments.^10−13^ Typically, the transposition is catalyzed by precious metals, such as palladium,^14−19^ ruthenium,^20−28^ or iridium.^29−32^ However, in recent years environmentally friendly alternatives, based on earth-abundant metals, such as iron,^33−38^ cobalt,^39−51^ and nickel,^52−55^ have been developed as well.^56−58^ Irrespective of the metal, isomerization occurs by distinct mechanistic pathways that proceed via a (i) metal-allyl,^59,60^ (ii) metal–alkyl,^61,62^ or (iii) radical intermediate,^53,63,64^ which allows for diverse reaction profiles to be developed for the synthesis of these useful and value-added products.^65,66^

**Figure 1:**
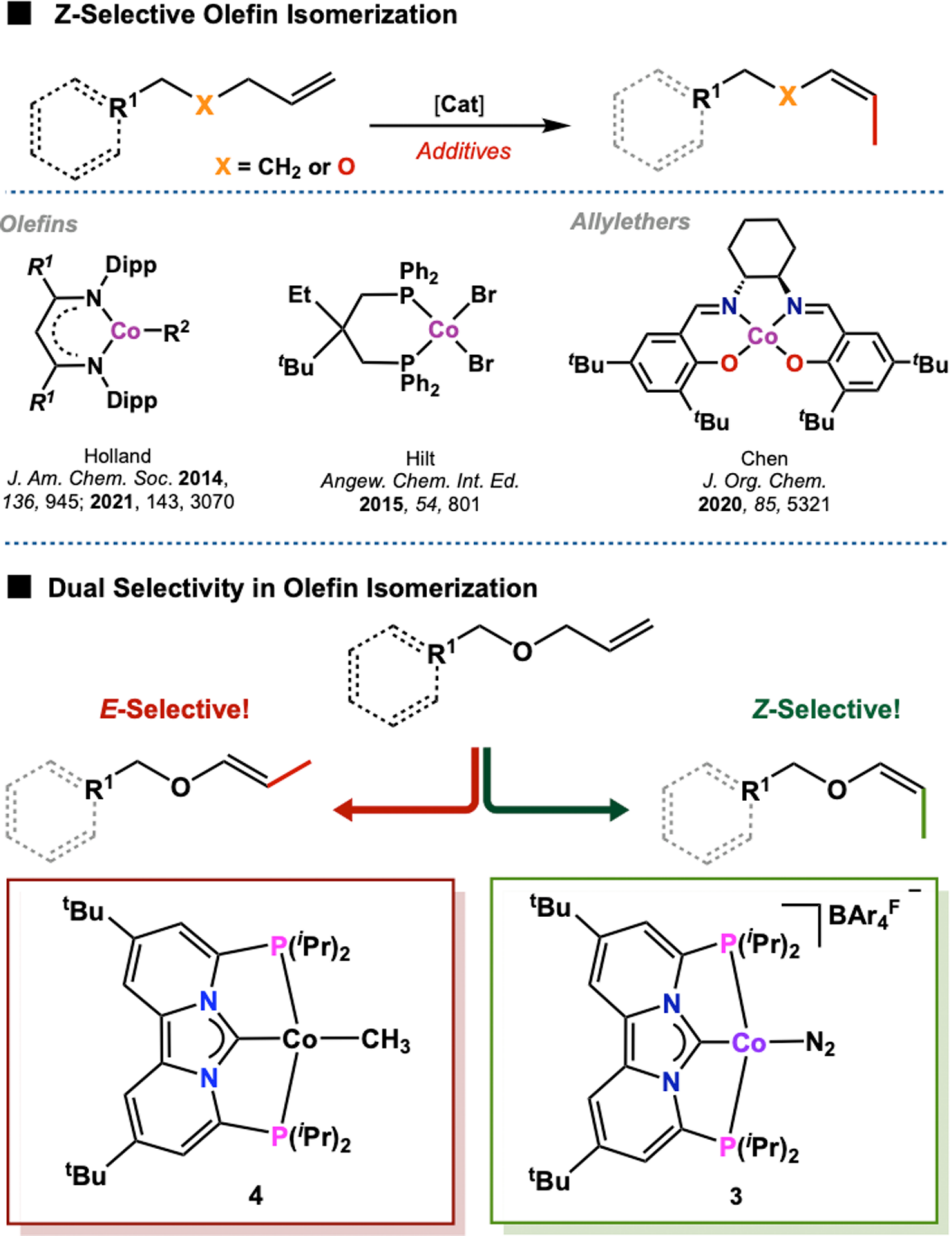
Selected examples of *Z*-selective cobalt catalyzed alkene isomerization, and the herein reported ligand effects for dual selectivity (*E* and *Z*) in allyl ether isomerization.

The type of mechanism, however, can have important consequences for the stereoselectivity of the reaction.^59^ In an alkyl-type mechanism, the stereoselectivity is usually determined during β-hydride elimination from a metal-alkyl species. However, discriminating between the nearly identical β-hydrogens is challenging, resulting in erosion of the stereoselectivity. By contrast, in an allyl-type mechanism, the differences in 1,2- or 1,3-allylic strain may be used to control the stereoselectivity. However, *Z*-alkenes are kinetically and thermodynamically disfavored,^67^ resulting in very few reported *Z*-selective alkene isomerization catalysts.^42,46,49,51,54,68^ (Figure 1). Mechanistically, almost all of the *Z*-selective alkene isomerization reactions proceed via an alkyl-type mechanism.

The origin of the observed selectivity is unfortunately often underexplored. In a rare example, Holland and co-workers determined that steric factors were mainly responsible for the observed *Z*-selectivity in alkene isomerization with a cobalt(II) alkyl complex.^51^ Interestingly, when the catalyst was exchanged for a cobalt(I) η^6^-arene species, not only was a switch to the more uncommon allyl mechanism observed, further mechanistic studies also revealed that a spin-state change of the cobalt metal center was responsible for the observed *Z*-selectivity.^42^ Besides these studies, mechanistic information regarding the origin of the observed stereoselectivity is limited in alkene isomerization, hampering the development of *Z*-selective alkene isomerization catalysts.

Recently, we reported the synthesis of an anionic iron(0) hydride complex supported by a PC_NHC_P pincer ligand with a N-heterocyclic carbene as a central donor.^34^ This complex proved to be extremely active for the selective one-bond isomerization of a variety of alkenes. Since our mechanistic studies indicated a spin-accelerated alkyl-type mechanism, we became interested in developing new reaction methodologies that rely on an allyl-type mechanism rather than an alkyl-type mechanism to improve *Z*-stereoselectivity. Herein, we present the first examples of cobalt PC_NHC_P pincer complexes (Figure 1) that show orthogonal selectivity (*E* vs *Z*) for allyl ether isomerization, despite having nearly identical ligands and overall structure. The isomerization occurs under moderate conditions and is compatible with a wide variety of functional groups. Experimental and computational studies were used to provide a better mechanistic understanding regarding the origins of the dichotomy between the stereoselectivity of complexes **3** (*Z*) and **4** (*E*). The experimental studies revealed a classical allyl-type mechanism and computational studies showed interesting ligand effects (N_2_ vs Me), where N_2_ dissociation in complex **3** favors a more *Z*-selective pathway for allyl ether isomerization. By contrast, a 1,2-alkyl migration of the methyl substituent from cobalt to the NHC carbon is invoked for the *E*-selective isomerization with complex **4**, which is further discussed in this report.

## Results and Discussion

### Synthesis of Cobalt PC_NHC_P Pincer Complexes

**Scheme 1:**
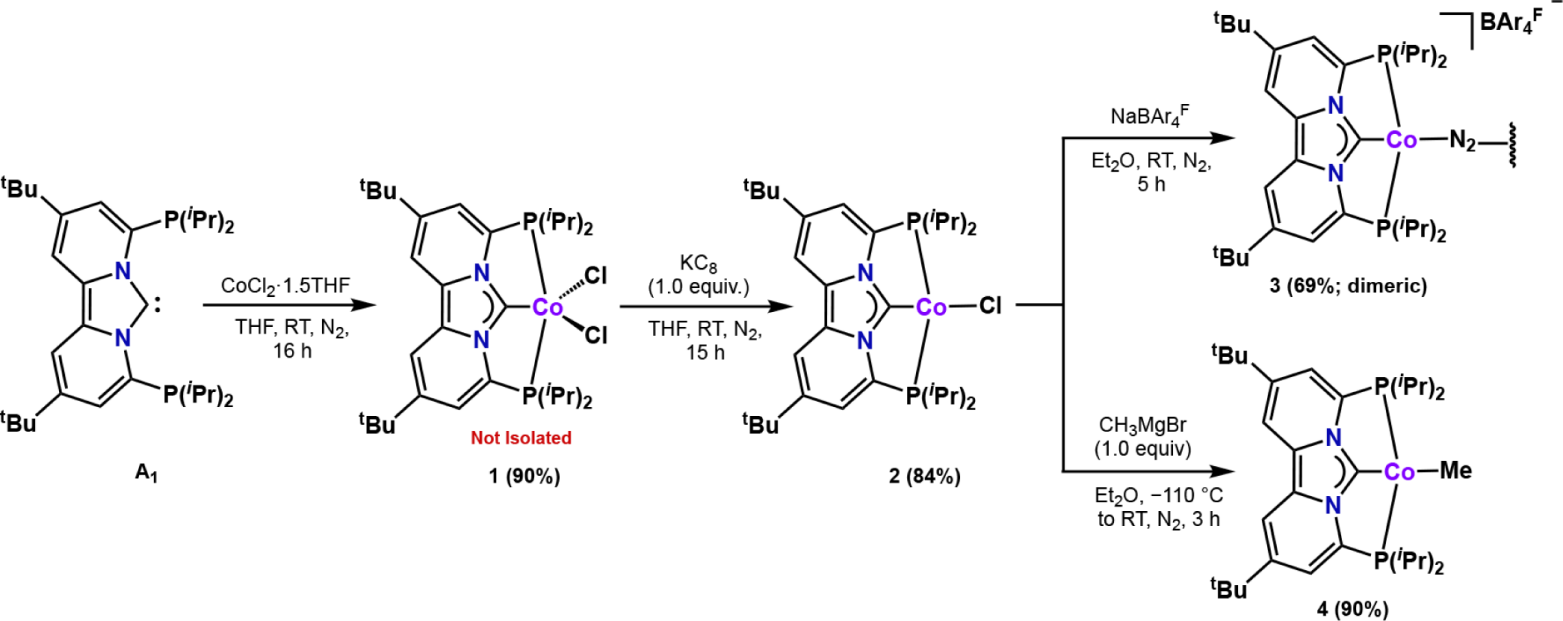
Synthesis of Cobalt Complexes **1**–**4**

**Figure 2:**
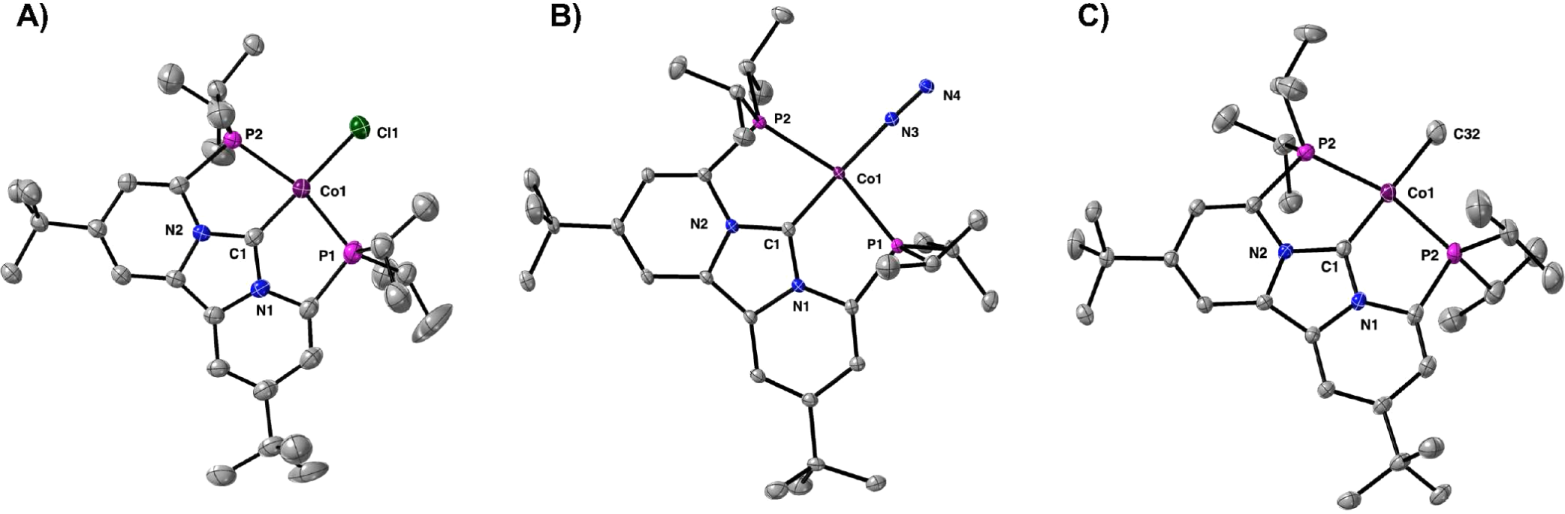
Solid-state structures of (A) [(PC_NHC_P)CoCl] (2), (B) [((PC_NHC_P)Co)_2_-μ-N_2_][BAr_4_^F^]_2_ (3), and (C) [(PC_NHC_P)CoMe] (4). Thermal ellipsoids are shown at the 50% probability level. Counter anions and cocrystallized solvent molecules are omitted for clarity. Complex 3 has been truncated to the monomer for comparison.

To develop the necessary methodology for *Z*-selective alkene isomerization, we focused our attention on our PC_NHC_P pincer ligands, which are an excellent platform for coordinating earth-abundant metals and for enabling unusual reactivity.^69−72^ Using a similar synthetic strategy to the one developed recently for our iron complexes,^71^ we were able to obtain the corresponding [(PC_NHC_P)CoCl_2_] complex **1**, which was used without further purification in the next step (Scheme 1). The addition of potassium graphite (KC_8_; 1.0 equiv) to the crude reaction mixture containing complex **1** resulted in a clean one-electron reduction to yield the diamagnetic cobalt(I) complex [(PC_NHC_P)CoCl] (**2**). The addition of sodium tetrakis[3,5-bis(trifluoromethyl)phenyl]borate (NaBAr_4_^F^) to a solution of **2** in diethyl ether (Et_2_O) resulted in facile abstraction of the chloride to furnish the cationic cobalt(I) dinitrogen complex **3**, which is a dimer in the solid state (Figure 2). Alternatively, the addition of 1.0 equiv of methyl-magnesium bromide (MeMgBr) to complex **2** furnished the diamagnetic cobalt(I) methyl species (**4**) in excellent yields (Scheme 1).

According to X-ray crystallography (Figure 2), complexes **2**–**4** all feature a cobalt metal center with square planar geometry. The Co–C1 carbene bond distances of 1.784(4) Å (**2**), 1.837(1) Å (**3**), and 1.798(2) Å (**4**) are quite short but comparable to other cobalt pincer complexes that feature a carbene donor.^73−76^ The Co–C1 distance in complex **3**, however, is slightly elongated compared to those in **2** and **4** due to competitive π-backbonding with the N_2_ ligand. Although dimeric, the two Co(I) centers in complex **3** do not activate the N–N bond (1.139(3) Å) to a great extent.^77^

Overall, the spectroscopic and structural data confirm the formation of well-defined Co(I) complexes with nearly identical bond metrics (Table S2). Because these complexes are iso-structural, we embarked on a comparative study into allyl ether isomerization.

### Allyl Ether Isomerization with Iso-Structural Cobalt(I) PC_NHC_P Pincer Complexes

Having fully characterized complexes **2**–**4**, we turned to investigate their activity in the isomerization of terminal allyl ethers for the following reasons (i) the corresponding enol ethers could be used in subsequent reactions such as the Coates-Claisen rearrangement^78^ and (ii) to the best of our knowledge, there has only been one report on the *Z*-selective isomerization of allyl ethers with earth-abundant metals (Figure 1).^46^ In this report, however, Chen and co-workers used stoichiometric amounts of Me_3_NFPy·OTf and Me_2_SiH_2_ to facilitate isomerization, severely limiting scalability and sustainability. In addition, the iso-structural nature of our complexes also allows us to establish important structure–function relationships that could affect the stereoselectivity of the isomerization reaction.

Because complex **2** only exhibits trace amounts of catalytic activity, only complex **3** and **4** were evaluated in this study. Using allylbenzyl ether as benchmark substrate, it was quickly established that the optimal conditions for positional alkene isomerization are 60 °C temperature with 1 mol % catalyst **3** and benzene-*d*_6_ as solvent (Table S1). Under these conditions, complete conversion of allylbenzyl ether to the corresponding enol ether was observed. Interestingly, the *Z*-isomer was predominantly detected in the reaction mixture (*E*/*Z* = 1:3). Gratifyingly, these conditions could also be applied to the isomerization of electronically and sterically differentiated allyl ethers (Table 1), all giving roughly the same *E*/*Z* ratio favoring the *Z*-isomer. For example, under the optimized conditions, allylbenzyl ethers containing both electron-donating (−OMe) and electron-withdrawing (−CF_3_) groups could be efficiently isomerized to their corresponding enol ethers **5d** and **5f**, with stereoselectivities of ≥1:3 (*E/Z*). *Ortho*-substituted allylbenzyl ethers are also well tolerated, with little to no influence of the substitution pattern on the yield or stereoselectivity of the reaction (Table 1; **5c** and **5e**). Linear aliphatic allyl ethers were also isomerized selectively with stereoselectivities similar to those of their benzyl counterparts (Table 1; **5i**–**5j**). Since disubstituted alkenes are not isomerized, selectivity is obtained when several double bonds are present in the substrate (Table 1; **5k** and **5l**). These enol ethers could subsequently be used for the Coates-Claisen rearrangement as beautifully illustrated by Marek and co-workers.^78^ Any other substitution on the allyl fragments leads to loss of catalytic activity, most likely due to steric crowding around the metal center.^34^ Sensitive functional groups such as epoxides and allyl silyl ethers are tolerated as well (Table 1**: 5n**–**5s**). Moreover, allyl ethers bearing natural product-derived substituents, such as bornane (**5q** and **5r**) and menthol (**5t**) are also isomerized efficiently. Overall, catalyst **3** exhibits a wide substrate scope and operates under mild conditions (60 °C, 5–16 h). Interestingly, while catalyst **4** presents a similar substrate scope (Table 1), the observed stereoselectivity is opposite to that of catalyst **3**, whereby now the *E*-isomer is observed as the major product. Although the *E*/*Z* ratios are modest, typically 2:1, the reversal of selectivity is remarkable, considering that the only structural change is replacement of −N_2_ for a methyl (−Me) substituent on the cobalt metal center.

**Table 1:**
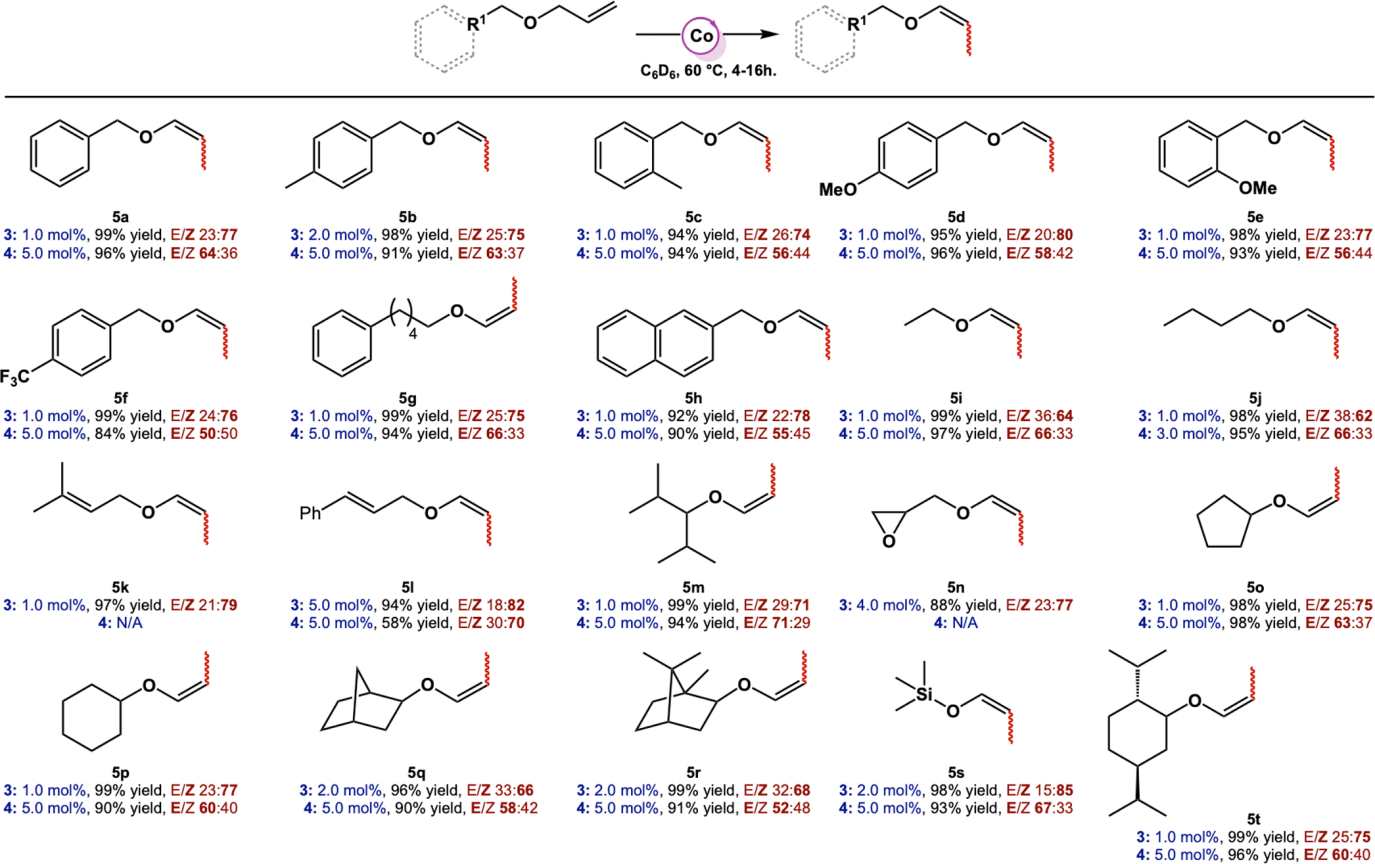
Isomerization of Allyl Ethers Catalyzed by Complexes 3 and 4.^a^^b^

a Reactions were performed with 1–5 mol % catalyst **3**, and 0.30 mmol alkene in 400 μL benzene-*d*_6_ for 5–16 h at 60 °C.

b Reactions were performed with 2–5 mol % catalyst **4**, and 0.15 mmol alkene in 400 μL toluene-*d*_8_ for 3–16 h at 80 °C. Yields and stereoselectivities were determined by ^1^H and ^13^C NMR spectroscopy.

To elucidate the mechanism of this reaction, we performed a series of deuterium labeling experiments.

For synthetic simplicity,^79^ we performed a crossover experiment with 3,3-*d*_2_-dec-1-ene and 4-allyl-1,2-dimethoxybenzene. Analysis of the reaction mixture revealed no scrambling of the deuterium label for either catalyst (Figures S91–S94). The absence of any scrambling is highly indicative of an allyl-type mechanism. Moreover, when 1,1-*d*_2_-dec-1-ene was used as substrate and complex **4** as catalyst, deuterium was primarily incorporated at the 1,3-position, not only supporting an allyl-type mechanism, but also indicating that the isomerization process is reversible (Figures S95–S100). In contrast, when catalyst **3** was used, deuterium was only incorporated marginally, indicating limited reversibility of the isomerization. In addition, monitoring the isomerization reaction as a function of time showed that for both catalysts **3** and **4** the *E*/*Z* ratios remained constant, indicating their formation is under kinetic control, although formation of the *E*-isomer is thermodynamically favored (*vide supra*). The same experiments also demonstrated that complexes **3** and **4** are stable under the reaction conditions, for instance the characteristic triplet for complex **4** at −0.14 ppm is clearly visible after the reaction is completed.

Finally, a radical-type mechanism was ruled out because the isomerization of allylbenzyl ether proceeded smoothly in the presence of known radical scavengers such as 9,10-dihydroanthracene, xanthene, or 1,1-diphenyl-ethylene (Figures S101–S112).^48^ Other radical scavengers, such as di-*tert*-butylhydroxytoluene or TEMPO, were not used as they interact with the metal center, producing catalytically inactive complexes.^34^ Furthermore, for substrates exhibiting a 1,6-diene motif, no cyclization was observed, as shown in Table 1 (**5k** and **5l**), ruling out a radical mechanism, as well. Overall, these experimental studies are consistent with an allyl-type mechanism for allyl ether isomerization, where the observed stereoselectivity is catalyst-dependent and does not result from ready interconversion of one isomer into the other (Figures S89 and S90).

### Computational Mechanistic Investigations

**Figure 3:**
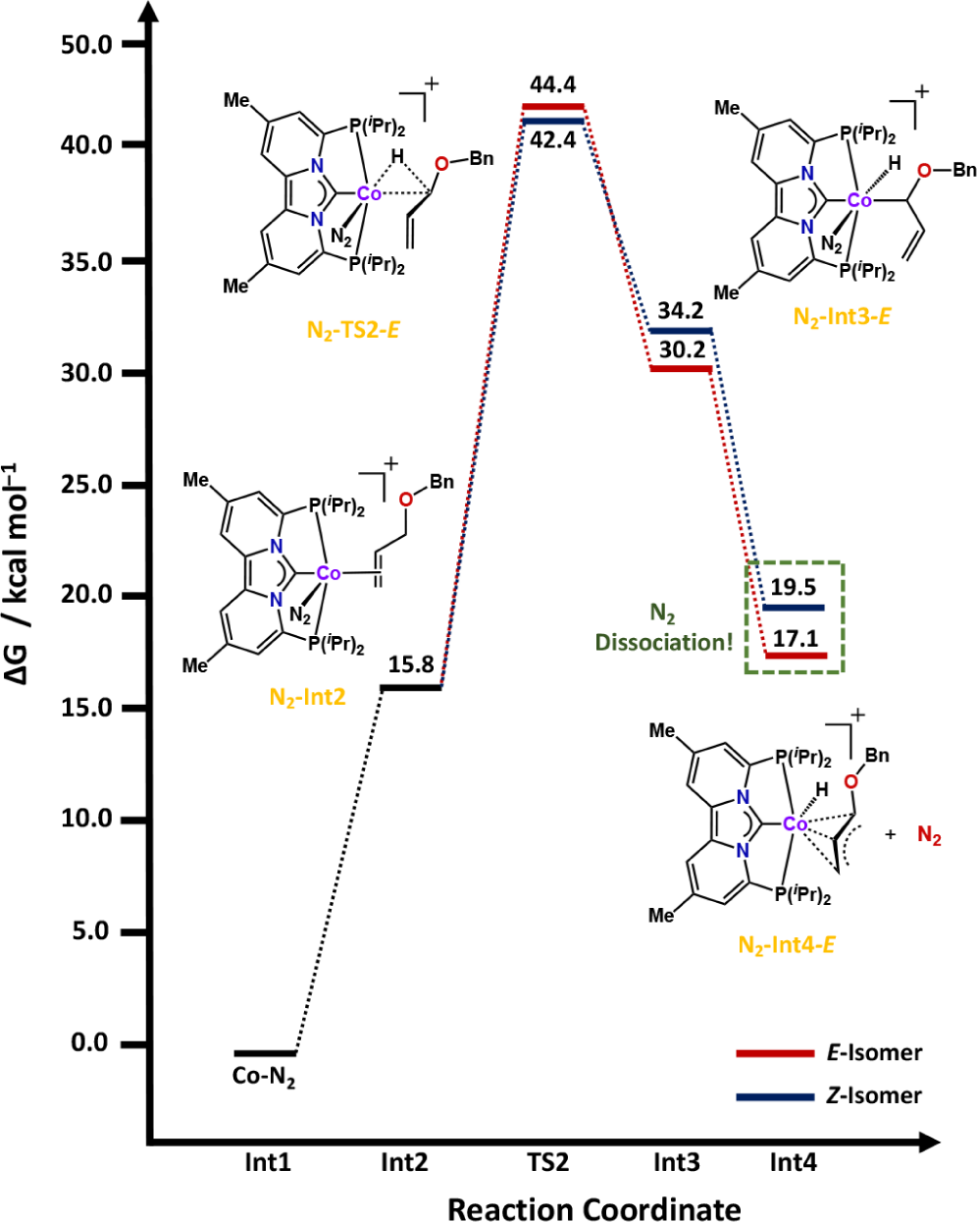
Calculated free energy profiles (Δ*G*) in kcal/mol at 333.15 K, for the isomerization of allylbenzyl ether with complex 3. See the Methods Section for more computational details.

**Figure 4:**
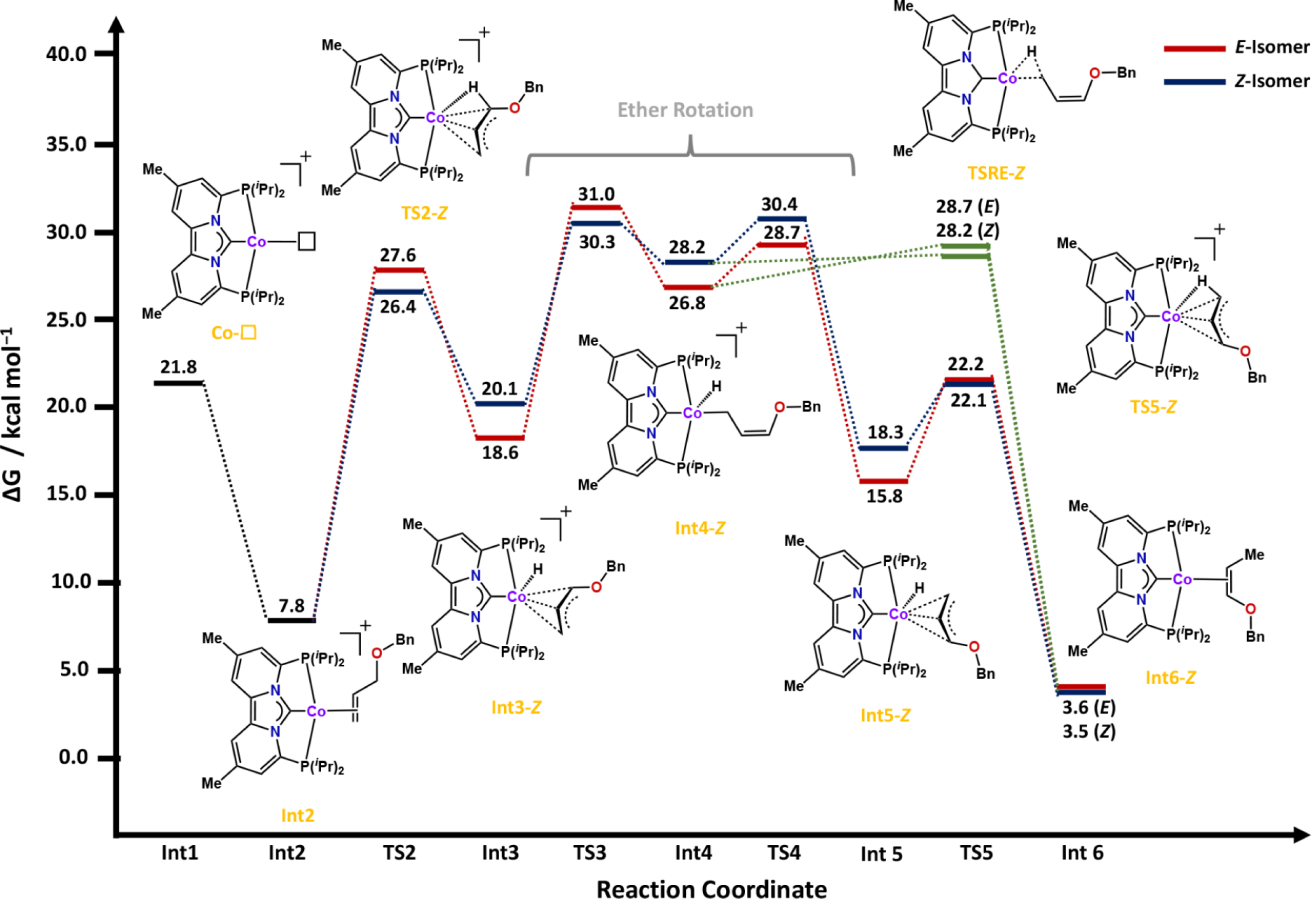
Calculated free energy profiles (Δ*G*) in kcal/mol at 333.15K, for the isomerization of allylbenzyl ether with complex 3 after N_2_ dissociation. See the Methods Section for more computational details.

**Figure 5:**
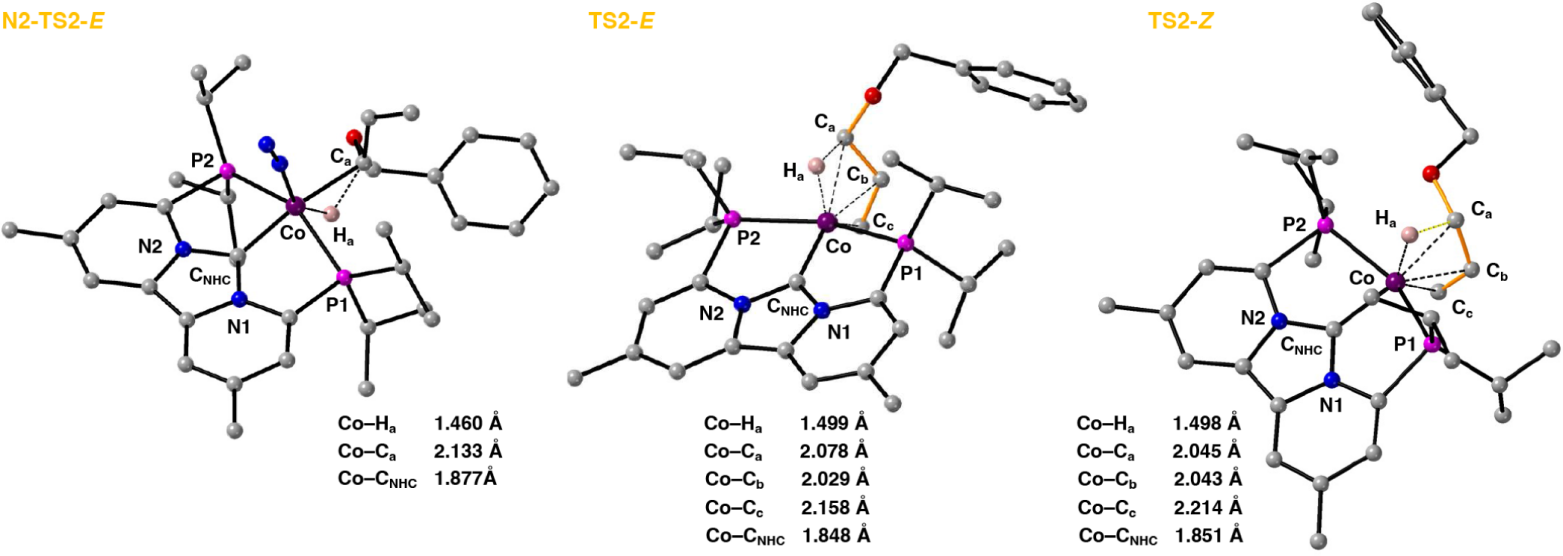
Optimized geometries of the calculated transitions states N2-TS2-*E* (A), TS2-*E*, and TS2-*Z* on the singlet potential energy surface. See the Methods Section for more computational details.

**Figure 6:**
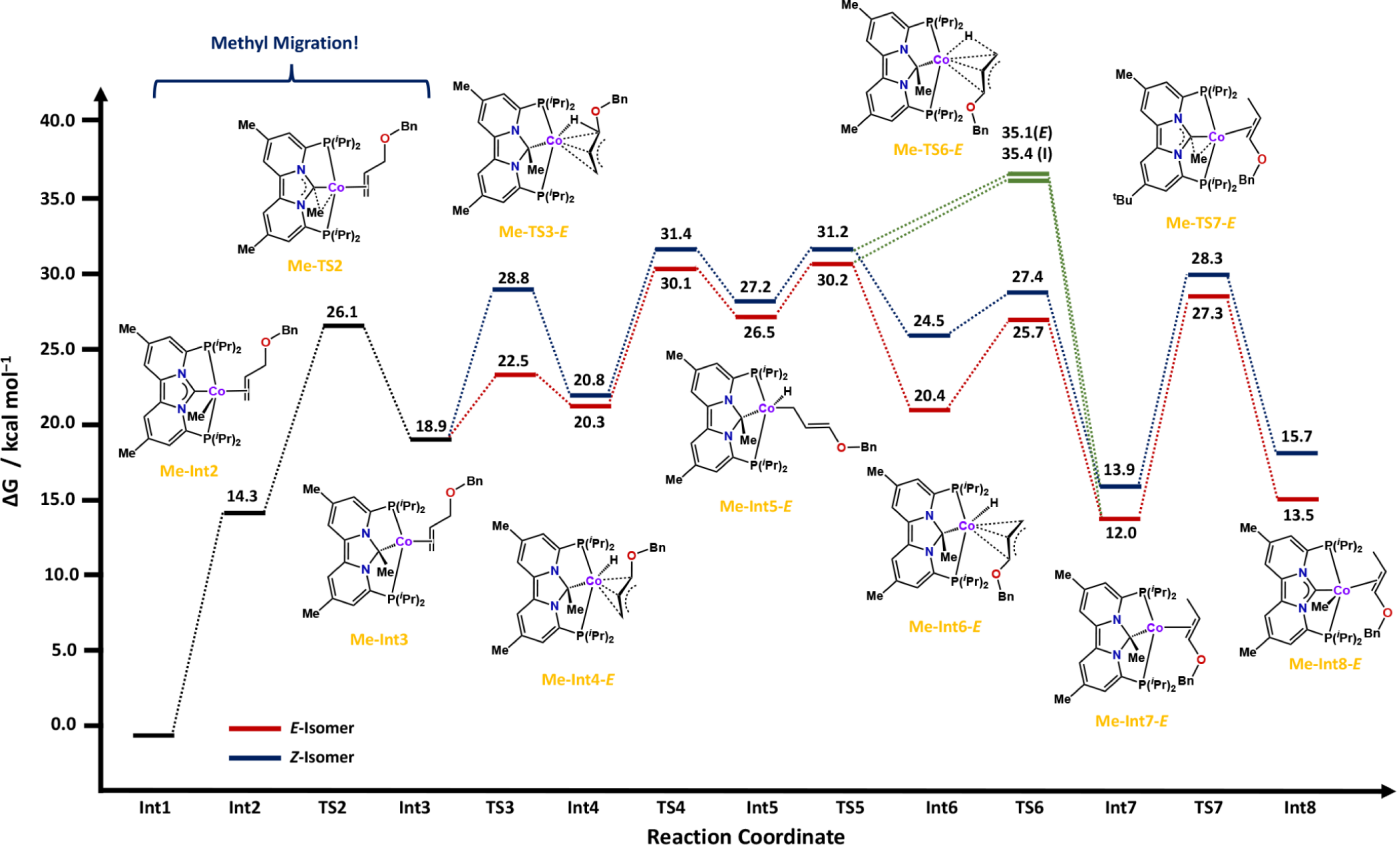
Calculated free energy profiles (Δ*G*) in kcal/mol at 353.15 K, for the isomerization of allylbenzyl ether with complex 4. See the Methods Section for more computational details.

To gain more insight into the origin of stereoselectivity and to identify the reasons for the reversal of stereoselectivity, we used density functional theory (DFT) calculations to characterize the relevant stationary points and transition-state structures along the reaction pathway. To alleviate computational complexity, calculations were performed with truncated models of the catalyst (Figures 3–6), in which the *tert*-butyl substituents on the backbone were replaced with methyl groups (see the Methods Section for computational details). The X-ray structures of complexes **3** and **4** were used to benchmark several levels of theory, following which we selected the PBE0-D3BJ/def2-SVP combination for C, H, N, O, and the PBE0-D3BJ/def2-TZVP level of theory for Co.^80−83^ The optimized geometries of **3** and **4** are shown in Figure S132, and the calculated structural parameters show good agreement with those determined by X-ray crystallography (Table S5).

We began our mechanistic evaluation by studying the isomerization of allylbenzyl ether with complex **3** in its monomeric form (Figure 3). Starting from complex **3**, coordination of the alkene is energetically uphill by 15.8 kcal/mol. Upon coordination of the allyl ether, the geometry around the cobalt metal center changes from square planar to distorted square pyramidal, which mainly results in an increase of the Co–N (N_2_) bond distance from 1.784 Å in **Co–N**_**2**_ to 1.865 Å in **N**_**2**_**–Int2**, due to decreased π-backbonding when N_2_ moves from an equatorial to an axial position. Thereafter, oxidative addition occurs via one of two distinct transition-states (**N**_**2**_**-TS2-*****E*** = 44.4 kcal/mol; **N**_**2**_**-TS2-*****Z*** = 42.4 kcal/mol), leading to either the *E*- or *Z*-isomer of the secondary cobalt alkyl complex **N**_**2**_**–Int3-*****E*** (30.2 kcal/mol) or **N**_**2**_**–Int3-*****Z*** (34.2 kcal/mol), respectively. As is evident from the transition-state energies, the *Z*-isomer is favored by 2.0 kcal/mol, which agrees with our experimental data. However, the calculated transition-state barriers of 44.4 and 42.4 kcal/mol are too high in energy to be accessible under the experimental reaction conditions. Even if these were accessible, isomerization of the secondary cobalt alkyl complex to the π-allyl intermediate results in a loss of the N_2_ ligand, changing the overall mechanistic and energetic landscape of the reaction. As a result, an alternative pathway was evaluated whereby N_2_ dissociates prior to coordination of the alkene (Figure 4).

Starting from **Co–N**_**2**_, dissociation of N_2_ results in the formation of a T-shaped cobalt complex (**Co-□**),^84^ which is energetically uphill by 21.8 kcal/mol. Coordination of the allyl ether stabilizes this coordinatively unsaturated species by 14.0 kcal/mol through formation of a cobalt-alkene complex (**Int2**). Alternatively, N_2_ could also dissociate from **N**_**2**_**–Int2**, (Figure 3), leading to the coordinated alkene complex **Int2** with an overall stabilization of 8.0 kcal/mol, providing a crossover between the N_2_-coordinated (Figure 3) and decoordinated pathways (Figure 4). From this square planar intermediate (**Int2**), oxidative addition occurs with transition-state barriers of 27.6 or 26.4 kcal/mol for the *E*- and *Z*-isomers, respectively. Thus, compared to the previously calculated transition-state barriers of 44.4 and 42.4 kcal/mol (*vide supra*), dissociation of the N_2_ ligand prior to oxidative addition lowers the respective barriers by approximately 16.8 kcal/mol for the *E*-isomer and by 16.0 kcal/mol for the *Z*-isomer. In both scenarios, the stereoselectivity of the oxidative addition is in accordance with our experimental results (Figure 4). The difference is, however, that dissociation of the additional N_2_-ligand allows for formation of the π-allyl interaction that stabilizes the transition-state, resulting in overall lowering of the energy barrier (Figure 5). As a result, when N_2_ dissociates, the product of C–H bond activation is not the secondary-alkyl hydride **N**_**2**_**–Int3*****E*****/*****Z***, but rather the more stable π-allyl hydride cobalt complex **Int3-*****E*****/*Z*** with a preferred *Z*-geometry of the coordinated η^3^-allyl (Figures 3**–**5). From π-allyl intermediate **Int3-*****E*****/*Z***, the coordinated allyl ether rearranges via transition states **TS3-*****E*****/*****Z*** to η^1^-allyl cobalt complexes **Int4-*****E*****/*Z***, respectively.

From these intermediates, two distinct pathways are possible. In the first pathway (Figure 4; blue and red traces), the η^1^-allyl isomers **Int4-*****E*****/*****Z*** revert to their η^3^-allyl counterparts, effectively accomplishing a 180° rotation of the allyl ether fragment (Figure 4; **Int3-*E*/*****Z*** vs **Int5-*E*/*****Z***). Interestingly, upon rotation, the *E*-isomer of the allyl ether fragment becomes more stable than its *Z*-congener (*E;* 15.8 kcal/mol vs *Z*; 18.3 kcal/mol). This energetic difference is preserved in the transition states for reductive elimination (**TS-5*E*/*Z***), leading to enol ethers **Int6-*****E***/***Z***. However, our experimental results demonstrate that allyl ether isomerization with complex **3** is not reversible (*vide supra*), signifying that the stereoselectivity is determined at the first oxidative additions step (Figure 4; **TS2**-***E***/***Z***), for which the *Z*-isomer is preferred by 1.2 kcal/mol. Furthermore, this mechanistic pathway also reveals that for both the *E*- and *Z*-isomers isomerization of the allyl fragment from an η^3^- to an η^1^-allyl intermediate has the highest transition-state barrier where again the Z-isomer is preferred by 0.7 kcal/mol (Figure 4, **TS3-*****E*** = 31.0 kcal/mol; **TS3-*****Z*** = 30.3 kcal/mol).

Alternatively, in the second pathway (Figure 4; green trace), the reductive elimination occurs immediately from the η^1^-allyl intermediates **Int4-*E*/*****Z*** to produce the corresponding enol ethers. The reductive elimination occurs with transition-state barriers of 28.7 (*E*) and 28.2 (*Z*) kcal/mol. These computational data thus suggest that regardless of the overall chosen pathway, formation of the *Z*-isomer is preferred, either by ΔΔ*G*^‡^= 1.2 kcal/mol (pathway 1) or by ΔΔ*G*^‡^= 0.5 kcal/mol (pathway 2),^85^ which is in good qualitative agreement with the experimentally observed preference for the *Z*-isomer (Table 1).

At this point, we wish to emphasize that the selectivity corresponds to differences in energies on the order of single kilocalories per mole (Figure 4), which are well within the error of DFT. However, it is our aim to provide qualitative chemical insight into the isomerization process, rather than predicting the exact energy of each stationary point and transition state. Notwithstanding, the obtained computational results are in good agreement with the experimental observations, with energies that are within the realm of our experimental conditions. Furthermore, they provide important insight into how the observed *Z*-stereoselectivity results from loss of the N_2_ ligand enabling the initial formation of an η^3^-allyl cobalt intermediate with a preferred *Z*-geometry. This *Z*-selectivity is preserved throughout the reaction through either (i) a series of ether rotations followed by reductive elimination or (ii) direct reductive elimination of the enol ether from a η^1^-allyl intermediate. Although the *Z*-enol ether is thermodynamically only marginally more stable than the *E*-isomer. The *Z*-isomer is also the kinetic product of the reaction whose formation is dictated by the initial oxidative addition, which is in good agreement with our experimental observations and our deuterium labeling experiments (*vide supra*).

Thus, far, our computational studies revealed the importance of the dissociation of the N_2_-ligand to enable the observed *Z*-selectivity. For cobalt complex **4**, however, such a dissociation is rather unlikely. However, reversible migration of the methyl substituent to the carbene carbon atom (C_NHC_) would generate a vacant coordination site, enabling the formation of an η^3^-allyl intermediate in an analogous manner to N_2_ dissociation. As such, the PC_NHC_P ligand would hereby be considered non-innocent. Such migrations, although rare, have been reported in the literature.^86−90^ For example, Fryzuk, Green and co-workers reported the migration of an ethyl-fragment from nickel to the NHC-carbon in a similar PC_NHC_P pincer ligand.^91^ Similarly, Bercaw and co-workers reported the reversible migration of a benzyl-substituent from zirconium to an NHC-carbon.^92^ Supporting these earlier observations, our computational studies also indicate that such a mechanism is indeed the lowest energy pathway toward isomerization with complex **4** (Figure 6).

The isomerization process starts with coordination of the allyl ether to the cobalt metal center, which is energetically uphill by 14.3 kcal/mol (Figure 6; **Me-Int2**). Hereafter, we identified two potential pathways: The first is a lower-energy pathway that involves migration of the methyl substituent from the cobalt metal center to the NHC carbon of the ligand with a transition state of 26.1 kcal/mol (Figure 6; **Me**-**TS2**). The second pathway involves oxidative addition of the allylic C–H bond with transition-state barriers of 44.8 and 42.7 kcal/mol leading to the *E*- and *Z*-isomers of the secondary cobalt alkyl complex, respectively (See Figure S133).

This second pathway (oxidative addition) is akin to that calculated for cationic intermediate **N**_**2**_**–Int2** and is likewise too high in energy to be feasible under the experimental reaction conditions. In addition, despite extensive efforts, we were unable to find the π-allyl transition-state allowing the η^3^-allyl to η^1^-allyl migration and enabling the cobalt metal center to move from the internal to the terminal carbon of the allyl ether. The reason is that the methyl ligand not only provides a steric barrier but also occupies one of the d-orbitals that is necessary for π-allyl formation. As a result, the methyl substituent must migrate from cobalt to NHC carbon to generate a vacant coordination site.

**Figure 7:**
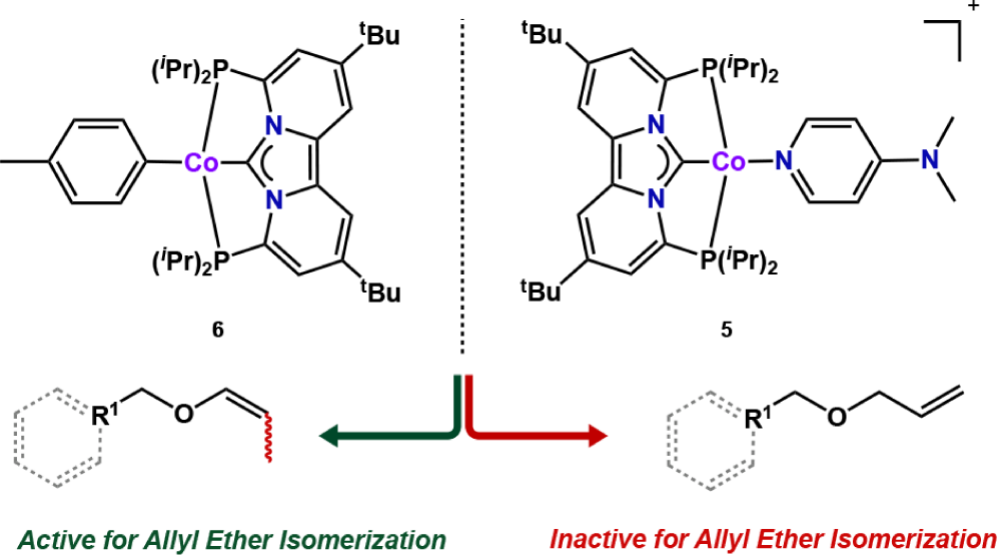
Cobalt complexes that are inactive (5) or active (6) for allyl ether isomerization, depending on the lability of the dimethylaminopyridyl or tolyl ligand (e.g., dissociation or migration).

The necessity for methyl migration was also supported by experimental studies. To illustrate, a cobalt complex containing the strongly coordinating dimethylaminopyridine group [(PC_NHC_P)CoDMAP] [BAr_4_^F^] (**5**), that is unlikely to dissociate or migrate, is unactive for allyl ether isomerization (Figure S118). By contrast, the structurally similar complex [(PC_NHC_P)Co(C_7_H_8_)] (**6**) is active for Z-selective allyl ether isomerization (Figure S122). These data are summarized in Figure 7 and strongly indicate that the generation of a vacant coordination site through alkyl/aryl migration is a necessary prerequisite for catalysis.

Returning to the first pathway, after migration of the methyl to the NHC (Figure 6**; Me-Int3**), oxidative addition of the allylic C–H bond readily occurs to produce the *E*- and *Z*-isomers of **Me-Int4** with transition-state barriers of 22.5 (*E*) and 28.8 (*Z*) kcal/mol. With the methyl group out of the way, isomerization occurs via a mechanism similar to that calculated for complex **3**. From the π-allyl intermediate **Me**-**Int4-***E*/***Z***, the coordinated allyl ether rearranges via an η^3^- to η^1^- to η^3^-interaction to effectively accomplish a 180° rotation of the allyl ether fragment (Figure 6; **Me**-**Int4-***E*/*Z* → **Me**-**Int6-***E*/*Z*). For both the *E*- and *Z*-isomers, isomerization to the η^1^-allyl intermediate (**Me-Int5-***E*/*Z*) is energetically the most demanding, with the *E*-isomer pathway being slightly lower in energy (Figure 5; compare **Me**-**TS4**-***E***: 30.1 kcal/mol vs **Me**-**TS4**-**Z:** 31.4 kcal/mol). Akin to complex **3**, direct reductive elimination from the η^1^-allyl intermediate **Me-Int5-***E*/*Z* to the enol ether intermediate **Me**-**Int6-***E***/***Z* is also possible, with transition-states barriers that are nearly identical in energy (Figure 6; **TSRE-***E*: 35.1 kcal/mol; **TSRE-***Z*: 35.4 kcal/mol). With the isomerization accomplished, the methyl-substituent now migrates back from the NHC carbon to the cobalt metal center via a transition-state with a barrier of 27.3 kcal/mol (*E*) or 28.3 kcal/mol (*Z*), completing the isomerization of the allyl ether to the enol ether (Figure 6; **Me-TS7-***E*/*Z*).

Comparing the isomerization of allyl ethers with complex **4** to that with complex **3** reveals one notable difference, besides the methyl migration. Namely, the transition state toward oxidative addition (**Me**-**TS3**) has the opposite stereoselectivity. Whereas for complex **3**, the *Z*-isomer is lower in energy, for complex **4** the *E*-isomer is lower in energy by about 6.3 kcal/mol (compare **TS2** and **Me**-**TS3**; Figures 4 and 5). The reversal is most likely due to the increased steric congestion around the metal center, even when the methyl has migrated to the NHC carbon. As a result, the *E*-isomer pathway is preferred across the entire reaction coordinate (Figure 6). This could explain the observed experimental differences in stereoselectivity, whereby our deuterium labeling studies indicate that allyl ether isomerization catalyzed by complex **4** is reversible (Figures S98–S100). As a result, the thermodynamically most stable product is expected. Indeed, here also the *E*-isomer is preferred by about 2.2 kcal/mol (Figure 6; compare **Me**-**Int8**-*E*/*Z*). Although these differences are within the error of DFT, these values nonetheless indicate that both isomers are expected at almost equal quantities, with a slight preference for the *E*-isomer, which agrees with our experimental data. More importantly, however, is the first demonstration of a reversible 1,2-methyl migration that is relevant for catalysis, which to the best of our knowledge is unprecedented in catalysis and yet has large consequences for the isomerization of allyl ethers.

Although the computational studies eloquently demonstrate a 1,2-methyl migration from cobalt to the NHC carbon, unfortunately, we have not been able to observe the migration experimentally. Addition of coordinating ligands of increasing steric congestion, either fail to bind to the metal center (e.g., PPh_3_ or IMes-NHC) or result in a dynamical equilibrium with the Co-Me complex (PMe_3_; Figures S123 and S124). Nevertheless, the lack of direct experimental evidence does not detract from the novelty of the herein reported reversible 1,2-methyl migration, which can serve as a stepping-stone for further research. Namely, we have shown that stabilizing ligands on the catalysts that can either dissociate (N_2_) or migrate (alkyl/aryl), are favorable for cobalt catalyzed allyl ether isomerization. These design principles can help direct research and experimental studies toward systems where such 1,2-alkyl migration can come to fruition in catalysis.

## Summary and Conclusions

In summary, we reported the synthesis of cobalt PC_NHC_P pincer complexes [(PC_NHC_P)Co(N_2_)][BAr_4_^F^] (**3**) and [(PC_NHC_P)Co(Me)] (**4**) that are active for the selective isomerization of a variety of sterically and electronically differentiated allyl ethers. The reaction occurs between 60 and 100 °C and shows preferences for either the *Z*-isomer (complex **3**) or *E*-isomer (complex **4**). Mechanistic and computational studies showed that the reaction occurs via an allyl-type mechanism, where the stereochemistry is determined by (i) the ability to form a stable π-allyl intermediate and (ii) the steric crowding around this π-allyl intermediate. As a result, for complex **3** dissociation of N_2_ is necessary to allow the formation of a η^3^-π-allyl intermediate that prefers the *Z*-isomer. In contrast, for complex **4**, such dissociation is not possible and allyl ether isomerization proceeds via a reversible 1,2-migration of the methyl substituent from the cobalt metal center to the NHC carbon, which enables the formation of the π-allyl intermediate with a preferred *E*-geometry. Overall, the isomerization occurs with good yields, and the computational studies provide ample benchmarking data for future ligand designs for *Z*-selective olefin isomerization.

## Methods

### General Procedures

All reactions were performed at room temperature either by using standard Schlenk techniques or by using a N_2_-filled MBraun or Vigor Gloveboxe unless otherwise specified. Glassware was oven-dried at 140 °C for at least 2 h prior to use, and allowed to cool under vacuum. All reagents were used as received unless mentioned otherwise. The PC_NHC_P carbene,^71^ CoCl_2_·1.5 THF,^93^ KC_8_ and NaBAr_4_^F^ were synthesized according to literature procedures.^94,95^ CH_3_MgBr in Et_2_O (3M), CH_3_Li in Et_2_O (1.6M), allyl ethyl ether, allyl butyl ether, allyl trimethylsilyl ether, 1-decene, 4-allyl-1,2-dimethoxybenzene, 1,1-diphenylethylene, 9,10-dihydroanthracene, xanthene, anhydrous unstabilized tetrahydrofuran (THF), pentane, and diethyl ether (Et_2_O) were all purchased from Sigma-Aldrich and used as received. allyl benzyl ether,^96^ allyl 4-methylbenzyl ether,^97^ allyl 2-methylbenzyl ether,^98^ allyl 4-methoxybenzyl ether,^99^ allyl 2-methoxybenzyl ether,^100^ 2-[(2-propenyloxy)methyl)naphthalene,^101^ 1-[(2-propen-1-yloxy)methyl]-4-(trifluoromethyl),^96^ 3-(allyloxy)-2,4-dimethylpentane,^102^ [(1E)-3-(2-propen-1-yloxy)-1-propen-1-yl]benzene,^103^ 3-Methyl-1-(2-propen-1-yloxy)-2-butene,^104^ allyl glycidal ether,^105^ (allyloxy)cyclopentane,^102^ (allyloxy)cyclohexane,^102^ (1S,2R,4R)-2-(allyloxy)-1-isopropyl-4-methylcyclo-hexane,^102^ (1S, 2S, 4R)-2-(allyloxy)bicyclo[2.2.1]-heptane,^102^ (1S,2R,4S)-2-(allyloxy)-1,7,7-trimethyl-bicyclo[2.2.1]heptane,^102^ dec-1-ene-3,3-*d*_2_,^106^ and do-dec-1-ene-1,1-*d*_2_^107^ were synthesized according to literature procedures. The ^1^H, ^2^H, ^13^C {^1^H}, and ^31^P NMR spectra were recorded on Bruker AVANCE III 200, 300, 400, and 500 NMR spectrometers at room temperature, unless mentioned otherwise. All chemical shifts (δ) are reported in parts per million, and coupling constants (*J*) are in Hz. The ^1^H and ^13^C {^1^H} NMR spectra were referenced by using residual solvent peaks in the deuterated solvent. The ^31^P chemical shifts are reported relative to the internal lock signal. Deuterated solvents (CDCl_3_, benzene-*d*_*6*_, toluene-*d*_*8*_ and THF-*d*_8_) were purchased from Cambridge Isotope Laboratories, dried over calcium hydride, degassed by three freeze–pump–thaw cycles, and vacuum-transferred prior to use. Elemental Analysis were performed by Kolbe Microanalytical laboratory in Oberhausen (Germany).

### Physical Methods

#### Single-Crystal X-Ray Diffraction

For compounds 2 and 4, low temperature (100 K) diffraction data were collected using a Rigaku Synergy R 4-circle Kappa goniometer with rotating anode generator, Cu Kα (λ = 1.54184 Å) radiation, equipped with a HyPix-Arc 150 detector. For compound 3, low temperature (100 K) diffraction data were collected on Rigaku Synergy R X-ray 4-circle Kappa goniometer with rotating anode generator Mo Kα, (λ = 0.71073 Å) radiation equipped with a HyPix-Arc 100 detector. All crystals were coated in Hampton Parbar Cryoprotectant oil, mounted on a MiTeGen loop and flash frozen in the liquid nitrogen gasous stream of Oxford Cryosystem prior to x-ray data collection. All Rigaku diffractometer manipulations, including data collection, integration, and scaling and absorption corrections were done with CrysAlisPro 1.171.42.94a (Rigaku OD, 2023). All structures were solved by direct methods using SHELXT 2018/4^110^ and refined against *F*^2^ on all data by full-matrix least-squares with SHELXL-2016 or SHELXL-2018^111^ using established refinement techniques.^112^ All non-hydrogen atoms were refined anisotropically. All hydrogen atoms were included in the model at geometrically calculated positions and refined using a riding model. The isotropic displacement parameters of all hydrogen atoms were fixed to 1.2 times the *U* value of the atoms they are linked to (1.5 times for methyl groups).

### Computational Methods

The density functional theory (DFT) calculations were performed using Gaussian 09, Revision D.01.^113^ Geometry optimization and frequency calculations for all systems were carried out using the PBE0^83^ functional with Grimme’s empirical D3 dispersion correction with revised Becke–Johnson damping parameters,^80−82^ in combination with the basis set def2SVP for C, H, N, P, O, and def2TZVP for Co. The optimized minima and transition states were confirmed through harmonic vibrational analysis, ensuring that the minima exhibited no imaginary frequencies, and the transition states displayed only one appropriate imaginary frequency. For all stationary points, we performed single-point calculations at the PBE0-D3BJ/def2TZVP level of theory including solvation calculations using the SMD model,^114^ implemented in Gaussian 09 with benzene as a solvent to reproduce the reaction conditions. The level of theory was selected following a benchmarking procedure (see below) using the crystal structures of complexes **3** and **4**. Likewise, all stationary points were prepared by using the crystal structures of complexes **3** and **4** as the initial geometries. The intermediates and transition states may adopt various conformations due to the flexibility of the substrate allylbenzyl ether. To ensure that the entire conformation space was explored, we used CREST (Conformer-Rotamer Ensemble Sampling Tool).^200^ The (up to five) lowest-energy conformers according to CREST were further optimized using DFT to identify the global minimum. These selected structures were then employed as the main intermediates and transition states in mechanistic investigations. All energies discussed in this work are Gibbs free energies, defined as the sum of electronic energy, solvation correction, and thermal correction to Gibbs free energy at 333.15 K with concentration correction (from standard state in gas phase, 1 atm, to standard state in solution, 1 mol/L), according to eq 1:
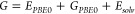
1where *E*_*PBE*0_ is the potential energy resulting from calculations with PBE0-D3BJ, *G*_*PBE*0_ is the thermal correction to the Gibbs free energy, calculated at the optimization level at 333.15 K including concentration correction (from the standard state in the gas phase, 1 atm, to the standard state in solution, 1 mol/L), and *E*_*solv*_ is a solvent correction calculated at the M052X-D3 level of theory with the basis set def2SVP for C, H, N, P, O, and def2TZVP for Co. To achieve the highest accuracy in calculating Gibbs free energies, we employed the M052X-D3 functional. This choice was driven by the fact that the universal solvation model, which is based on the quantum mechanical charge density of a solute molecule interacting with a continuum solvent description, was parametrized using two functionals: M052X^115^ and B3LYP.^116^ Thermal correction to Gibbs free energy at 333.15K and concentration correction were calculated using GoodVibes; a Python package to compute thermochemical data from electronic structure.^117^

DFT has been applied to nearly all transition metals in diverse fields, such as organic/inorganic synthesis, homogeneous catalysis, material properties, molecular biology, and medicine. Zhao and Truhlar proposed the concept of an ideal functional suitable for all chemical and physical applications,^118^ though such a functional is unlikely to be discovered in the near future. Therefore, functionals must be designed to achieve the broadest possible accuracy across key criteria: transition-metal bonding, thermochemistry, electronic spectroscopy, barrier heights, and weak interactions.

Unfortunately, it is highly unlikely that one will find a functional that reproduces all of these within chemical accuracy. This is especially true when attempting to characterize intermediates and transition states that are very close in energy. Small energy differences can have dramatic effects on selectivity (a 1.4 kcal/mol difference in energy leads to a 90:10 product distribution) but are also very challenging to replicate when the margin of error of a calculation is a few kcal/mol (in the best-case scenarios). Considering the difficulties mentioned above, we were particularly gratified to reproduce the *E*/*Z* selectivity observed experimentally. However, the agreement is not quantitative, which is very clearly seen in the calculated transition-state barriers-they are higher than expected, given that the reaction proceeds at 60 °C. This may be because our cobalt complexes have partially filled d-orbitals, leading to strong electron correlation effects and artificial electron delocalization due to self-interaction error.^119,120^ Such effects are not captured by the DFT methods well enough. Multireference methods can be applied to overcome these limitations; however, these methods are computationally very expensive, and for the size of our systems, perhaps even infeasible to perform.^121^ Overall, in our case, the cost-benefit ratio favors using DFT.

### Synthetic Procedures

#### Synthesis of [(PC_NHC_P)CoCl_2_] (**1**)

In the glovebox, a suspension of CoCl_2_·1.5THF (352 mg, 1.48 mmol), in THF (15 mL) was added dropwise to a stirred yellow solution of the PC_NHC_P (free carbene **A1**, 830 mg, 1.62 mmol) in THF (20 mL) at room temperature. The resulting deep brown mixture was stirred for an additional 16 h, whereafter the solvent was evaporated under reduced pressure to yield a crude brown solid. The crude solid was washed with hexane (2 × 10 mL) and without further purification, it was used for the next step. Yield 856 mg (90%). HRMS (TOF MS ES^+^, positive ion, *m*/*z*): calcd. For [C_31_H_50_ClCoN_2_P_2_]^+^; 606.2470, found 606.2469.

#### Synthesis of [(PC_NHC_P)CoCl] (**2**)

In the glovebox, to a thawing frozen suspension of [(PC_NHC_P)CoCl_2_] (**1**; 275 mg, 0.43 mmol) in THF (20 mL) was added dropwise a frozen suspension of KC_8_ (58 mg, 0.43 mmol) in THF (2 mL). The resulting reaction mixture was stirred for 30 min at −78 °C and for an additional 15 h at room temperature. Hereafter, the solvent was removed under reduced pressure, and the obtained black-purple solid was redissolved in diethyl ether (10 mL) and filtered through a pad of Celite, which was washed with an additional amount of diethyl ether (5 mL). The filtered mixture was concentrated under reduced pressure to yield a crude purple solid. The title compound was obtained as purple crystals from a concentrated ether solution at room temperature. Yield: 218 mg (84%). **^1^H NMR (400 MHz, benzene-*d*_6_)**: δ (ppm) 6.94 (s, 2H, *m*-bpy-H), 6.73 (s, 2H, *m*-bpy-H), 2.66–2.54 (br, m, 4H, (CH_3_)_2_CH), 1.62 (dd, *J* = 15.5, 7.3 Hz, 12H, (CH_3_)_2_CH), 1.33 (dd, *J* = 13.5, 6.2 Hz, 12H, (CH_3_)_2_CH), 1.14 (s, 18H, ^t^Bu). **^13^C {^1^H} NMR (100 MHz, benzene-*d*_6_)**: δ (ppm) 140.8, 137.5 (t, ^1^*J*_P–C_ = 14.9 Hz), 119.0 (t, ^2^*J*_P–C_ = 2.2 Hz), 114.3, 112.6, 34.9, 30.4, 25.7 (^1^*J*_P–C_ = 6.8 Hz), 19.4 (t, ^2^*J*_P–C_ = 2.9 Hz), 19.0 (*carbene carbon signals were not observed between −20 and 220 ppm*). ^31^P {^1^H} NMR (162 MHz, benzene-*d*_6_): δ (ppm) −79.8 (br, s). **Elemental Analysis**: Anal. Calcd for [C_31_H_50_N_2_P_2_CoCl] C, 61.33; H, 8.30; N, 4.61. Found: C, 61.19; H, 8.27; N, 4.59.

#### Synthesis of [(PC_NHC_PCo)_2_(μ-N_2_)](BAr_4_^F^)_2_ (**3**)

In the glovebox, to a thawing frozen suspension of **2** (132 mg, 0.22 mmol) in diethyl ether (8 mL) was added dropwise a solution of NaBAr_4_^F^(THF)_6_ (348.2 mg, 0.26 mmol) in diethyl ether (2 mL). The resulting blue mixture was allowed to warm to room temperature, and the mixture was stirred for an additional 5h. Hereafter, the reaction mixture was filtered through a pad of Celite, which was washed with extra diethyl ether (5 mL) to remove precipitated NaCl. The filtered deep blue solution was concentrated under reduced pressure to yield a crude blue solid. The title compound was obtained as dark blue crystals from a concentrated ether solution at room temperature. Yield: 223 mg (69%). ^**1**^**H NMR (400 MHz, THF-***d*_8_**)**: δ (ppm) 8.08 (br, s, 0.9H, *m*-bpy-H), 7.79 (s, 8H, BAr_4_^F^-H), 7.65 (br, s, 2H, *m*-bpy-H), 7.57 (s, 4H, BAr_4_^F^-H), 7.26 (br, s, 1.1H, *m*-bpy-H), 3.10–2.84 (br, m, 1.8H, (CH_3_)_2_CH), 2.84–2.57 (br, m, 2.2H, (CH_3_)_2_CH), 1.54–1.21 (m, 42H, (CH_3_)_2_CH and ^t^Bu). ^**13**^**C {**^**1**^**H} NMR (101 MHz, THF-***d*_8_**)**: δ (ppm) 162.8 (q, ^1^*J*_*B–C*_ = 49.9 Hz), 135.6, 130.2 (qq, ^2^*J*_*C–F*_ = 31.8, ^4^*J*_*C–F*_ = 2.7 Hz), 125.4 (q, ^1^*J*_*C–F*_ = 272.2 Hz), 118.3–118.0 (br, m), 36.0 (br), 30.6, 27.02 (br), 19.7 (br, s), 19.1 (carbene carbon signals were not observed between −20 and 220 652 ppm). ^**31**^**P{**^**1**^**H} NMR (162 MHz, THF-***d*_8_**)**: δ (ppm) 91.7 (br, s), 73.3 (br, s). **Elemental Analysis**: Anal. Calcd for [C_126_H_126_B_2_Co_2_F_48_N_6_P_4_] C, 52.19; H, 4.38; N, 2.9. Found: C, 52.11; H, 4.35; N, 2.87.

#### Synthesis of [(PC_NHC_P)Co(CH_3_)] (**4**)

**Method A**; from **1**. In the nitrogen-filled glovebox, a suspension of [(PC_NHC_P)CoCl_2_] (**1**; 260 mg, 0.41 mmol) in THF (15 mL) was cooled to −110 °C, whereafter a diluted solution of CH_3_MgBr (3M, 433 μL, 1.3 mmol) in THF (2 mL) was added dropwise. The resulting deep purple mixture was allowed to warm to room temperature, and the mixture was stirred for another 3h, after which the solvent was evaporated under reduced pressure. The purple residue was suspended in Et_2_O (10 mL) along with few drops of 1,4-dioxane and the resulting mixture was stirred for 30 min. Hereafter, the volatiles were removed under reduced pressure, and the residue was suspended in pentane (15 mL), which was subsequently filtered through a pad of Celite. The filtrate was collected and concentrated under reduced pressure to afford a deep purple solid. This filtration process was repeated one more time to remove all of the insoluble particulates. The title compound was obtained as X-ray quality crystals from a concentrated pentane solution at room temperature. Yield: 216 mg (90%). ^**1**^**H NMR (400 MHz, benzene-***d*_6_**)**: δ (ppm) 6.98 (s, 2H, *m*-bpy-H), 6.81 (s, 2H, *m*-bpy-H), 2.73–2.61 (br, m, 4H, (CH_3_)_2_C*H*), 1.5 (dd, *J* = 15.2, 7.3 Hz, 12H, (CH_3_)_2_CH), 1.31 (dd, *J* = 13.1, 6.7 Hz, 12H, (CH_3_)_2_CH)), 1.20 (s, 18H, tBu), −0.14 (t, *J* = 8.2 Hz, 3H, Co–CH_3_). ^**13**^**C {**^**1**^**H} NMR (101 MHz, benzene-***d*_6_**)**: δ (ppm) 140.5 (t, ^1^*J*_P–C_ = 14.3 Hz), 140.3, 118.4 (t, ^2^*J*_P–C_ = 2.8 Hz), 113.0, 112.2, 34.9, 30.5, 25.8 (t, ^1^*J*_P–C_ = 6.2 Hz), 19.6 (t, ^2^*J*_P–C_ = 3.4 Hz), 19.7 (*carbene carbon and Co-CH*_*3*_*carbon were not observed between −20 and 220 ppm*). ^**31**^**P {**^**1**^**H} NMR (162 MHz, benzene-***d*_6_**)**: δ (ppm) 91.3 (br, s). **Elemental Analysis**: Anal. Calcd for [C_32_H_53_CoN_2_P_2_**·** 0.35 C_4_H_8_O_2_] C, 64.97; H, 9.11; N, 4.78. Found: C, 64.96; H, 9.16; N, 4.59.

**Method B** (from **2**) In the nitrogen-filled glovebox, to a thawing frozen suspension of [(PC_NHC_P)CoCl] (**2**; 150 mg, 0.25 mmol) in Et_2_O (10 mL) was added dropwise a diluted solution of MeLi (1.6 M; 0.16 mL, 0.25 mmol) in Et_2_O (5 mL). The resulting deep purple mixture was allowed to warm to room temperature, and it was stirred for an additional 3h, after which the volatiles were removed under reduced pressure. The resulting crude purple solid was suspended in pentane (15 mL) and filtered through a pad of Celite. This process was repeated one more time to remove any insoluble particulates. The title compound was obtained as X-ray quality crystals from a concentrated pentane solution at room temperature. Yield: 126 mg (86%). Characterization data are identical to those of method A.

#### Synthesis of [(PC_NHC_P)Co(DMAP)](BAr_4_^F^) (5)

In the nitrogen-filled glovebox, [((PC_NHC_P)Co)_2_-μ-N_2_](BAr_4_^F^)_2_ (**3**; 100 mg, 0.035 mmol) was dissolved in THF (5 mL) and 4-dimethylaminopyridine (9 mg, 0.07 mmol) was added. The mixture was stirred at room temperature for 1 h then it was evaporated under reduced pressure to yield the title compound as a purple solid. Yield: 98 mg (90%). ^**1**^**H NMR (400 MHz, THF-***d*_8_**)**: δ (ppm) 8.15 (bs, 2H, py-H), 7.80 (s, 8H, BAr_4_^F^-H), 7.64 (s, 2H, *m*-bpy-H), 7.58 (s, 4H, BAr_4_^F^-H), 7.22 (s, 2H, m-bpy-H), 6.67 (bs, 2H, py-H), 3.00 (s, 6H, −N(CH_3_)_2_), 2.70–2.57 (br, m, 4H, CH_3_)_2_CH), 1.40–1.32 (m, 30H, (CH_3_)_2_CH and ^t^Bu), 1.14 (dd, *J* = 15.9, 7.4 Hz, 12H, (CH_3_)_2_CH). ^**13**^**C {**^**1**^**H} NMR (101 MHz, THF-***d*_8_**)**: δ (ppm) 162.9 (q, ^1^*J*_*B–C*_ = 49.7 Hz), 154.9, 151.0, 135.7, 134.7 (t, ^1^*J*_*P–C*_ = 19.5 Hz), 130.1 (qq, ^2^*J*_*C–F*_ = 31.7, ^4^*J*_*C–F*_ = 2.8 Hz), 125.6 (q, ^1^*J*_*C–F*_ = 272.3 Hz), 121.4 (br, s), 118.3 (br, s), 114.45, 109.0, 39.0, 36.0, 30.6, 25.9 (t, ^1^*J*_*P–C*_ = 7.7 Hz), 19.1 (t, ^2^*J*_*P–C*_ = 2.9 Hz), 19.0 (*carbene carbon signals were not observed between −20 and 220 ppm*). ^**31**^**P{**^**1**^**H} NMR (162 MHz, THF-***d*_8_**)**: δ (ppm) −79.5 (br, s).

#### Synthesis of [(PC_NHC_P)Co(p-tol)] (6)

In the nitrogen-filled glovebox, a suspension of [(PC_NHC_P)CoCl] (**2**; 100 mg, 0.17 mmol) in THF (5 mL) was frozen in cold well, whereafter a diluted solution of *p*-tolylmagnesium bromide (1M, 182 μL, 0.18 mmol) in THF (2 mL) was added dropwise. The resulting deep purple mixture was allowed to warm to room temperature and the mixture was stirred for another 3h, after which the solvent was evaporated under reduced pressure. The purple residue was suspended in Et_2_O (10 mL) along with few drops of 1,4–dioxane and the resulting mixture was stirred for 30 min. Hereafter, the volatiles were removed under reduced pressure and the residue was suspended in pentane (15 mL) The resulting suspension was subsequently filtered through a pad of Celite. This process was repeated one more time to remove any insoluble particulates. Hereafter, the filtrate was collected and concentrated under reduced pressure to afford a deep purple solid. The title compound was obtained as X-ray quality crystals from a concentrated pentane solution at room temperature. Yield: 98 mg (87%). ^**1**^**H NMR (400 MHz, benzene-***d*_6_**)**: δ (ppm) 7.55 (d, *J* = 7.7 Hz, 2H, *o*–Ar-H), 7.24 (d, *J* = 7.7 Hz, 2H, *m*–Ar-H), 7.01 (s, 2H, *m*-bpy-H), 6.80 (s, 2H, *m*-bpy-H), 2.65 (q, *J* = 7.5 Hz, 4H, (CH_3_)_2_CH), 2.44 (s, 3H, Ar–CH_3_), 1.33–1.15 (m, 42H, (CH_3_)_2_CH), ^t^Bu). ^**13**^**C {**^**1**^**H} NMR (101 MHz, benzene-***d*_6_**)**: δ (ppm) 140.7, 140.3 (t, ^1^*J*_P–C_ = 13.2 Hz), 138.4 (t, ^2^*J*_P–C_ = 2.4 Hz), 127.1, 126.7, 119.0 (t, ^2^*J*_P–C_ = 2.9 Hz), 113.7, 112.2, 35.0, 30.5, 25.0 (t, ^1^*J*_P–C_ = 7.0 Hz), 21.7, 19.3 (t, ^2^*J*_P–C_ = 3.1 Hz), 18.8 (*carbene carbon and Co-CH*_*3*_*carbon were not observed between −20 and 220 ppm***)**. ^**31**^**P {**^**1**^**H} NMR (162 MHz, benzene-***d*_6_**)**: δ (ppm) 91.0 (br, s).

##### General Procedure for the Isomerization of Allyl Ethers (A)

Inside the glovebox, an oven-dried J-Young tube was charged with catalyst **3** dissolved in THF (100 μL (1 mol %) to 500 μL (5 mol %), from a 0.03 M stock solution in THF). Next, the solvent was evaporated under reduced pressure, and the substrate (0.3 mmol) was added together with 400 μL of benzene-*d*_6_. The tube was sealed, taken out of the glovebox, and heated at 60 °C until the reaction was complete. The reaction was monitored by ^1^H NMR spectroscopy. After completion of the reaction, the crude reaction mixture was exposed to air, and 1,3,5-trimethylbenzene (4.0 mg, 0.033 mmol, 0.33 M stock solution in benzene-*d*_6_) was added as internal standard. The mixture was filtered through a short plug of neutral alumina plug to remove the cobalt catalyst, and the alumina was washed with an additional 300 μL of benzene-*d*_6_ to collect all the organic products. The yield and *E/Z* ratio of the products were determined by a combination of ^1^H and ^13^C NMR spectroscopy.

##### General Procedure for Single Bond Alkene Isomerization of Allyl Ethers (B)

Inside the glovebox, an oven-dried J-Young tube was charged with substrate (0.15 mmol) and catalyst **4** in toluene-*d*_8_ (50 l to 125 L, 0.003 mmol (2 mol %) to 0.0075 mmol (5 mol %), from a 0.06 M stock in toluene-*d*_8_) was added. To the reaction mixture was added an additional amount of toluene-*d*_8_ to make a total volume of 400 L. The tube was sealed, taken out of the glovebox, and heated at 70 or 80 °C until the reaction was complete. The reaction was monitored by ^1^H NMR spectroscopy. After completion of the reaction, the crude reaction mixture was exposed to air and filtered through a short neutral alumina plug to remove the cobalt catalyst. The alumina was washed with an additional 300 μL of toluene-*d*_8_ to collect all the organic products, and 1,3,5-trimethoxybenzene (8.4 mg, 0.05 mmol, 1 M stock solution in toluene-*d*_8_) was added as an internal standard. The yield and *E/Z* ratio of the products were determined by combination of ^1^H and ^13^C NMR spectroscopy.

## Supporting Information Available

Synthetic procedures, characterization data, catalysis, and computational studies (PDF)
Crystal data (CIF)
xyz-coordinate files (ZIP)

## Supplementary Material

au4c00529_si_001.pdf

au4c00529_si_002.cif
