# Supplementary material for: N2 Dissociation vs Reversible 1,2-Methyl Migration in PCNHCP Cobalt(I) Complexes in the Stereoselective Isomerization (E/Z) of Allyl Ethers
Source: JACS Au. 2024 Sep 18;4(11):4234–48. doi: 10.1021/jacsau.4c00529 (PMC11600169; doi:10.1021/jacsau.4c00529)
Supplement: Supplementary file 1 — au4c00529_si_001.pdf [file au4c00529_si_001.pdf]

Supporting Information

N_2 Dissociation vs. Reversible 1,2-Methyl Migration in $PC_{NHC}P$ Cobalt(I) Complexes in the Stereoselective Isomerization (*E/Z*) of Allyl Ethers.

Sakthi Raje,[ǁ†] Subhash Garhwal,[ǁ†] Katarzyna Młodzikowska-Pieńko,[ǁ†] Tofayel Sheikh Mohammad,[†] Ron Raphaeli,[†] Natalia Fridman,[†] Linda J. W. Shimon,[‡] Renana Gershoni-Poranne,[†*] and Graham de Ruiter[†*]

Email: graham@technion.ac.il
Email: rporanne@technion.ac.il

[†]*Schulich Faculty of Chemistry and the Resnick Sustainability Center for Catalysis, Technion – Israel Institute of Technology, Technion City, 3200008 Haifa, Israel;* [‡]*Department of Chemical Research Support, Weizmann Institute of Science, Rehovot, 7610001, Israel*

[ǁ]These authors contributed equally

Synthesis and characterization data of starting materials 7
Synthesis of [5-(2-propen-1-yloxy)pentyl]benzene: 7
Screening of complexes for single bond allyl ether isomerization 8
Substrate scope for single bond alkene isomerization 9
^{1}H NMR and high-resolution mass (HRMS) spectra of selected compounds 18
Figure S1. High-resolution mass spectrum of 1. 18
Figure S2. ^{1}H NMR spectrum (400 MHz) of 2 in benzene-d_6. 19
Figure S3. ^{13}C {^{1}H} NMR spectrum (101 MHz) of 2 in benzene-d_6. 19
Figure S4. ^{31}P NMR spectrum (162 MHz) of 2 in benzene-d_6. 20
Figure S5. ^{1}H NMR spectrum (400 MHz) of 3 in THF-d_8. 20
Figure S6. ^{13}C {^{1}H} NMR spectrum (101 MHz) of 3 in THF-d_8. 21
Figure S7. ^{31}P NMR spectrum (162 MHz) of 3 in THF-d_8. 21
Figure S8. ^{1}H NMR spectrum (400 MHz) of 4 in benzene-d_6. 22
Figure S9. ^{13}C {^{1}H} NMR spectrum (101 MHz) of 4 in benzene-d_6. 22
Figure S10. ^{31}P NMR spectrum (162 MHz) of 4 in benzene-d_6. 23
Figure S111. ^{1}H NMR spectrum (400 MHz) of [5-(2-propen-1-yloxy)pentyl]benzene in $CDCl_3$. 24
Figure S12. ^{13}C {^{1}H} NMR spectrum (101 MHz) of [5-(2-propen-1-yloxy)pentyl]benzene. 24
Figure S13. ^{1}H NMR spectrum (400 MHz) of 5a in benzene-d_6 (method A). 25
Figure S14. ^{1}H NMR spectrum (400 MHz) of 5a in toluene-d_8 (method B). 25
Figure S15. ^{13}C {^{1}H} NMR spectrum (101 MHz) of 5a in benzene-d_6 (method A). 26
Figure S16. ^{13}C {^{1}H} NMR spectrum (101 MHz) of 5a in toluene-d_8 (method B). 26
Figure S17. ^{1}H NMR spectrum (400 MHz) of 5b in benzene-d_6 (method A). 27
Figure S18. ^{1}H NMR spectrum (400 MHz) of 5b in toluene-d_8 (method B). 27
Figure S19. ^{13}C {^{1}H} NMR spectrum (101 MHz) of 5b in benzene-d_6 (method A). 28
Figure S20. ^{13}C {^{1}H} NMR spectrum (101 MHz) of 5b in toluene-d_8 (method B). 28
Figure S21. ^{1}H NMR spectrum (400 MHz) of 5c in benzene-d_6 (method A). 29
Figure S22. ^{1}H NMR spectrum (400 MHz) of 5c in toluene-d_8 (method B). 29
Figure S23. ^{13}C {^{1}H} NMR spectrum (101 MHz) of 5c in benzene-d_6 (method A). 30
Figure S24. ^{13}C {^{1}H} NMR spectrum (101 MHz) of 5c in toluene-d_8 (method B). 30
Figure S25. ^{1}H NMR spectrum (400 MHz) of 5d in benzene-d_6 (method A). 31
Figure S26. ^{1}H NMR spectrum (400 MHz) of 5d in toluene-d_8 (method B). 31
Figure S27. ^{13}C {^{1}H} NMR spectrum (101 MHz) of 5d in benzene-d_6 (method A). 32
Figure S28. ^{13}C {^{1}H} NMR spectrum (101 MHz) of 5d in toluene-d_8 (method B). 32
Figure S29. ^{1}H NMR spectrum (400 MHz) of 5e in benzene-d_6 (method A). 33
Figure S30. ^{1}H NMR spectrum (400 MHz) of 5e in toluene-d_8 (method B). 33
Figure S31. ^{13}C {^{1}H} NMR spectrum (101 MHz) of 5e in benzene-d_6 (method A). 34
Figure S32. ^{13}C {^{1}H} NMR spectrum (101 MHz) of 5e in toluene-d_8 (method B). 34
Figure S33. ^{1}H NMR spectrum (400 MHz) of 5f in benzene-d_6 (method A). 35
Figure S34. ^{1}H NMR spectrum (400 MHz) of 5f in toluene-d_8 (method B). 35
Figure S35. ^{13}C {^{1}H} NMR spectrum (101 MHz) of 5f in benzene-d_6 (method A). 36
Figure S36. ^{13}C {^{1}H} NMR spectrum (101 MHz) of 5f in toluene-d_8 (method B). 36
Figure S37. ^{1}H NMR spectrum (400 MHz) of 5g in benzene-d_6 (method A). 37
Figure S38. ^{1}H NMR spectrum (400 MHz) of 5g in toluene-d_8 (method B). 37

Figure S39. $^{13}C\ \{^{1}H\}$ NMR spectrum (101 MHz) of 5g in benzene-d_6 (method A)........................38
Figure S40. $^{13}C\ \{^{1}H\}$ NMR spectrum (101 MHz) of 5g in toluene-d_8 (method B). 38
Figure S41. ^{1}H NMR spectrum (400 MHz) of 5h in benzene-d_6 (method A)................................39
Figure S42. ^{1}H NMR spectrum (400 MHz) of 5h in toluene-d_8 (method B). 39
Figure S43. $^{13}C\ \{^{1}H\}$ NMR spectrum (101 MHz) of 5h in benzene-d_6 (method A)........................40
Figure S44. $^{13}C\ \{^{1}H\}$ NMR spectrum (101 MHz) of 5h in toluene-d_8 (method B). 40
Figure S45. ^{1}H NMR spectrum (400 MHz) of 5i in benzene-d_6 (method A).................................41
Figure S46. ^{1}H NMR spectrum (400 MHz) of 5i in toluene-d_8 (method B). 41
Figure S47. ^{1}H NMR spectrum (101 MHz) of 5i in benzene-d_6 (method A).................................42
Figure S48. $^{13}C\ \{^{1}H\}$ NMR spectrum (101 MHz) of 5i in toluene-d_8 (method B). 42
Figure S49. ^{1}H NMR spectrum (400 MHz) of 5j in benzene-d_6 (method A).................................43
Figure S50. ^{1}H NMR spectrum (400 MHz) of 5j in toluene-d_8 (method B). 43
Figure S51. $^{13}C\ \{^{1}H\}$ NMR spectrum (101 MHz) of 5j in benzene-d_6 (method A)........................44
Figure S52. $^{13}C\ \{^{1}H\}$ NMR spectrum (101 MHz) of 5j in toluene-d_8 (method B). 44
Figure S53. ^{1}H NMR spectrum (400 MHz) of 5k in benzene-d_6 (method A)................................45
Figure S54. $^{13}C\ \{^{1}H\}$ NMR spectrum (101 MHz) of 5k in benzene-d_6 (method A)........................45
Figure S55. ^{1}H NMR spectrum (400 MHz) of 5l in benzene-d_6 (method A).................................46
Figure S56. $^{13}C\ \{^{1}H\}$ NMR spectrum (101 MHz) of 5l in benzene-d_6 (method A)........................46
Figure S57. ^{1}H NMR spectrum (400 MHz) of 5l in toluene-d_8 (method B). 47
Figure S58. $^{13}C\ \{^{1}H\}$ NMR spectrum (101 MHz) of 5l in toluene-d_8 (method B)..........................47
Figure S59. ^{1}H NMR spectrum (400 MHz) of 5m in benzene-d_6 (method A)...............................48
Figure S60. ^{1}H NMR spectrum (400 MHz) of 5m in toluene-d_8 (method B). 48
Figure S61. $^{13}C\ \{^{1}H\}$ NMR spectrum (101 MHz) of 5m in benzene-d_6 (method A).......................49
Figure S62. $^{13}C\ \{^{1}H\}$ NMR spectrum (101 MHz) of 5m in toluene-d_8 (method B)........................49
Figure S63. ^{1}H NMR spectrum (400 MHz) of 5n in benzene-d_6 (method A)................................50
Figure S64. $^{13}C\ \{^{1}H\}$ NMR spectrum (101 MHz) of 5n in benzene-d_6 (method A)........................50
Figure S65. ^{1}H NMR spectrum (400 MHz) of 5o in benzene-d_6 (method A)................................51
Figure S66. ^{1}H NMR spectrum (400 MHz) of 5o in toluene-d_8 (method B). 51
Figure S67. $^{13}C\ \{^{1}H\}$ NMR spectrum (101 MHz) of 5o in benzene-d_6 (method A)........................52
Figure S68. $^{13}C\ \{^{1}H\}$ NMR spectrum (101 MHz) of 5o in toluene-d_8 (method B). 52
Figure S69. ^{1}H NMR spectrum (400 MHz) of 5p in benzene-d_6 (method A)................................53
Figure S70. ^{1}H NMR spectrum (400 MHz) of 5p in toluene-d_8 (method B). 53
Figure S71. $^{13}C\ \{^{1}H\}$ NMR spectrum (101 MHz) of 5p in benzene-d_6 (method A)........................54
Figure S72. $^{13}C\ \{^{1}H\}$ NMR spectrum (101 MHz) of 5p in toluene-d_8 (method B). 54
Figure S73. ^{1}H NMR spectrum (400 MHz) of 5q in benzene-d_6 (method A)................................55
Figure S74. ^{1}H NMR spectrum (400 MHz) of 5q in toluene-d_8 (method B). 55
Figure S75. $^{13}C\ \{^{1}H\}$ NMR spectrum (101 MHz) of 5q in benzene-d_6 (method A)........................56
Figure S76. $^{13}C\ \{^{1}H\}$ NMR spectrum (101 MHz) of 5q in toluene-d_8 (method B). 56
Figure S77. ^{1}H NMR spectrum (400 MHz) of 5r in benzene-d_6 (method A). 57
Figure S78. ^{1}H NMR spectrum (400 MHz) of 5r in toluene-d_8 (method B)..................................57
Figure S79. $^{13}C\ \{^{1}H\}$ NMR spectrum (101 MHz) of 5r in benzene-d_6 (method A). 58
Figure S80. $^{13}C\ \{^{1}H\}$ NMR spectrum (101 MHz) of 5r in toluene-d_8 (method B)..........................58

Figure S81. ^{1}H NMR spectrum (400 MHz) of 5s in benzene-d_6 (method A). 59
Figure S82. ^{1}H NMR spectrum (400 MHz) of 5s in toluene-d_8 (method B). 59
Figure S83. ^{13}C {^{1}H} NMR spectrum (101 MHz) of 5s in benzene-d_6 (method A). 60
Figure S84. ^{13}C {^{1}H} NMR spectrum (101 MHz) of 5s in toluene-d_8 (method B). 60
Figure S85. ^{1}H NMR spectrum (400 MHz) of 5t in benzene-d_6 (method A). 61
Figure S86. ^{1}H NMR spectrum (400 MHz) of 5t in toluene-d_8 (method B). 61
Figure S87. ^{13}C {^{1}H} NMR spectrum (101 MHz) of 5t in benzene-d_6 (method A). 62
Figure S88. ^{13}C {^{1}H} NMR spectrum (101 MHz) of 5t in toluene-d_8 (method B). 62
Allyl benzyl ether isomerization as a function of time. 63
Figure S89. Stacked ^{1}H NMR spectrum (400 MHz, C_6D_6) of allyl benzyl ether isomerization with catalyst 3 at different time intervals. 63
Figure S90. Stacked ^{1}H NMR spectrum (400 MHz, C_6D_6) of allyl benzyl ether isomerization with catalyst 4 at different time intervals. 64
Deuterium labelling crossover experiment of dec-1-ene-3,3-d_2 with 4-allyl-1,2-dimethoxybenzene....65
Figure S91. ^{1}H NMR spectrum (400 MHz, C_6D_6) after isomerization of dec-1-ene-3,3-d_2 and 4-allyl-1,2-dimethoxybenzene with catalyst 3. 65
Figure S92. ^{2}H NMR spectrum of isolated product of isomerized 4-allyl-1,2-dimethoxybenzene from the crossover experiment. 66
Figure S93. ^{1}H NMR spectrum (400 MHz, C_6D_6) after isomerization of dec-1-ene-3,3-d_2 and 4-allyl-1,2-dimethoxybenzene with catalyst 4. 67
Figure S94. ^{2}H NMR spectrum of isolated product of isomerized 4-allyl-1,2-dimethoxybenzene from the crossover experiment. 67
Deuterium labelling experiment with dodec-1-ene-1,1-d_2. 68
Figure S95. ^{1}H NMR spectrum (400 MHz, C_6D_6) after isomerization of dodec-1-ene-1,1-d_2 with catalyst 3. 68
Figure S96. ^{13}C {^{1}H} NMR spectrum (101 MHz, C_6D_6) after isomerization of dodec-1-ene-1,1-d_2 with catalyst 3. 69
Figure S97. ^{2}H NMR spectrum (46.07 MHz) after isomerization of dodec-1-ene-1,1-d_2 with catalyst 3 in benzene. 69
Figure S98. ^{1}H NMR spectrum (400 MHz, C_6D_6) after isomerization of dodec-1-ene-1,1-d_2 with catalyst 4. 70
Figure S99. ^{13}C {^{1}H} NMR spectrum (101 MHz, C_6D_6) after isomerization of dodec-1-ene-1,1-d_2 with catalyst 4. 71
Figure S100. ^{2}H NMR spectrum (46.07 MHz) after isomerization of dodec-1-ene-1,1-d_2 with catalyst 4 in benzene. 71
Radical Trapping Experiments. 72
Figure S101. ^{1}H NMR spectrum (400 MHz) after isomerization of allyl benzyl ether in presence of 9,10-dihydroanthracene with catalyst 3 in benzne-d_6. 72
Figure S102. ^{13}C {^{1}H} NMR spectrum (101 MHz) after isomerization of allyl benzyl ether in presence of 9,10-dihydroanthracene with catalyst 3 in benzne-d_6. 73
Figure S103. ^{1}H NMR spectrum (400 MHz) after isomerization of allyl benzyl ether in presence of xanthene with catalyst 3 in benzne-d_6. 73

Figure S104. ^{13}C {^{1}H} NMR spectrum (101 MHz) after isomerization of allyl benzyl ether in presence of xanthene with catalyst 3 in benzne-d_6. 74
Figure S105. ^{1}H NMR spectrum (400 MHz) after isomerization of allyl benzyl ether in presence of 1,1-diphenylethylene with catalyst 3 in benzne-d_6. 74
Figure S106. ^{13}C {^{1}H} NMR spectrum (101 MHz) after isomerization of allyl benzyl ether in presence of 1,1-diphenylethylene with catalyst 3 in benzne-d_6. 75
Figure S107. ^{1}H NMR spectrum (400 MHz) after isomerization of allyl benzyl ether in presence of 9,10-dihydroanthracene with catalyst 4 in toluene-d_8. 76
Figure S108. ^{13}C {^{1}H} NMR spectrum (101 MHz) after isomerization of allyl benzyl ether in presence of 9,10-dihydroanthracene with catalyst 4 in toluene-d_8. 77
Figure S109. ^{1}H NMR spectrum (400 MHz) after isomerization of allyl benzyl ether in presence of xanthene with catalyst 4 in toluene-d_8. 77
Figure S110. ^{13}C {^{1}H} NMR spectrum (101 MHz) after isomerization of allyl benzyl ether in presence of xanthene with catalyst 4 in toluene-d_8. 78
Figure S111. ^{1}H NMR spectrum (400 MHz) after isomerization of allyl benzyl ether in presence of 1,1-diphenylethylene with catalyst 4 in toluene-d_8. 78
Figure S112. ^{13}C {^{1}H} NMR spectrum (101 MHz) after isomerization of allyl benzyl ether in presence of 1,1-diphenylethylene with catalyst 4 in toluene-d_8. 79
Experiments performed to decoordinate N_2 ligand from compound 3. 80
Figure S113. ^{1}H NMR spectra (400 MHz) of N_2 ligand replacement in thf-d_8. 80
Figure S114. ^{31}P NMR spectrum (162 MHz) of N_2 ligand replacement in thf-d_8. 80
Figure S115. ^{1}H NMR spectrum (400 MHz) of [($PC_{NHC}P$)Co(DMAP)](BAr_4^F) in thf-d_8. 81
Figure S116. ^{31}P NMR spectrum (162 MHz) of [($PC_{NHC}P$)Co(DMAP)](BAr_4^F) in thf-d_8. 81
Figure S117. ^{13}C {^{1}H} NMR spectrum (101 MHz) of [($PC_{NHC}P$)Co(DMAP)](BAr_4^F) in thf-d_8. 82
Isomerization of allyl benzyl ether with [($PC_{NHC}P$)Co(DMAP)](BAr_4^F) (5). 83
Figure S118. ^{1}H NMR spectrum (400 MHz) of isomerization of allyl benzyl ether with [($PC_{NHC}P$)Co)(DMAP)](BAr_4^F) in benzene-d_6. 83
Figure S119. ^{1}H NMR spectrum (400 MHz) of [($PC_{NHC}P$)Co(p-tol)] in benzene-d_6. 84
Figure S120. ^{31}P NMR spectrum (162 MHz) of [($PC_{NHC}P$)Co(p-tol)] in benzene-d_6. 84
Figure S121. ^{13}C {^{1}H} NMR spectrum (101 MHz) of [($PC_{NHC}P$)Co(p-tol)] in benzene-d_6. 85
Isomerization of allyl benzyl ether with [($PC_{NHC}P$)Co(p-tol)] (6). 85
Figure S122. ^{1}H NMR spectrum (400 MHz) of isomerization of allyl benzyl ether with [($PC_{NHC}P$)Co)(p-tol)] in benzene-d_6. 85
Experiments performed to observe methyl migration 86
Figure S123. ^{1}H NMR spectrum (400 MHz) of Complex 4 with various monodentate ligand (PPh_3, PMe_3 and IMes carbene) in benzene-d6 after 24h. 86
Figure S124. ^{31}P NMR spectrum (162 MHz) of Complex 4 with various monodentate ligand (PPh_3, PMe_3 and IMes carbene) in benzene-d_6 after 24h. 87
Figure S125. ^{1}H NMR spectrum (400 MHz) of (1:1) mixture of complex 4 and allyl benzyl ether in benzene-d_6 after 9 h at 60 °C. 87
Catalytic cycle for allyl ether isomerization. 88
Figure S126. Plausible mechanism for allyl ether isomerization by catalyst 3. 88
Figure S127. Plausible mechanism for allyl ether isomerization by catalyst 4. 88

Figure S128. Isotope labelling studies for alkene isomerization by catalyst 3 and 4........................89
X-Ray Crystallography **90**
Figure S129. Solid state structure of [(($PC_{NHC}P$)CoCl)] (2)..90
Special Refinement Details for 2...90
Figure S130. Solid state structure of [($PC_{NHC}P$)Co)$_2$(μ-N_2)](BAr_4^F)$_2$ (3). 91
Special Refinement Details for 3...91
Figure S131. Solid state structure of [(($PC_{NHC}P$)CoMe)] (4). 92
Special Refinement Details for 4...92
Tables **93**
Table S2. Selected bond angles and distances for complexes 2–4..93
Table S3. Crystal and refinement data for complexes 2–4..94
Computational Details **95**
Figure S132. Computationally optimized structures for complexes 3 (left) and 4 (right). 95
Benchmarking procedure. 95
Table S5. Calculated and experimentally determined bond distances for complexes 3 and 4..........96
Figure S133. Calculated free energy profiles (ΔG) in kcal/mol at 353.15 K, for the isomerization of allylbenzyl ether with complex 4 without methyl migration. 96
Table S6. Total energy values (E) and Gibbs free energy values for the isomerization of allylbenzyl ether with complex 3 and 4 97
Table S7. Calculated Free energy profiles (ΔG) in kcal/mol for the isomerization of allylbenzyl ether with complex 3 and 4. 99
Geometries of intermediates and transition states involved in the alkene isomerization...............100
Input templates. 100
References **102**

Synthesis and characterization data of starting materials.

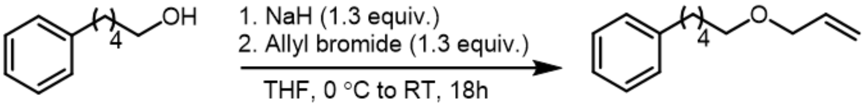

Synthesis of [5-(2-propen-1-yloxy)pentyl]benzene: To a 100 mL oven dried round-bottom flask, containing a magnetic stirring bar, was added 5-phenylpentan-1-ol (1.463 g, 8.9 mmol) in THF (30 mL). After cooling the reaction mixture to 0 °C, NaH (60% dispersion in mineral oil, 462.2 mg, and 11.6 mmol) was added in portion-wise and the resulting mixture was stirred for 30 minutes. To the cloudy mixture, allyl bromide (1.398 g, 11.57 mmol) was added and the reaction mixture was allowed to warm to room temperature. It was stirred for another 18h at room temperature and the reaction was monitored by TLC. Hereafter, the reaction mixture was quenched with water (20 mL) and the compound was extracted with Et_2O (3 x 30 mL). The combined organic layer was washed with brine (30 mL), whereafter dried over Na_2SO_4 and evaporated under reduced pressure to obtain an oily residue. The crude compound was purified by column chromatography (hexane/Et_2O 3:2) to afford a colorless oil. Yield: 1.72 g (84%). ^{1}H NMR (400 MHz, $CDCl_3$) δ 7.43 – 7.30 (m, 2H), 7.30 – 7.15 (m, 3H), 5.99 (ddd, J = 22.8, 10.8, 5.6 Hz, 1H), 5.29 (ddd, J = 13.8, 11.6, 1.4 Hz, 2H), 4.03 (d, J = 5.6 Hz, 2H), 3.50 (t, J = 6.7 Hz, 2H), 2.71 – 2.66 (m, 2H), 1.71 (dd, J = 15.1, 7.4 Hz, 4H), 1.52 – 1.44 (m, 2H). ^{13}C NMR (101 MHz, $CDCl_3$) δ 142.8, 135.2, 128.4 (d, J = 15.1 Hz), 125.7, 116.8, 71.9 , 70.5, 36.0, 31.5, 29.8, 26.0.

Screening of complexes for single bond allyl ether isomerization

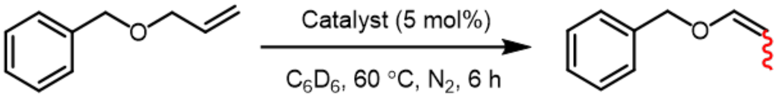

Inside the glovebox, an oven-dried J-Young tube was charged with substrate (0.2 mmol) and catalyst (5 mol%) and 400 µL solvent was added. The reaction mixture was stirred at 60 °C for 6 h. The progress of the reaction was monitored by ^{1}H NMR spectroscopy. The reaction was optimized by changing to reaction conditions according to **Table 1**.

Table S1. Reaction optimization and catalyst screening for allyl ether isomerization.[a]

Entry	**Cat. (mol%)**	*Solvent*	*Temp. (°C)*	*Time (h)*	*Product Conversion (Z:E)*[b]
**1**	**3** (5)	THF	60	6	98 (3:2)
**2**	**3** (5)	THF	60	12	99 (3:2)
**3**	**3** (5)	THF	60	24	99 (1:1)
**4**	**3** (1)	Et_2O	60	9	98 (3:1)
**5**	**3** (1)	Et_2O	60	22	98 (3:2)
**6**	**3** (5)	toluene-d_8	60	2	97 (2:1)
**7**	**3** (5)	toluene-d_8	60	6.5	98 (1:1)
**8**	**3** (5)	benzene-d_6	60	2	98 (2:1)
**9**	**3** (5)	benzene-d_6	60	6.5	99 (3:2)
**10**	**3** (5)	benzene-d_6	90	1	99 (1:1)
**11**	**3** (1)	benzene-d_6	60	4.5	99 (3:1)
**12**	**3** (1)	benzene-d_6	90	2	99 (3:2)
**13**	**3** (1)	benzene-d_6	30	24	50 (3:1)
**14**	**3** (0.5)	benzene-d_6	60	18	75 (3:1)
**15**	**4** (5)	toluene-d_8	60	20	98 (1:2)
**16**	**4** (5)	toluene-d_8	70	14	98 (1:2)
**17**	**4** (5)	toluene-d_8	80	6	98 (2:3)
**18**	**4** (5)	benzene-d_6	60	20	98 (1:2)

[a]Unless otherwise noted, the reaction was performed with ((allyloxy)methyl)benzene (0.2 mmol), catalyst (x mol%) in400 µL solvent at the specified temperature and time. [b]The product conversion and *E*/*Z* ratio were determined by ^{1}H NMR analysis.

Substrate scope for single bond alkene isomerization

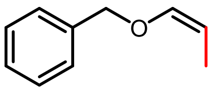

Compound 5a. **Prepared according to general procedure (A)**. (5h, Colorless oil, 99% yield, *E/Z* 1:3.4 (**1 mol%, cat.3**)) ***Z*-5a:** ^{1}H NMR (400 MHz, benzene-d_6): δ (ppm) 7.23 – 7.06 (m, 5H), 5.88 (dq, *J* = 6.2, 1.7 Hz, 1H), 4.44 – 4.38 (m, 3H), 1.74 (dd, *J* = 6.8, 1.7 Hz, 3H). ^{13}C {^{1}H} NMR (101 MHz, benzene-d_6): δ 145.8, 138.4, 128.6, 127.9, 127.5, 101.5, 73.5, 9.7. NMR spectra are consistent with previous reported data.[1]

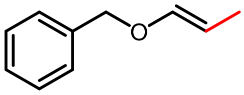

Compound 5a. **Prepared according to general procedure (B)**. (5h, Colorless oil, 96% yield, *E/Z* 1.8:1.0 (**5 mol%, cat.4**)) ***E*-5a**: ^{1}H NMR (400 MHz, toluene-d_8): δ (ppm) 7.20 – 7.01 (m, 5H), 6.22 (dd, *J* = 12.5, 1.5 Hz, 1H), 4.75 (dq, *J* = 13.3, 6.7 Hz, 1H), 4.42 (s, 2H), 1.45 (dd, *J* = 6.7, 1.5 Hz, 3H). ^{13}C {^{1}H} NMR (101 MHz, toluene-d_8): δ (ppm) 147.4, 138.5, 128.9, 128.1, 127.9, 99.3, 71.3, 13.1. NMR spectra are consistent with previous reported data.[1]

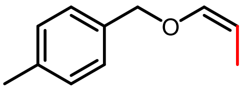

Compound 5b. Prepared according to general procedure (A). (10h, Colorless oil, 98% yield, *E/Z* 1:3.0 (**2 mol%, cat.3**)) ***Z*-5b:** ^{1}H NMR (400 MHz, benzene-d_6): δ (ppm) 7.18 – 7.11 (m, 2H), 6.99 - 6.95 (m, 2H), 5.93 (ddd, *J* = 6.2, 3.3, 1.6 Hz, 1H), 4.47 (s, 2H), 4.45 – 4.39 (m, 1H), 2.08 (s, 3H), 1.75 (dd, *J* = 6.8, 1.7 Hz, 3H). ^{13}C {^{1}H} NMR (101 MHz, benzene-d_6): δ (ppm) 145.8, 137.4, 135.4, 129.3, 127.8, 101.4, 73.5, 21.1, 9.7.

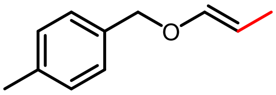

Compound 5b. Prepared according to general procedure (B). (10h, Colorless oil, 91% yield, *E/Z* 1.7:1.0 (**5 mol%, cat.4**)) ***E*-5b**:^{1}H NMR (400 MHz, toluene-d_8): δ (ppm) 7.12 (d, *J* = 8.1 Hz, 2H), 6.95 (d, *J* = 7.7 Hz, 2H), 6.25 (dd, *J* = 12.5, 1.4 Hz, 1H), 4.78 (dq, *J* = 13.2, 6.7 Hz, 1H), 4.45 (s, 2H), 2.11 (s, 3H), 1.47 (dd, *J* = 6.7, 1.5 Hz, 3H). ^{13}C {^{1}H} NMR (101 MHz, toluene-d_8): δ (ppm) 147.5, 137.6, 135.5, 129.6, 128.1, 99.1, 71.3, 21.4, 13.1.

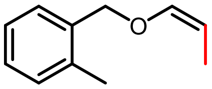

Compound 5c. Prepared according to general procedure (A). (8h, Colorless oil, 94% yield, *E/Z* 1:2.8 (**1 mol%, cat.3**)) ***Z*-5c:** ^{1}H NMR (400 MHz, benzene-d_6): δ (ppm) 7.31 – 7.24 (m, 1H), 7.07 – 6.95 (m, 3H), 5.91 (ddd, *J* = 6.0, 3.2, 1.6 Hz, 1H), 4.46– 4.45 (m, 2H), 4.45 – 4.38 (m, 1H), 2.08 (s, 3H), 1.72 (dd, *J* = 6.8, 1.6 Hz, 3H). ^{13}C {^{1}H} NMR (101 MHz, benzene-d_6): δ (ppm) 145.7, 136.6, 136.2, 130.5, 128.5, 128.2, 126.1, 101.4, 72.2, 18.7, 9.6.

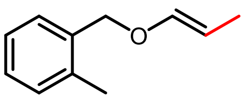

Compound 5c. Prepared according to general procedure (B). (10h, Colorless oil, 94% yield, *E/Z* 1.3:1.0 (**5 mol%, cat.4**)) ***E*-6c:** ^{1}H NMR (400 MHz, toluene-d_8): δ (ppm) 7.27 – 7.20 (m, 1H), 7.07 – 7.02 (m, 2H), 6.98 – 6.92 (m, 1H), 6.23 (dd, *J* = 12.5, 1.4 Hz, 1H), 4.79 (dq, *J* = 13.3, 6.7 Hz, 1H), 4.42 (s, 2H), 2.10 (s, 3H), 1.47 (dd, *J* = 6.7, 1.5 Hz, 3H). ^{13}C {^{1}H} NMR (101 MHz, toluene-d_8): δ (ppm) 147.4, 136.9, 136.3, 130.8, 128.8, 128.5, 126.4, 99.1, 69.9, 19.0, 13.1.

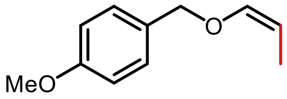

Compound 5d. Prepared according to general procedure (A). (9h, Colorless oil, 95% yield, *E/Z* 1:3.9 (**1 mol%, cat.3**)) ***Z*-5d:** ^{1}H NMR (400 MHz, benzene-d_6): δ (ppm) 7.16 – 7.10 (m, 2H), 6.77 – 6.72 (m, 2H), 5.95 (dq, *J* = 6.2, 1.7 Hz, 1H), 4.47 – 4.43 (m, 2H), 4.47 – 4.40 (m, 1H), 3.28 (s, 3H), 1.75 (dd, *J* = 6.8, 1.7 Hz, 3H). ^{13}C {^{1}H} NMR (101 MHz, benzene-d_6): δ (ppm) 159.9, 145.8, 130.3, 129.3, 114.1, 101.3, 73.4, 54.7, 9.7. NMR spectra are consistent with previous reported data.[2]

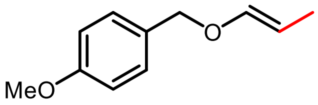

Compound 5d. Prepared according to general procedure (B). (3h, Colorless oil, 96% yield, *E/Z* 1.4:1.0 (**5 mol%, cat.4**)) ***E*-5d:** ^{1}H NMR (400 MHz, toluene-d_8): δ (ppm) 7.12 – 7.07 (m, 2H), 6.72 – 6.67 (m, 2H), 6.25 (dd, *J* = 12.5, 1.5 Hz, 1H), 4.79 (dq, *J* = 13.2, 6.7 Hz, 1H), 4.42 (s, 2H), 3.30 (s, 3H), 1.48 (dd, *J* = 6.7, 1.5 Hz, 3H). ^{13}C {^{1}H} NMR (101 MHz, toluene-d_8): δ (ppm) 160.2, 147.5, 130.4, 129.5, 114.4, 99.1, 71.2, 55.0, 13.2. NMR spectra are consistent with previous reported data.[3]

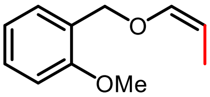

Compound 5e. Prepared according to general procedure (A). (12h, Colorless oil, 98% yield, *E/Z* 1:3.3 (**1 mol%, cat.3**)) ***Z*-5e:** ^{1}H NMR (400 MHz, benzene-d_6): δ (ppm) 7.54 - 7.47 (m, 1H), 7.11 – 7.05 (m, 1H), 6.95 – 6.86 (m, 1H), 6.53 – 6.46 (m, 1H), 6.04 (ddd, *J* = 6.1, 3.2, 1.5 Hz, 1H), 4.86 (s, 2H), 4.42 (p, *J* = 6.7 Hz, 1H), 3.23 (s, 3H), 1.77 (dd, *J* = 6.8, 1.6 Hz, 3H). ^{13}C {^{1}H} NMR (101 MHz, benzene-d_6): δ (ppm) 157.2, 146.4, 128.9, 128.8, 127.4, 120.8, 110.3, 101.0, 69.0, 54.8, 9.7.

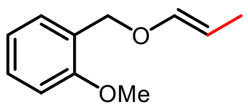

Compound 5e. Prepared according to general procedure (B). (10h, Colorless oil, 93% yield, *E/Z* 1.3:1.0 (**5 mol%, cat.4**)) ***E*-5e:** ^{1}H NMR (400 MHz, toluene-d_8): δ (ppm) 7.47 (d, *J* = 7.4 Hz, 1H), 7.11 – 7.02 (m, 1H), 6.88 – 6.83 (m, 1H), 6.48 – 6.44 (m, 1H), 6.29 (dd, *J* = 12.5, 1.4 Hz, 1H), 4.89 – 4.81 (m, 1H), 4.78 (s, 2H), 3.30 (s, 3H), 1.45 (dd, *J* = 6.7, 1.5 Hz, 3H). ^{13}C {^{1}H} NMR (101 MHz, toluene-d_8): δ (ppm) 157.0, 147.3, 128.6, 128.5, 127.0, 126.6, 120.7, 98.6, 66.2, 54.6, 12.8.

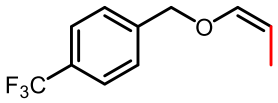

Compound 5f. Prepared according to general procedure (A). (12h, Colorless oil, 99% yield, *E/Z* 1:3.2 (**1 mol%, cat.3**)) ***Z*-5f:** ^{1}H NMR (400 MHz, benzene-d_6): δ (ppm) 7.32 – 7.28 (m, 2H), 7.00 – 6.93 (m, 2H), 5.74 (ddd, *J* = 6.1, 3.3, 1.6 Hz, 1H), 4.42 (p, *J* = 6.7 Hz, 1H), 4.25 (s, 2H), 1.71 (dd, *J* = 6.8, 1.7 Hz, 3H). ^{13}C {^{1}H} NMR (101 MHz, benzene-d_6): δ (ppm) 145.5, 142.3, 127.4, 125.5, 125.5, 102.2, 72.4, 9.6.

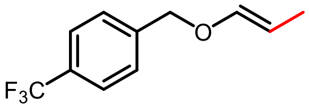

Compound 5f. Prepared according to general procedure (B). (10h, Colorless oil, 84% yield, *E/Z* 1.0:1.0 (**5 mol%, cat.4**)) ***E*-5f:** ^{1}H NMR (400 MHz, toluene-d_8): δ (ppm) 7.30 – 7.26 (m, 2H), 6.99 – 6.92 (m, 2H), 6.18 – 6.12 (m, 1H), 4.70 (dq, *J* = 13.3, 6.7 Hz, 1H), 4.27 (s, 2H), 1.45 (dd, *J* = 6.7, 1.4 Hz, 3H). ^{13}C {^{1}H} NMR (101 MHz, toluene-d_8): δ (ppm) 147.0, 142.7, 127.8, 127.6, 125.8, 125.8, 99.8, 70.1, 13.0.

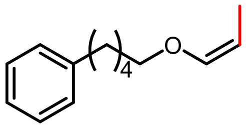

Compound 5g. Prepared according to general procedure (A). (7h, Colorless oil, 99% yield, *E/Z* 1:3.0 (**1 mol%, cat.3**)) ***Z*-5g:** ^{1}H NMR (400 MHz, benzene-d_6): δ (ppm) 7.20 – 7.16 (m, 2H), 7.10 – 7.04 (m, 3H), 5.84 (ddd, *J* = 6.1, 3.3, 1.6 Hz, 1H), 4.41 (p, *J* = 6.7, 1H), 3.4 (t, *J* = 6.5 Hz, 2H), 2.45 – 2.40 (m, 2H), 1.75 (dd, *J* = 6.8, 1.7 Hz, 3H), 1.48 – 1.38 (m, 4H), 1.30 – 1.18 (m, 2H). ^{13}C {^{1}H} NMR (101 MHz, benzene-d_6): δ (ppm) 146.2, 142.8, 128.7, 128.6, 126.0, 100.5, 71.9, 36.2, 31.5, 30.0, 25.8, 9.7.

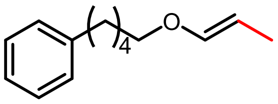

Compound 5g. Prepared according to general procedure (B). (5h, Colorless oil, 94% yield, *E/Z* 1.9:1.0 (**5 mol%, cat.4**)) ***E*-5g:** ^{1}H NMR (400 MHz, toluene-d_8): δ (ppm) 7.16 – 7.12 (m, 2H), 7.07 – 6.95 (m, 3H), 6.20 (dd, *J* = 12.6, 1.4 Hz, 1H), 4.68 (dq, *J* = 13.0, 6.6 Hz, 1H), 3.41 – 3.36 (m, 2H), 2.44 – 2.39 (m, 2H), 1.52 – 1.38 (m, 7H), 1.31 – 1.18 (m, 2H). ^{13}C {^{1}H} NMR (101 MHz, toluene-d_8): δ (ppm) 147.8, 143.1, 129.8, 128.9, 126.4, 97.9, 69.1, 36.6, 32.0, 30.0, 26.5, 13.2.

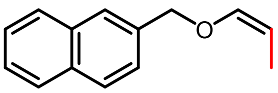

Compound 5h. **Prepared according to general procedure (A).** (9h, Colorless oil, 92% yield, *E/Z* 1:3.5 (**1 mol%, cat.3**)) ***Z*-5h:** ^{1}H NMR (400 MHz, benzene-d_6): δ (ppm) 7.63 – 7.57 (m, 4H), 7.35 – 7.22 (m, 3H), 5.95 (ddd, *J* = 6.1, 3.2, 1.6 Hz, 1H), 4.58 (s, 2H), 4.45 (p, *J* = 6.7 Hz, 1H), 1.79 (dd, *J* = 6.8, 1.7 Hz, 3H). ^{13}C {^{1}H} NMR (101 MHz, benzene-d_6): δ (ppm) 145.8, 135.8, 135.6, 133.8, 133.5, 128.5, 126.5, 126.4, 126.2, 125.6, 101.7, 73.6, 71.1, 9.8.

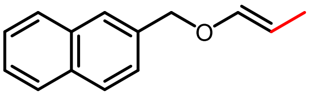

Compound 5h. **Prepared according to general procedure (B)**. (10h, Colorless oil, 90% yield, *E/Z* 1.2:1.0 (**5 mol%, cat.4**)) ***E*-5h:** ^{1}H NMR (400 MHz, toluene-d_8): δ (ppm) 7.60 – 7.52 (m, 4H), 7.31 – 7.27 (m, 1H), 7.26 – 7.21 (m, 2H), 6.29 (dd, *J* = 12.5, 1.2 Hz, 1H), 4.83 (dq, *J* = 13.3, 6.7 Hz, 1H), 4.58 (s, 2H), 1.48 (dd, *J* = 6.7, 1.3 Hz, 3H). ^{13}C {^{1}H} NMR (101 MHz, toluene-d_8): δ (ppm) 147.0, 135.8, 135.5, 133.8, 133.5, 128.3, 128.0, 126.4, 126.2, 126.0, 125.6, 99.0, 71.0, 12.7.

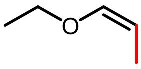

Compound 5i. **Prepared according to general procedure (A)**. (7h, Colorless oil, 99% yield, *E/Z* 1:1.8 (7 h, **1 mol%, cat.3**)) ***Z*-5i:** ^{1}H NMR (400 MHz, benzene-d_6): δ (ppm) 5.81 (ddd, *J* = 6.1, 3.2, 1.6 Hz, 1H), 4.40 (p, *J* = 6.7 Hz, 1H), 3.41 (q, *J* = 7.0 Hz, 2H), 1.74 (dd, *J* = 6.8, 1.7 Hz, 3H), 0.98 (t, *J* = 7.0 Hz, 3H). ^{13}C {^{1}H} NMR (101 MHz, benzene-d_6): δ (ppm) 145.9, 100.6, 67.3, 15.4, 9.6. . NMR spectra are consistent with previous reported data.[4]

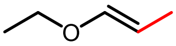

Compound 5i. **Prepared according to general procedure (B)**. (5h, Colorless oil, 97% yield, *E/Z* 2.1:1.0 (**3 mol%, cat.4**)) ***E*-5i:** ^{1}H NMR (400 MHz, toluene-d_8): δ (ppm) 6.17 (dd, *J* = 12.7, 1.4 Hz, 1H), 4.66 (dq, *J* = 13.2, 6.7 Hz, 1H), 3.43 – 3.36 (m, 2H), 1.48 (dd, *J* = 6.7, 1.4 Hz, 3H), 1.05 (t, *J* = 7.0 Hz, 3H). ^{13}C {^{1}H} NMR (101 MHz, toluene-d_8): δ (ppm) 147.6, 98.0, 64.5, 15.3, 13.2. NMR spectra are consistent with previous reported data.[4]

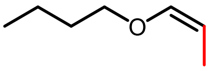

Compound 5j. **Prepared according to general procedure (A)**. (7h, Colorless oil, 98% yield, *E/Z* 1:1.6 (**1 mol%, cat.3**)) ***Z*-5j:** ^{1}H NMR (400 MHz, benzene-d_6): δ (ppm) 5.84 (ddd, *J* = 6.1, 3.2, 1.6 Hz, 1H), 4.40 (p, *J* = 6.7 Hz, 1H), 3.43 (t, *J* = 6.5 Hz, 2H), 1.74 (dd, *J* = 6.8, 1.6 Hz, 3H), 1.48 – 1.22 (m, 4H), 0.79 (q, *J* = 7.4 Hz, 3H). ^{13}C {^{1}H} NMR (101 MHz, benzene-d_6): δ (ppm) 146.3, 100.5, 71.7, 32.2, 19.4, 13.9, 9.6. NMR spectra are consistent with previous reported data.[4]

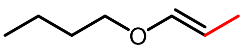

Compound 5j. **Prepared according to general procedure (B).** (5h, Colorless oil, 95% yield, *E/Z* 2.2:1.0 (**3 mol%, cat.4**)) ***E*-5j:** ^{1}H NMR (400 MHz, toluene-d_8): δ (ppm) 6.20 (d, *J* = 11.5 Hz, 1H), 4.68 (dq, *J* = 13.2, 6.7 Hz, 1H), 3.43 – 3.39 (m, 2H), 1.49 (d, *J* = 7.9 Hz, 3H), 1.47 – 1.42 (m, 2H), 1.35 – 1.21 (m, 2H), 0.84 – 0.79 (m, 3H). ^{13}C {^{1}H} NMR (101 MHz, toluene-d_8): δ (ppm) 147.9, 97.8, 68.9, 32.2, 20.0, 14.3, 13.2 NMR spectra are consistent with previous reported data.[4]

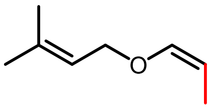

Compound 5k. **Prepared according to general procedure (A)**. (9h, Colorless oil, 97% yield, *E/Z* 1:3.8 (**1 mol%, cat.3**)) ***Z*-5k:** ^{1}H NMR (400 MHz, benzene-d_6): δ (ppm) 5.95 (ddd, *J* = 6.2, 3.3, 1.6 Hz, 1H), 5.37 (tdd, *J* = 5.5, 2.8, 1.4 Hz, 1H), 4.44 (p, *J* = 6.7 Hz, 1H), 4.08 (m, 2H), 1.77 (dd, *J* = 6.8, 1.7 Hz, 3H), 1.50 (m, 3H), 1.38 (s, 3H). ^{13}C {^{1}H} NMR (101 MHz, benzene-d_6): δ (ppm) 145.8, 136.5, 121.6, 100.7, 68.4, 25.6, 17.8, 9.7. NMR spectra are consistent with previous reported data.[1]

Compound 5l. **Prepared according to general procedure (A)**. (16h, Colorless oil, 94% yield, *E/Z* 1:4.5 (**5 mol%, cat.3**)) ***Z*-5l:** ^{1}H NMR (400 MHz, benzene-d_6): δ (ppm) 7.22 – 7.14 (m, 2H), 7.13 – 7.01 (m, 3H), 6.42 (d, *J* = 16.0, 1H), 6.22 – 6.02 (m, 1H), 5.92 (ddd, *J* = 6.2, 3.3, 1.7 Hz, 1H), 4.45 (p, *J* = 6.8 Hz, 1H), 4.12 – 4.02 (m, 2H), 1.78 (dd, *J* = 6.8, 1.7 Hz, 3H). ^{13}C {^{1}H} NMR (101 MHz, benzene-d_6): δ (ppm) 145.7, 137.0, 132.6, 128.8, 128.0, 126.9, 125.9, 101.2, 72.2, 9.7. NMR spectra are consistent with previous reported data.[5]

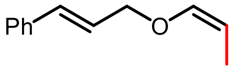

Compound 5l. **Prepared according to general procedure (B)**. (24h, Colorless oil, 58% yield, *E/Z* 1:2.3 (**5 mol%, cat.4**)) ***Z*-5l:** ^{1}H NMR (400 MHz, toluene-d_8): δ (ppm) 7.19 – 7.09 (m, 5H), 6.40 (d, *J* = 16.0, 1H), 6.20 – 5.99 (m, 1H), 5.90 – 5.88 (m, 1H), 4.42 (p, *J* = 6.8 Hz, 1H), 4.07 – 4.03 (m, 2H), 1.76 (dd, *J* = 6.8, 1.7 Hz, 3H). ^{13}C {^{1}H} NMR (101 MHz, toluene-d_6): δ (ppm) 146.0, 137.4, 132.9, 129.1, 128.3, 127.2, 126.2, 101.4, 72.6, 10.0. NMR spectra are consistent with previous reported data.[5]

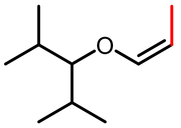

Compound 5m. **Prepared according to general procedure (A)**. (10h, Colorless oil, 99% yield, *E/Z* 1:2.4 (**1 mol%, cat.3**)) ***Z*-5m:** ^{1}H NMR (400 MHz, benzene-d_6): δ (ppm) 5.88 (m, 1H), 4.24 (p, *J* = 6.7 Hz, 1H), 2.74 (t, *J* = 5.8 Hz, 1H), 1.75 (m, 5H), 0.96 - 0.93 (m, 6H), 0.83 – 0.79 (m, 6H). ^{13}C {^{1}H} NMR (101 MHz, benzene-d_6): δ (ppm) 149.4, 97.5, 93.2, 30.8, 20.0, 17.6, 9.6. NMR spectra are consistent with previous reported data.[6]

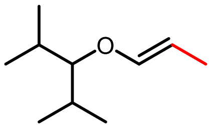

Compound 5m. **Prepared according to general procedure (B).** (5h, Colorless oil, 94% yield, *E/Z* 2.5:1.0 (**5 mol%, cat.4**)) ***E*-5m:** ^{1}H NMR (400 MHz, toluene-d_8): δ (ppm) 5.99 (dd, *J* = 12.1 1.4 Hz, 1H), 4.91 (dq, *J* = 13.5, 6.8 Hz, 1H), 2.78 (t, *J* = 5.8 Hz, 1H), 1.78 – 1.69 (m, 2H), 1.49 (dd, *J* = 6.8 1.5 Hz, 3H), 0.93 – 0.90 (m, 6H), 0.83 – 0.79 (m, 6H). ^{13}C {^{1}H} NMR (101 MHz, toluene-d_8): δ (ppm) 150.9, 98.0, 92.4, 31.3, 20.4, 18.1, 12.9.

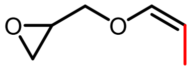

Compound 5n. **Prepared according to general procedure (A)**. (16h, Colorless oil, 88% yield, *E/Z* 1:3.3 (**4 mol%, cat.3**)) ***Z*-5n:** ^{1}H NMR (400 MHz, benzene-d_6): δ (ppm) 5.86 (dq, *J* = 6.1, 1.7 Hz, 1H), 4.42 – 4.35 (m, 1H), 3.48 – 3.43 (m, 1H), 3.25 – 3.17 (m, 1H), 2.71 (ddt, *J* = 5.7, 4.1, 2.8 Hz, 1H), 2.19 (dd, *J* = 5.1, 4.1 Hz, 1H), 2.06 (dd, *J* = 5.2, 2.6 Hz, 1H), 1.69 (dd, *J* = 6.8, 1.7 Hz, 3H). ^{13}C {^{1}H} NMR (101 MHz, benzene-d_6): δ (ppm) 146.1, 101.4, 72.4, 50.4, 43.4, 9.5. NMR spectra are consistent with previous reported data.[7]

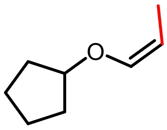

Compound 5o. **Prepared according to general procedure (A).** (9h, Colorless oil, 98% yield, *E/Z* 1:3.0 (**1 mol%, cat.3**)) ***Z*-5o:** ^{1}H NMR (400 MHz, benzene-d_6): δ (ppm) 5.87 (ddd, *J* = 6.2, 3.3, 1.6 Hz, 1H), 4.44 (p, *J* = 6.7 Hz, 1H), 3.91 (m, 1H), 1.76 – 1.70 (m, 3H), 1.77 – 1.61 (m, 4H), 1.48 – 1.31 (m, 4H). ^{13}C {^{1}H} NMR (101 MHz, benzene-d_6): δ (ppm) 144.9, 101.2, 83.1, 33.0, 23.8, 9.7. NMR spectra are consistent with previous reported data.[6]

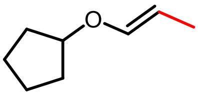

Compound 5o. **Prepared according to general procedure (B).** (5h, Colorless oil, 98% yield, *E/Z* 1.7:1.0 (**5 mol%, cat.4**)) ***E*-5o:** ^{1}H NMR (400 MHz, toluene-d_8): δ (ppm) 6.04 (dd, *J* = 12.5, 1.3 Hz, 1H), 4.76 (dq, *J* = 13.3, 6.7 Hz, 1H), 4.05 – 4.01 (m, 1H), 1.79 – 1.59 (m, 4H), 1.54 – 1.27 (m, 7H). ^{13}C {^{1}H} NMR (101 MHz, toluene-d_8): δ (ppm) 146.4, 99.7, 81.1, 33.2, 24.5, 13.3.

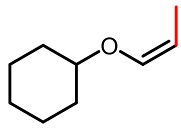

Compound 5p. **Prepared according to general procedure (A).** (9h, Colorless oil, 99% yield, *E/Z* 1:3.3 (**1 mol%, cat.3**)) ***Z*-5p:** ^{1}H NMR (400 MHz, benzene-d_6): δ (ppm) 5.91 (ddd, *J* = 6.1, 3.2 1.6 Hz, 1H), 4.44 (p, *J* = 6.7 Hz, 1H), 3.32 (m, 1H), 1.76 (dd, *J* = 6.8, 1.7 Hz, 3H), 1.71 – 1.55 (m, 4H), 1.43 – 1.26 (m, 4H), 1.14 – 1.05 (m, 2H). ^{13}C {^{1}H} NMR (101 MHz, benzene-d_6): δ (ppm) 144.8, 100.7, 78.9, 32.7, 25.9, 23.8, 9.8. NMR spectra are consistent with previous reported data.[6]

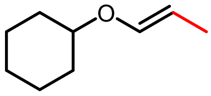

Compound 5p. **Prepared according to general procedure (B)**. (5h, Colorless oil, 95% yield, *E/Z* 1.5:1.0 (**5 mol%, cat.4**)) ***E*-5p:** ^{1}H NMR (400 MHz, toluene-d_8): δ (ppm) 6.00 (dd, *J* = 12.3, 1.5 Hz, 1H), 4.92 (dq, *J* = 13.5, 6.7 Hz, 1H), 3.39 (ddd, *J* = 12.4, 8.5, 3.6 Hz, 1H), 1.70 – 1.57 (m, 4H), 1.51 (dd, *J* = 6.7, 1.2 Hz, 3H), 1.42 – 1.27 (m, 4H), 1.15 – 1.06 (m, 2H). ^{13}C {^{1}H} NMR (101 MHz, toluene-d_8): δ (ppm) 146.3, 100.2, 77.8, 32.8, 26.4, 24.2, 13.2. NMR spectra are consistent with previous reported data.[8]

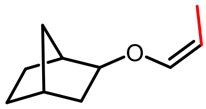

Compound 5q. **Prepared according to general procedure (A).** (10h, Colorless oil, 96% yield, *E/Z* 1:2.0 (**2 mol%, cat.3**)) ***Z*-5q:** ^{1}H NMR (400 MHz, benzene-d_6): δ (ppm) 5.94 (ddd, *J* = 6.1, 3.2, 1.6 Hz, 1H), 4.43 (p, *J* = 6.7 Hz, 1H), 3.20 (td, *J* = 10.6, 4.3 Hz, 1H), 2.37 – 2.28 (m, 1H), 1.92 (m, 1H), 1.77 (dd, *J* = 6.8, 1.7 Hz, 3H), 1.34 (ddd, *J* = 10.3, 7.2, 3.5 Hz 2H), 1.01 - 0.85 (m, 2H), 0.83 – 0.80 (m, 4H). ^{13}C {^{1}H} NMR (101 MHz, benzene-d_6): δ (ppm) 145.5, 100.2, 81.5, 48.2, 42.1, 34.7, 31.7, 26.3, 24.0, 22.3, 20.9, 16.8, 9.8. NMR spectra are consistent with previous reported data.[6]

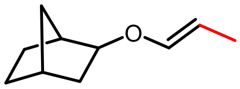

Compound 5q. **Prepared according to general procedure (B).** (5h, Colorless oil, 90% yield, *E/Z* 1.4:1.0 (**5 mol%, cat.4**)) ***E*-5q:** ^{1}H NMR (400 MHz, toluene-d_8): δ (ppm) 6.03 (dd, *J* = 12.3, 1.4 Hz, 1H), 4.95 (dq, *J* = 13.4, 6.7 Hz, 1H), 3.25 (td, *J* = 10.6, 4.2 Hz, 1H), 2.33 – 2.22 (m, 1H), 2.0 – 1.96 (m, 1H), 1.54 – 1.44 (m, 5H), 0.97 – 0.77 (m, 6H). ^{13}C {^{1}H} NMR (101 MHz, toluene-d_8): δ (ppm) 146.9, 99.9, 80.3, 48.6, 41.8, 35.2, 32.0, 26.7, 24.3, 22.9, 21.3, 17.1, 13.1.

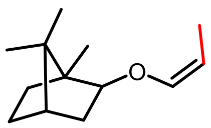

Compound 5r. **Prepared according to general procedure (A).** (10h, Colorless oil, 99% yield, *E/Z* 1:2.1 (**2 mol%, cat.3**)) ***Z*-5r:** ^{1}H NMR (400 MHz, benzene-d_6): δ (ppm) 5.84 (ddd, *J* = 6.1, 3.2, 1.6 Hz, 1H), 4.37 (p, *J* = 6.7 Hz, 1H), 3.33 (dd, *J* = 7.9, 3.3 Hz, 1H), 1.89 – 1.81 (m, 1H), 1.71 (dd, *J* = 6.7, 1.7 Hz, 3H), 1.61 – 1.52 (m, 2H), 1.47 – 1.30 (m, 2H), 1.15 (s, 3H), 0.95 (s, 3H), 0.93 – 0.80 (m, 2H), 0.76 (s, 3H).^{13}C{^{1}H} NMR (101 MHz, benzene-d_6): δ (ppm) 146.1, 99.9, 89.0, 49.7, 46.8, 45.4, 39.1, 34.2, 27.5, 20.4, 20.4, 20.3, 12.1, 9.8. NMR spectra are consistent with previous reported data.[6]

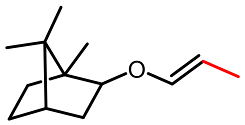

Compound 5r. **Prepared according to general procedure (B).** (10h, Colorless oil, 91% yield, *E/Z* 1.2:1.0 (**5 mol%, cat.4**)) ***E*-5r:** ^{1}H NMR (400 MHz, toluene-d_8): δ (ppm). 6.01 (dd, *J* = 12.5, 1.1 Hz, 1H), 4.73 (td, *J* = 13.3, 6.7 Hz, 1H), 3.44 (dd, *J* = 7.6, 3.3 Hz, 1H), 1.87 – 1.77 (m, 1H), 1.63 – 1.28 (m, 7H), 1.08 (s, 3H), 0.99 – 0.80 (m, 5H), 0.76 (s, 3H). ^{13}C {^{1}H} NMR (101 MHz, toluene-d_8): δ (ppm) 146.7, 99.0, 86.9, 49.7, 47.3, 45.9, 39.6, 34.8, 28.0, 20.8, 20.6, 13.3, 12.4.

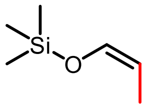

Compound 5s. **Prepared according to general procedure (A).** (8h, Colorless oil, 98% yield, *E/Z* 1:5.5 (**2 mol%, cat.3**)) ***Z*-5s:** ^{1}H NMR (400 MHz, benzene-d_6): δ (ppm) 6.17 (ddd, *J* = 5.4, 3.4, 1.7 Hz, 1H), 4.60 – 4.54 (m, 1H), 1.71 (dd, *J* = 6.7, 1.7 Hz, 3H), 0.07 (s, 9H).^{13}C {^{1}H} NMR (101 MHz, benzene-d_6): δ (ppm) 139.0, 105.4, 9.4, 2.1, -0.5. NMR spectra are consistent with previous reported data.[9]

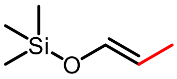

Compound 5s. **Prepared according to general procedure (B).** (12h, Colorless oil, 93% yield, *E/Z* 2.1:1.0 (**5 mol%, cat.4**)) ***E*-5s:** ^{1}H NMR (400 MHz, toluene-d_8): δ (ppm) 6.21 (dd, *J* = 11.9, 1.6 Hz, 1H), 5.07 (dq, *J* = 13.5, 6.8 Hz, 1H), 1.46 (dd, *J* = 6.8, 1.6 Hz, 3H), 0.11 – 0.08 (m, 9H). ^{13}C {^{1}H} NMR (101 MHz, toluene-d_8): δ (ppm) 140.9, 106.3, 12.8, -0.1. NMR spectra are consistent with previous reported data.[9]

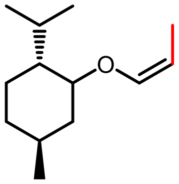

Compound 5t. **Prepared according to general procedure (A).** (7h, Colorless oil, 99% yield, *E/Z* 1:2.9 (**1 mol%, cat.3**)) ***Z*-5t:** ^{1}H NMR (400 MHz, benzene-d_6): δ (ppm) 5.88 (ddd, *J* = 6.2, 3.3, 1.6 Hz, 1H), 4.44 (p, *J* = 6.7 Hz, 1H), 3.42 (d, *J* = 6.9 Hz 1H), 2.23 (d, *J* = 3.6 Hz, 1H), 2.05 (m, 2H), 1.72 (dd, *J* = 6.8, 1.7 Hz, 3H), 1.66 (dd, *J* = 9.6, 1.6 Hz, 1H), 1.54 – 1.49 (m, 1H) 1.41 – 1.17 (m, 8H), 0.99 – 0.70 (m, 5H). ^{13}C {^{1}H} NMR (101 MHz, benzene-d_6): δ (ppm) 144.9, 144.8, 101.2, 101.1, 84.0, 41.8, 41.6, 39.5, 35.7, 35.6, 35.0, 28.8, 22.4, 9.9, 9.7. NMR spectra are consistent with previous reported data.[6]

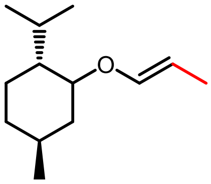

Compound 5t. **Prepared according to general procedure (B).** (5h, Colorless oil, 96% yield, *E/Z* 1.5:1.0 (**5 mol%, cat.4**)) ***E*-5t:** ^{1}H NMR (400 MHz, toluene-d_8) δ (ppm) 6.06 (dd, *J* = 12.6, 1.3 Hz, 1H), 4.73 (dq, *J* = 13.2, 6.7 Hz, 1H), 3.54 – 3.50 (m, 1H), 2.30 – 2.27 (m, 1H), 2.07 – 2.04 (m, 1H), 1.64 – 1.60 (m, 1H), 1.51 – 1.44 (m, 6H), 1.41 – 1.21 (m, 6H), 1.01 – 0.71 (m, 4H). ^{13}C {^{1}H} NMR (101 MHz, toluene-d_8): δ (ppm) 146.1, 99.4, 81.9, 41.7, 40.2, 36.1, 35.6, 29.2, 24.9, 13.3. NMR spectra are consistent with previous reported data.[10]

^{1}H NMR and high-resolution mass (HRMS) spectra of selected compounds

Elemental Composition Report **Page 1**

Single Mass Analysis
Tolerance = 40.0 mDa / DBE: min = -1.5, max = 200.0
Element prediction: Off
Number of isotope peaks used for i-FIT = 3

Monoisotopic Mass, Odd and Even Electron Ions
22 formula(e) evaluated with 7 results within limits (up to 50 closest results for each mass)
Elements Used:
C: 30-35 H: 45-55 N: 1-3 P: 1-3 Cl: 1-2 Co: 1-2
SMT 2050 2
Dr_3688 18 (0.150) Cm (15:37) 1: TOF MS ES+ 1.37e+006

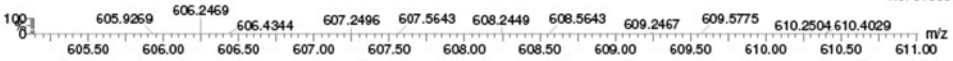

Mass	Calc. Mass	mDa	PPM	DBE	i-FIT	Norm	Conf (%)	Formula
Minimum:				-1.5				
Maximum:		40.0	10.0	200.0				
606.2469	606.2470	-0.1	-0.2	8.5	851.5	1.710	18.08	C31 H50 N2 P2 Cl Co
	606.2467	0.2	0.3	13.0	856.3	6.535	0.15	C35 H47 N P Cl Co
	606.2344	12.5	20.6	9.0	850.0	0.237	78.93	C30 H48 N3 P2 Cl Co
	606.2596	-12.7	-20.9	8.0	853.4	3.621	2.67	C32 H52 N P2 Cl Co
	606.2341	12.8	21.1	13.5	856.1	6.385	0.17	C34 H45 N2 P Cl Co
	606.2233	23.6	38.9	9.0	863.7	13.987	0.00	C32 H48 N P Cl2 Co
	606.2108	36.1	59.5	9.5	865.0	15.234	0.00	C31 H46 N2 P Cl2 Co

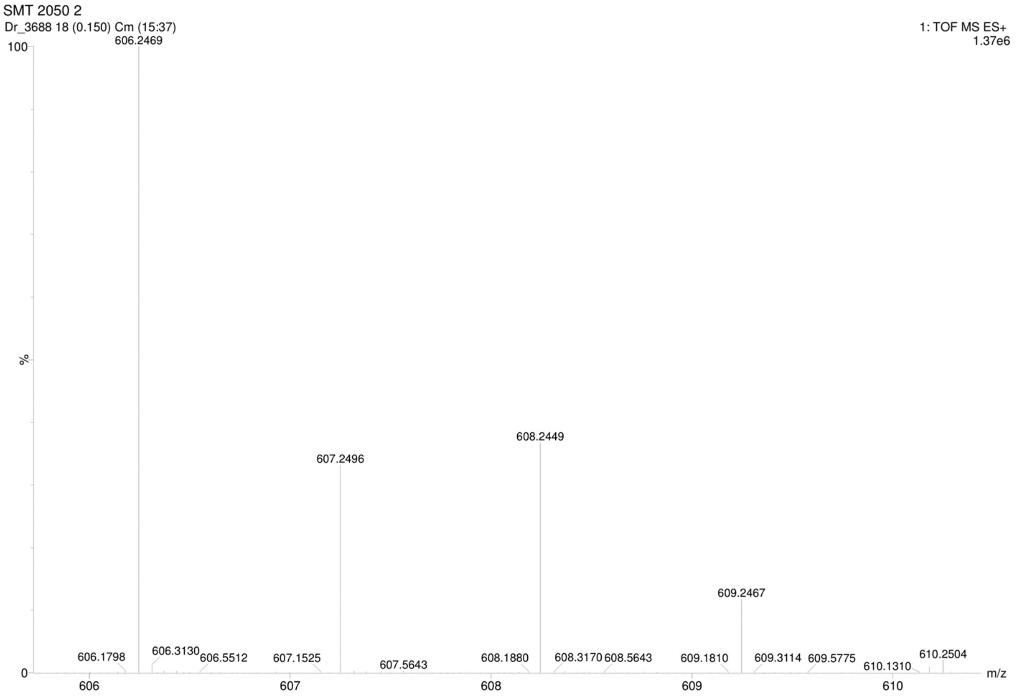

Figure S1. High-resolution mass spectrum of **1**.

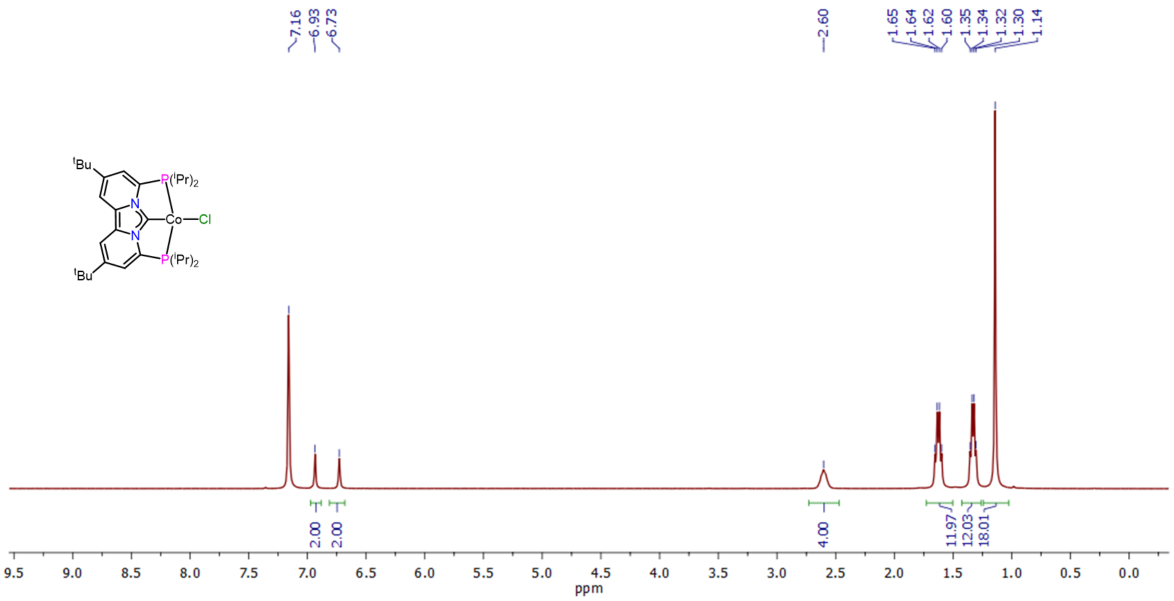

Figure S2. ^{1}H NMR spectrum (400 MHz) of **2** in benzene-d_6.

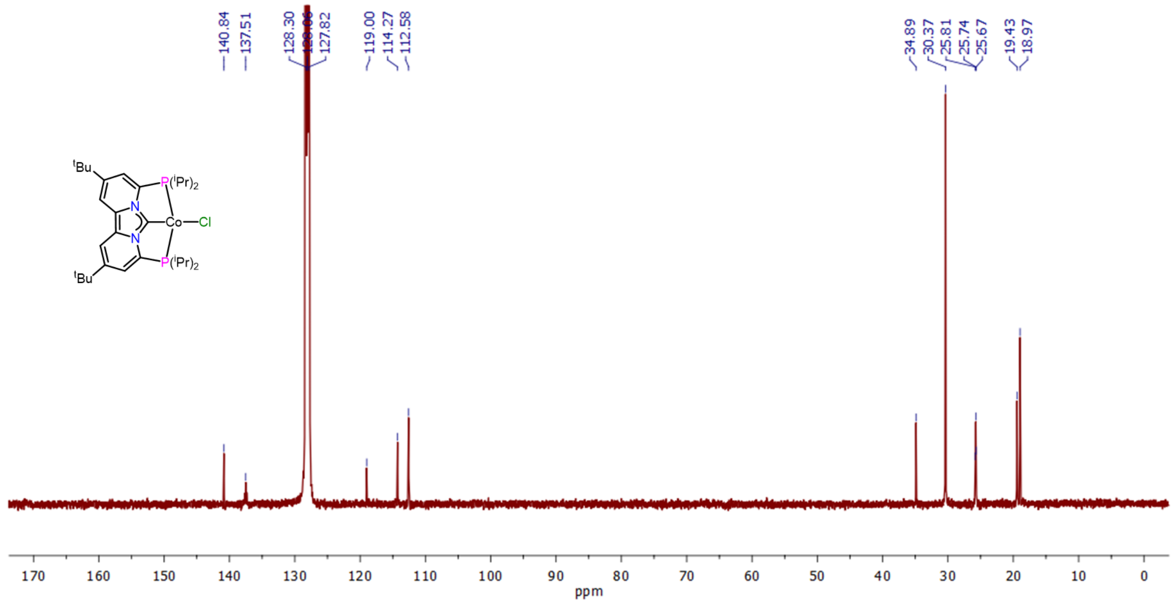

Figure S3. ^{13}C {^{1}H} NMR spectrum (101 MHz) of **2** in benzene-d_6.

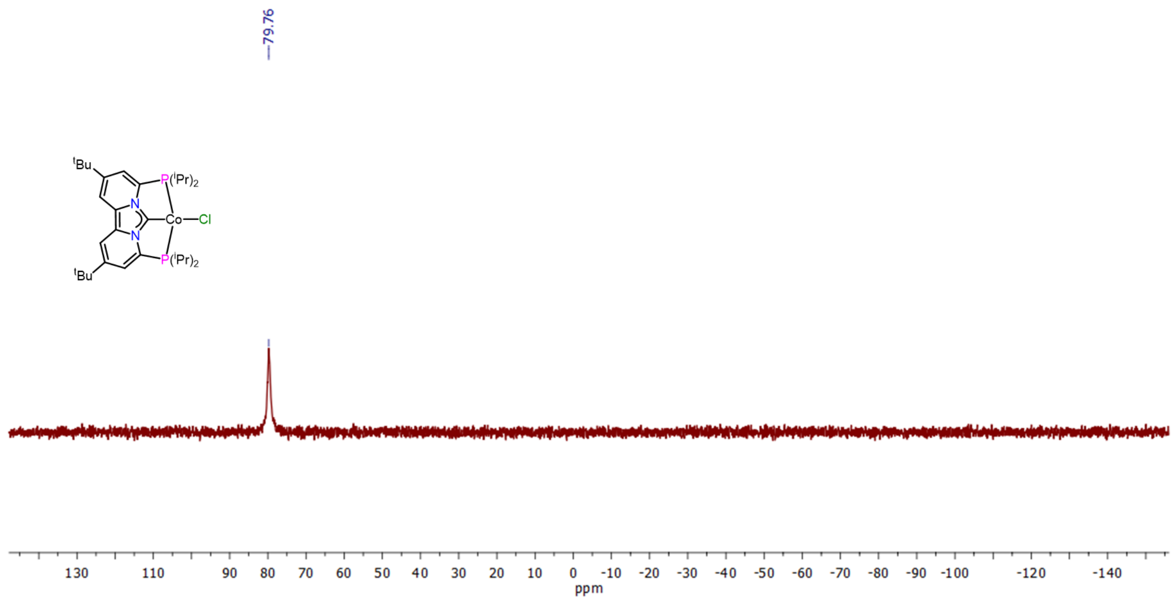

Figure S4. ^{31}P NMR spectrum (162 MHz) of **2** in benzene-d_6.

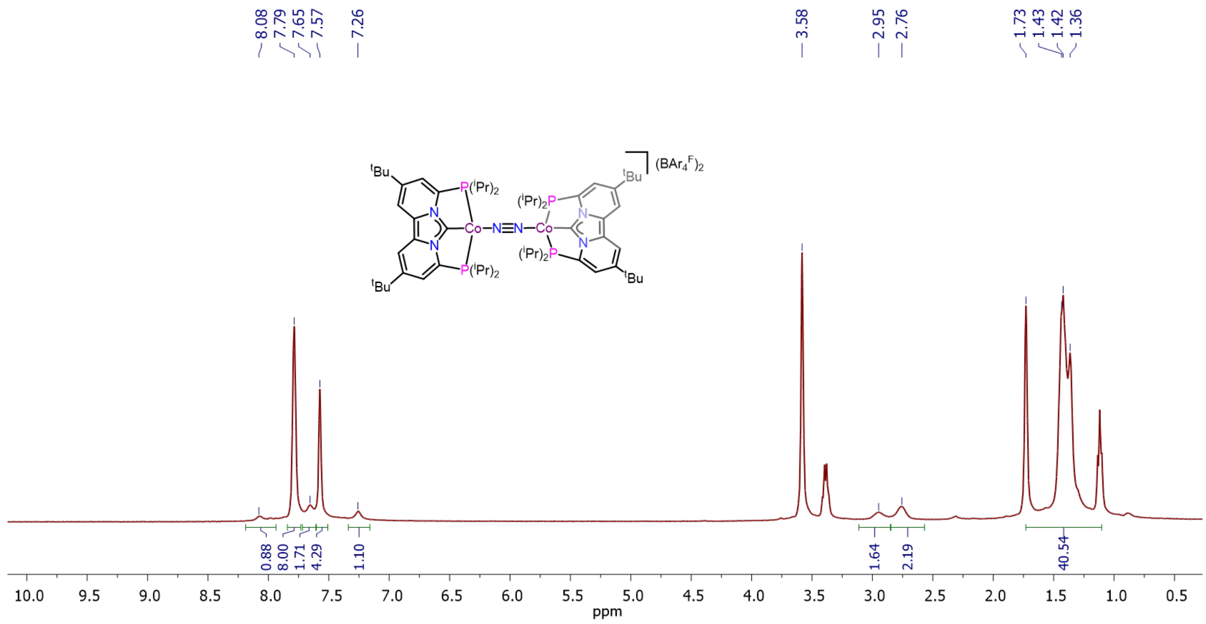

Figure S5. ^{1}H NMR spectrum (400 MHz) of **3** in THF-d_8.

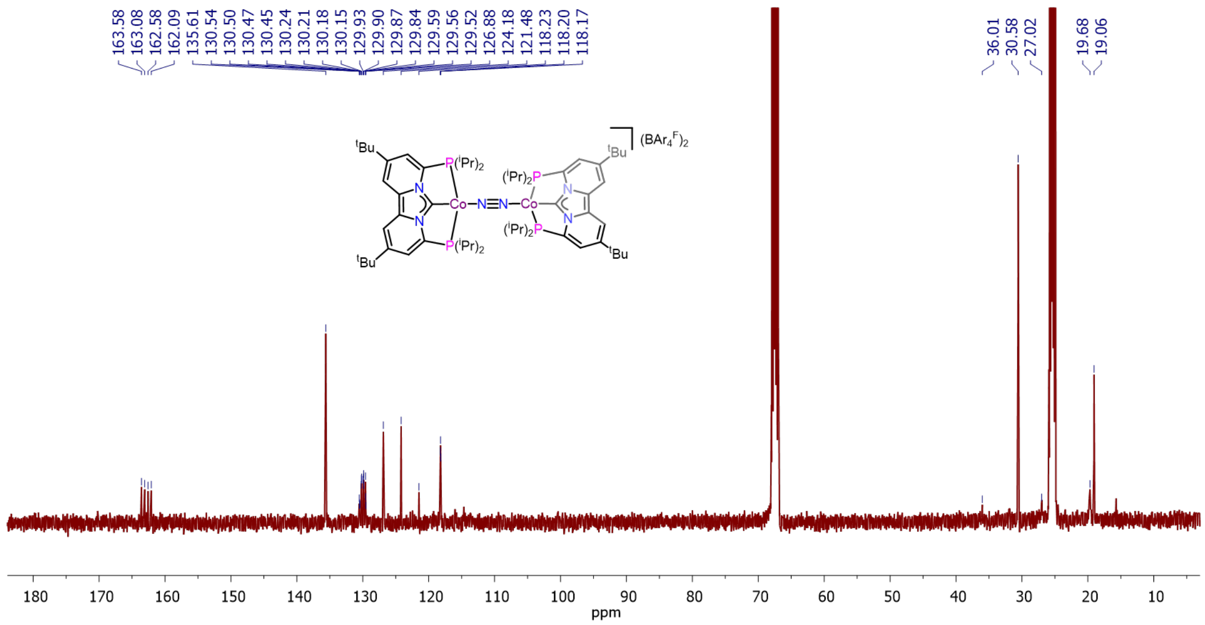

Figure S6. ^{13}C {^{1}H} NMR spectrum (101 MHz) of **3** in THF-d_8.

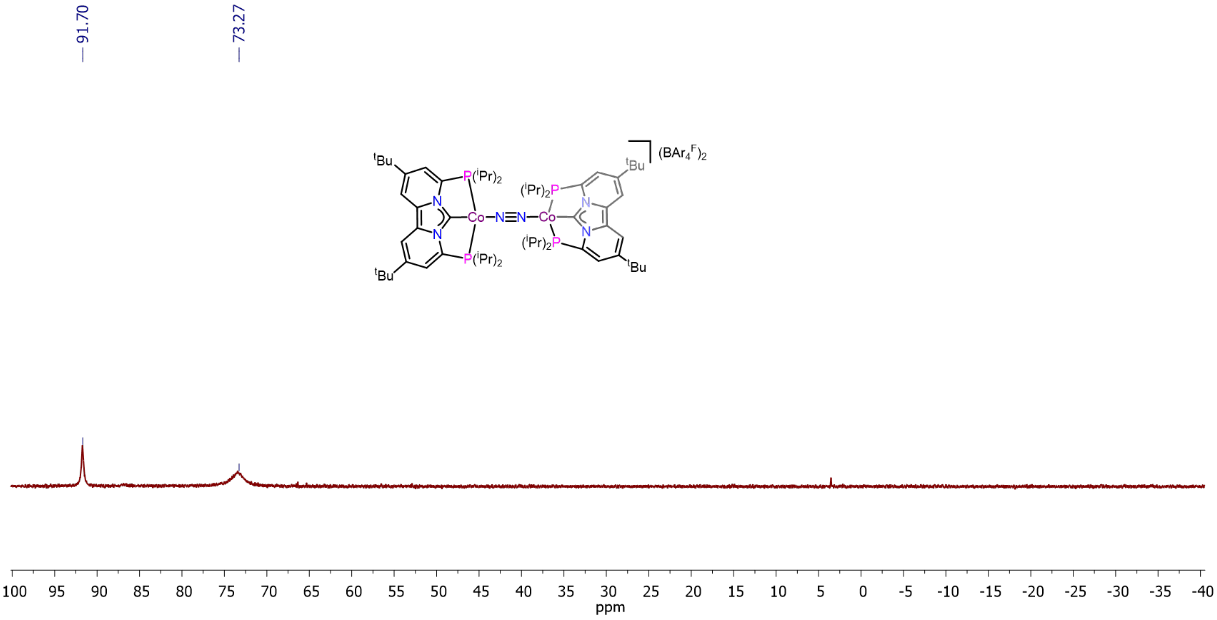

Figure S7. ^{31}P NMR spectrum (162 MHz) of **3** in THF-d_8.

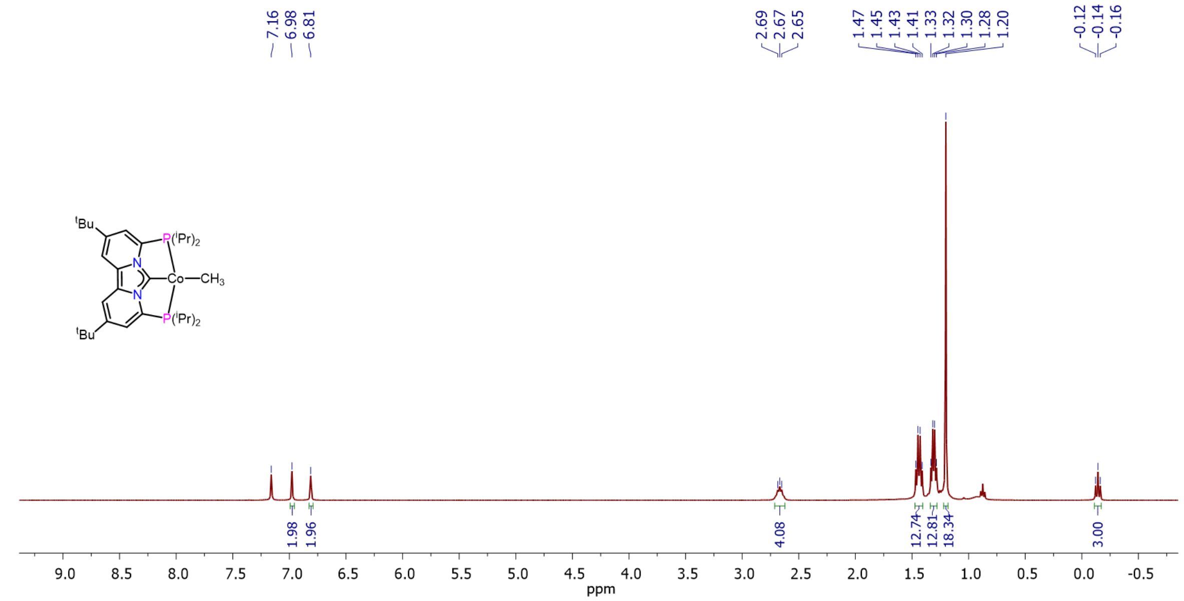

Figure S8. ^{1}H NMR spectrum (400 MHz) of **4** in benzene-d_6.

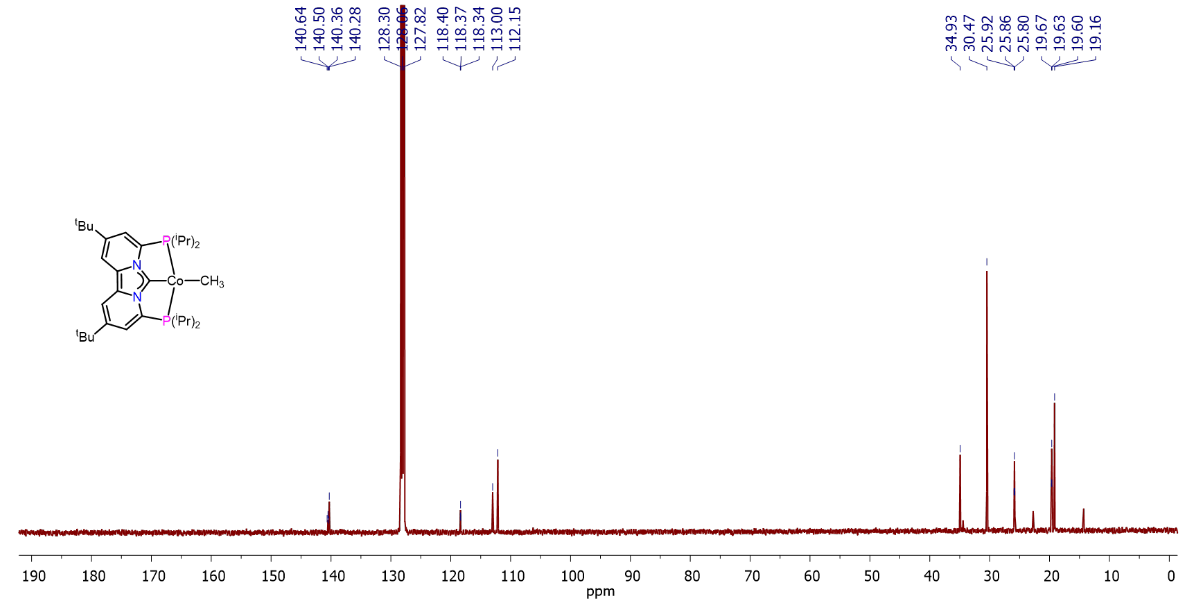

Figure S9. ^{13}C {^{1}H} NMR spectrum (101 MHz) of **4** in benzene-d_6.

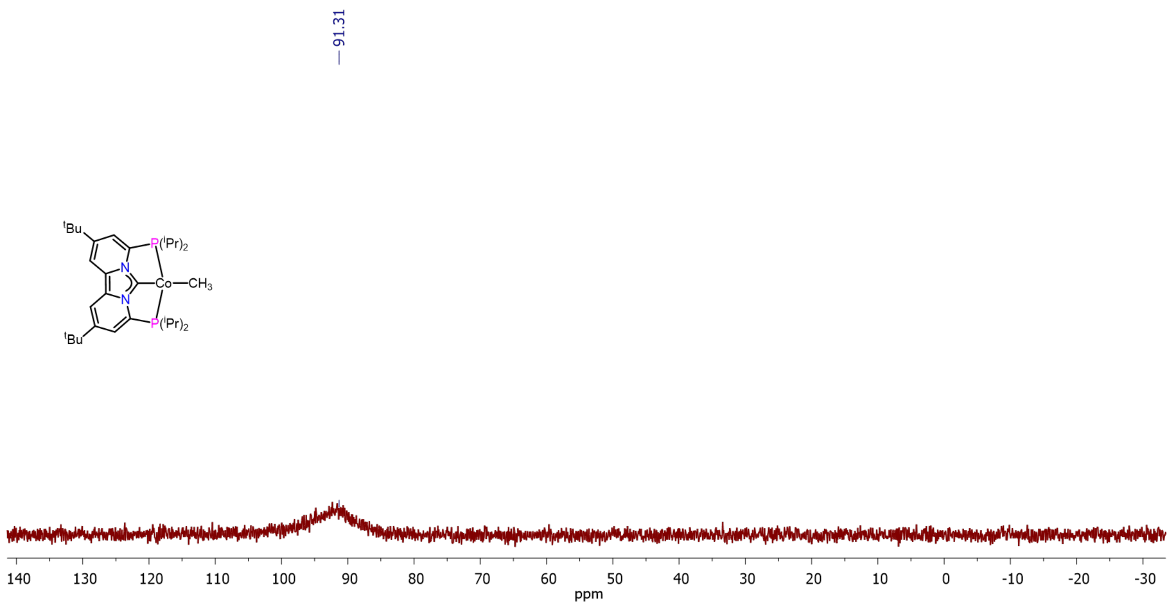

Figure S10. ^{31}P NMR spectrum (162 MHz) of **4** in benzene-d_6.

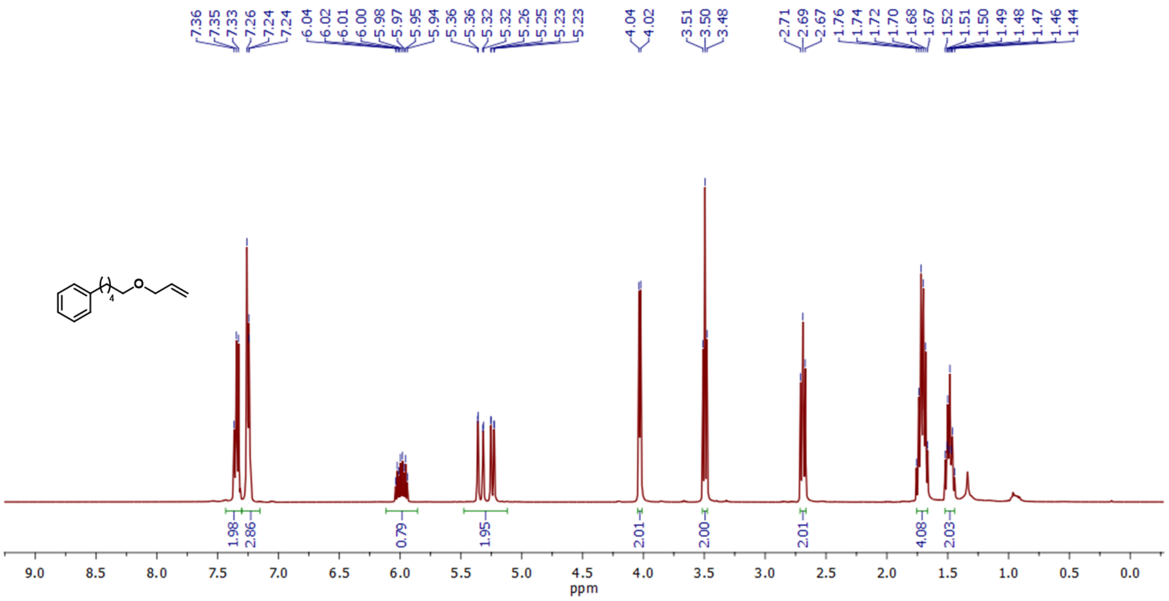

Figure S111. ^{1}H NMR spectrum (400 MHz) of **[5-(2-propen-1-yloxy)pentyl]benzene** in $CDCl_3$.

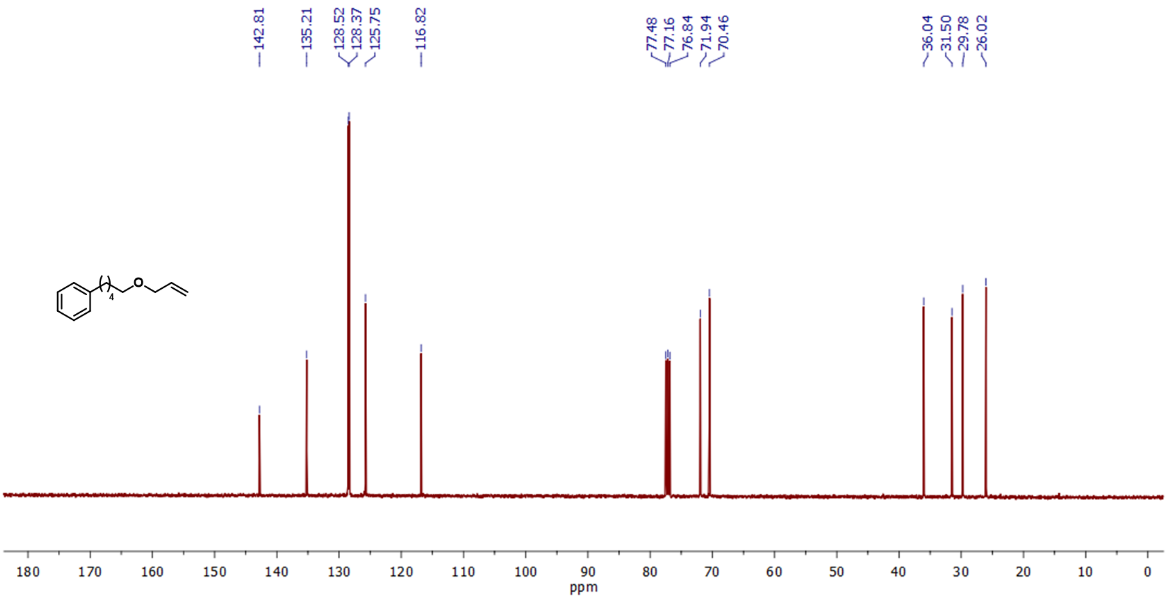

Figure S12. ^{13}C {^{1}H} NMR spectrum (101 MHz) of **[5-(2-propen-1-yloxy)pentyl]benzene** in $CDCl_3$.

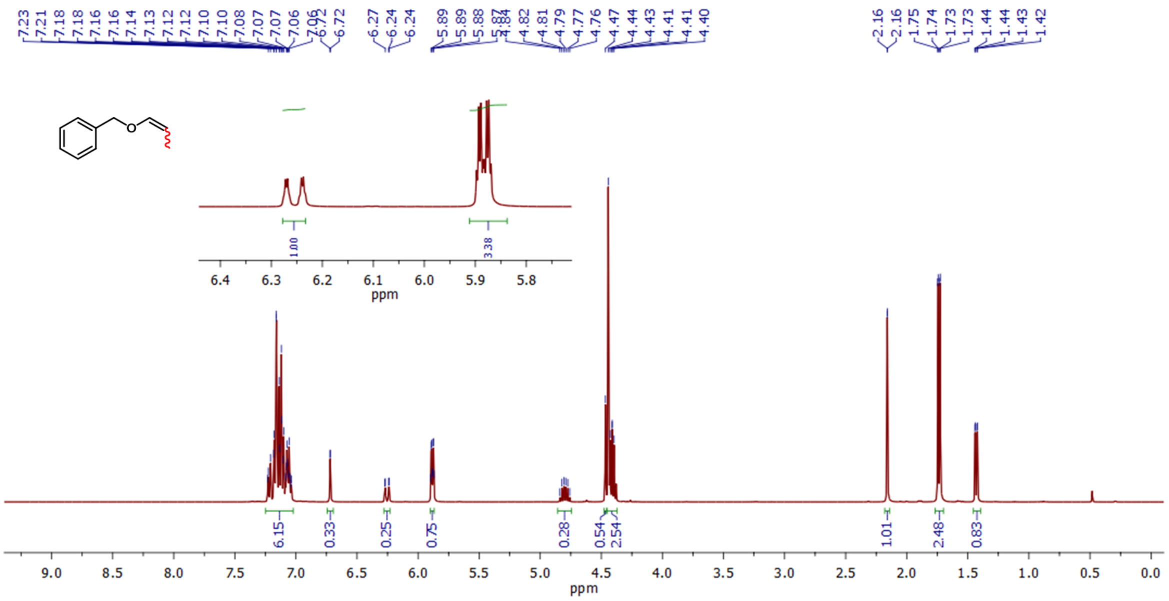

Figure S13. ^{1}H NMR spectrum (400 MHz) of **5a** in benzene-d_6 (method A).

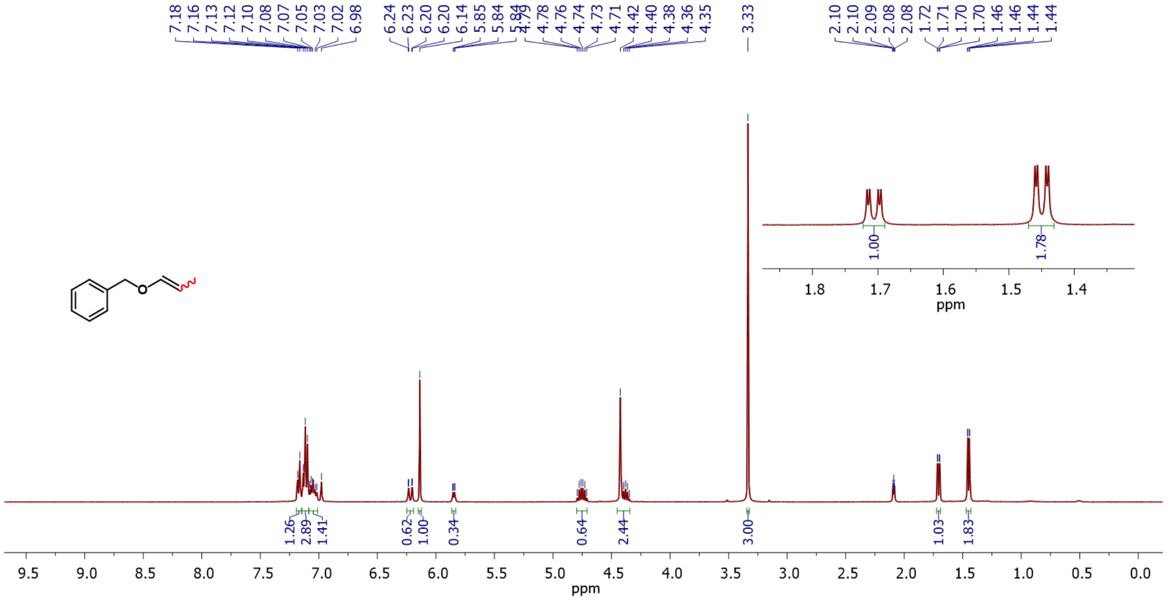

Figure S14. ^{1}H NMR spectrum (400 MHz) of **5a** in toluene-d_8 (method B).

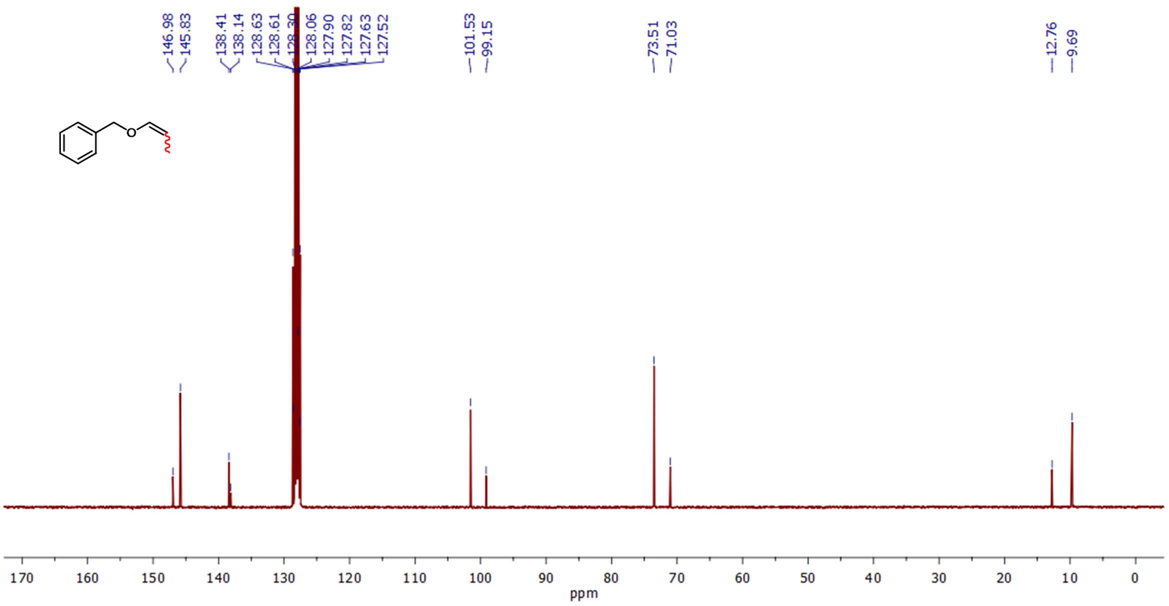

Figure S15. ^{13}C {^{1}H} NMR spectrum (101 MHz) of **5a** in benzene-d_6 (method A).

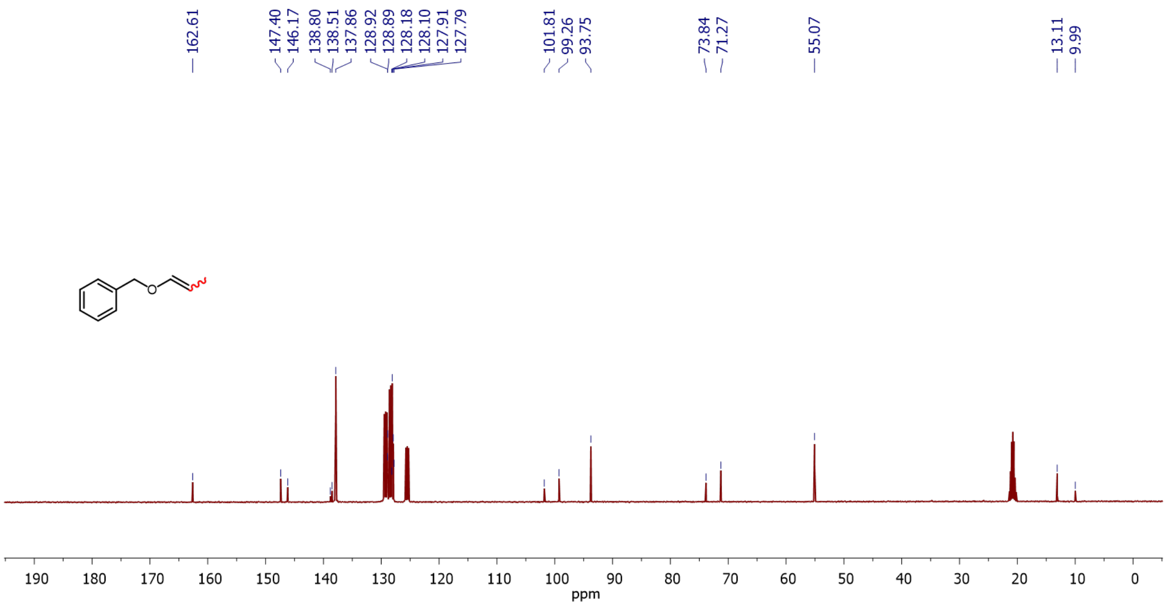

Figure S16. ^{13}C {^{1}H} NMR spectrum (101 MHz) of **5a** in toluene-d_8 (method B).

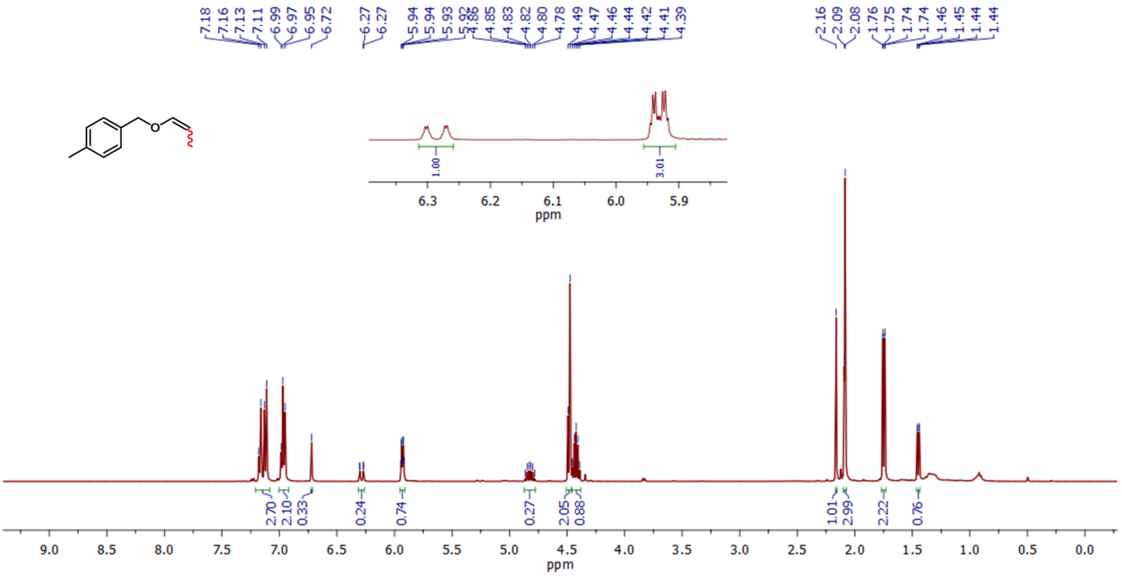

Figure S17. ^{1}H NMR spectrum (400 MHz) of **5b** in benzene-d_6 (method A).

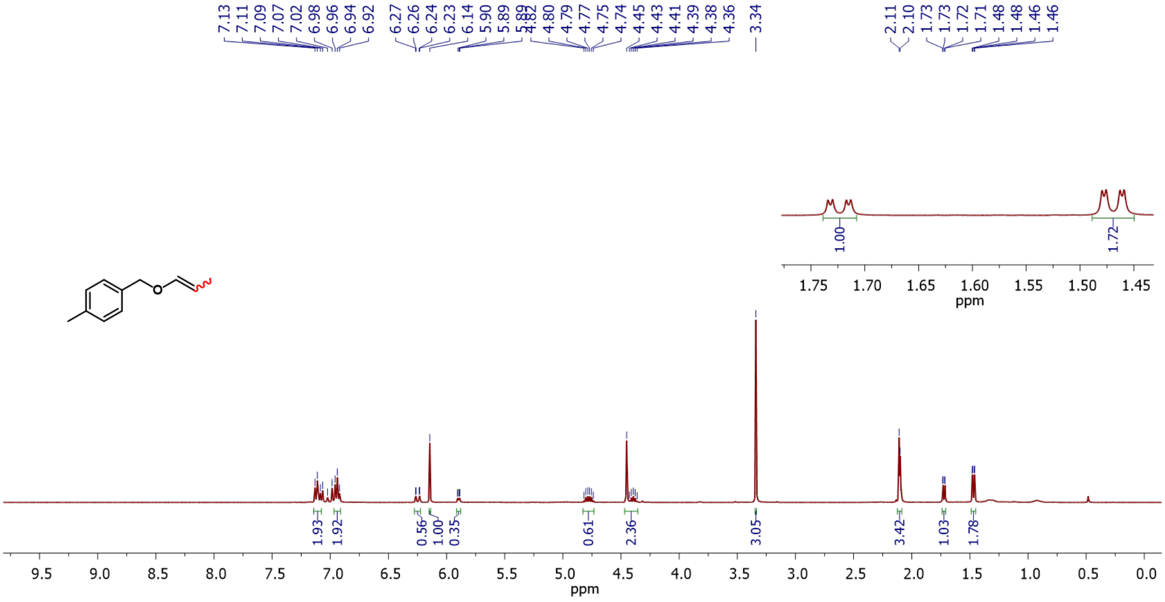

Figure S18. ^{1}H NMR spectrum (400 MHz) of **5b** in toluene-d_8 **(method B).**

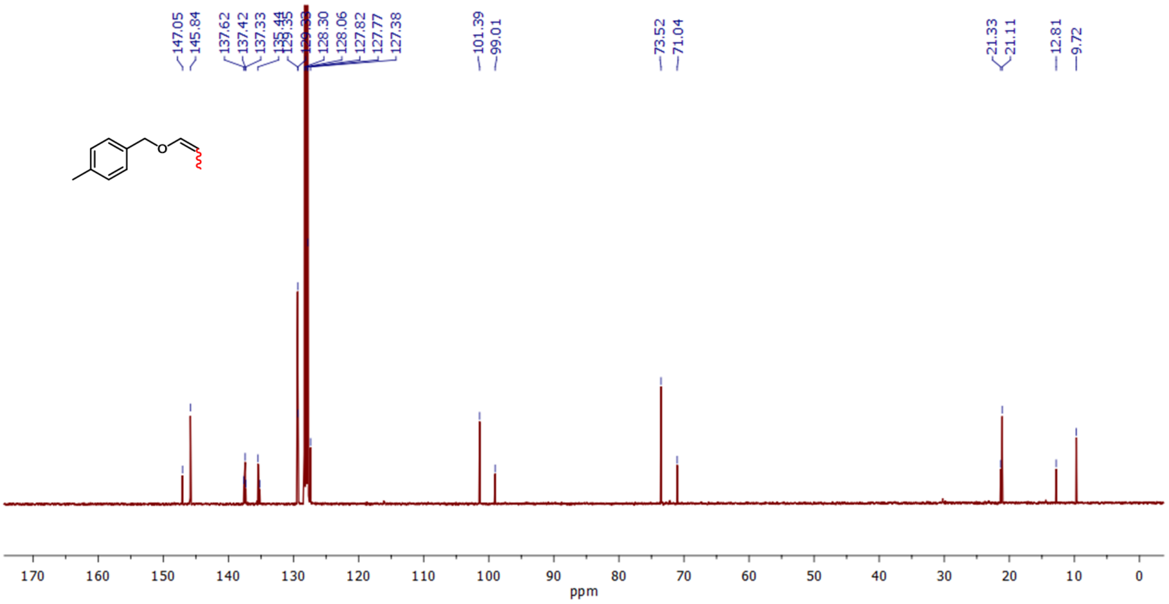

Figure S19. ^{13}C {^{1}H} NMR spectrum (101 MHz) of **5b** in benzene-d_6 (method A).

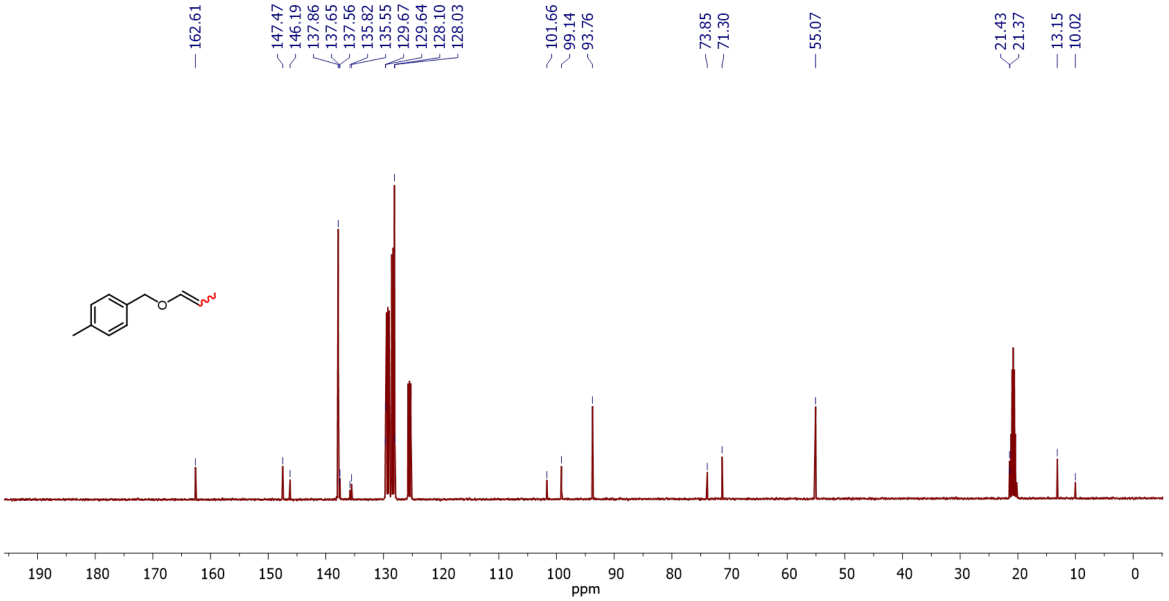

Figure S20. ^{13}C {^{1}H} NMR spectrum (101 MHz) of **5b** in toluene-d_8 (method B).

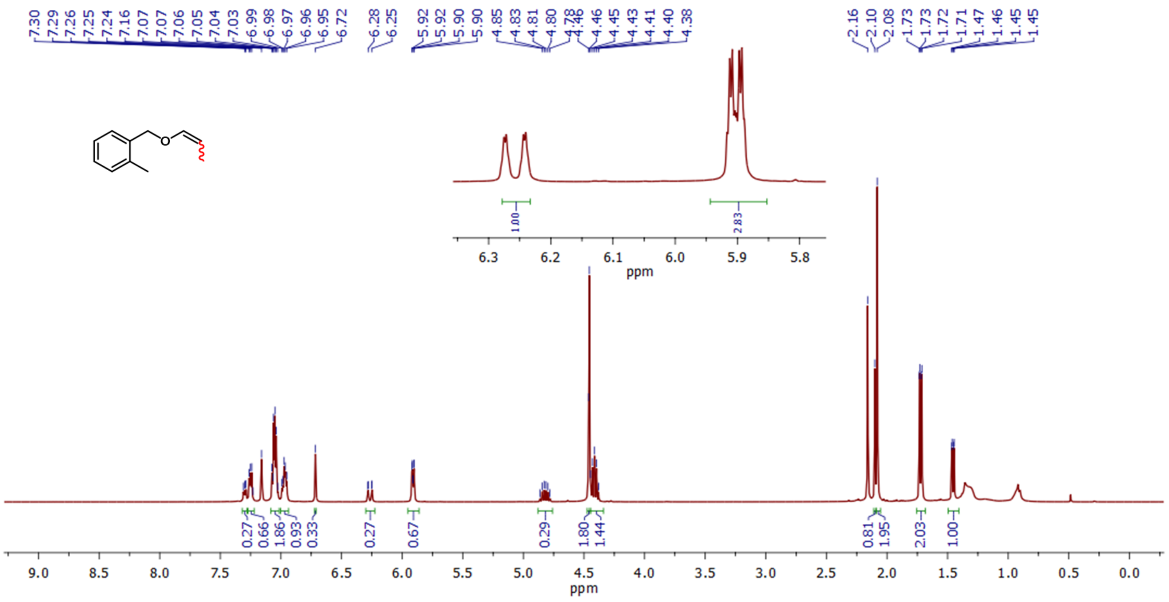

Figure S21. ^{1}H NMR spectrum (400 MHz) of **5c** in benzene-d_6 (method A).

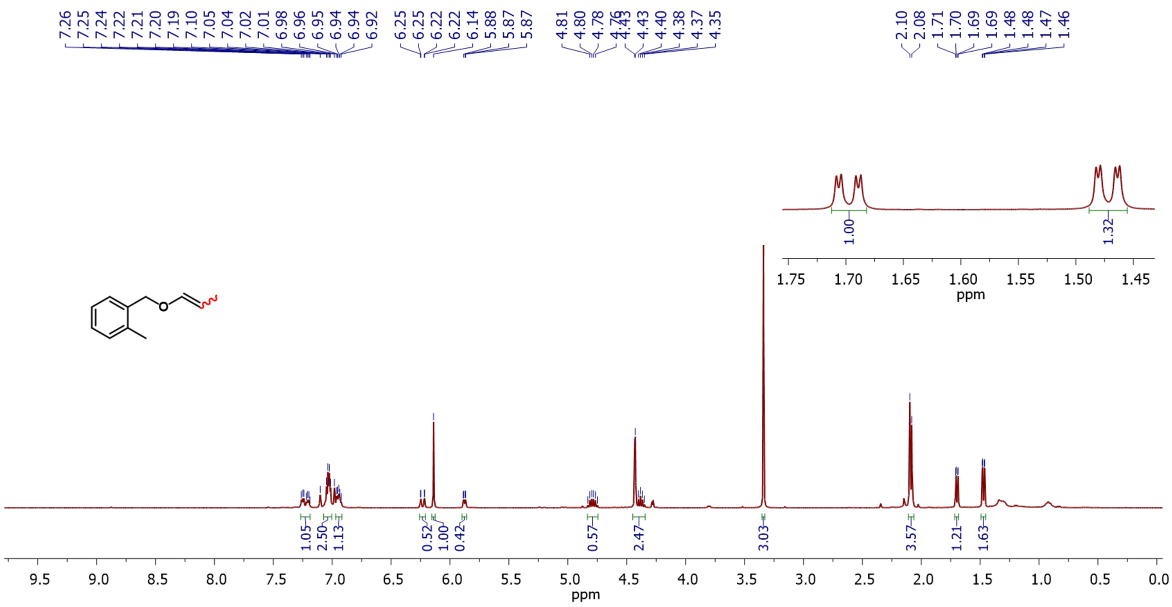

Figure S22. ^{1}H NMR spectrum (400 MHz) of **5c** in toluene-d_8 (method B).

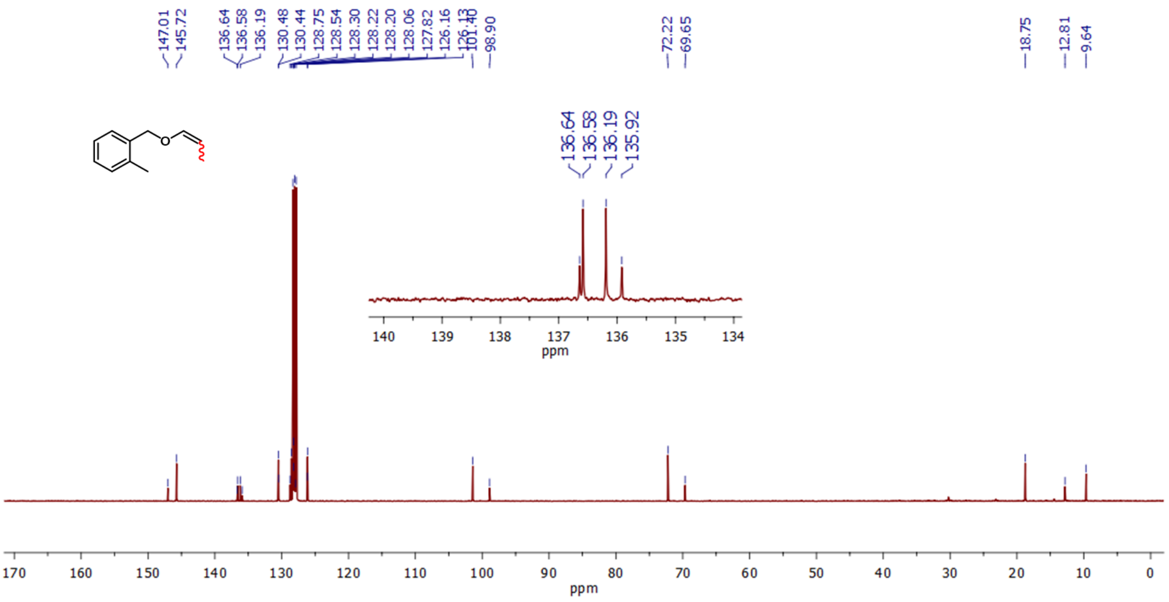

Figure S23. $^{13}C\ \{^{1}H\}$ NMR spectrum (101 MHz) of **5c** in benzene-d_6 (method A).

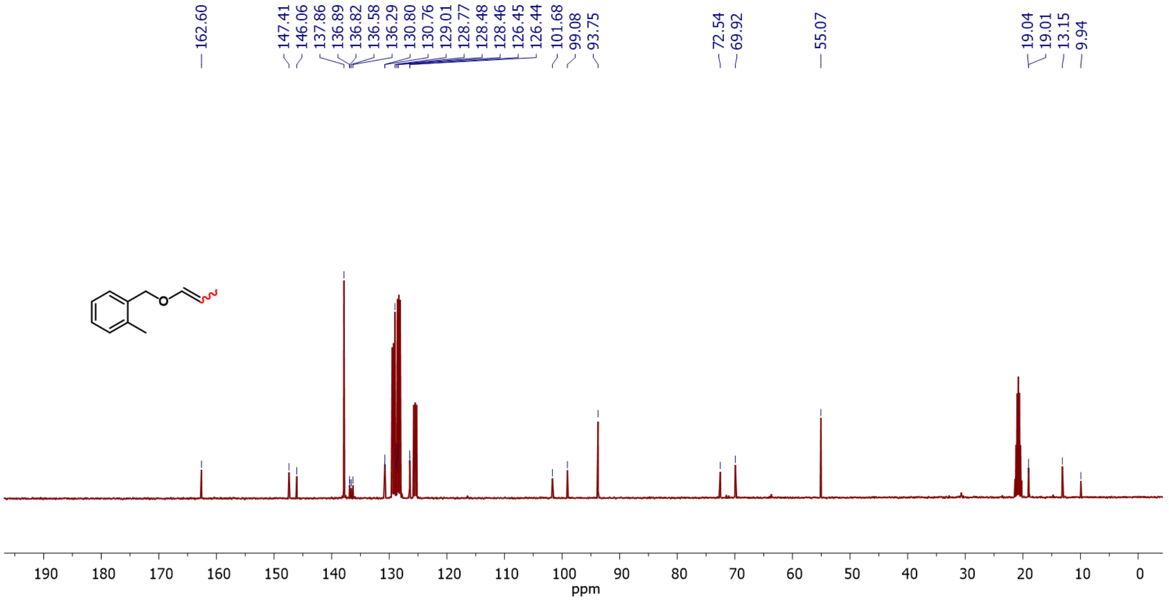

Figure S24. $^{13}C\ \{^{1}H\}$ NMR spectrum (101 MHz) of **5c** in toluene-d_8 (method B).

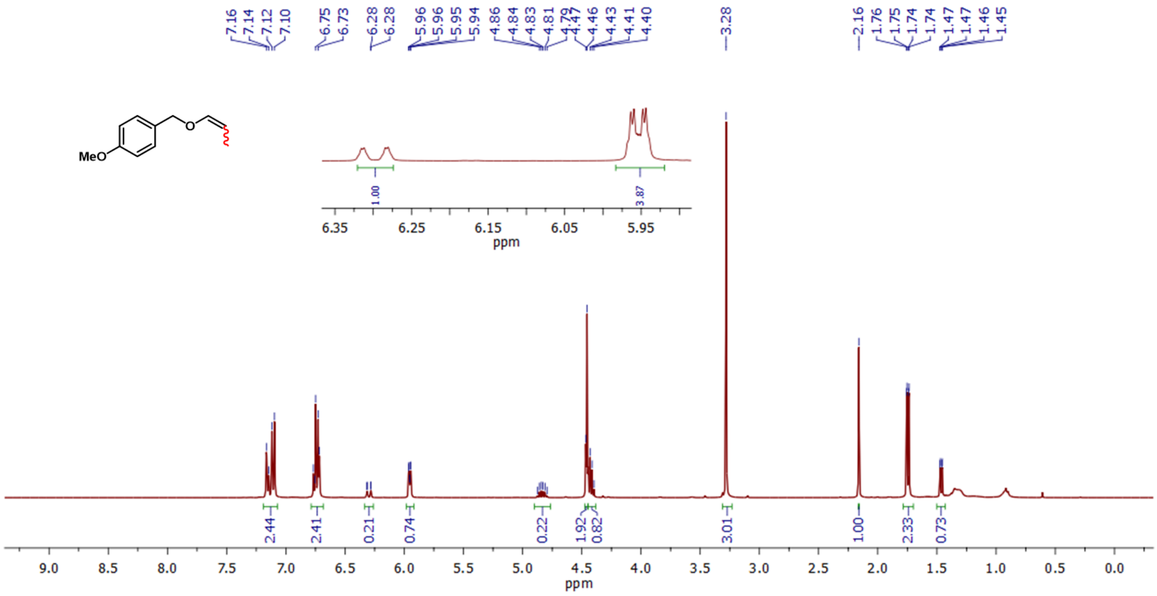

Figure S25. ^{1}H NMR spectrum (400 MHz) of **5d** in benzene-d_6 (method A).

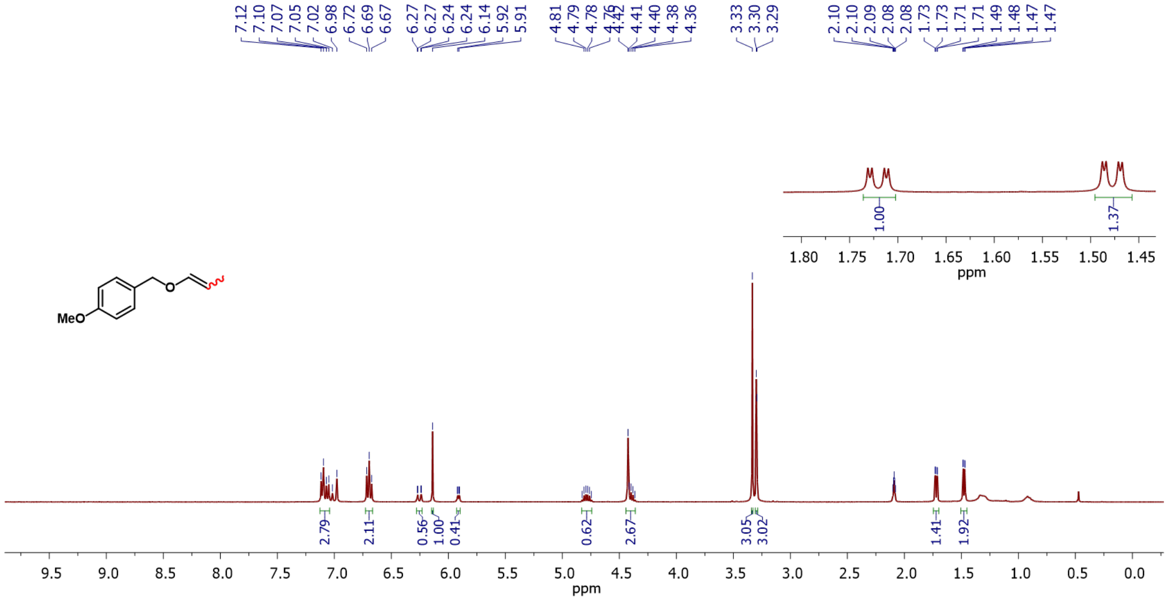

Figure S26. ^{1}H NMR spectrum (400 MHz) of **5d** in toluene-d_8 (method B).

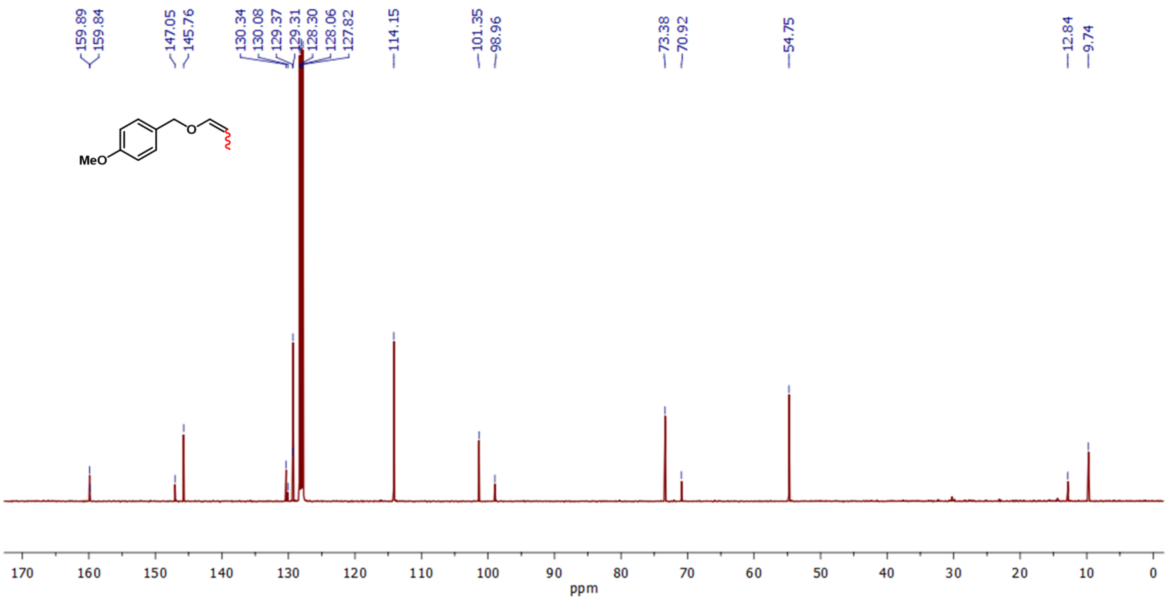

Figure S27. ^{13}C {^{1}H} NMR spectrum (101 MHz) of **5d** in benzene-d_6 (method A).

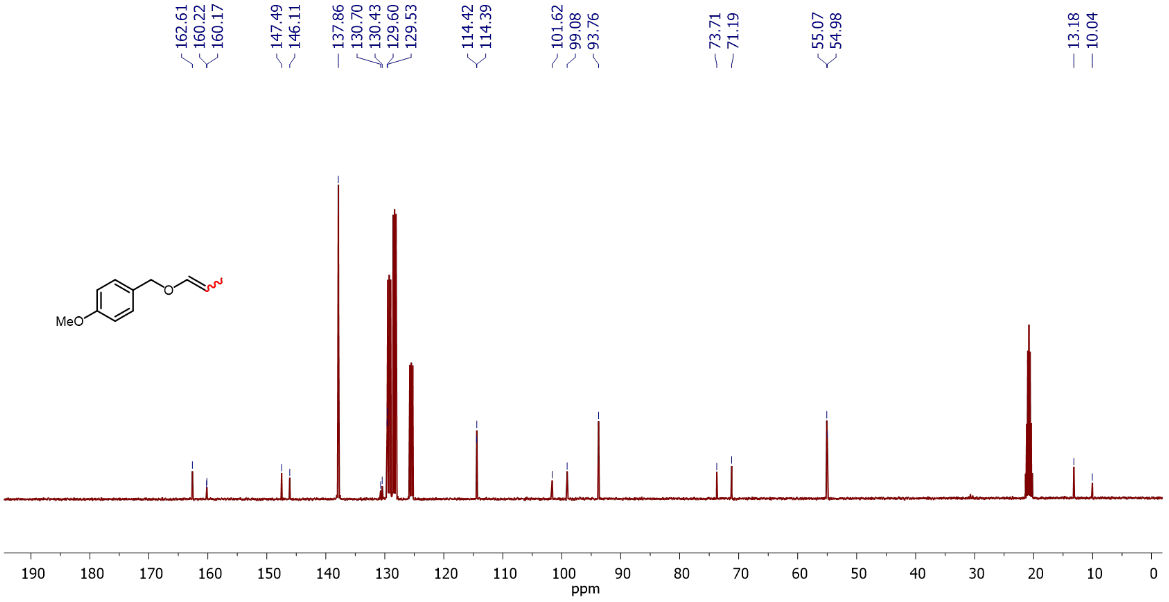

Figure S28. ^{13}C {^{1}H} NMR spectrum (101 MHz) of **5d** in toluene-d_8 (method B).

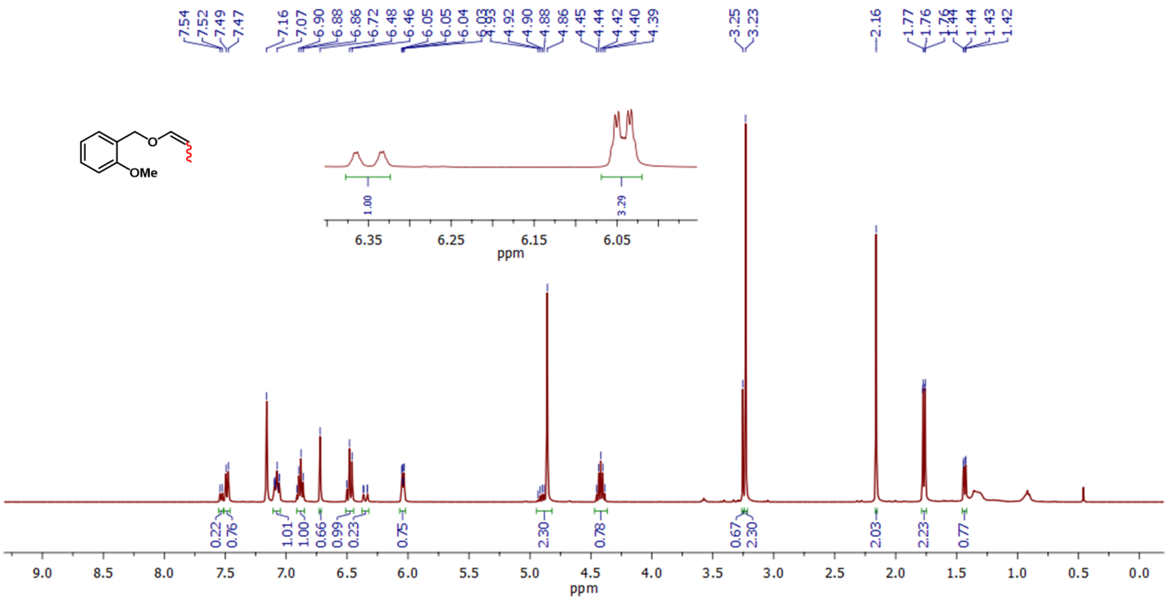

Figure S29. ^{1}H NMR spectrum (400 MHz) of **5e** in benzene-d_6 (method A).

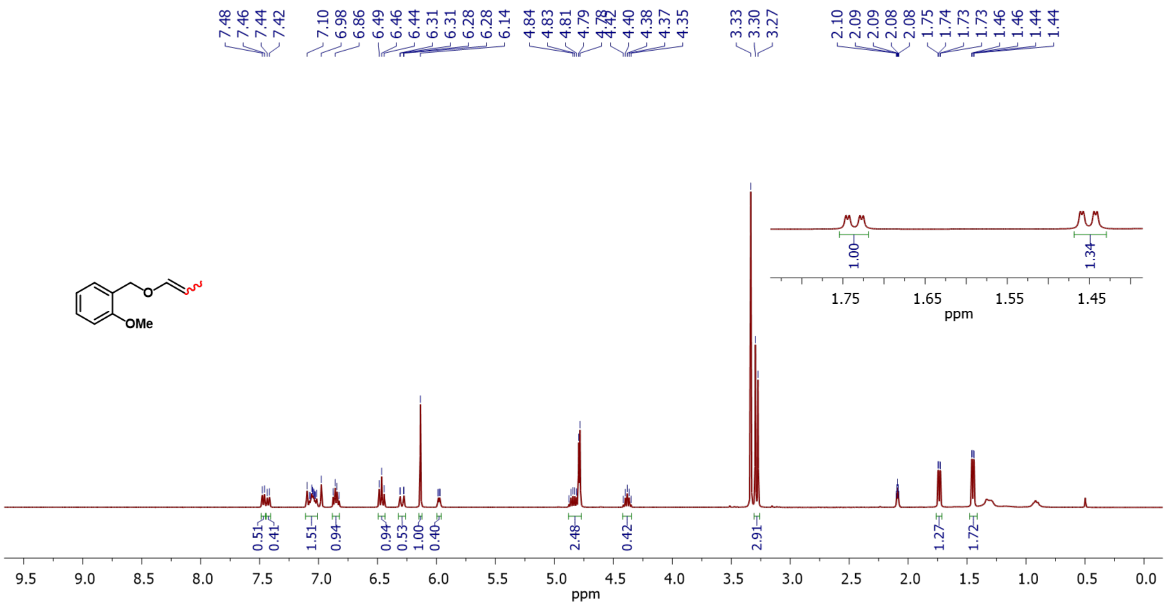

Figure S30. ^{1}H NMR spectrum (400 MHz) of **5e** in toluene-d_8 (method B).

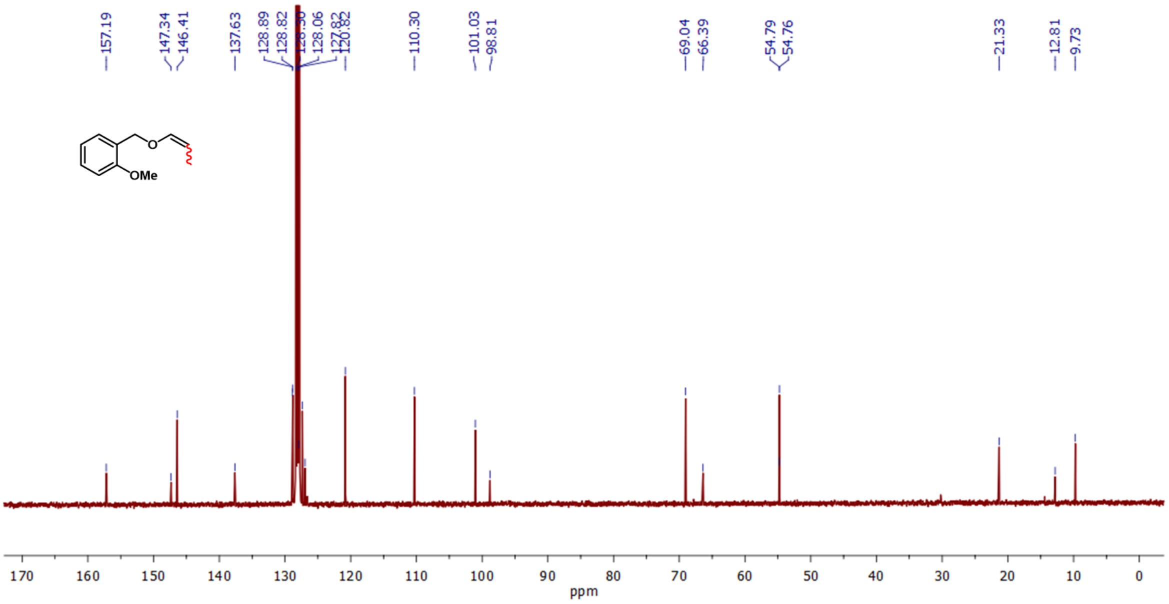

Figure S31. ^{13}C {^{1}H} NMR spectrum (101 MHz) of **5e** in benzene-d_6 (method A).

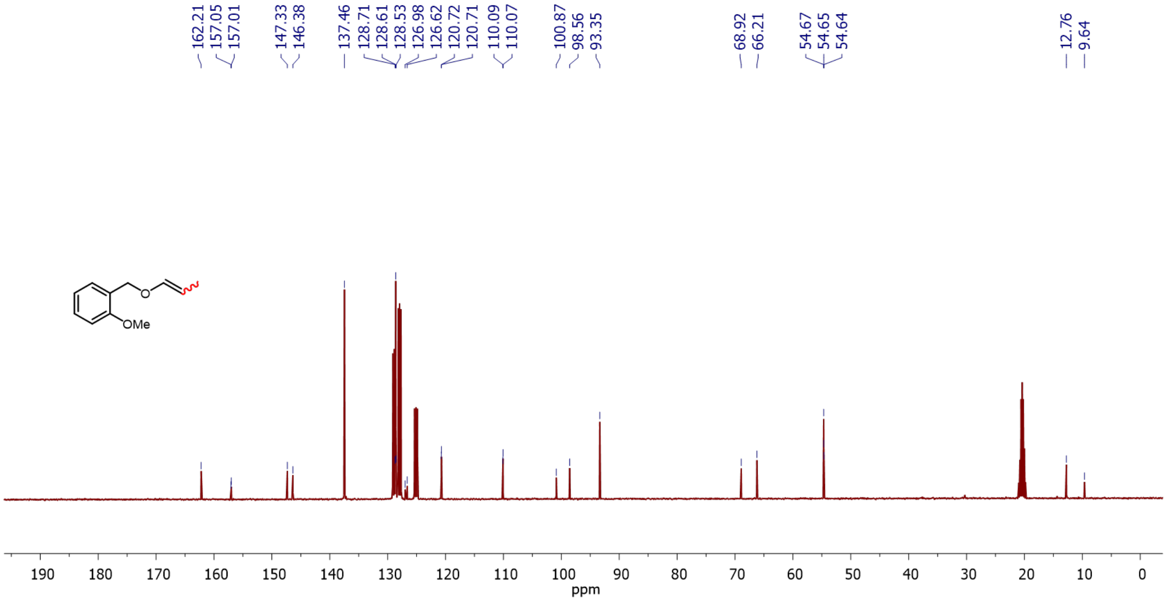

Figure S32. ^{13}C {^{1}H} NMR spectrum (101 MHz) of **5e** in toluene-d_8 (method B).

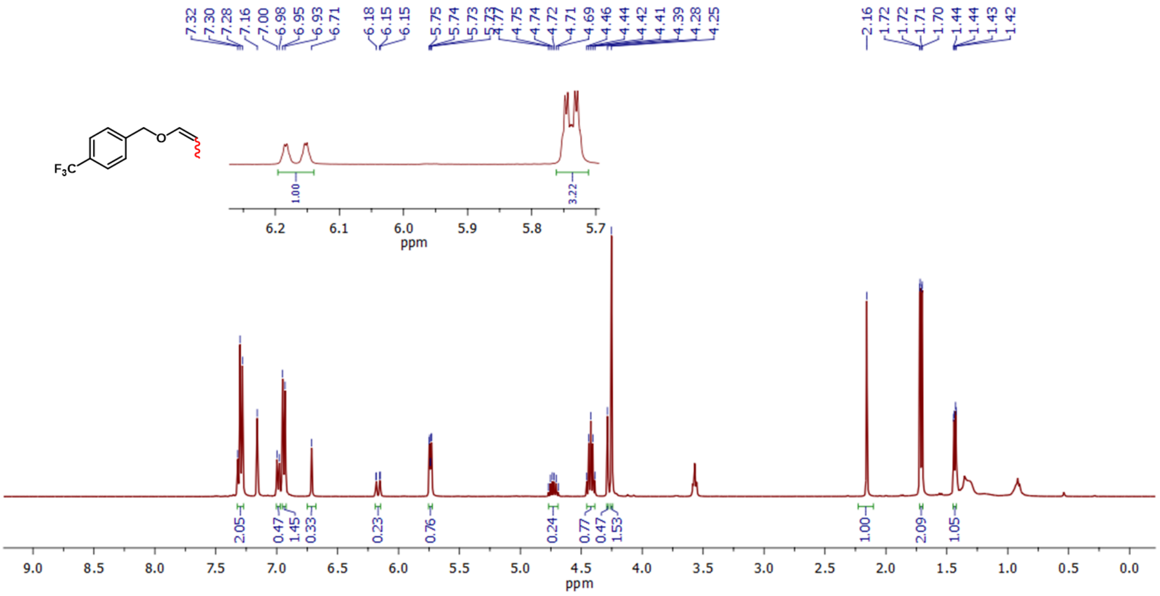

Figure S33. ^{1}H NMR spectrum (400 MHz) of **5f** in benzene-d_6 (method A).

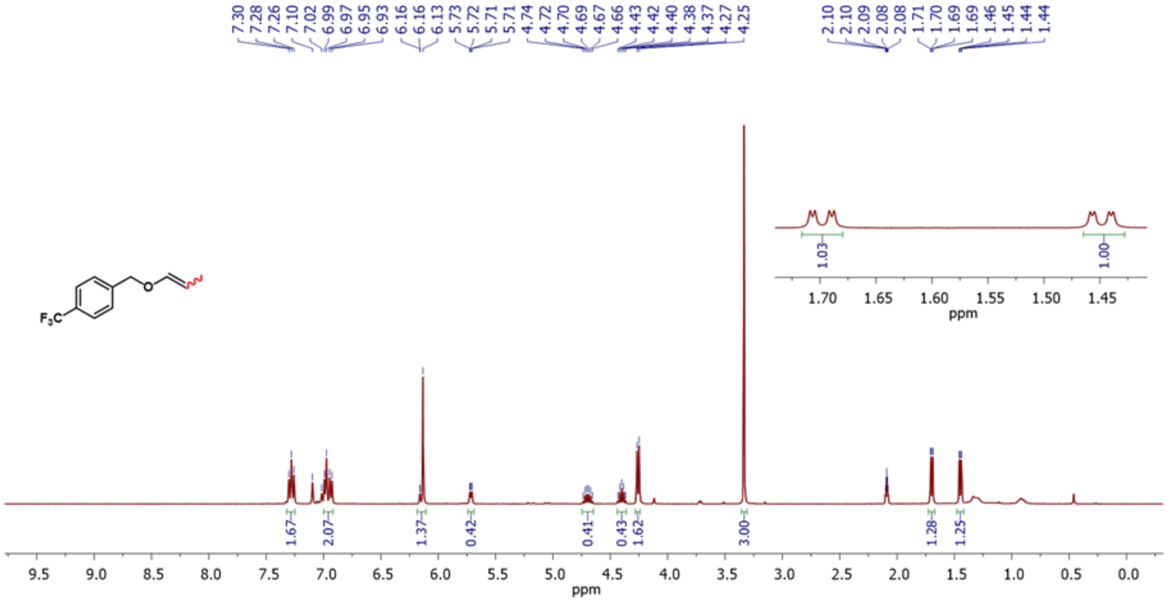

Figure S34. ^{1}H NMR spectrum (400 MHz) of **5f** in toluene-d_8 (method B).

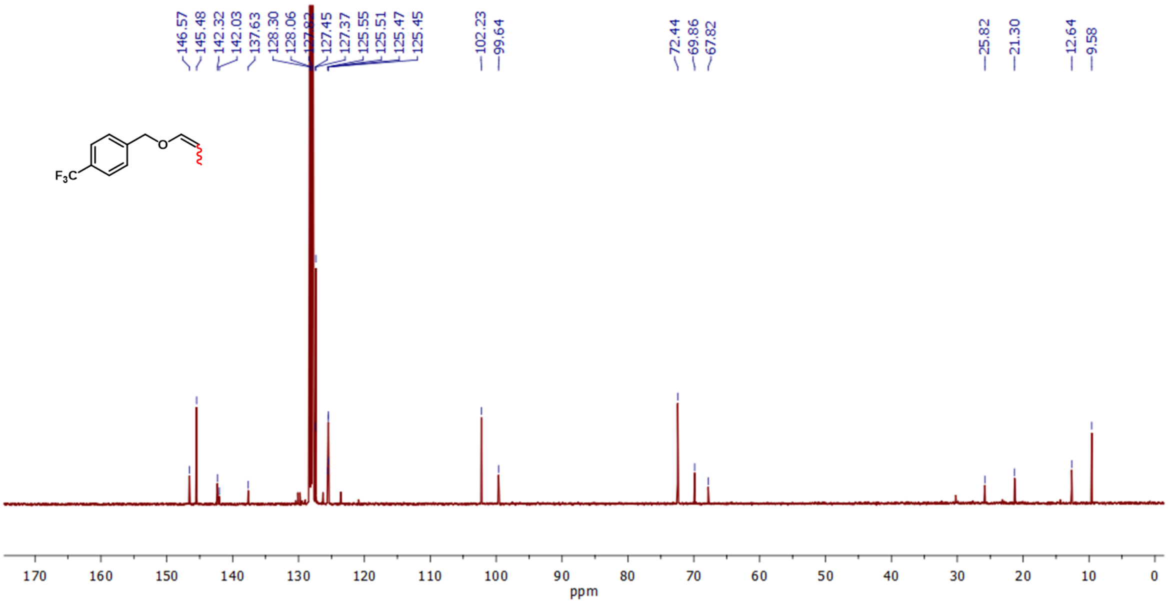

Figure S35. $^{13}C\ \{^1H\}$ NMR spectrum (101 MHz) of **5f** in benzene-d_6 (method A).

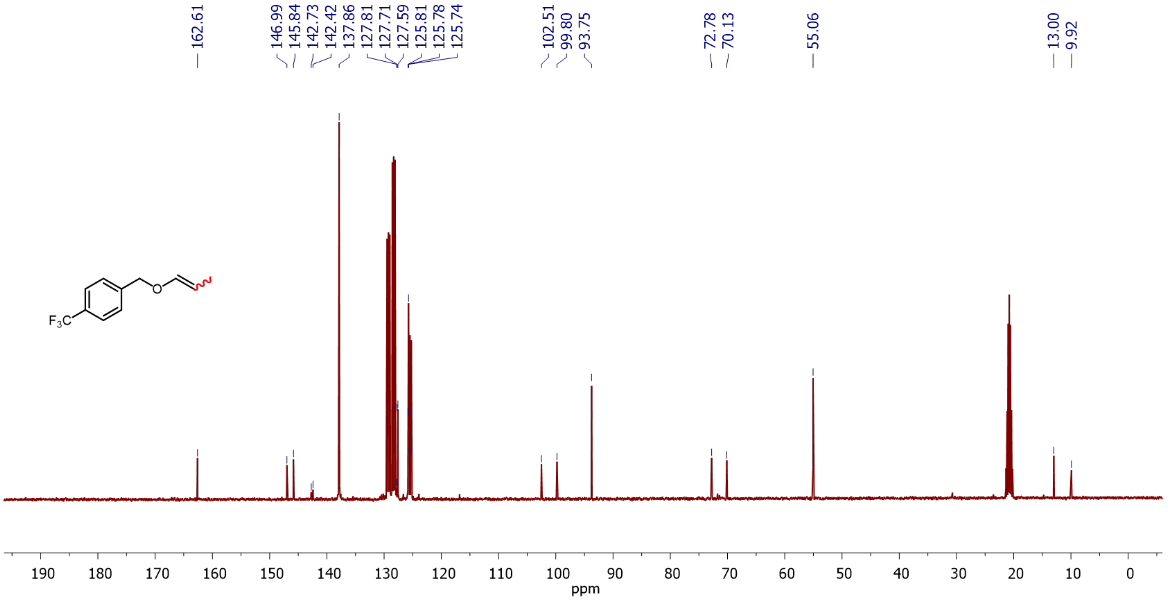

Figure S36. $^{13}C\ \{^1H\}$ NMR spectrum (101 MHz) of **5f** in toluene-d_8 (method B).

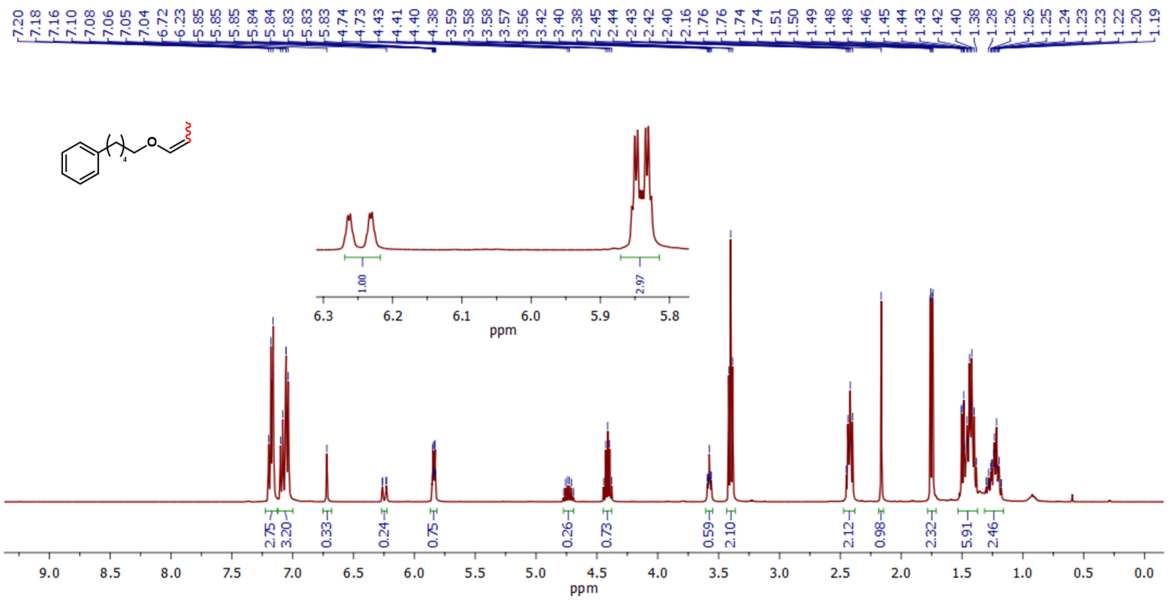

Figure S37. ^{1}H NMR spectrum (400 MHz) of **5g** in benzene-d_6 (method A).

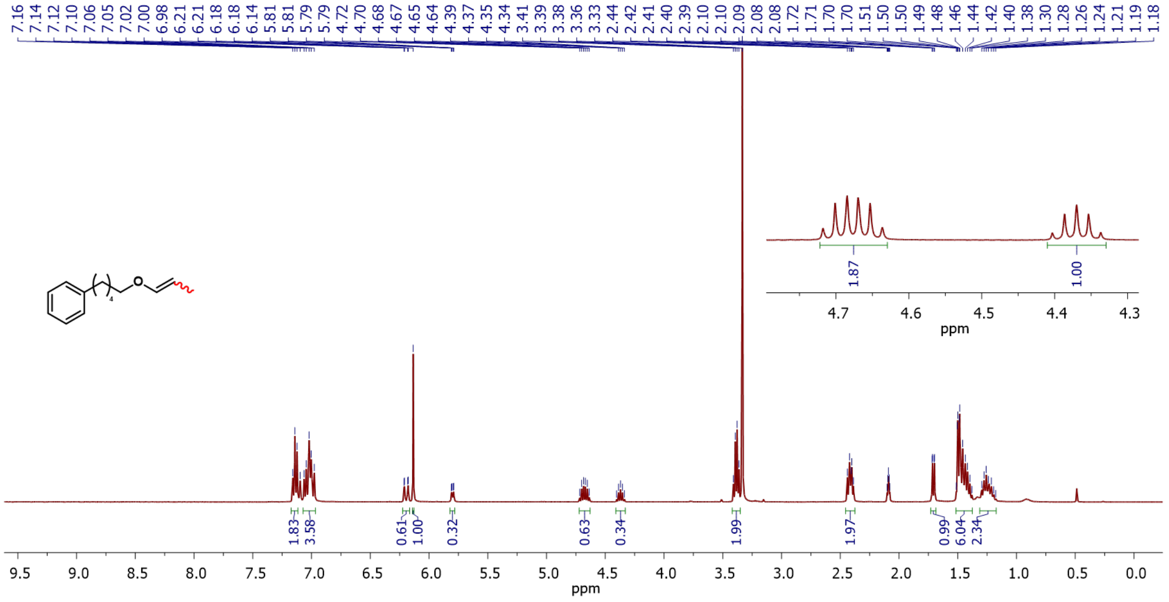

Figure S38. ^{1}H NMR spectrum (400 MHz) of **5g** in toluene-d_8 (method B).

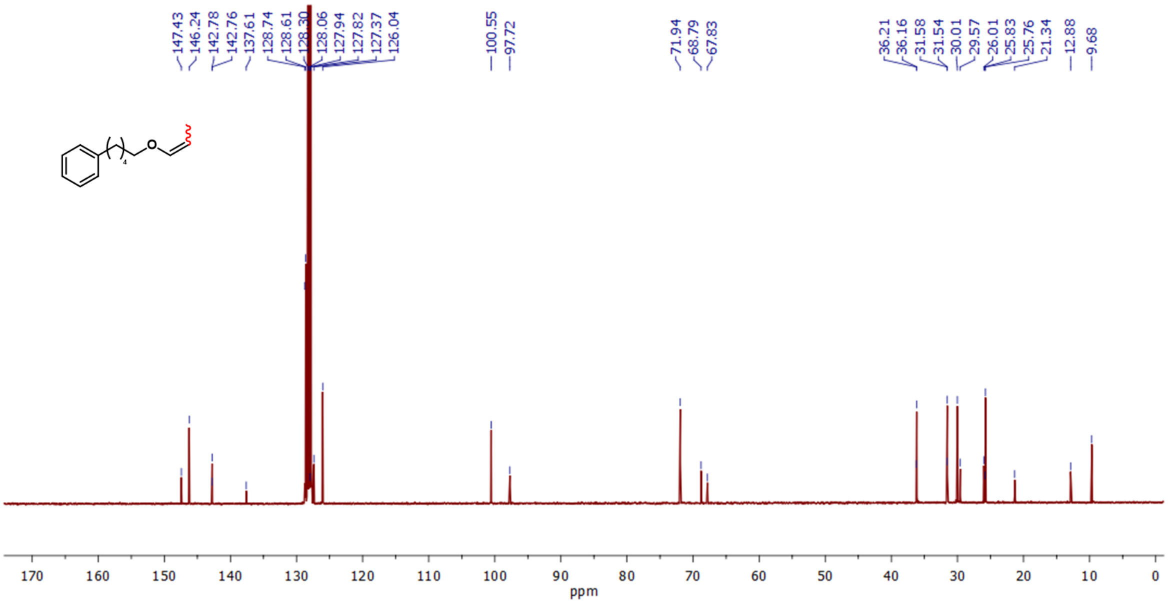

Figure S39. ^{13}C {^{1}H} NMR spectrum (101 MHz) of **5g** in benzene-d_6 (method A).

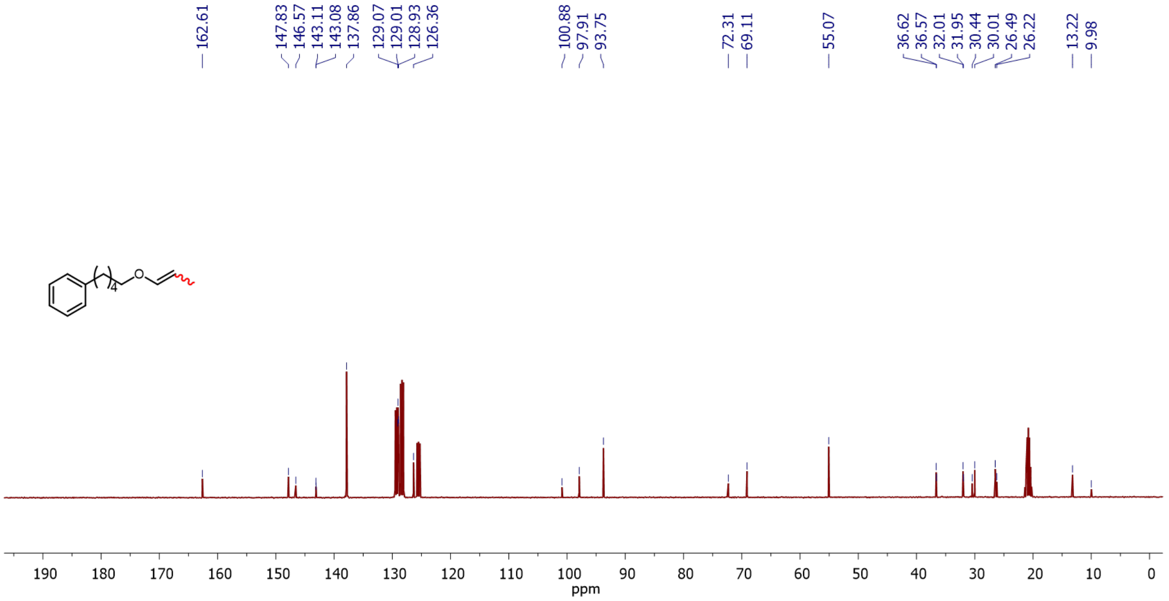

Figure S40. ^{13}C {^{1}H} NMR spectrum (101 MHz) of **5g** in toluene-d_8 (method B).

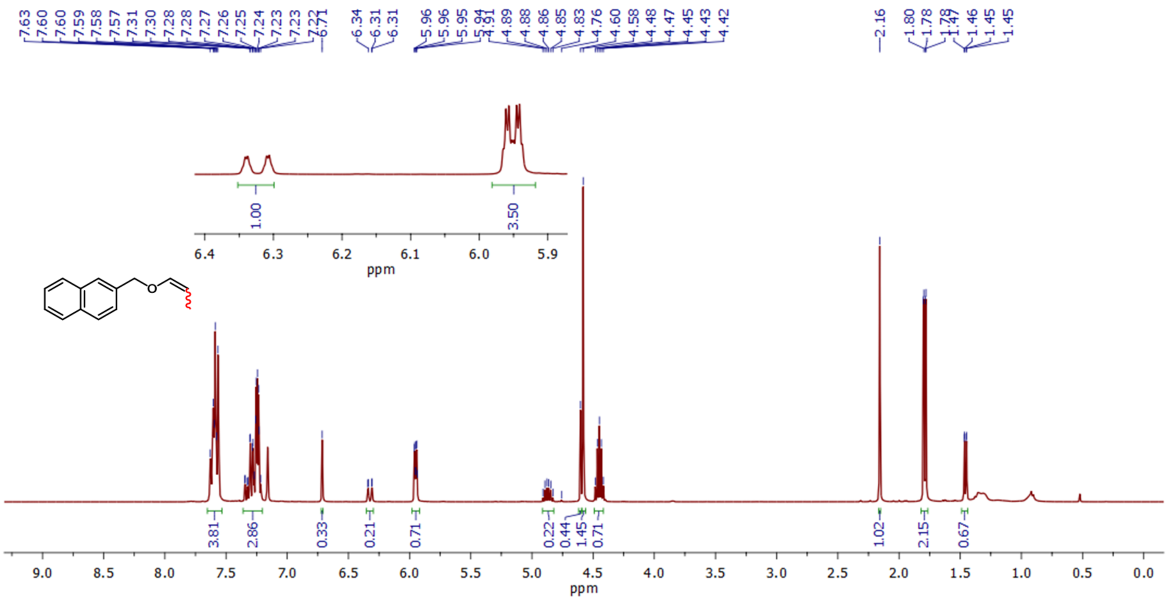

Figure S41. ^{1}H NMR spectrum (400 MHz) of **5h** in benzene-d_6 (method A).

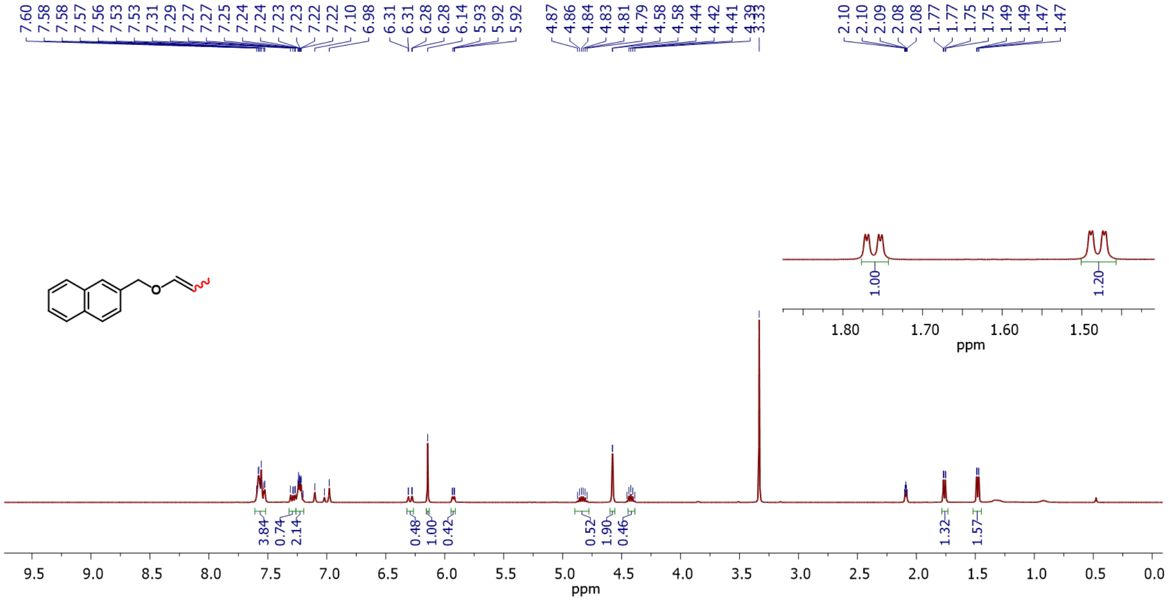

Figure S42. ^{1}H NMR spectrum (400 MHz) of **5h** in toluene-d_8 (method B).

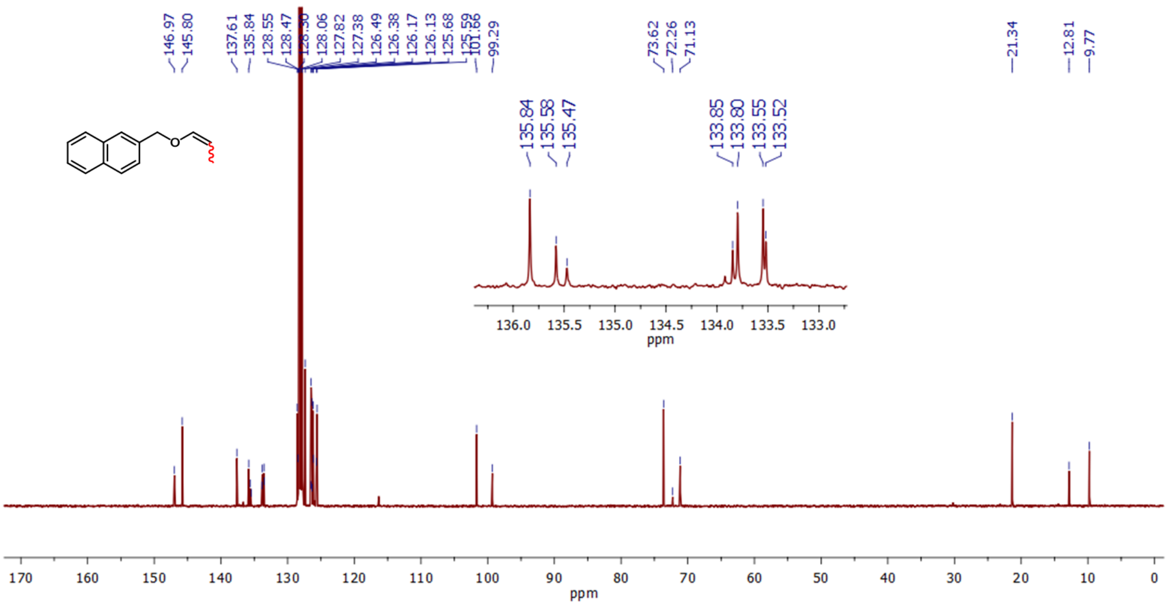

Figure S43. ^{13}C {^{1}H} NMR spectrum (101 MHz) of **5h** in benzene-d_6 (method A).

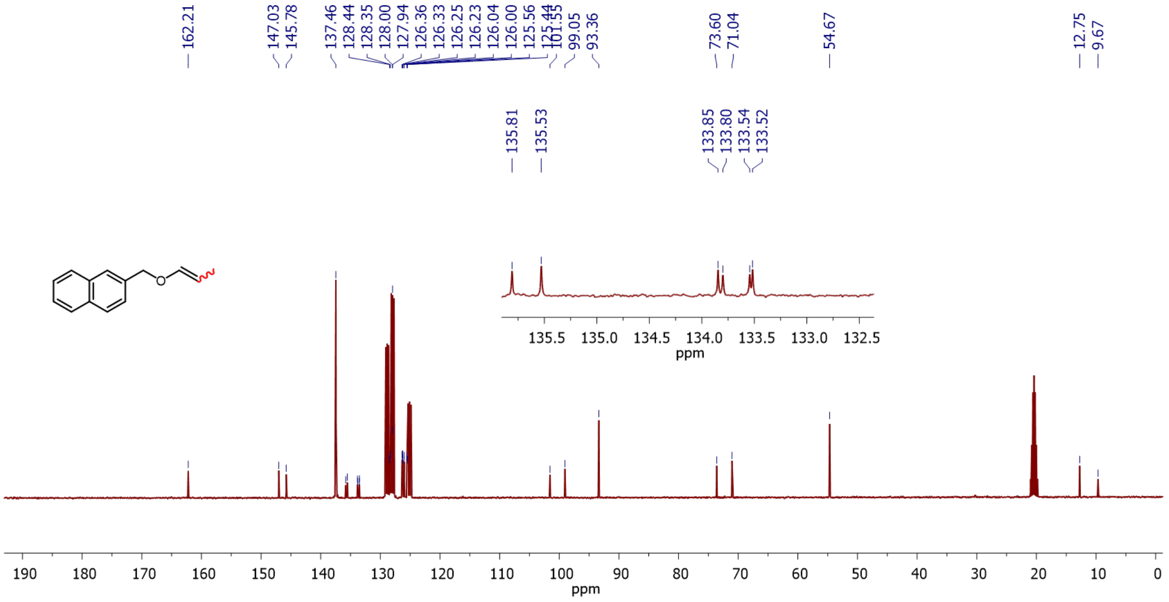

Figure S44. ^{13}C {^{1}H} NMR spectrum (101 MHz) of **5h** in toluene-d_8 (method B).

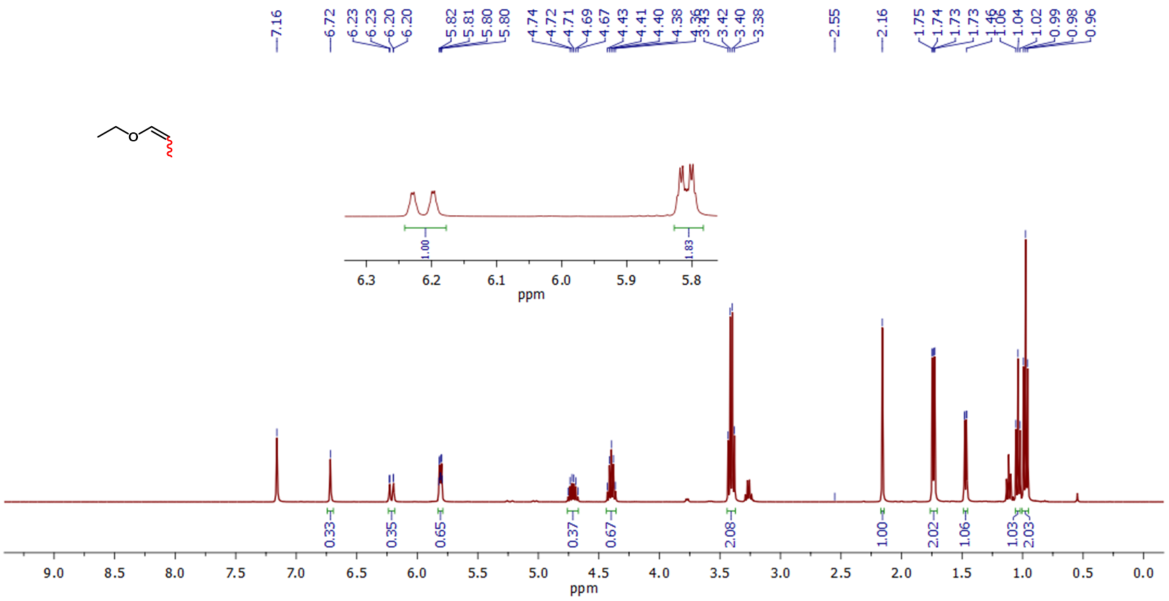

Figure S45. ^{1}H NMR spectrum (400 MHz) of **5i** in benzene-d_6 (method A).

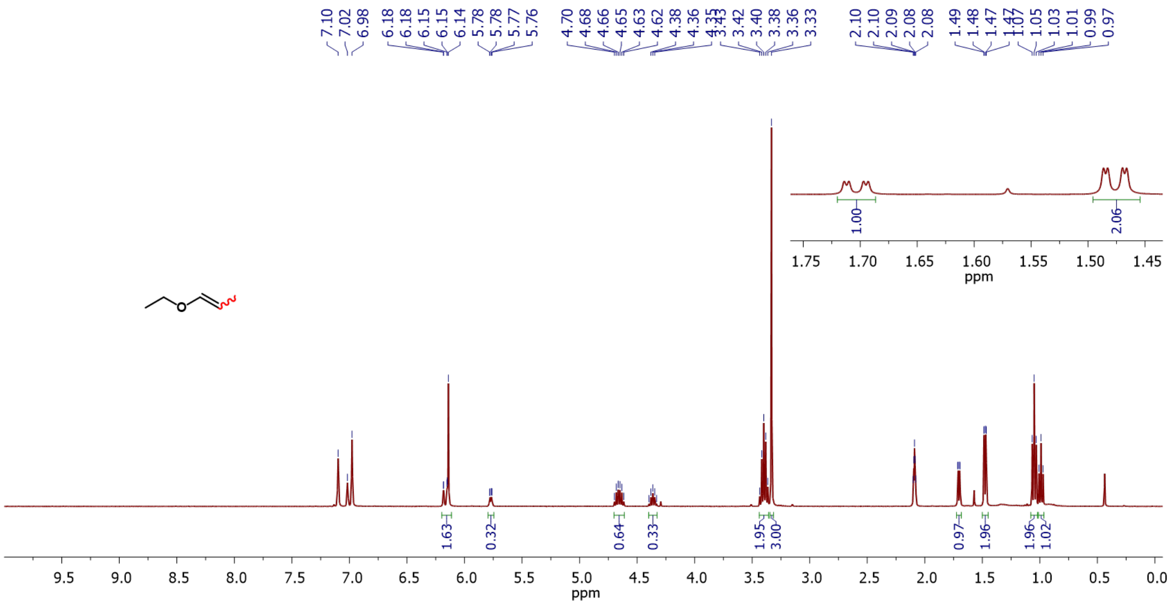

Figure S46. ^{1}H NMR spectrum (400 MHz) of **5i** in toluene-d_8 (method B).

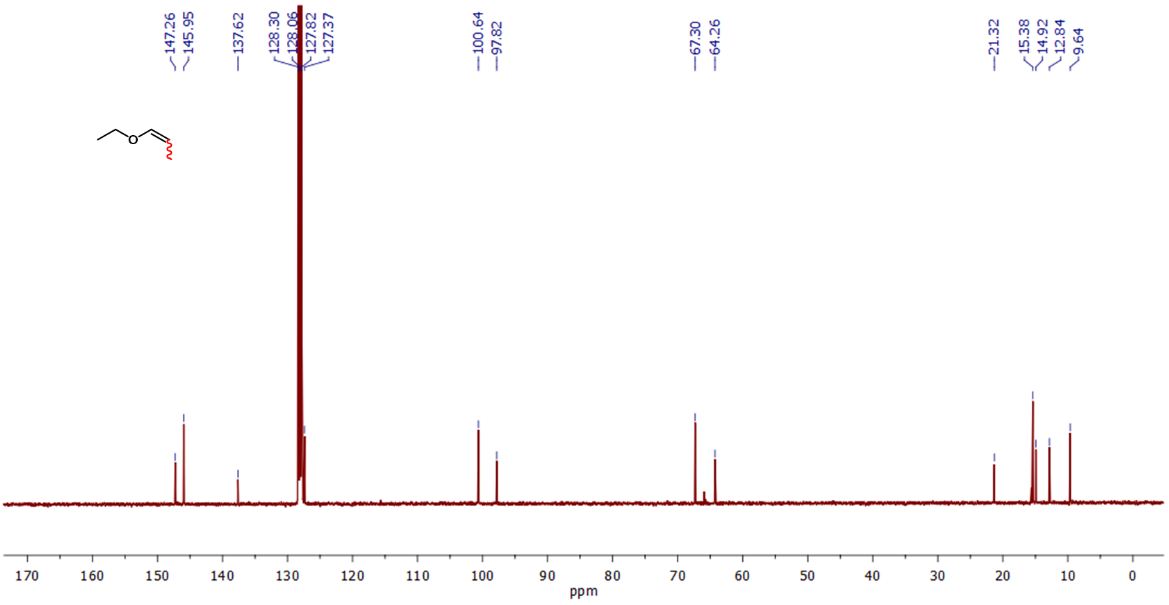

Figure S47. ^{1}H NMR spectrum (101 MHz) of **5i** in benzene-d_6 (method A).

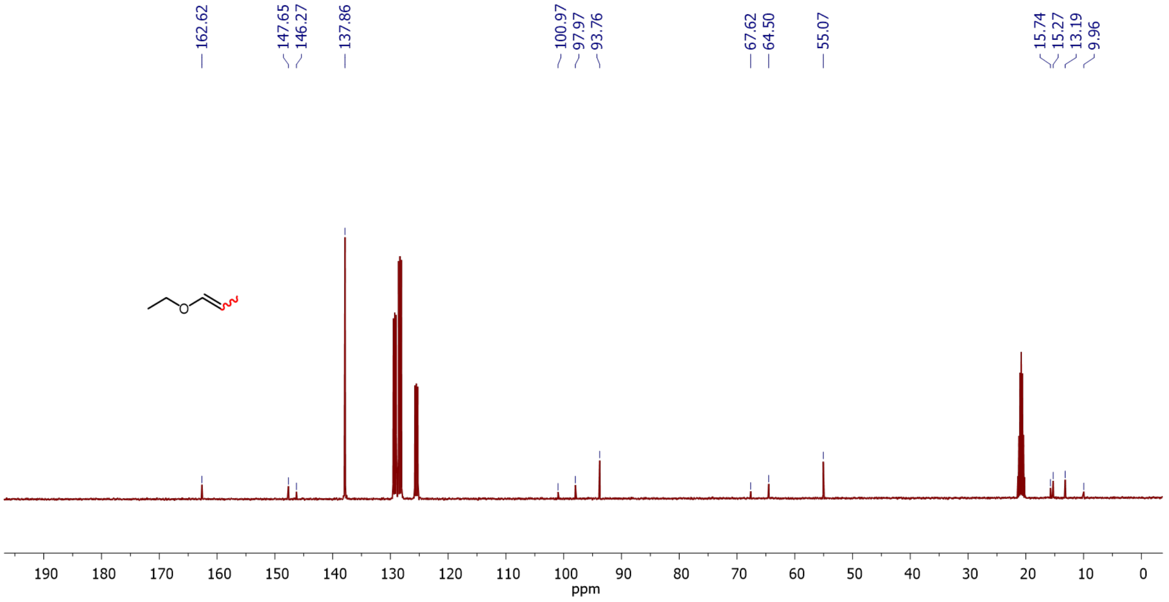

Figure S48. ^{13}C {^{1}H} NMR spectrum (101 MHz) of **5i** in toluene-d_8 (method B).

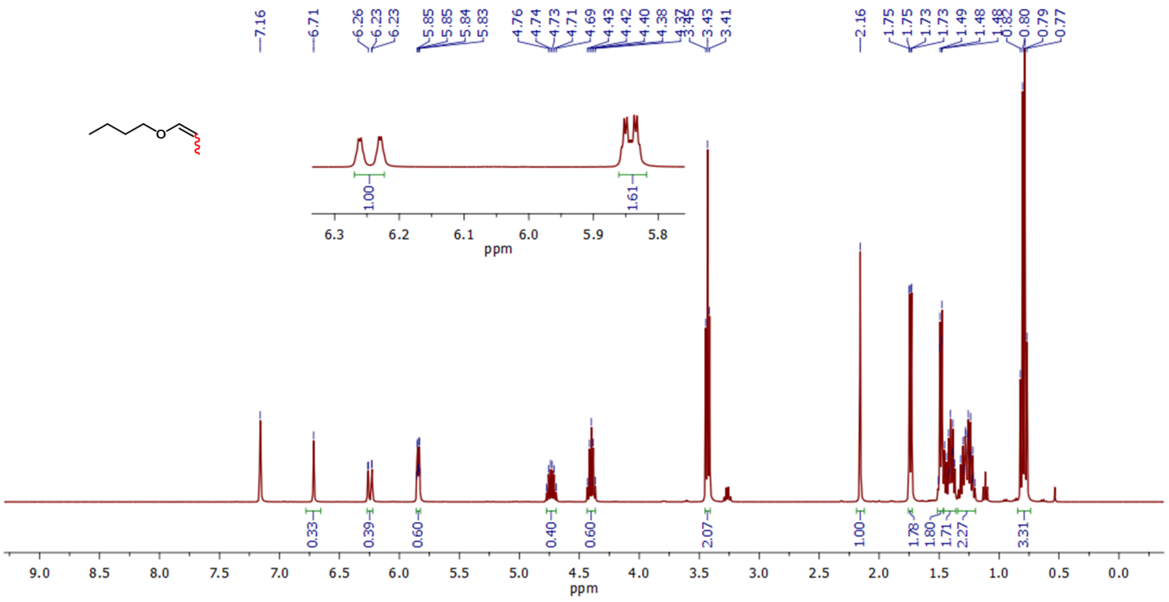

Figure S49. ^{1}H NMR spectrum (400 MHz) of **5j** in benzene-d_6 (method A).

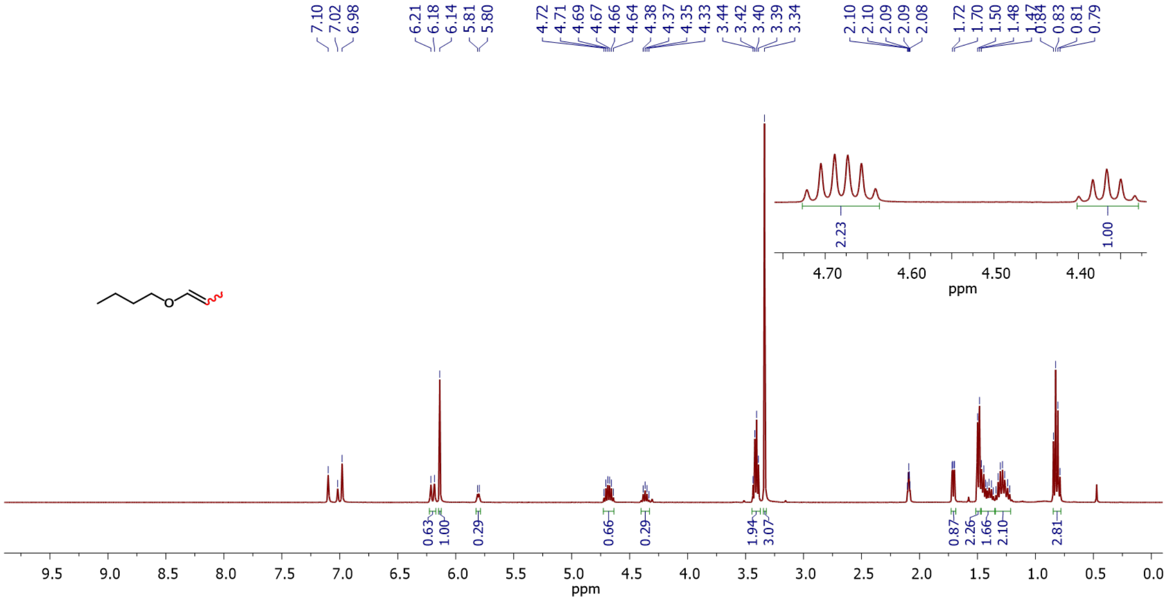

Figure S50. ^{1}H NMR spectrum (400 MHz) of **5j** in toluene-d_8 (method B).

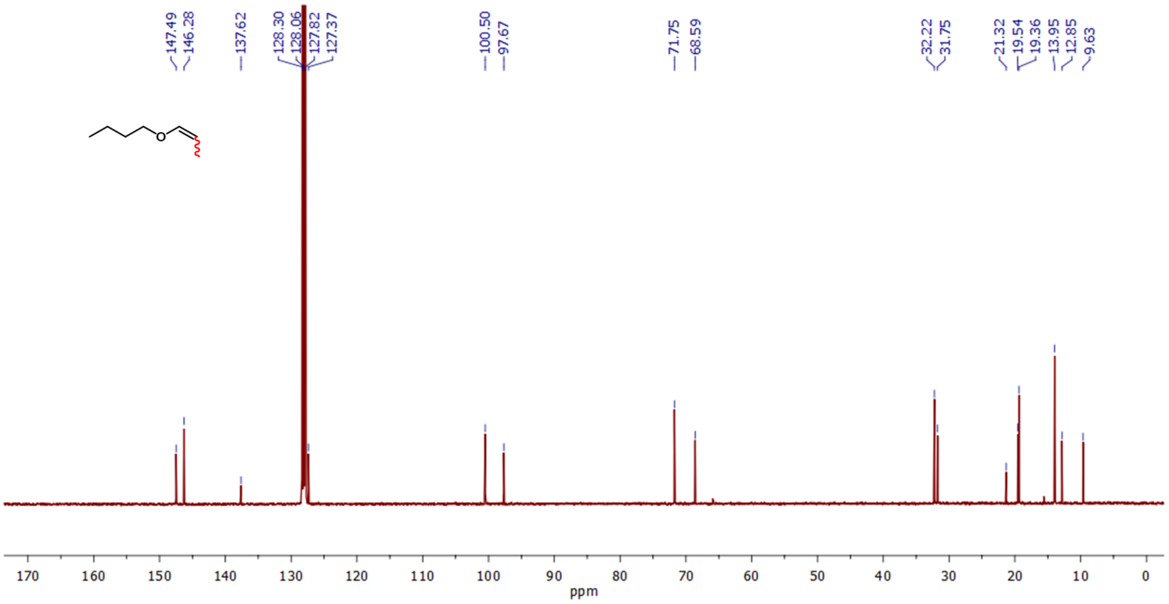

Figure S51. ^{13}C {^{1}H} NMR spectrum (101 MHz) of **5j** in benzene-d_6 (method A).

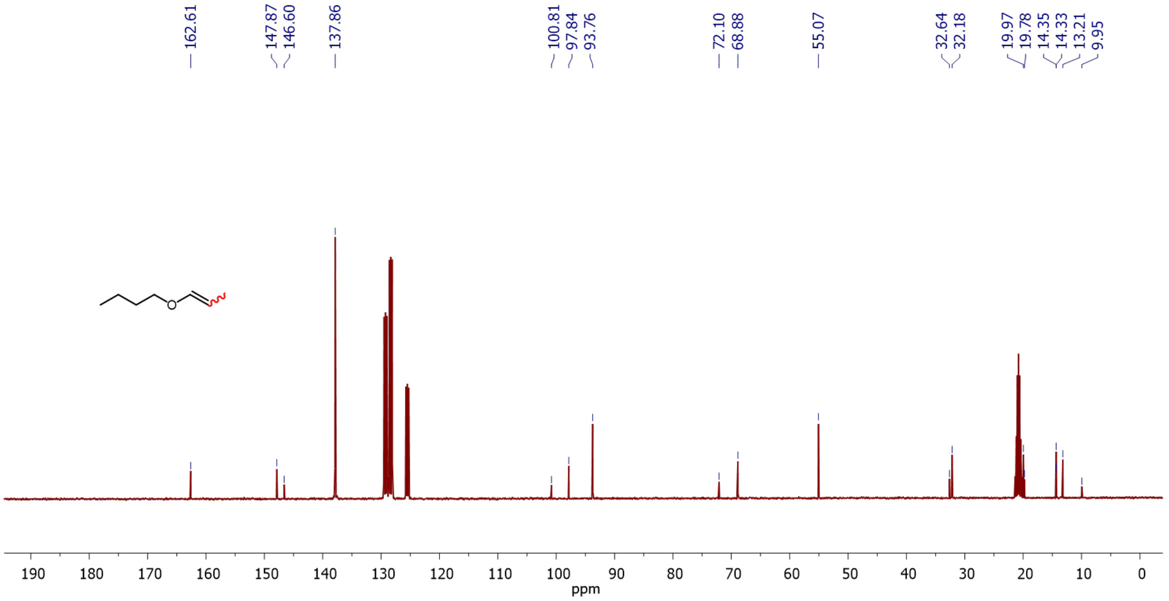

Figure S52. ^{13}C {^{1}H} NMR spectrum (101 MHz) of **5j** in toluene-d_8 (method B).

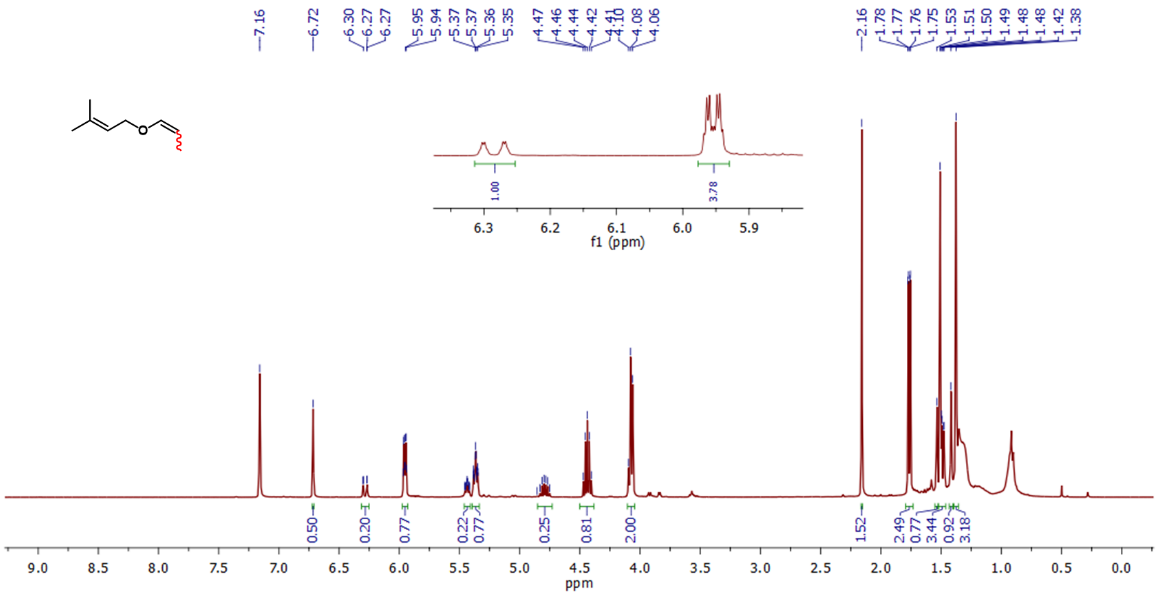

Figure S53. ^{1}H NMR spectrum (400 MHz) of **5k** in benzene-d_6 (method A).

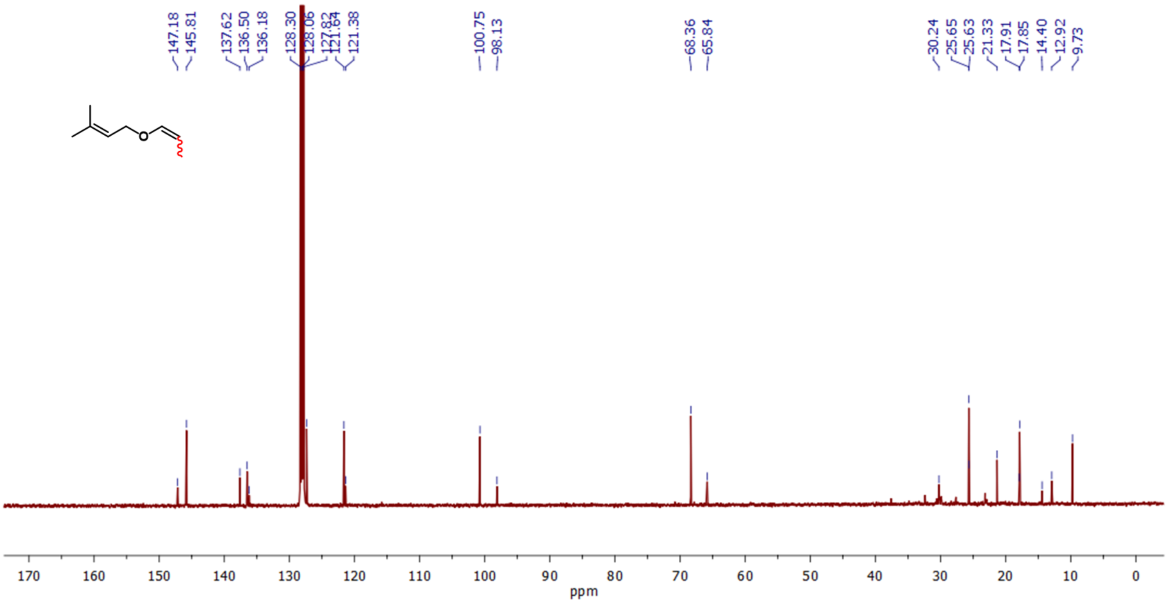

Figure S54. ^{13}C {^{1}H} NMR spectrum (101 MHz) of **5k** in benzene-d_6 (method A).

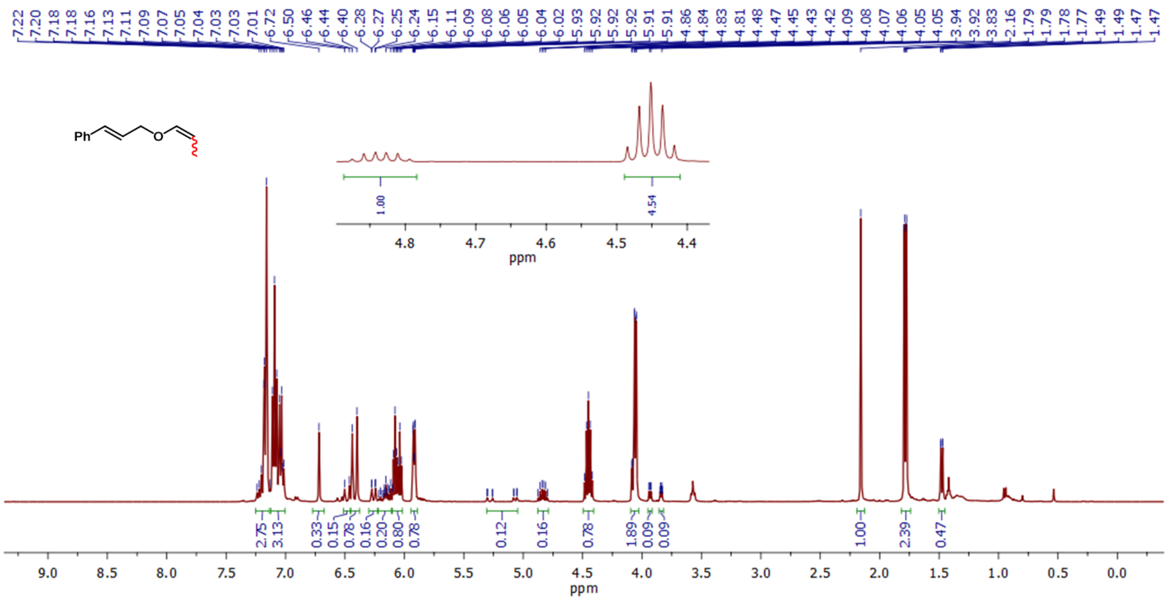

Figure S55. ^{1}H NMR spectrum (400 MHz) of **5l** in benzene-d_6 (method A).

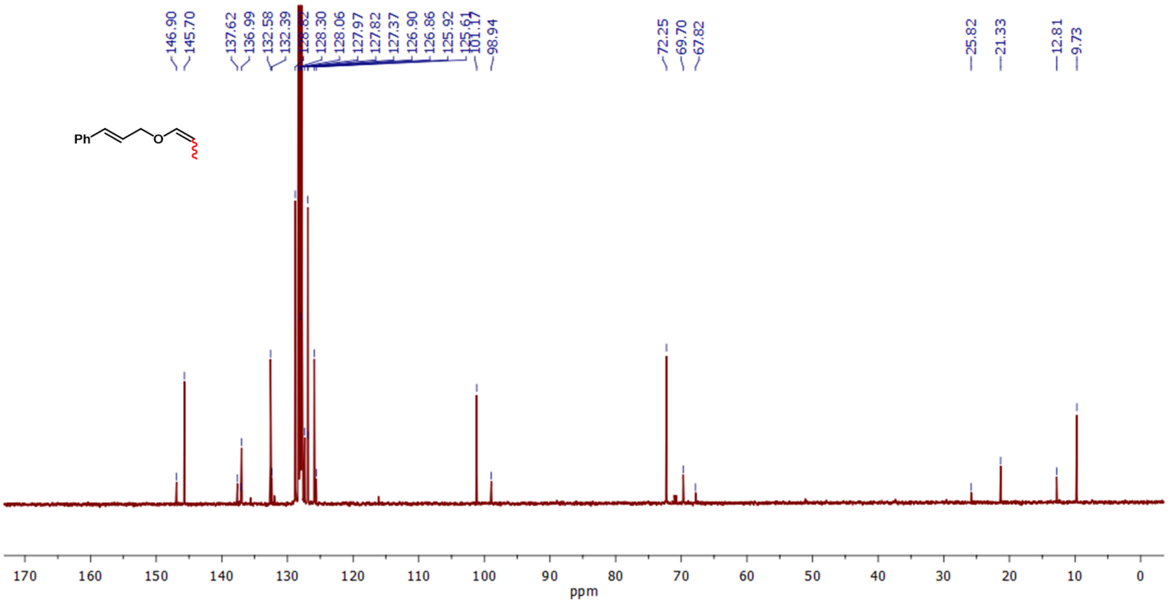

Figure S56. ^{13}C {^{1}H} NMR spectrum (101 MHz) of **5l** in benzene-d_6 (method A).

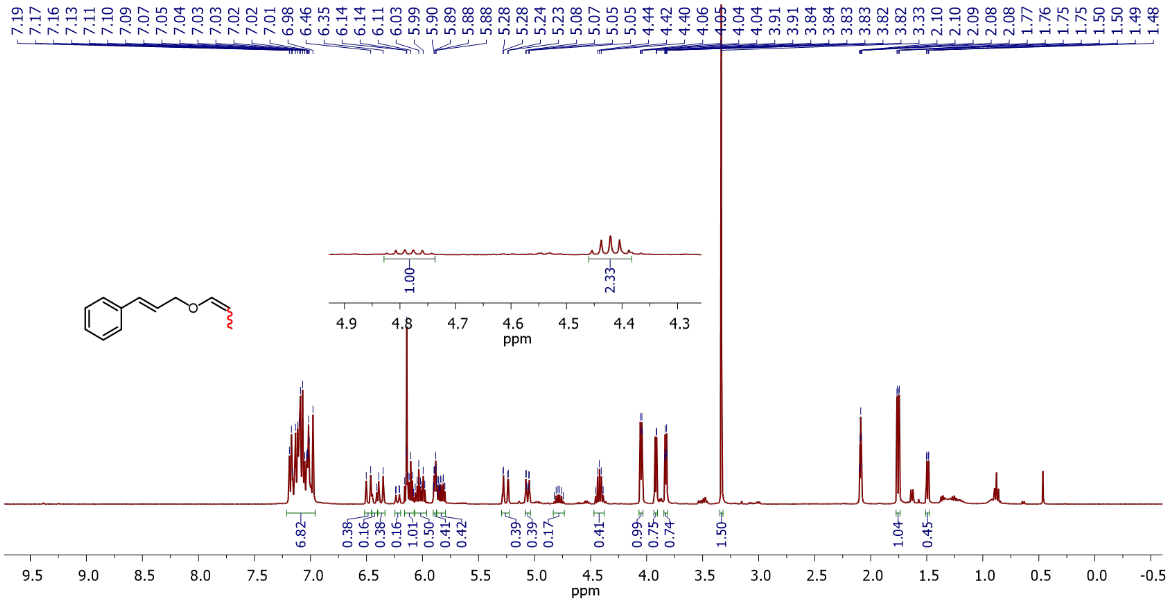

Figure S57. ^{1}H NMR spectrum (400 MHz) of **5l** in toluene-d_8 (method B).

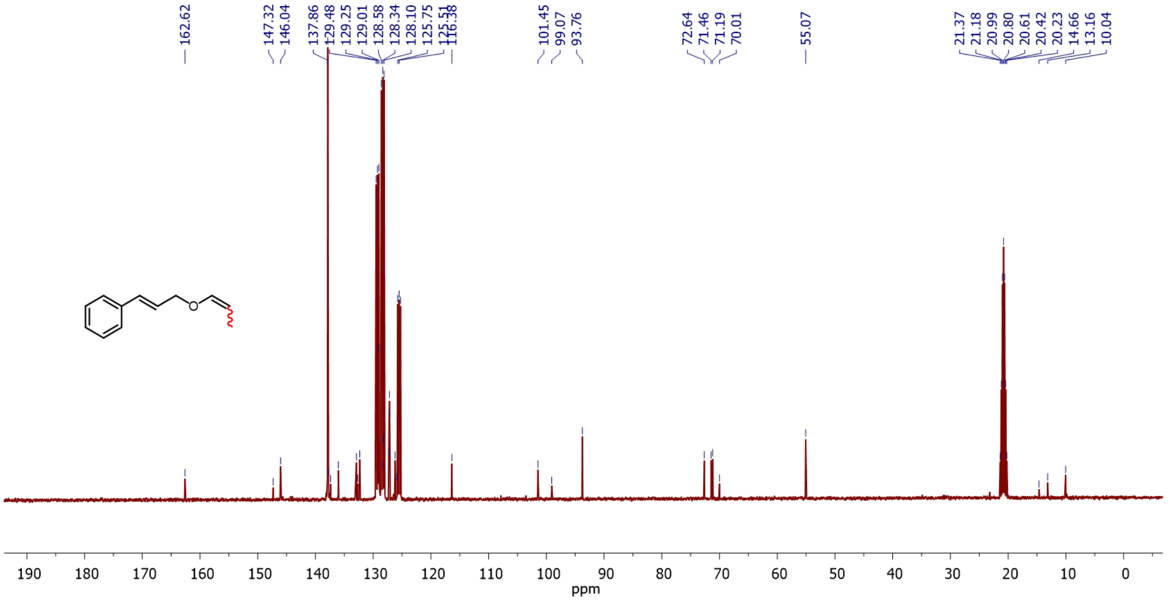

Figure S58. ^{13}C {^{1}H} NMR spectrum (101 MHz) of **5l** in toluene-d_8 (method B).

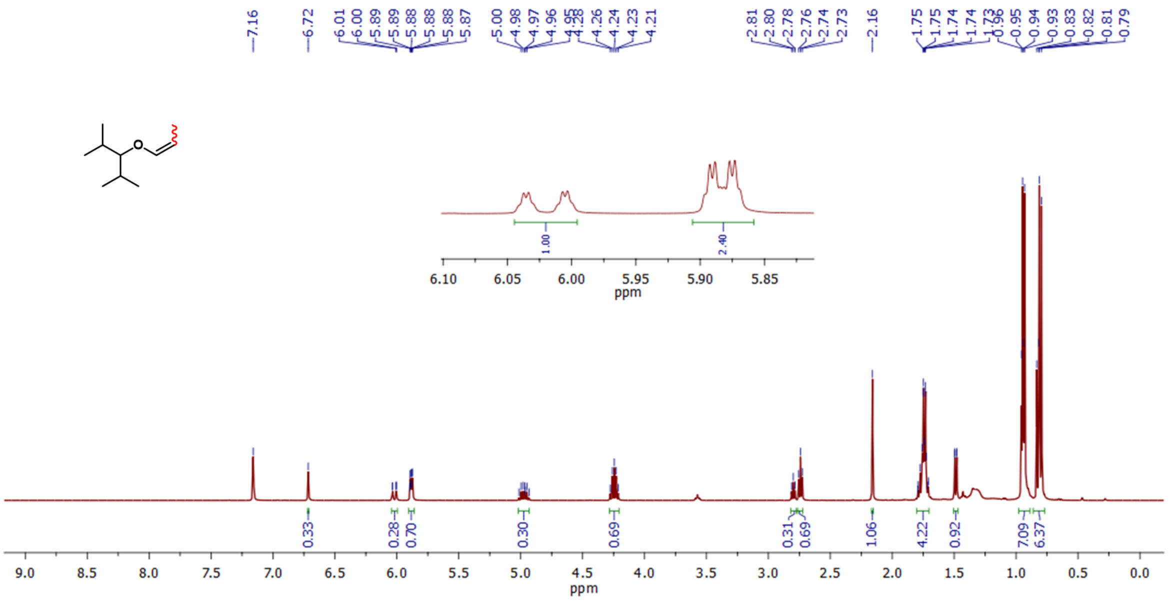

Figure S59. ^{1}H NMR spectrum (400 MHz) of **5m** in benzene-d_6 (method A).

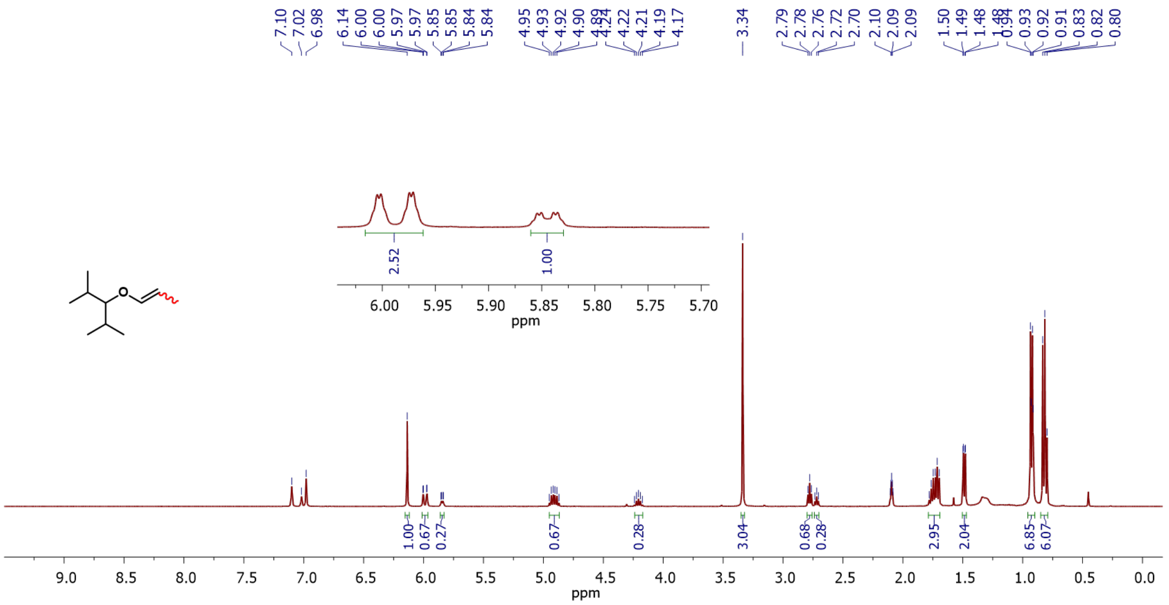

Figure S60. ^{1}H NMR spectrum (400 MHz) of **5m** in toluene-d_8 (method B).

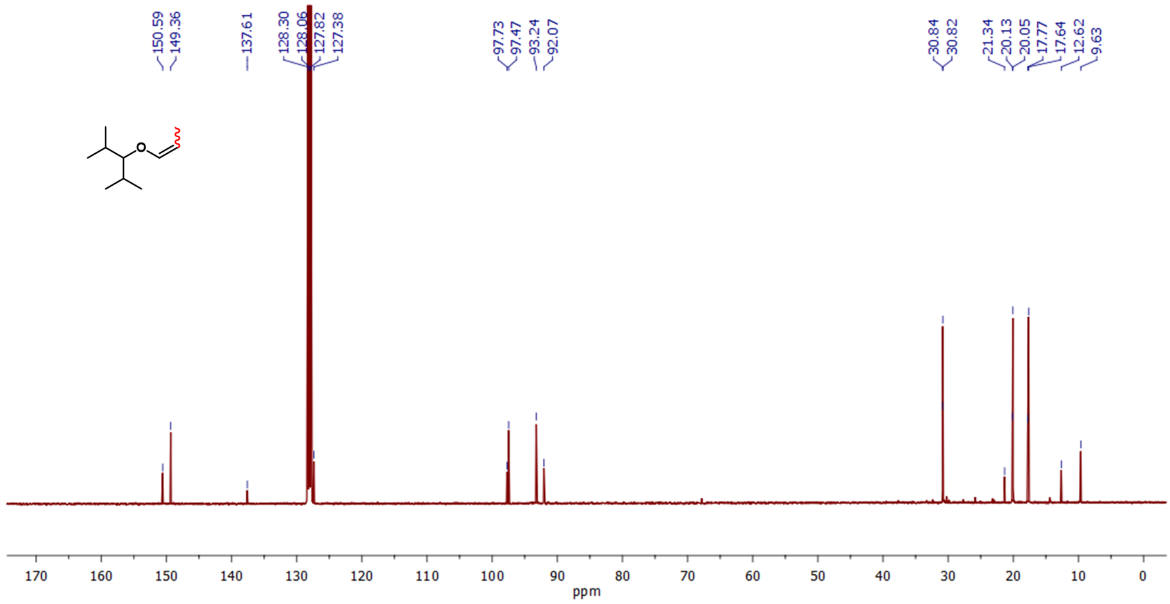

Figure S61. ^{13}C {^{1}H} NMR spectrum (101 MHz) of **5m** in benzene-d_6 (method A).

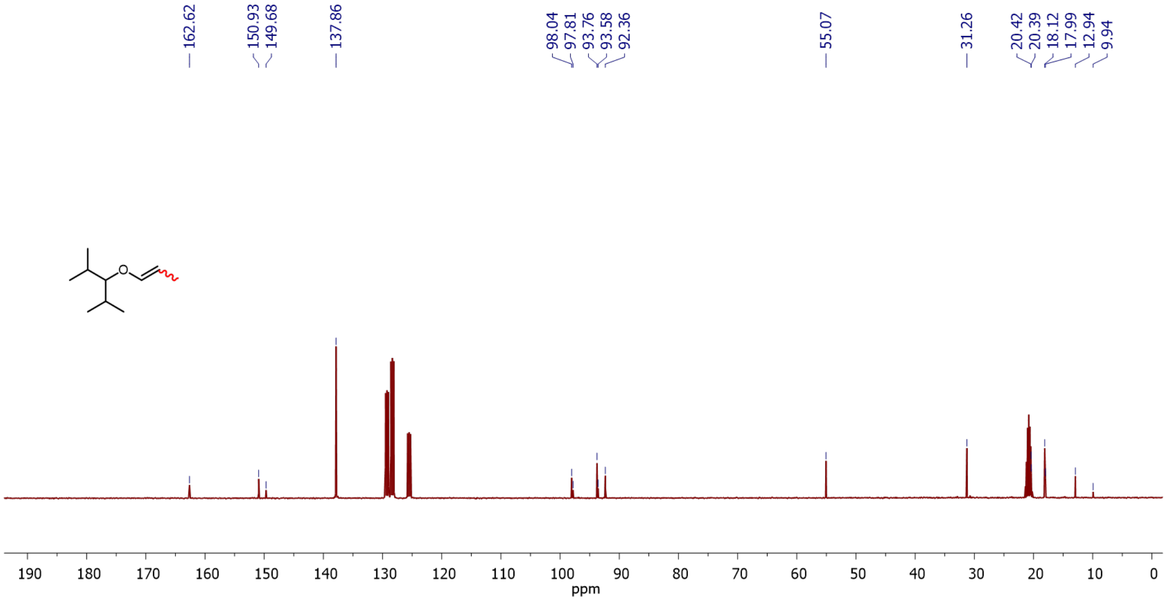

Figure S62. ^{13}C {^{1}H} NMR spectrum (101 MHz) of **5m** in toluene-d_8 (method B).

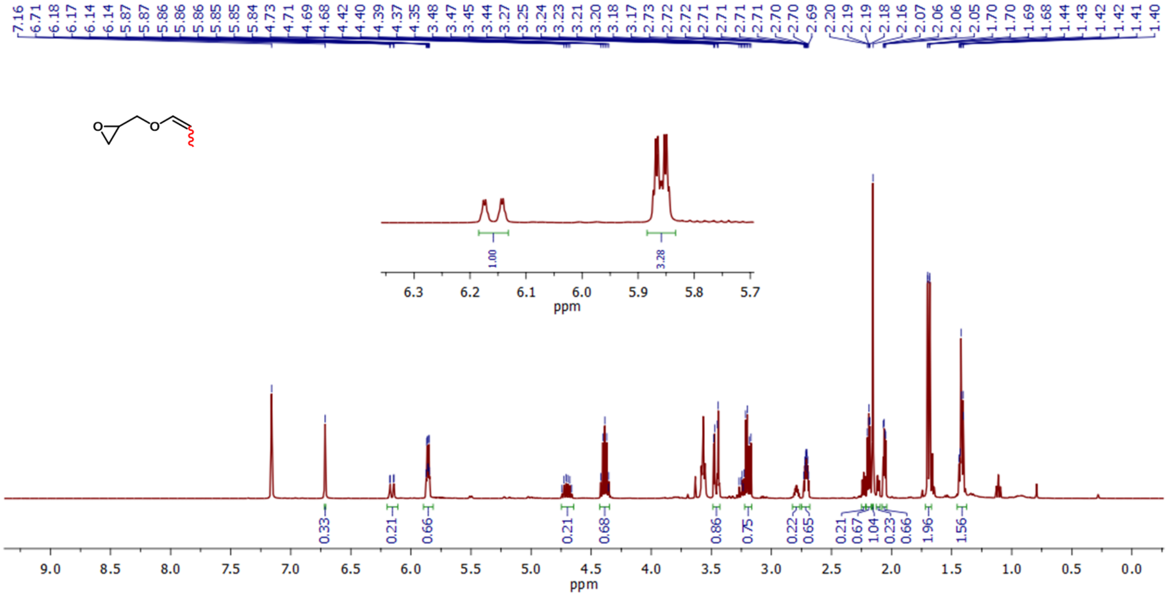

Figure S63. ^{1}H NMR spectrum (400 MHz) of **5n** in benzene-d_6 (method A).

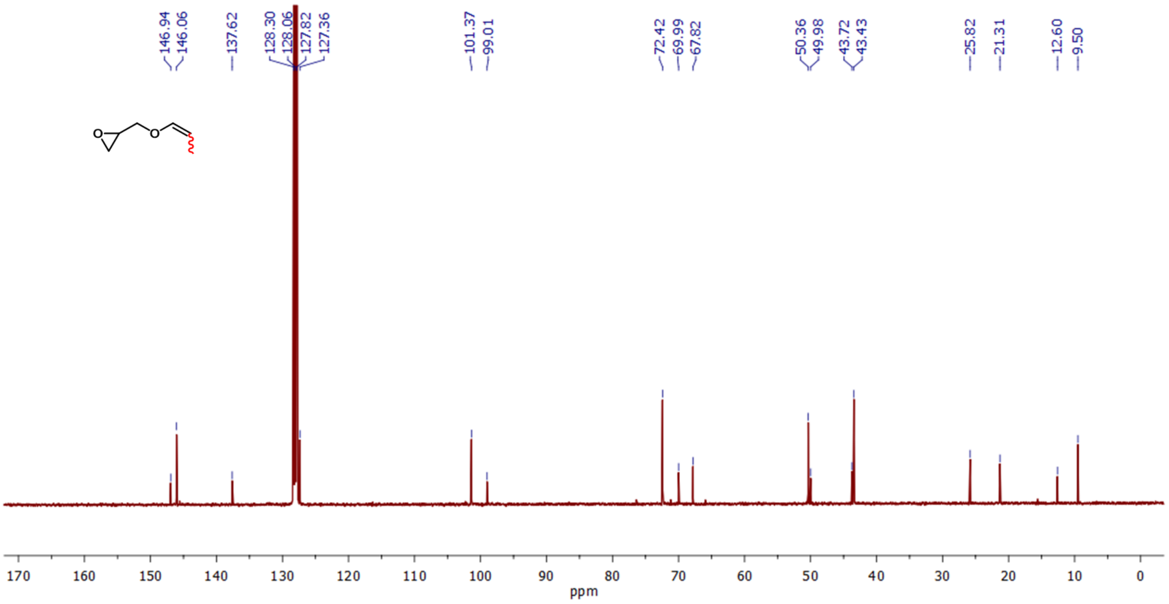

Figure S64. ^{13}C {^{1}H} NMR spectrum (101 MHz) of **5n** in benzene-d_6 (method A).

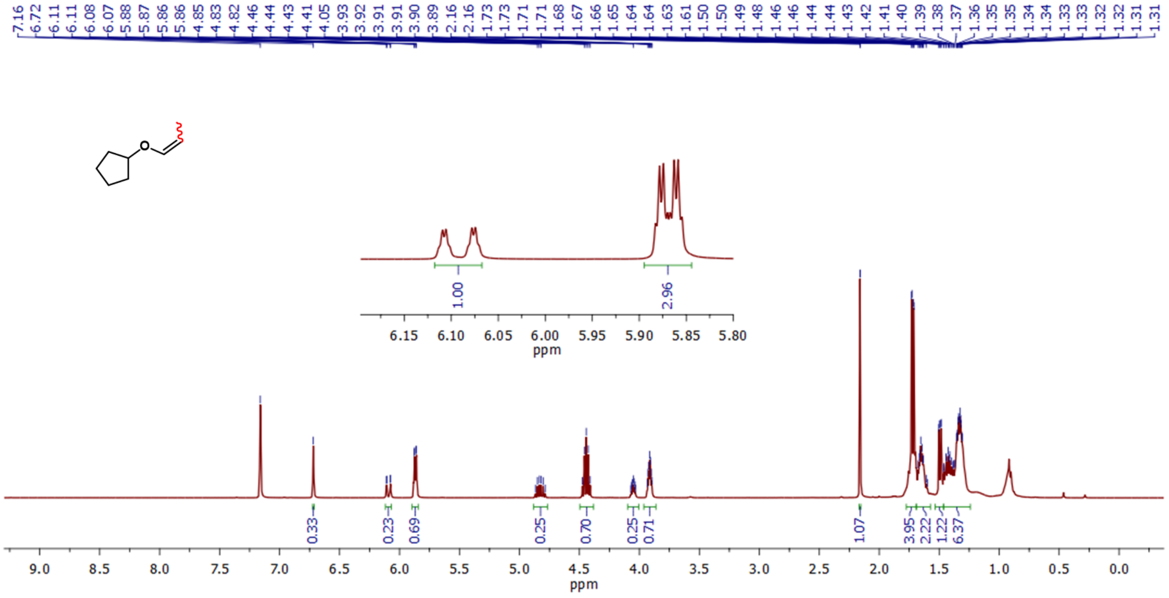

Figure S65. ^{1}H NMR spectrum (400 MHz) of **5o** in benzene-d_6 (method A).

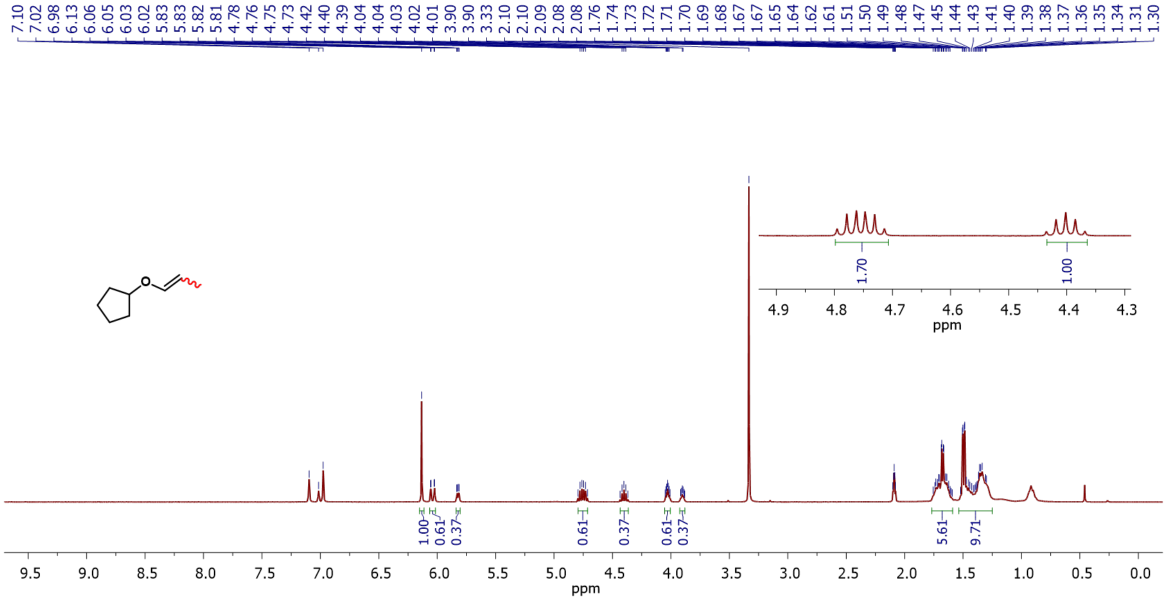

Figure S66. ^{1}H NMR spectrum (400 MHz) of **5o** in toluene-d_8 (method B).

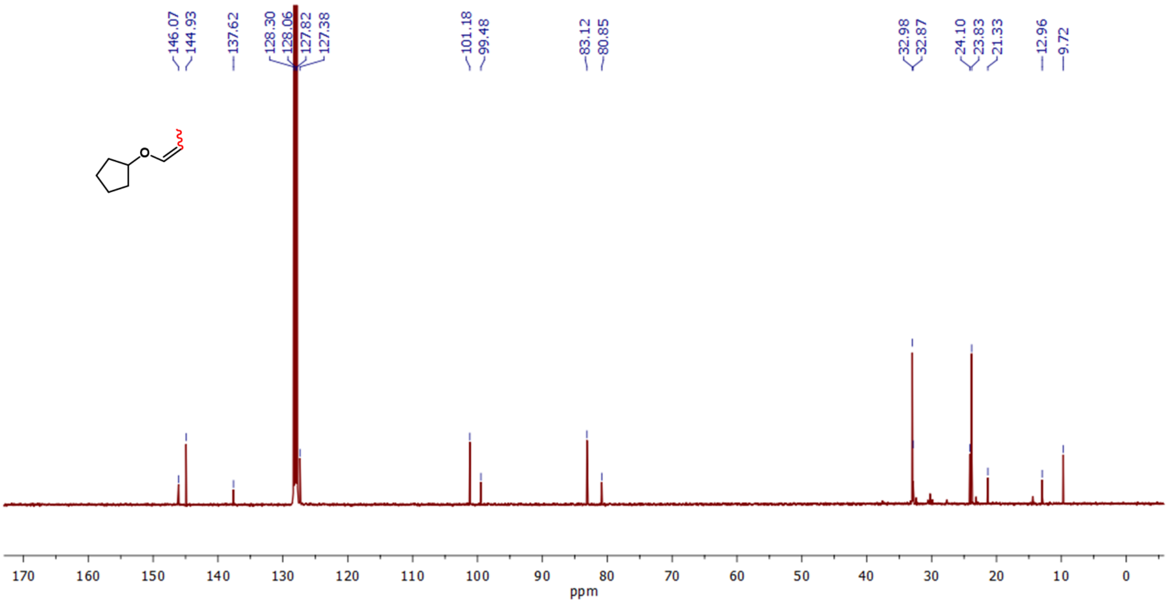

Figure S67. ^{13}C {^{1}H} NMR spectrum (101 MHz) of **5o** in benzene-d_6 (method A).

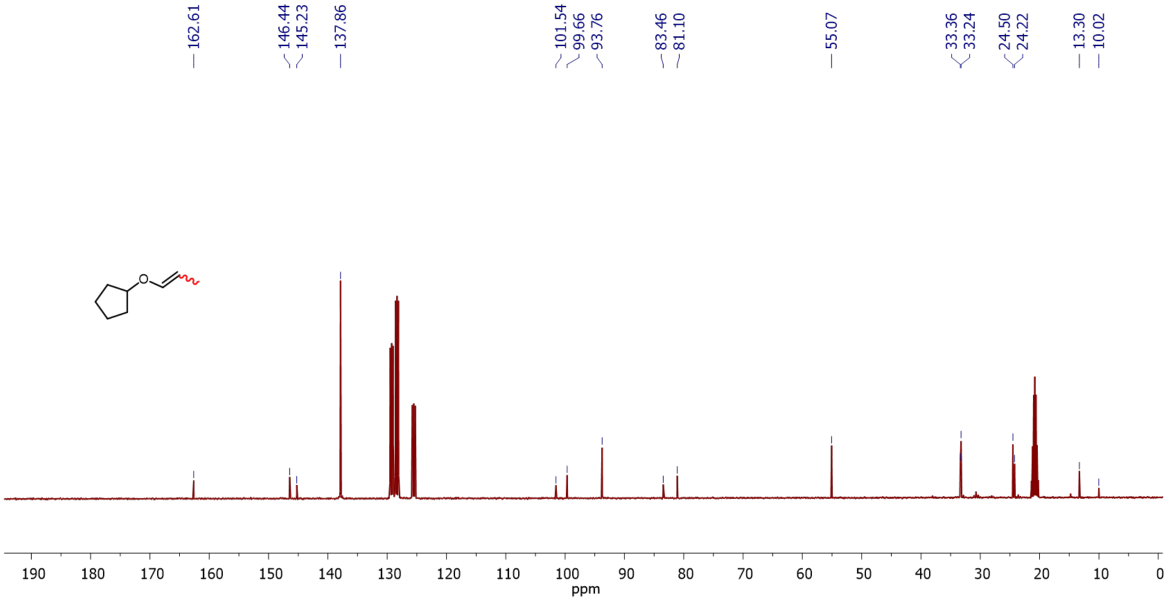

Figure S68. ^{13}C {^{1}H} NMR spectrum (101 MHz) of **5o** in toluene-d_8 (method B).

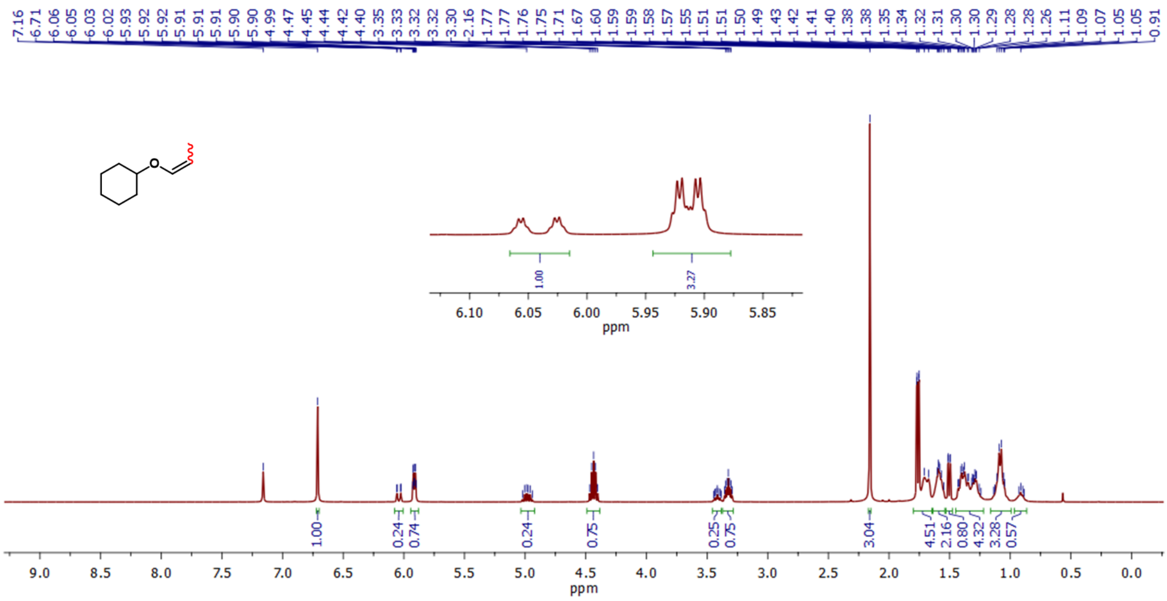

Figure S69. ^{1}H NMR spectrum (400 MHz) of **5p** in benzene-d_6 (method A).

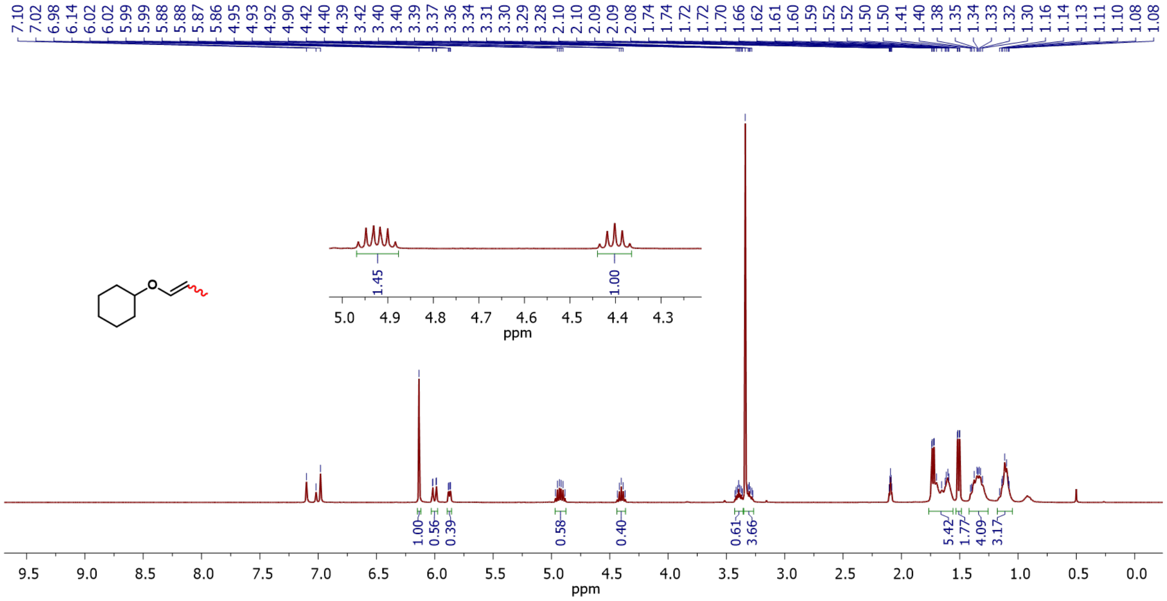

Figure S70. ^{1}H NMR spectrum (400 MHz) of **5p** in toluene-d_8 (method B).

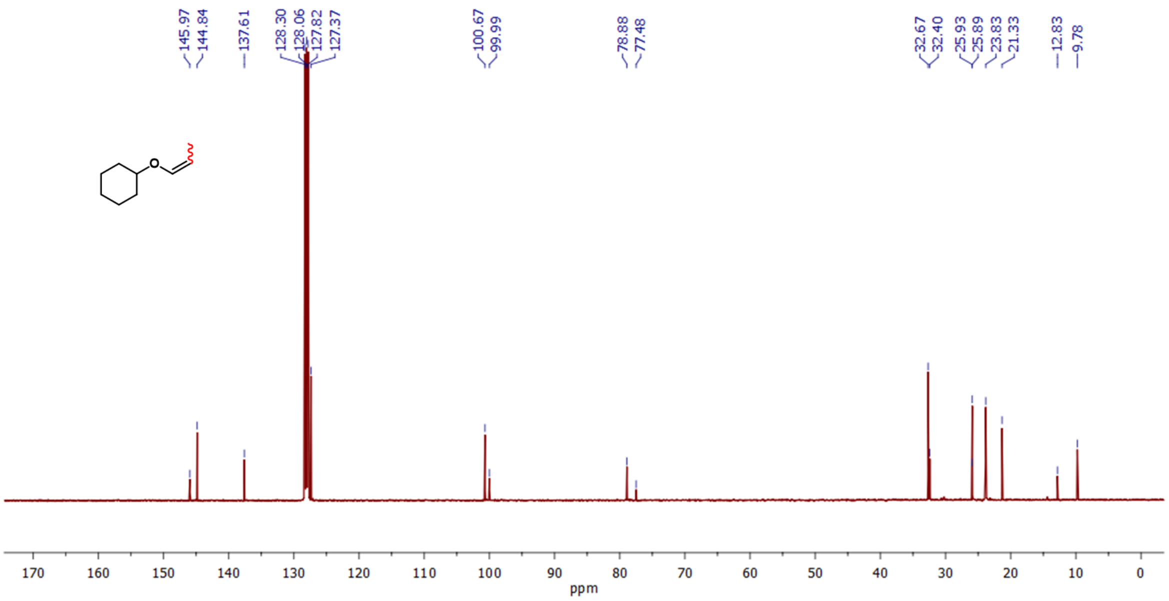

Figure S71. ^{13}C {^1H} NMR spectrum (101 MHz) of **5p** in benzene-d_6 (method A).

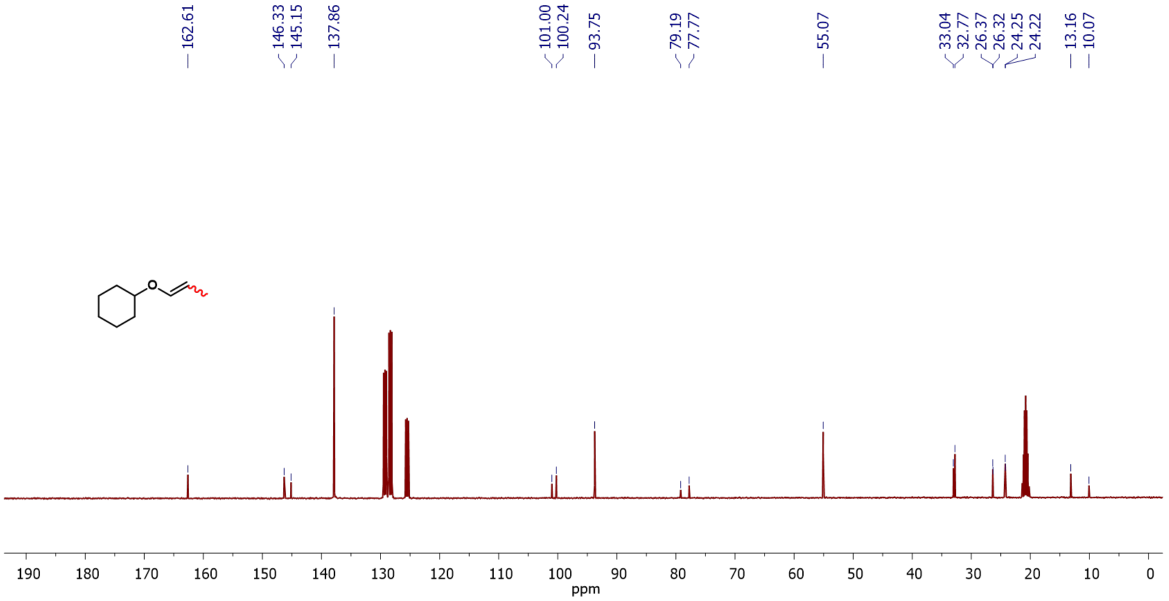

Figure S72. ^{13}C {^1H} NMR spectrum (101 MHz) of **5p** in toluene-d_8 (method B).

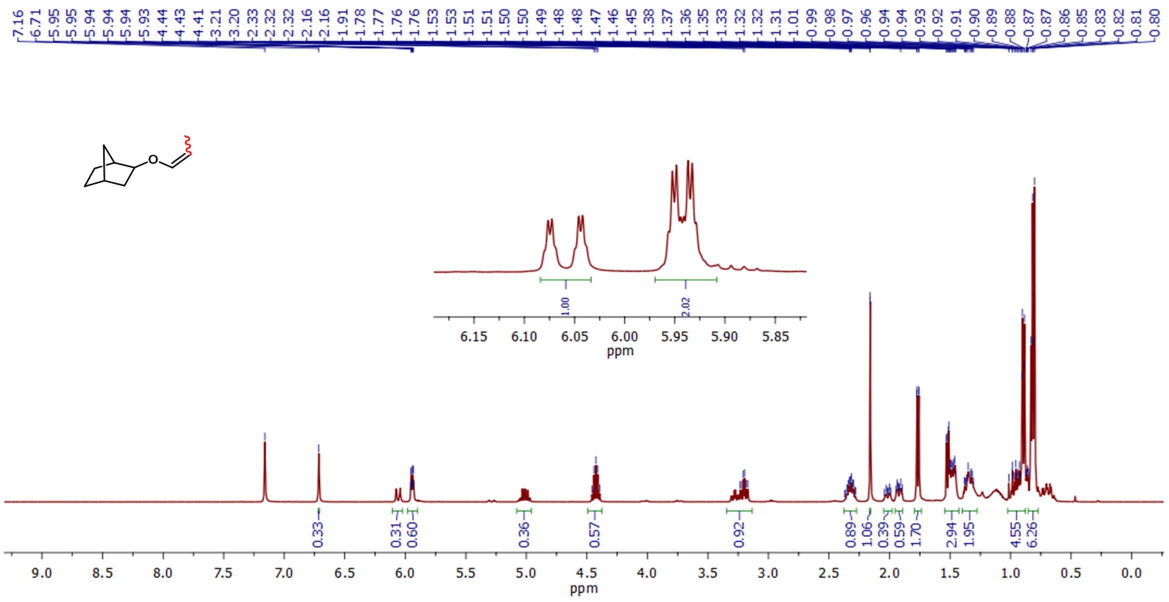

Figure S73. ^{1}H NMR spectrum (400 MHz) of **5q** in benzene-d_6 (method A).

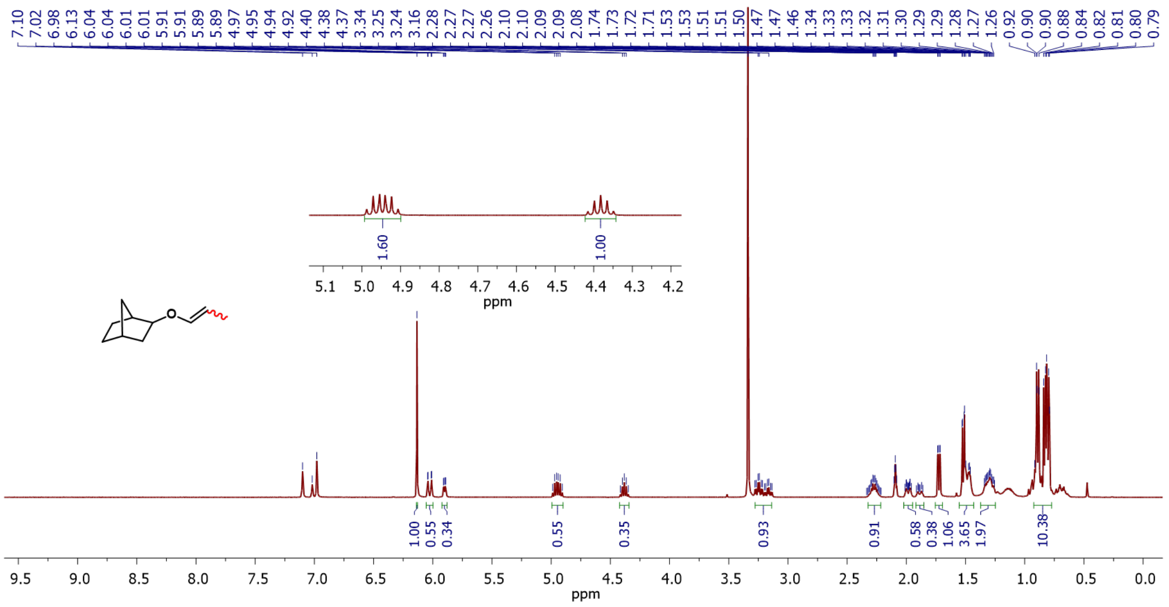

Figure S74. ^{1}H NMR spectrum (400 MHz) of **5q** in toluene-d_8 (method B).

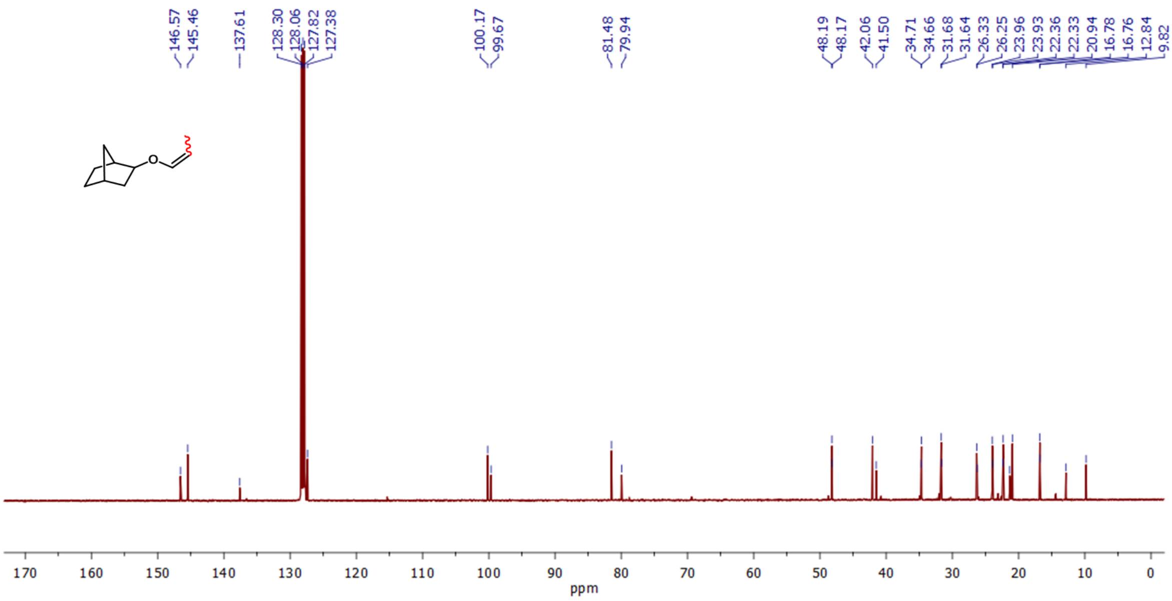

Figure S75. ^{13}C {^{1}H} NMR spectrum (101 MHz) of **5q** in benzene-d_6 (method A).

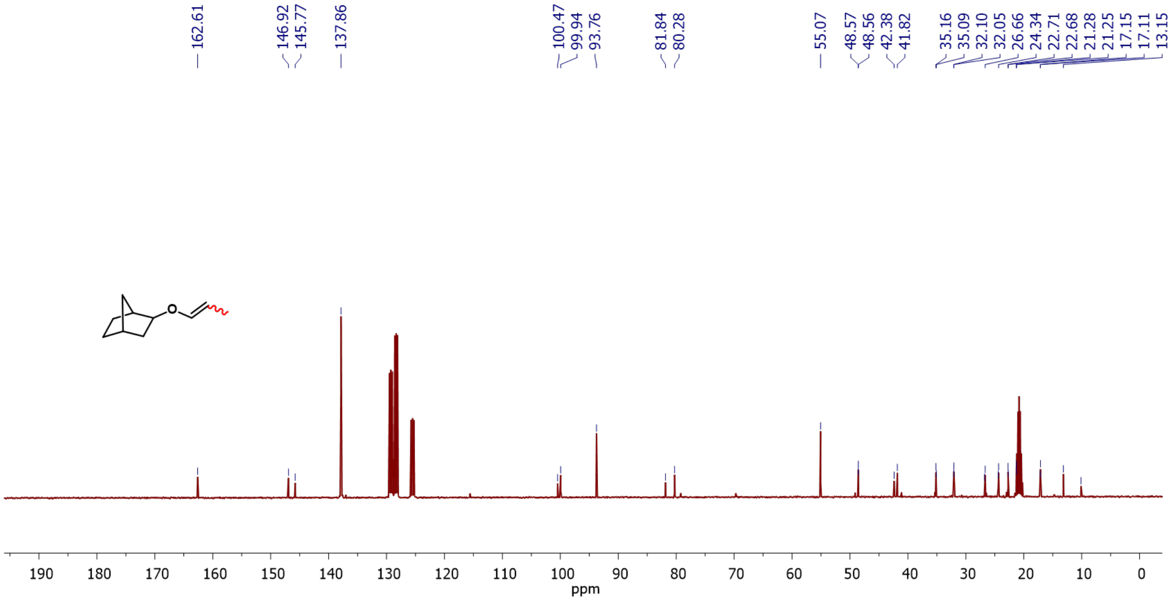

Figure S76. ^{13}C {^{1}H} NMR spectrum (101 MHz) of **5q** in toluene-d_8 (method B).

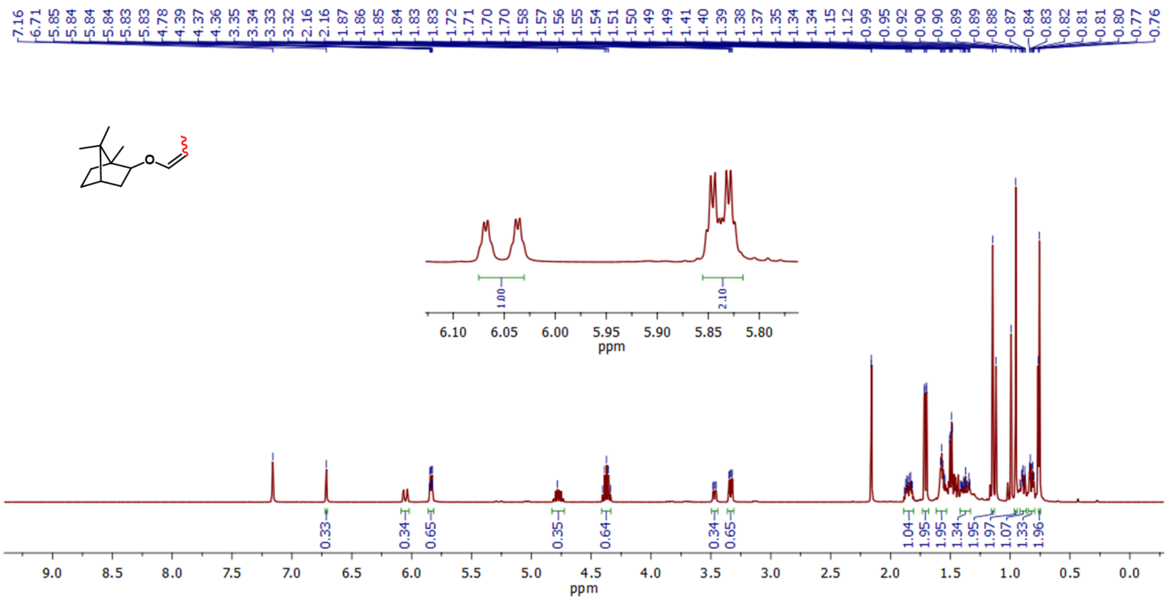

Figure S77. ^{1}H NMR spectrum (400 MHz) of **5r** in benzene-d_6 (method A).

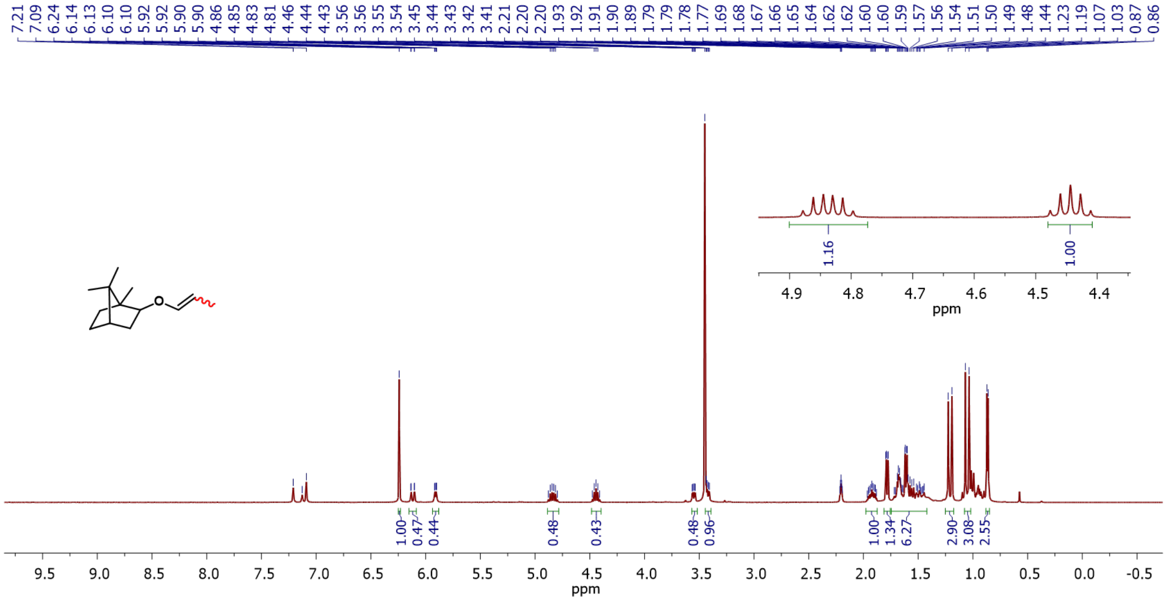

Figure S78. ^{1}H NMR spectrum (400 MHz) of **5r** in toluene-d_8 (method B).

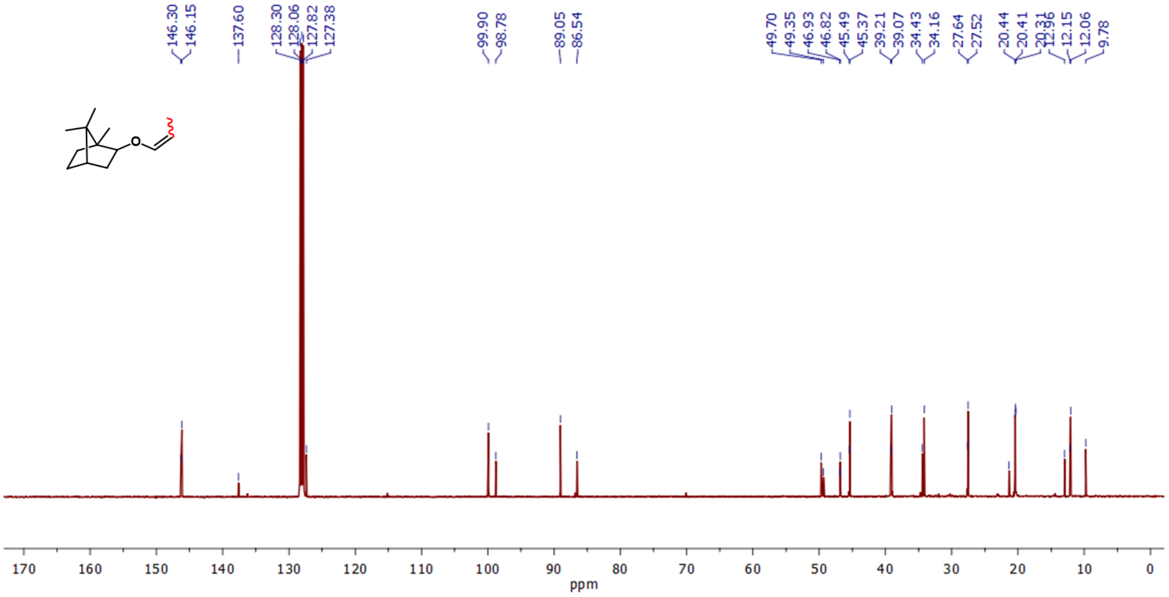

Figure S79. ^{13}C {^{1}H} NMR spectrum (101 MHz) of **5r** in benzene-d_6 (method A).

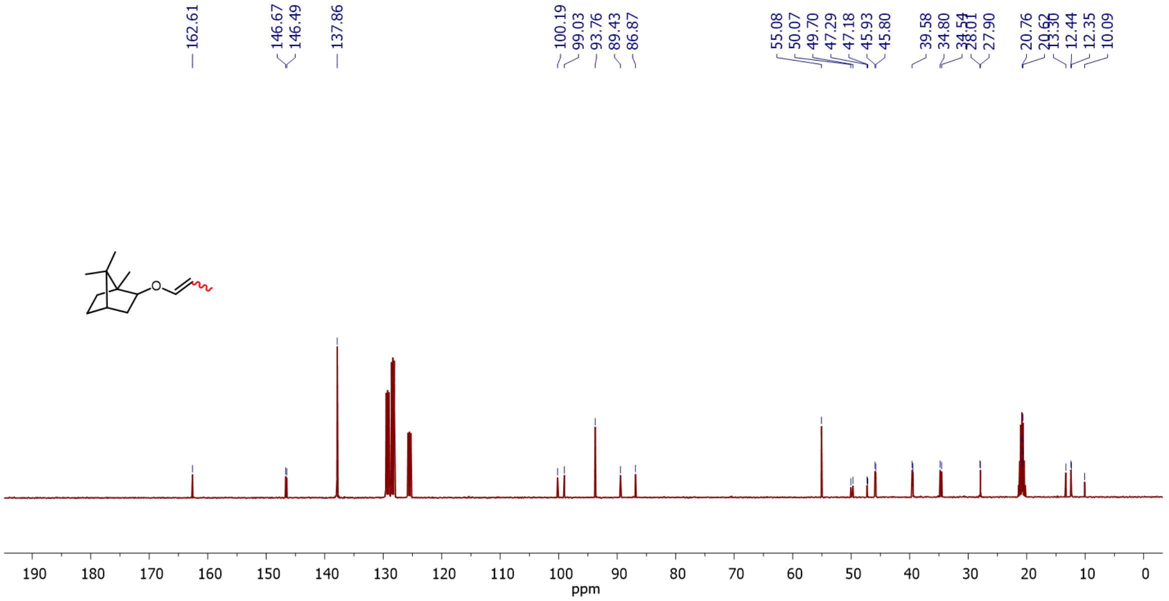

Figure S80. ^{13}C {^{1}H} NMR spectrum (101 MHz) of **5r** in toluene-d_8 (method B).

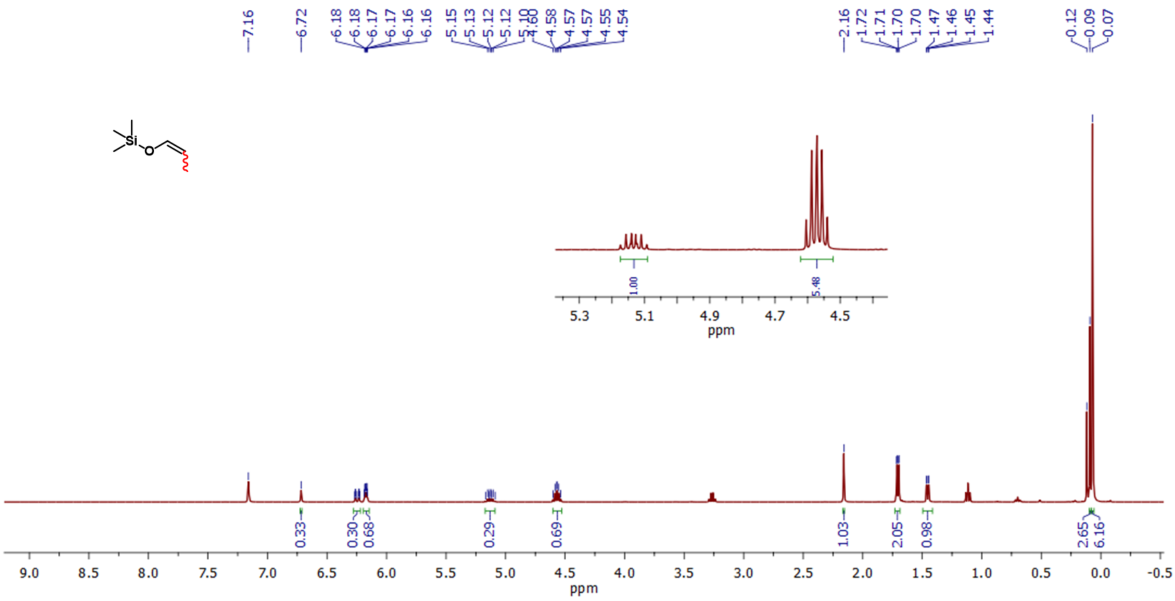

Figure S81. ^{1}H NMR spectrum (400 MHz) of **5s** in benzene-d_6 (method A).

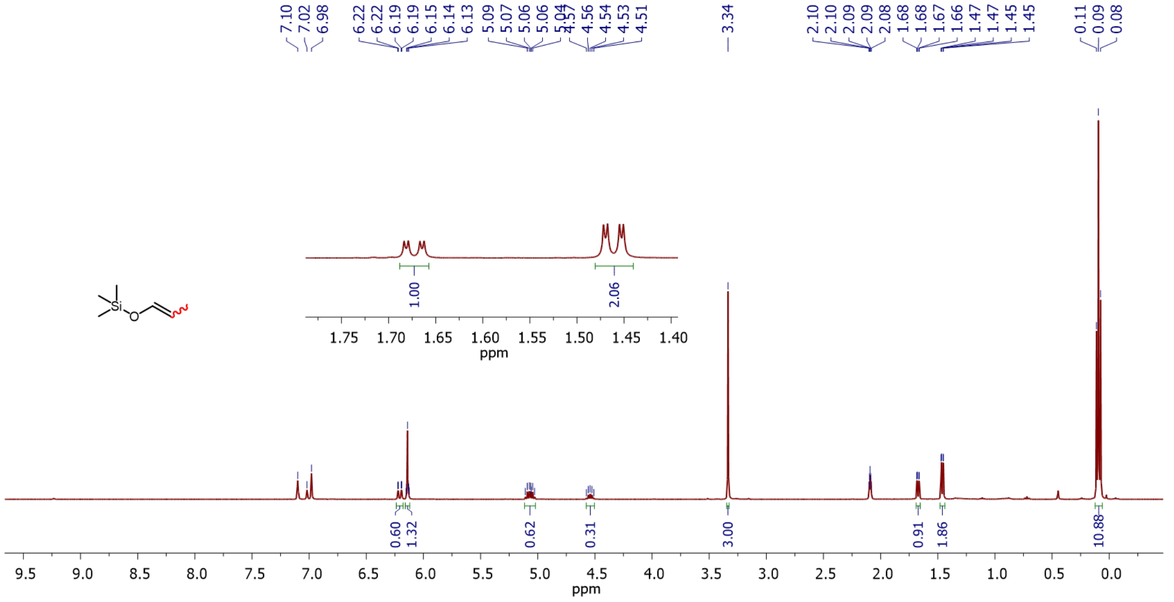

Figure S82. ^{1}H NMR spectrum (400 MHz) of **5s** in toluene-d_8 (method B).

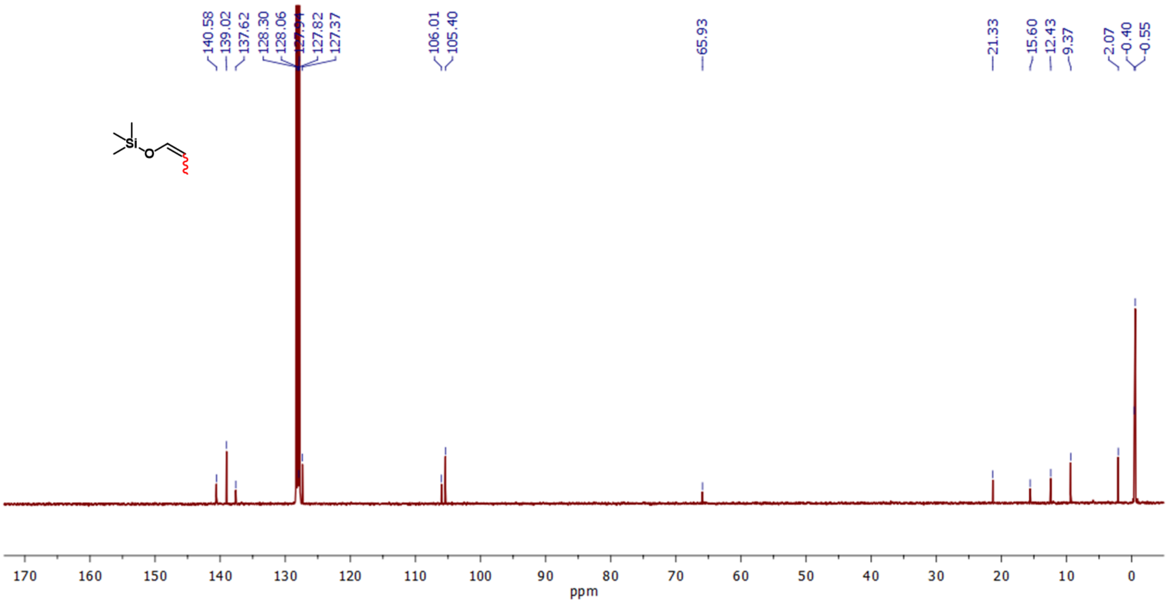

Figure S83. $^{13}C\ \{^{1}H\}$ NMR spectrum (101 MHz) of **5s** in benzene-d_6 (method A).

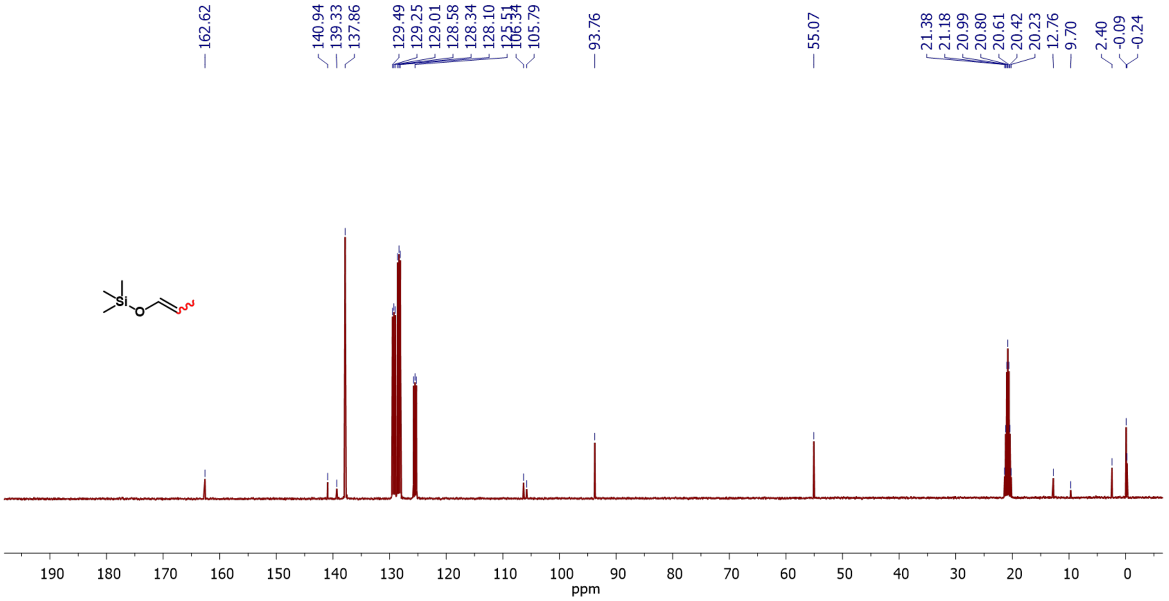

Figure S84. $^{13}C\ \{^{1}H\}$ NMR spectrum (101 MHz) of **5s** in toluene-d_8 (method B).

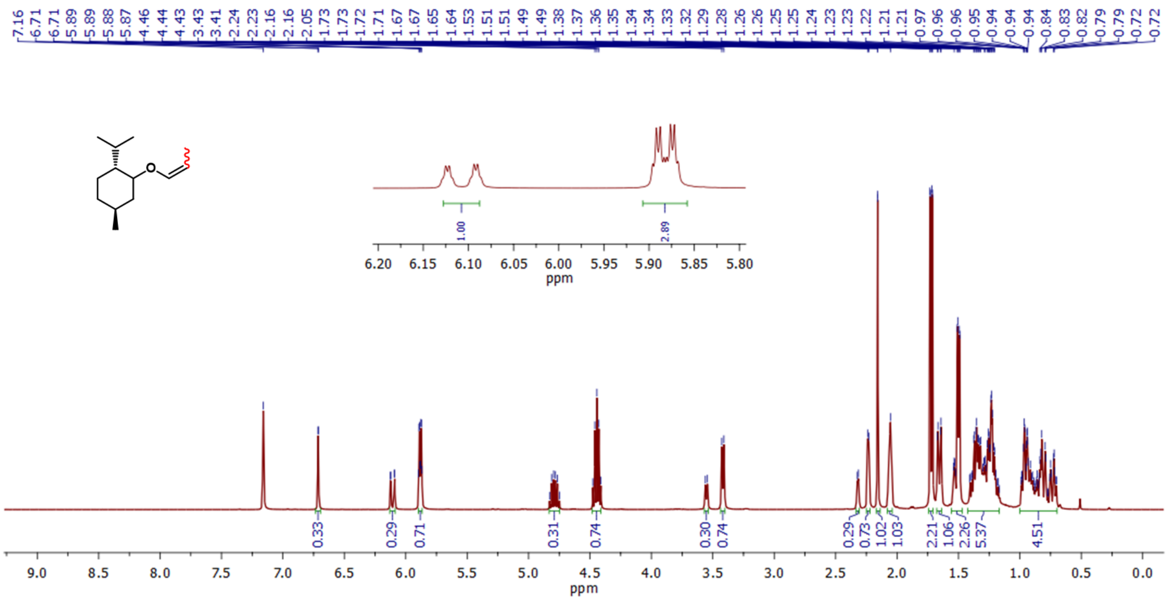

Figure S85. ^{1}H NMR spectrum (400 MHz) of **5t** in benzene-d_6 (method A).

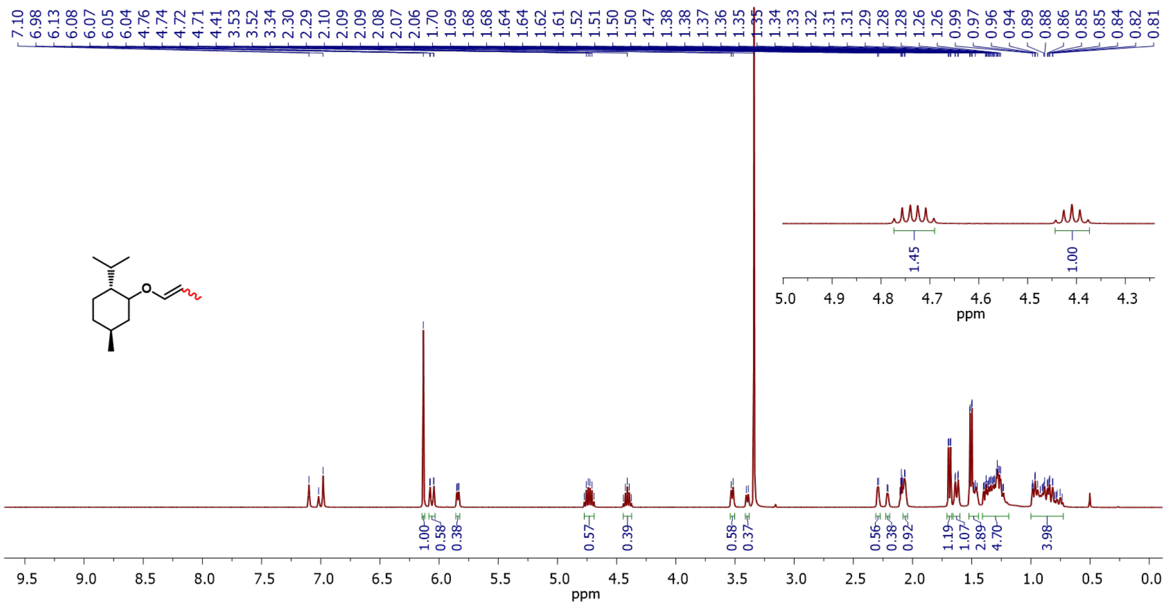

Figure S86. ^{1}H NMR spectrum (400 MHz) of **5t** in toluene-d_8 (method B).

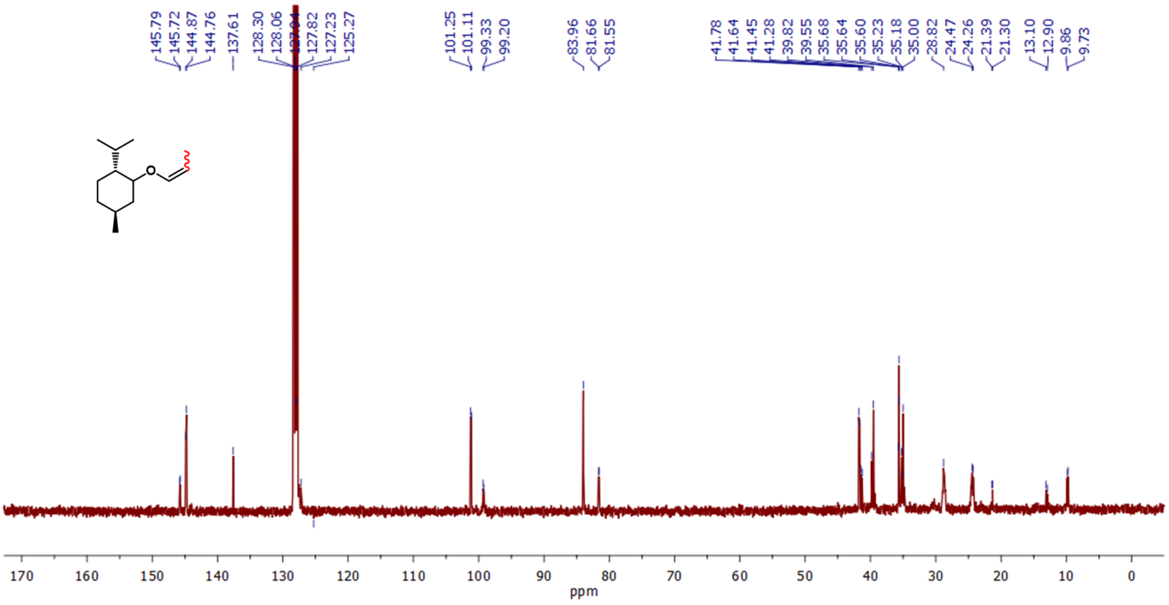

Figure S87. $^{13}C\ \{^{1}H\}$ NMR spectrum (101 MHz) of **5t** in benzene-d_6 (method A).

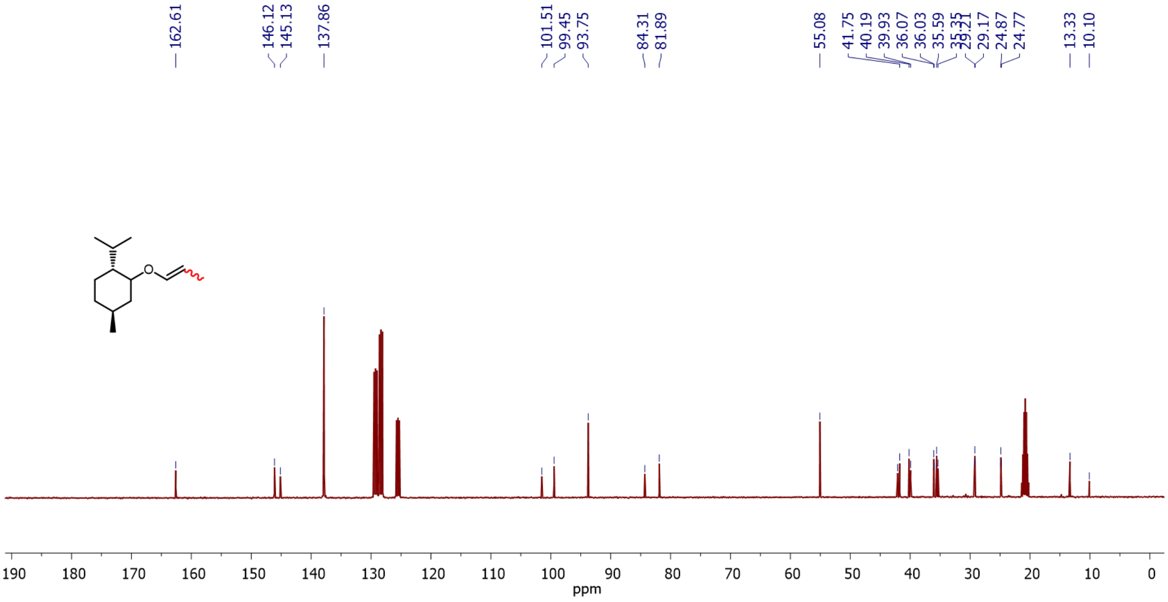

Figure S88. $^{13}C\ \{^{1}H\}$ NMR spectrum (101 MHz) of **5t** in toluene-d_8 (method B).

Allyl benzyl ether isomerization as a function of time.

According to general procedure (A), inside the glovebox, an oven-dried J-Young tube was charged with catalyst **3** dissolved in THF (100 µL, 1 mol%, 0.03 mM stock solution). Next the solvent was evaporated under reduced pressure and allyl benzyl ether (51 mg, 0.3 mmol) was added together with 400 µL of benzene-d_6. The progress of the reaction was monitored by ^{1}H NMR spectroscopy at different time intervals.

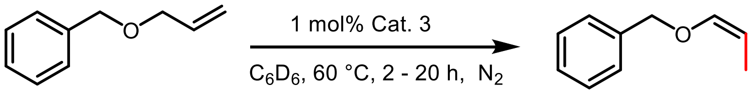

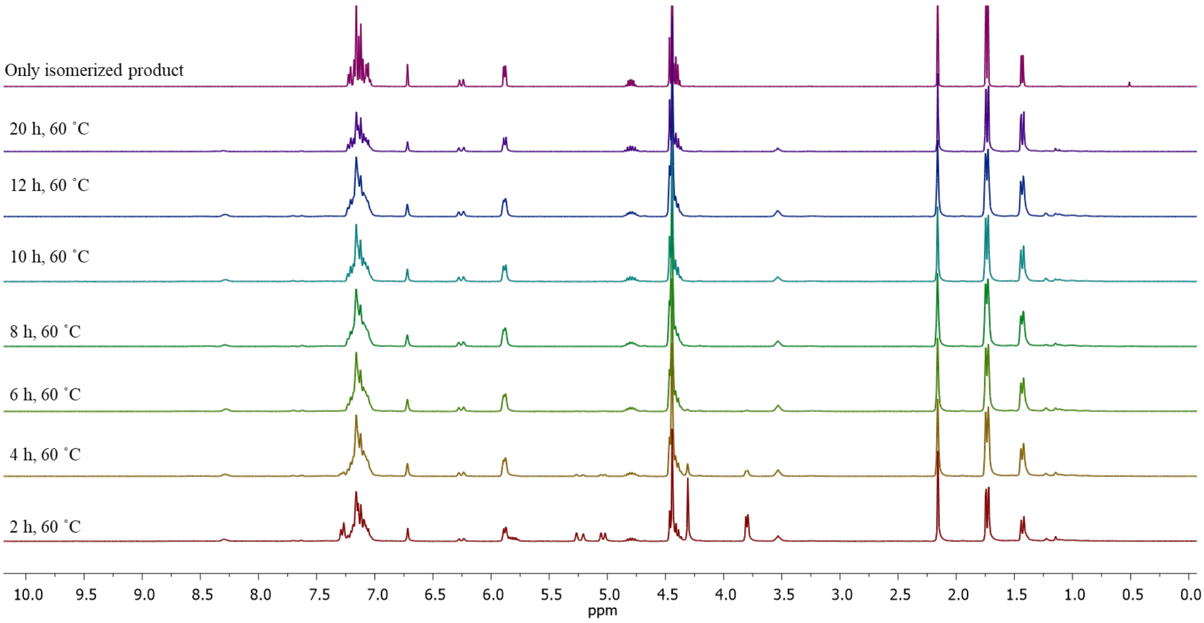

Figure S89. Stacked ^{1}H NMR spectrum (400 MHz, C_6D_6) of allyl benzyl ether isomerization with catalyst **3** at different time intervals.

According to general procedure (B), inside the glovebox, an oven-dried J-Young tube was charged with allyl benzyl ether (26 mg, 0.15 mmol) and catalyst **4** in benzene-d_6 (125 µL, 0.0075 mmol, 5.0 mol% from a 0.06 mM stock solution prepared in benzene-d_6). To the reaction mixture was added an additional amount of benzene-d_6 to make a total volume of 400 µL. The progress of the reaction was monitored by ^{1}H NMR spectroscopy at different time intervals.

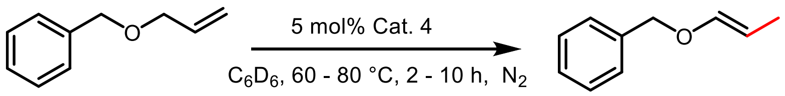

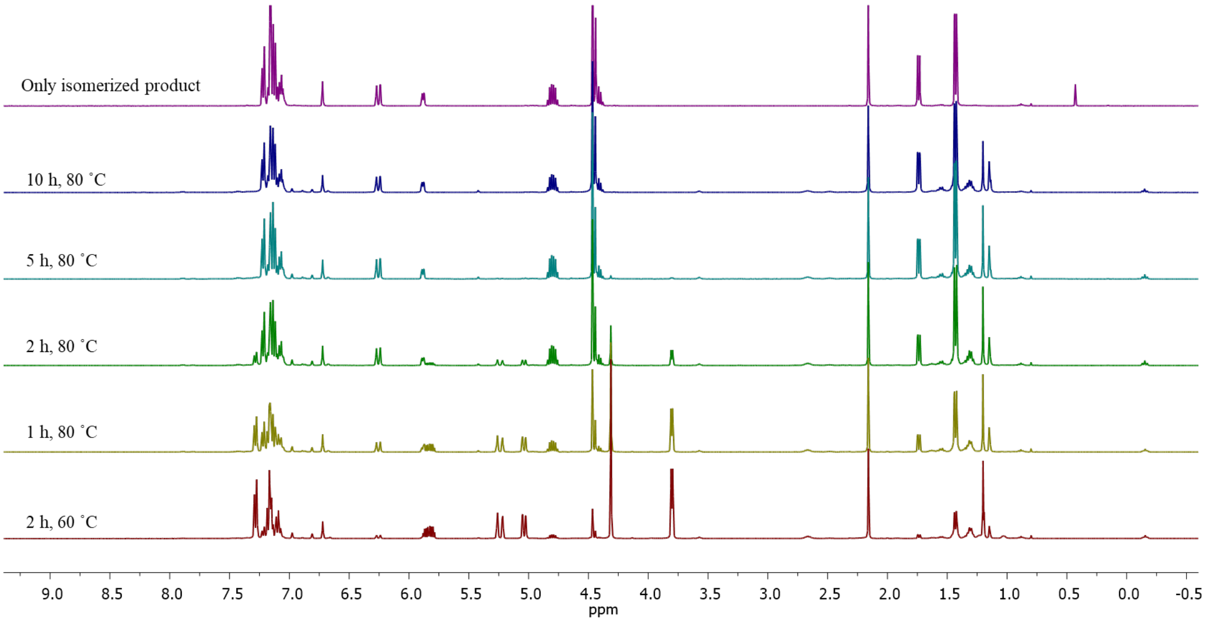

Figure S90. Stacked ^{1}H NMR spectrum (400 MHz, C_6D_6) of allyl benzyl ether isomerization with catalyst **4** at different time intervals.

Deuterium labelling crossover experiment of dec-1-ene-3,3-d$_2$ with 4-allyl-1,2-dimethoxybenzene.

According to general procedure (A), inside the glovebox, an oven-dried J-Young tube was charged with catalyst **3** dissolved in THF (114 µL, 1 mol%, 0.03 mM stock solution). Next the solvent was evaporated under reduced pressure, dec-1-ene-3,3-d$_2$ (24 mg, 0.17 mmol) and 4-allyl-1,2-dimethoxybenzene (30 mg, 0.17 mmol) was added together with 400 µL of benzene-*d*$_6$. The J-Young tube was sealed, taken out of the glovebox, and heated at 60°C for 20h. After the reaction was completed, the J-Young tube was exposed to air and the contents filtered through a short plug of neutral alumina to remove the cobalt catalyst. The alumina was washed with an additional benzene-*d*$_6$ (400 µL*)* to collect all the organic products. The crude ^{1}H NMR spectrum was recorded to determine the product conversion and regioselectivity. Hereafter, the different products were separated by column chromatography (SiO_2; 8.0 cm in Pasteur pipette, benzene followed by dichloromethane) to obtained the decenes and methoxystyrenes. The ^{1}H and ^{2}H spectra were recorded to determine the deuterium scrambling.

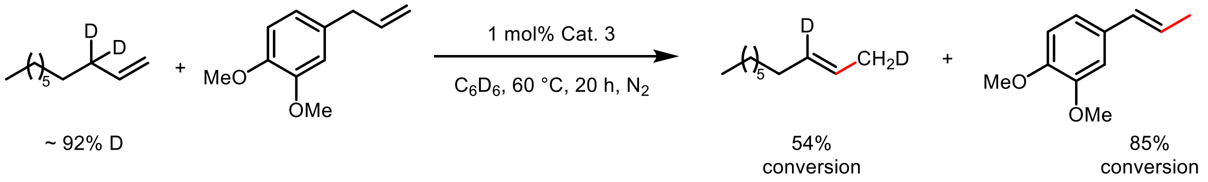

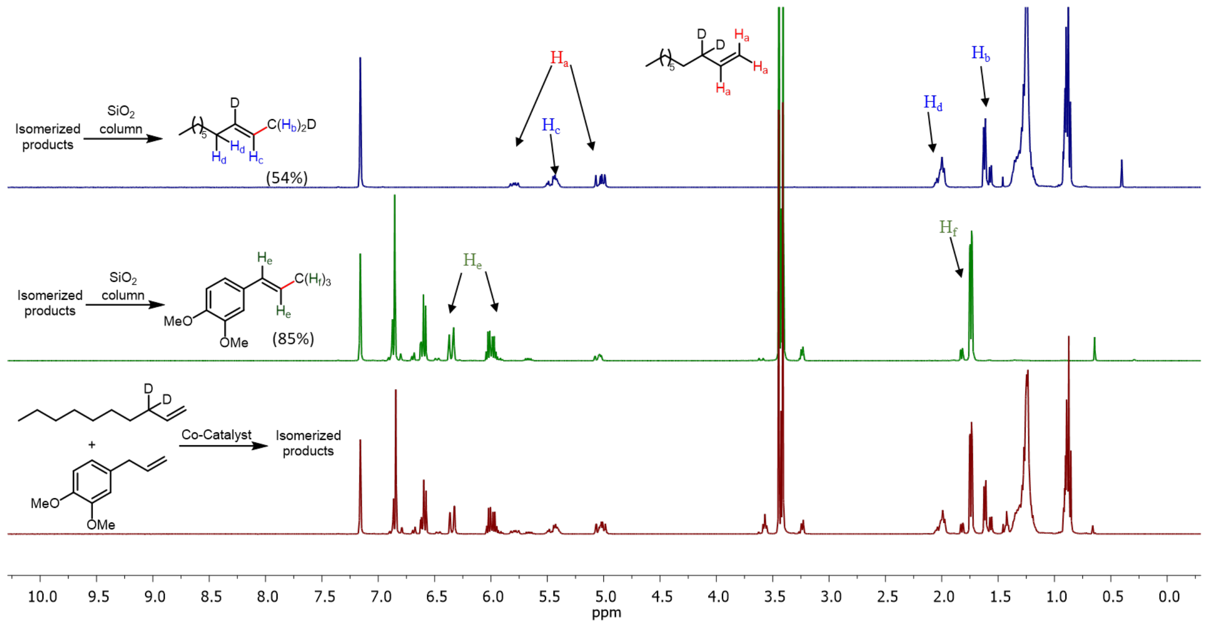

Figure S91. ^{1}H NMR spectrum (400 MHz, C_6D_6) after isomerization of dec-1-ene-3,3-*d*$_2$ and 4-allyl-1,2-dimethoxybenzene with catalyst **3**.

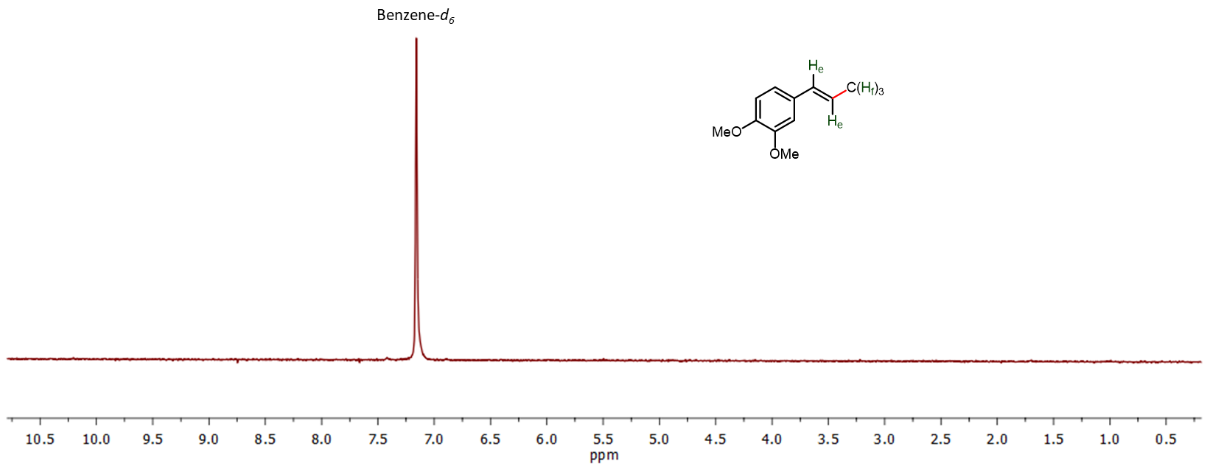

Figure S92. ^{2}H NMR spectrum of isolated product of isomerized 4-allyl-1,2-dimethoxybenzene from the crossover experiment.

According to general procedure (B), inside the glovebox, an oven-dried J-Young tube was charged with dec-1-ene-3,3-d_2 (24 mg, 0.17 mmol), 4-allyl-1,2-dimethoxybenzene (30 mg, 0.17 mmol) and catalyst **4** in benzene-d_6 (114 µL, 0.0068 mmol, 2.0 mol% from a 0.06 mM stock solution prepared in benzene-d_6). To the reaction mixture was added an additional amount of benzene-d_6 to make total volume 400 µL. The J-Young tube was sealed, taken out of the glovebox, and heated at 60°C for 18h. After the reaction was completed, the J-Young tube was exposed to air and the contents filtered through a short plug of neutral alumina to remove the cobalt catalyst. The alumina was washed with an additional benzene-d_6 (400 µL) to collect all the organic products. The crude ^{1}H NMR spectrum was recorded to determine the product conversion and regioselectivity. Hereafter, the different products were separated by column chromatography (SiO_2; 8.0 cm in Pasteur pipette, benzene followed by dichloromethane) to obtained the decenes and methoxystyrenes. The ^{1}H and ^{2}H spectra were recorded to determine the deuterium scrambling.

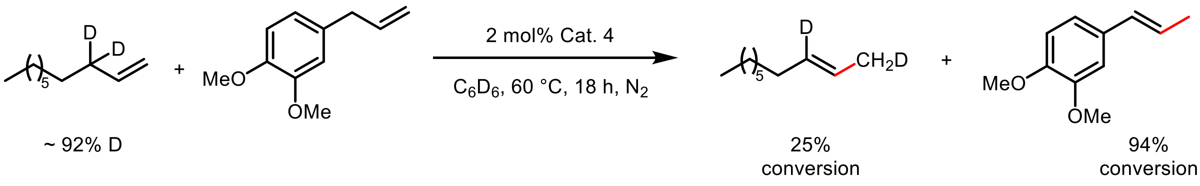

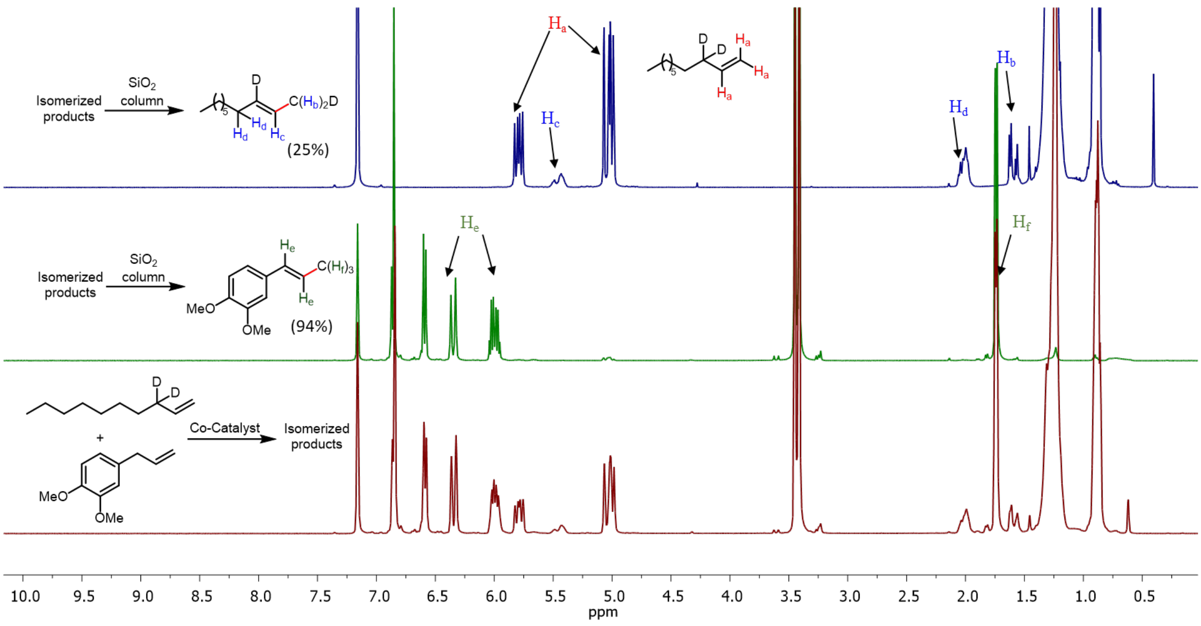

Figure S93. ^{1}H NMR spectrum (400 MHz, C_6D_6) after isomerization of dec-1-ene-3,3-d_2 and 4-allyl-1,2-dimethoxybenzene with catalyst **4**.

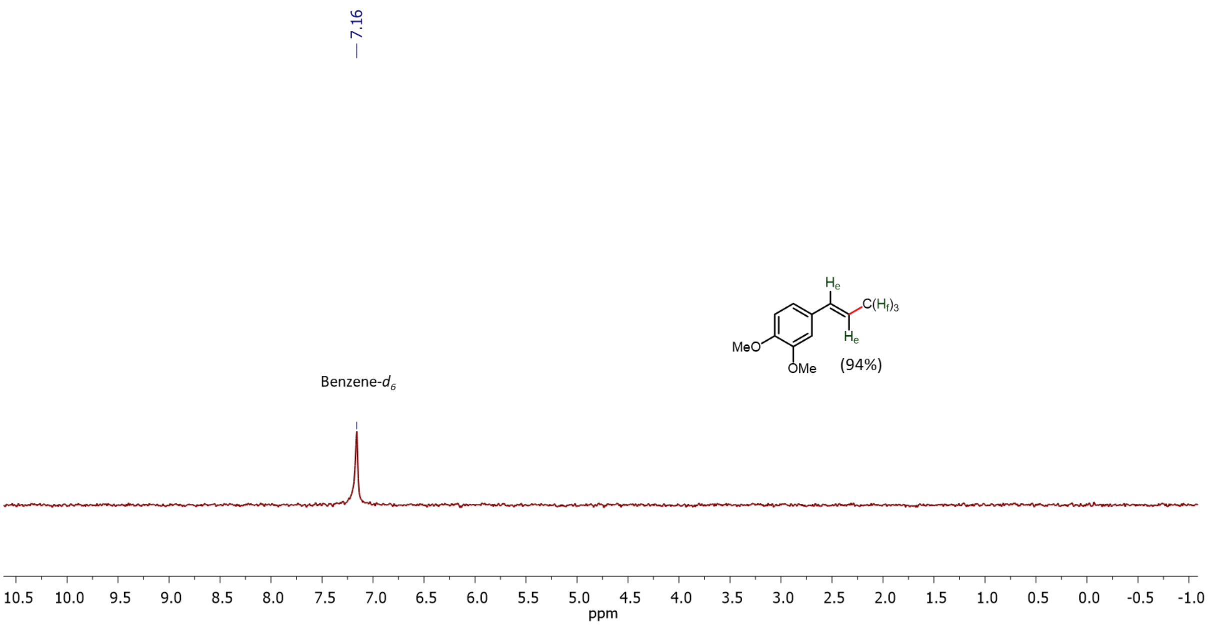

Figure S94. ^{2}H NMR spectrum of isolated product of isomerized 4-allyl-1,2-dimethoxybenzene from the crossover experiment.

Deuterium labelling experiment with dodec-1-ene-1,1-d_2.

According to general procedure (A), inside the glovebox, an oven-dried J-Young tube was charged with catalyst **3** dissolved in THF (100 µL, 1 mol%, 0.03 mM stock solution). Next the solvent was evaporated under reduced pressure and dodec-1-ene-1,1-d_2 (51 mg, 0.3 mmol) was added together with 400 µL of benzene-d_6. The J-Young tube was sealed, taken out of the glovebox, and heated at 60°C for 20h. After the reaction was completed, the J-Young tube was exposed to air and the contents filtered through a short plug of neutral alumina to remove the cobalt catalyst. The alumina was washed with an additional benzene-d_6 (400 µL) to collect all the organic products. The ^{1}H and ^{2}H spectra were recorded to determine the deuterium scrambling.

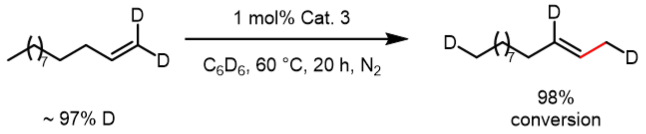

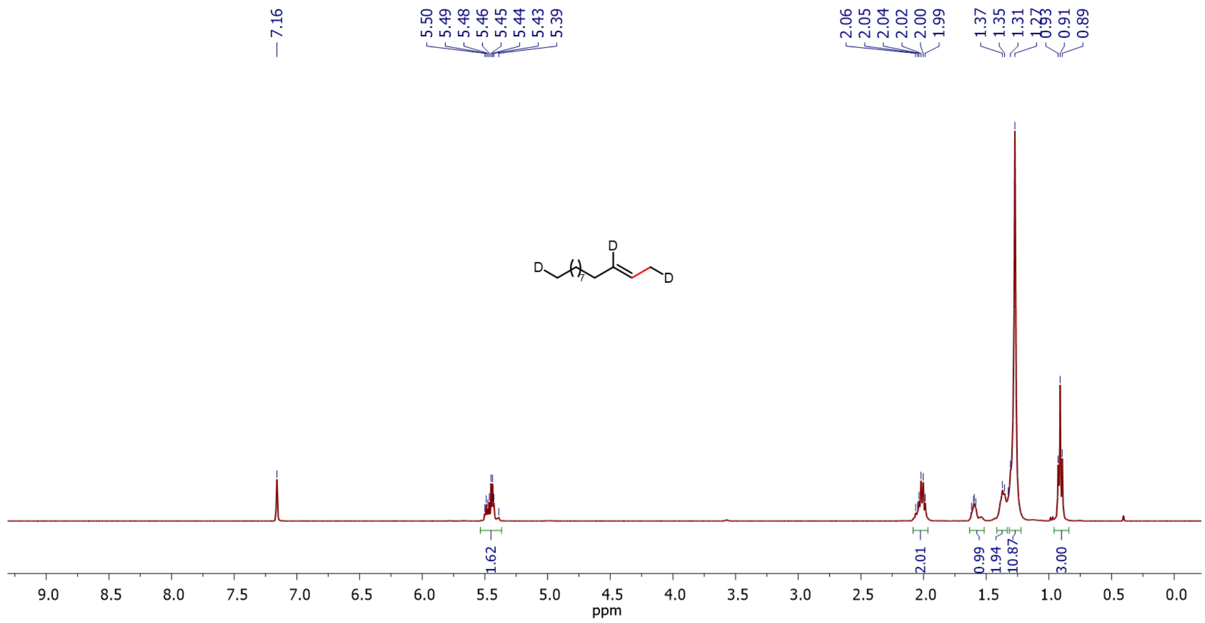

Figure S95. ^{1}H NMR spectrum (400 MHz, C_6D_6) after isomerization of dodec-1-ene-1,1-d_2 with catalyst **3**.

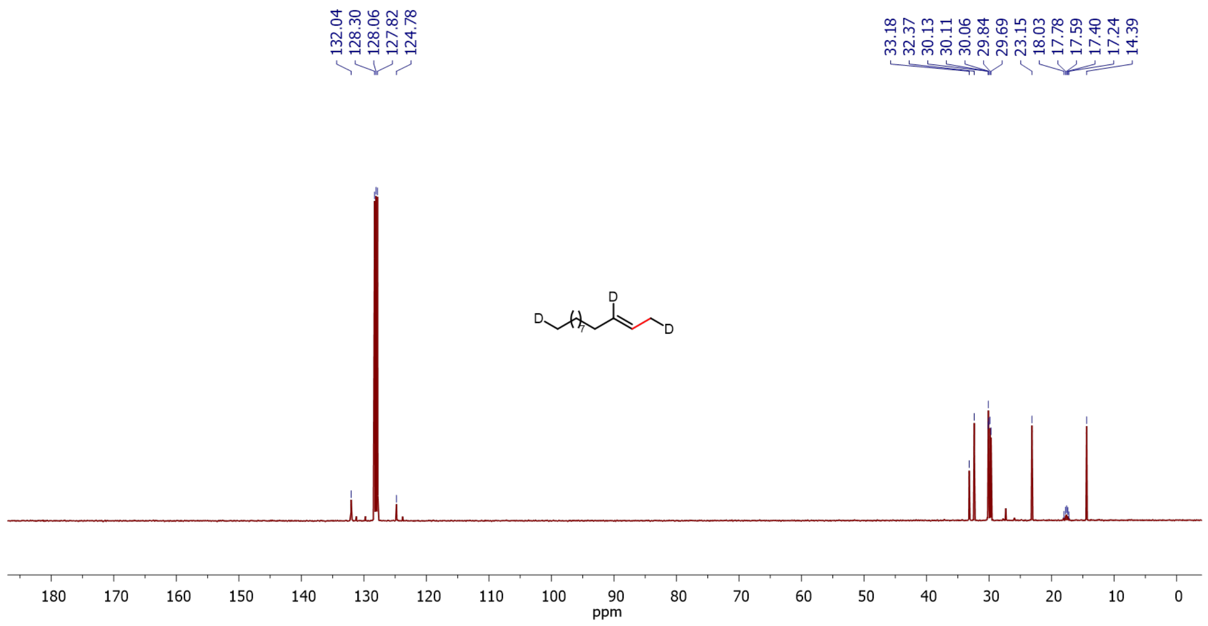

Figure S96. ^{13}C {^{1}H} NMR spectrum (101 MHz, C_6D_6) after isomerization of dodec-1-ene-1,1-d_2 with catalyst **3.**

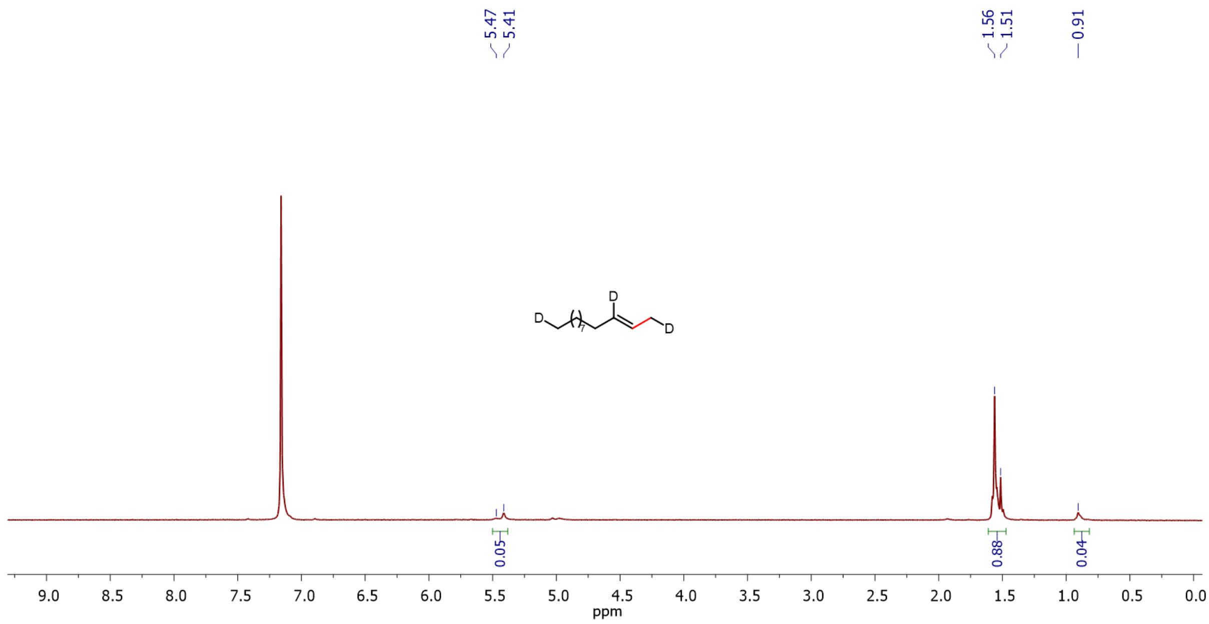

Figure S97. ^{2}H NMR spectrum (46.07 MHz) after isomerization of dodec-1-ene-1,1-d_2 with catalyst **3** in benzene.

According to general procedure (B), inside the glovebox, an oven-dried J-Young tube was charged with dodec-1-ene-1,1-d_2 (26 mg, 0.15 mmol) and catalyst **4** in benzene-d_6 (50 µL, 0.003 mmol, 2.0 mol% from a 0.06 mM stock solution prepared in benzene-d_6). To the reaction mixture was added an additional amount of benzene-d_6 to make total volume 400 µL. The J-Young tube was sealed, taken out of the glovebox, and heated at 60˚C for 18h. After the reaction was completed, the J-Young tube was exposed to air and the contents filtered through a short plug of neutral alumina to remove the cobalt catalyst. The alumina was washed with an additional benzene-d_6 (400 µL) to collect all the organic products. The ^{1}H and ^{2}H spectra were recorded to determine the deuterium scrambling.

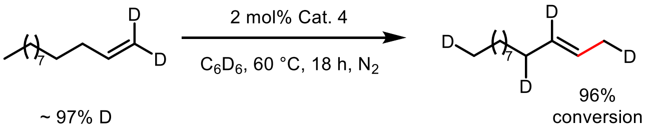

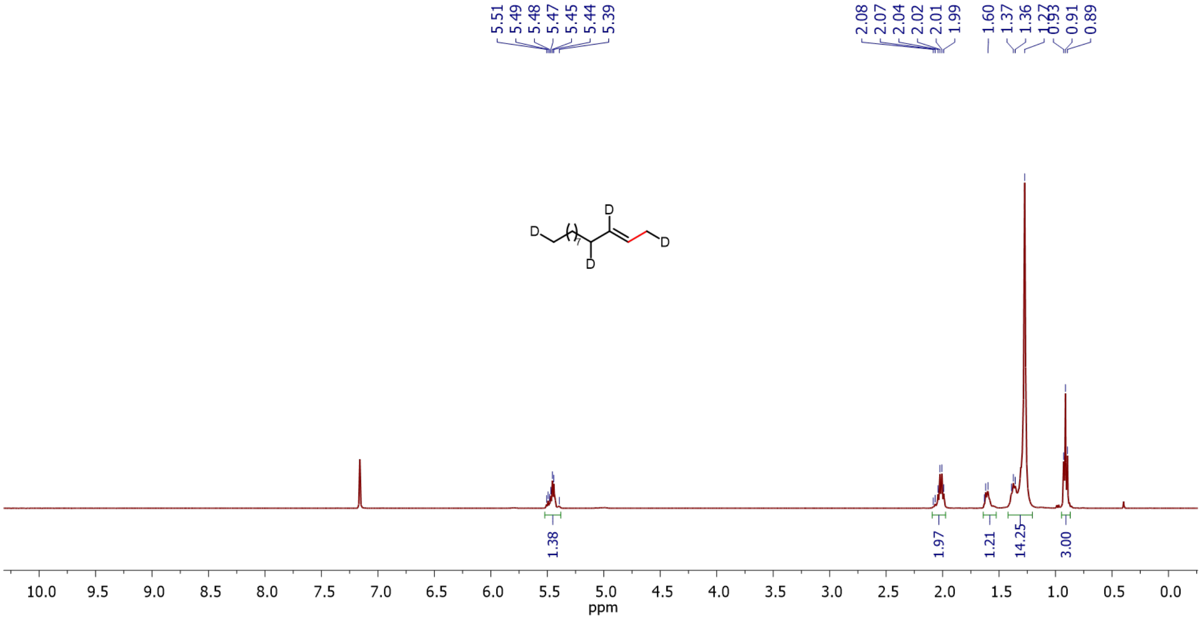

Figure S98. ^{1}H NMR spectrum (400 MHz, C_6D_6) after isomerization of dodec-1-ene-1,1-d_2 with catalyst **4**.

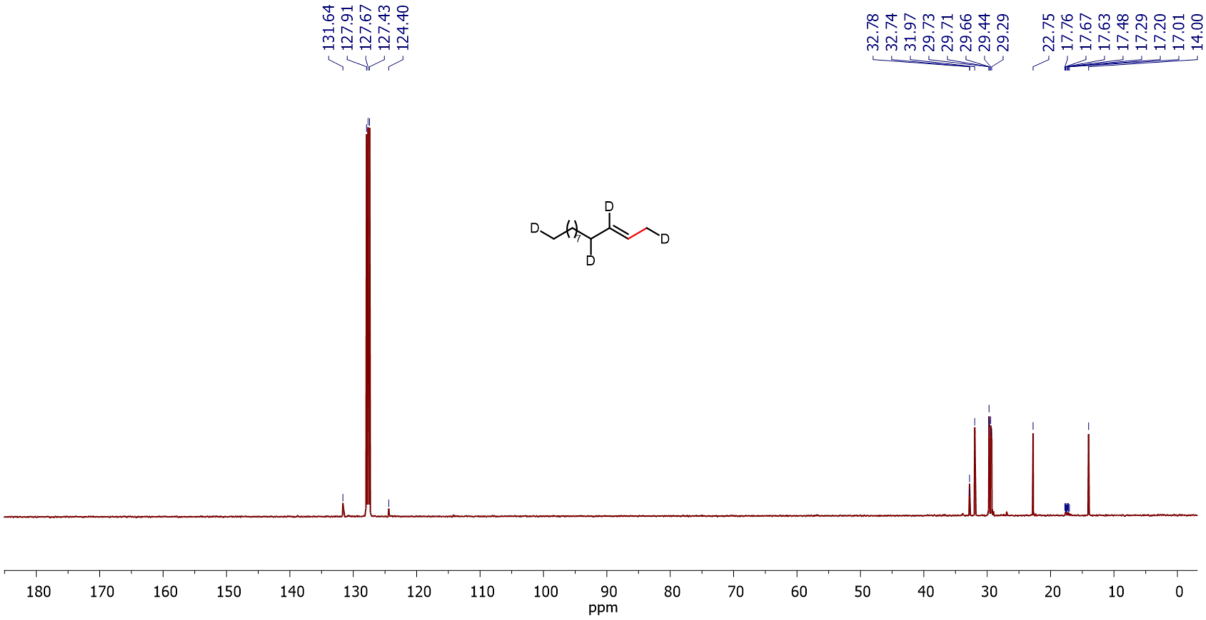

Figure S99. $^{13}C\ \{^{1}H\}$ NMR spectrum (101 MHz, C_6D_6) after isomerization of dodec-1-ene-1,1-d_2 with catalyst **4**.

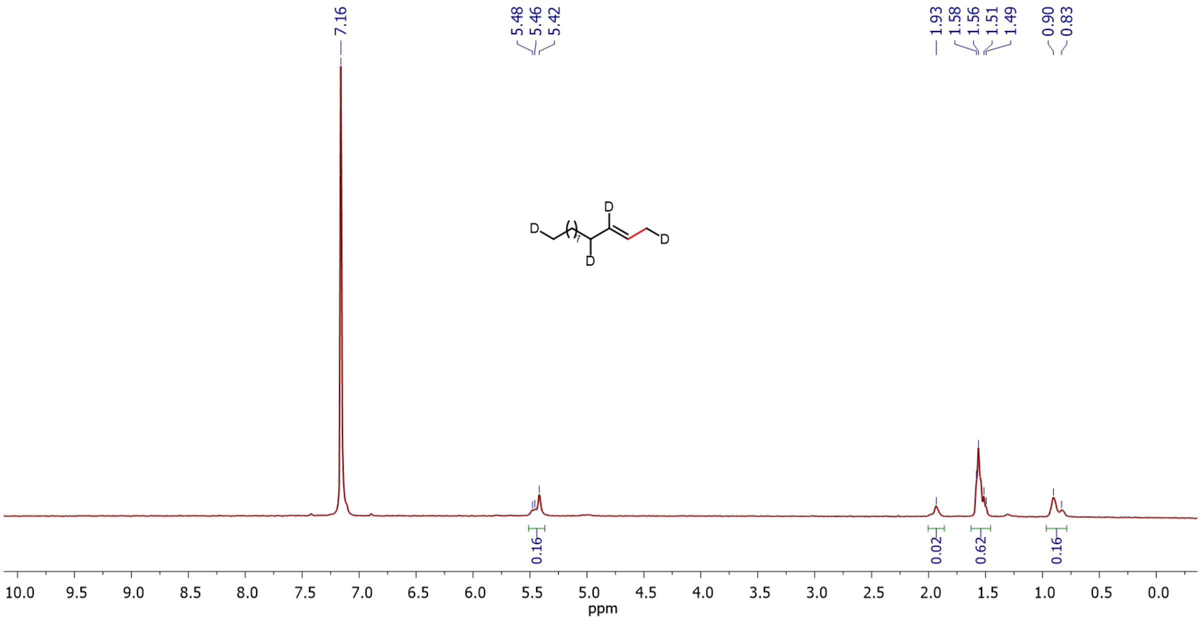

Figure S100. ^{2}H NMR spectrum (46.07 MHz) after isomerization of dodec-1-ene-1,1-d_2 with catalyst **4** in benzene.

Radical Trapping Experiments.

Control studies to exclude radical pathway. According to general procedure (A), inside the glovebox, an oven-dried J-Young tube was charged with catalyst **3** dissolved in THF (100 µL, 1 mol%, 0.03 mM stock solution). Next the solvent was evaporated under reduced pressure, allyl benzyl ether (45 mg, 0.3 mmol) and additive (0.15 mmol) were added together with 400 µL of benzene-d_6. The J-Young tube was sealed, taken out of the glovebox, and heated at 60˚C for 5h. After the reaction was completed, the J-Young tube was exposed to air and the contents filtered through a short plug of neutral alumina to remove the cobalt catalyst. The alumina was washed with an additional benzene-d_6 (400 µL*)* to collect all the organic products. ^{1}H and ^{13}C NMR spectrum was taken to determine the product conversion.

In the presence of radical trapping reagents 9,10-dihydroanthracene, 1,1-diphenylethylene or xanthene were not hampered the isomerization yield.

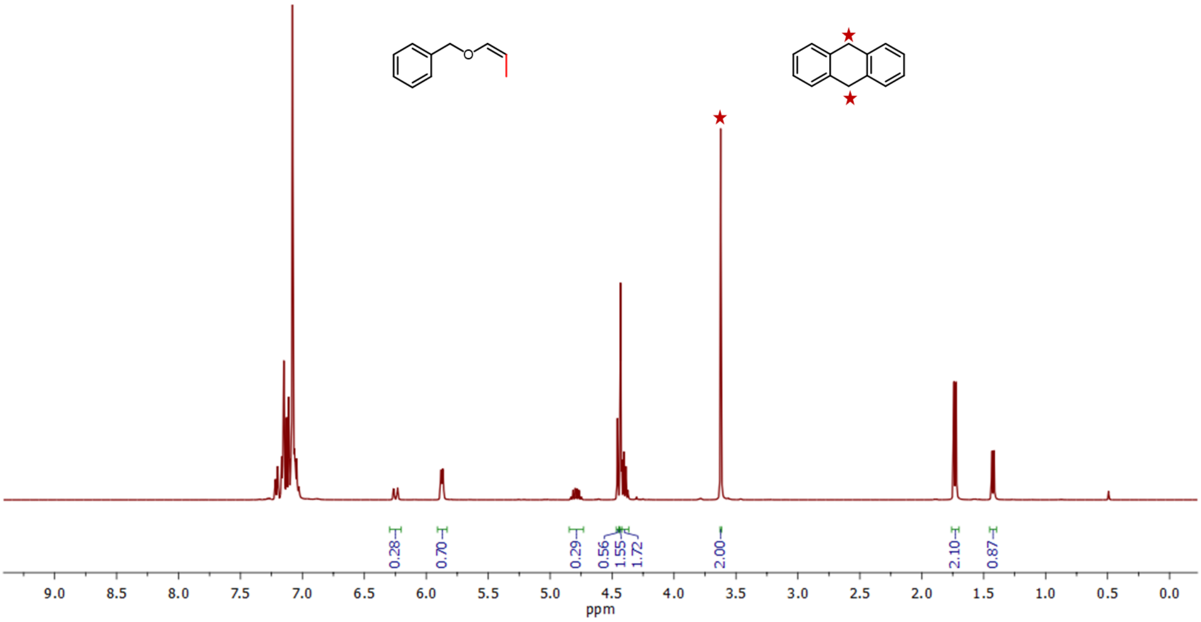

Figure S101. ^{1}H NMR spectrum (400 MHz) after isomerization of allyl benzyl ether in presence of 9,10-dihydroanthracene with catalyst **3** in benzne-d_6.

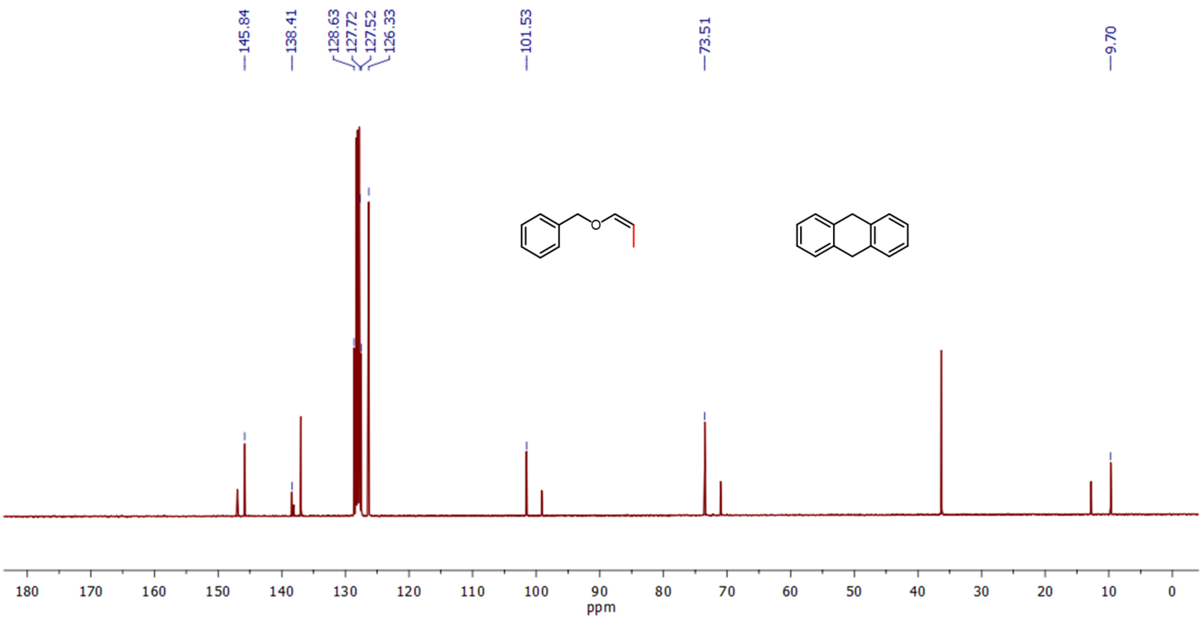

Figure S102. ^{13}C {^{1}H} NMR spectrum (101 MHz) after isomerization of allyl benzyl ether in presence of 9,10-dihydroanthracene with catalyst **3** in benzne-d_6.

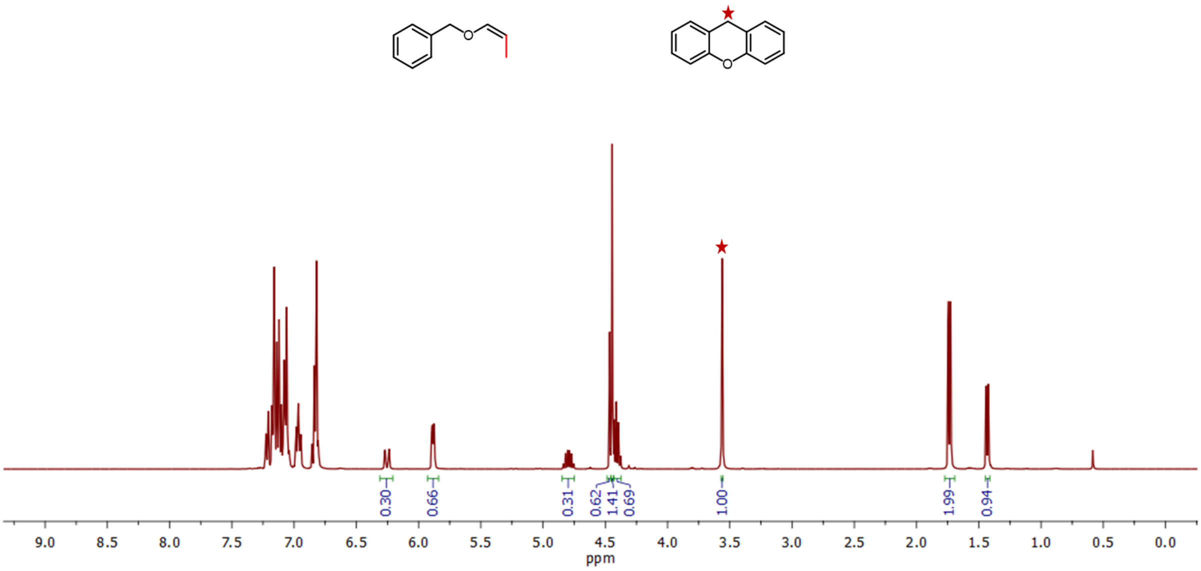

Figure S103. ^{1}H NMR spectrum (400 MHz) after isomerization of allyl benzyl ether in presence of xanthene with catalyst **3** in benzne-d_6.

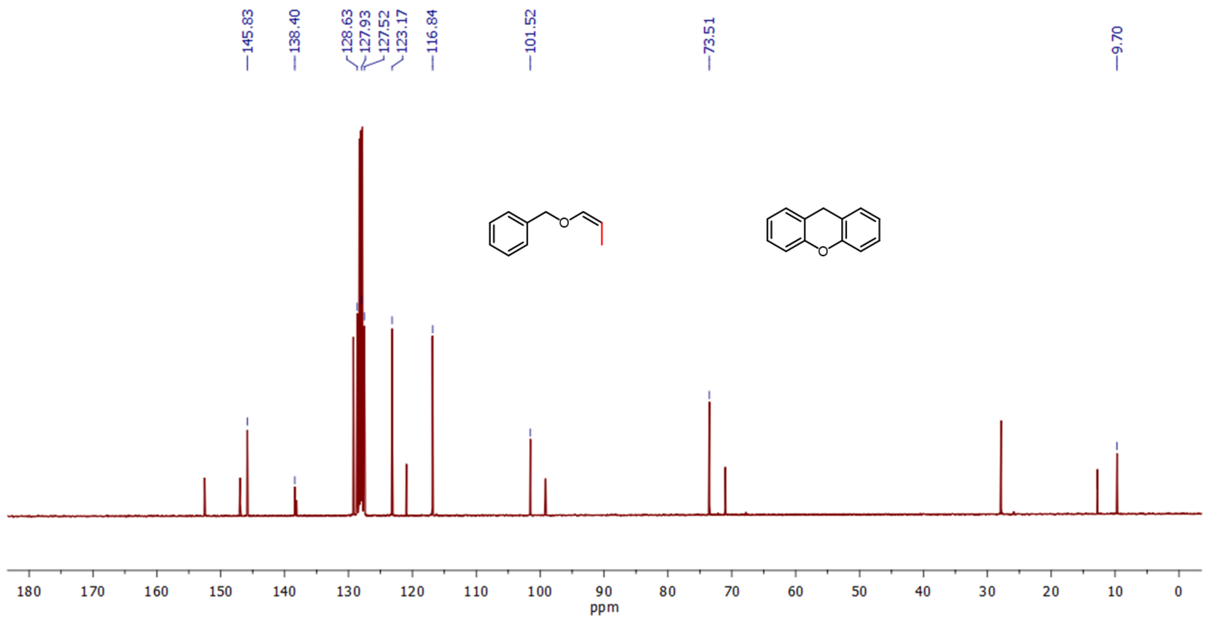

Figure S104. $^{13}C\{^{1}H\}$ NMR spectrum (101 MHz) after isomerization of allyl benzyl ether in presence of xanthene with catalyst **3** in benzne-d_6.

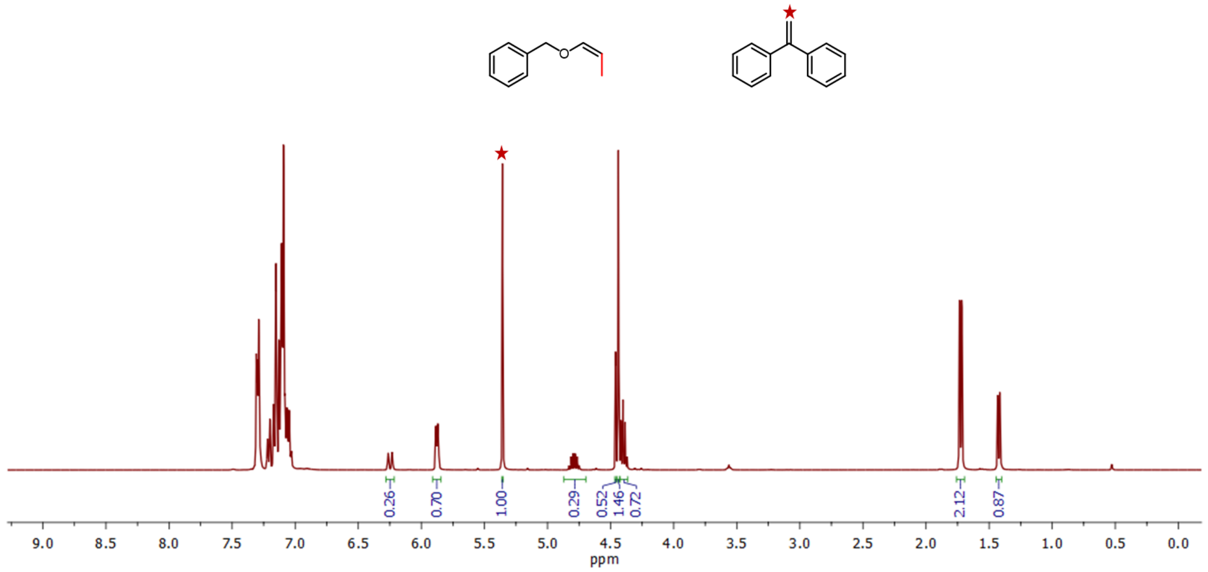

Figure S105. ^{1}H NMR spectrum (400 MHz) after isomerization of allyl benzyl ether in presence of 1,1-diphenylethylene with catalyst 3 in benzne-d_6.

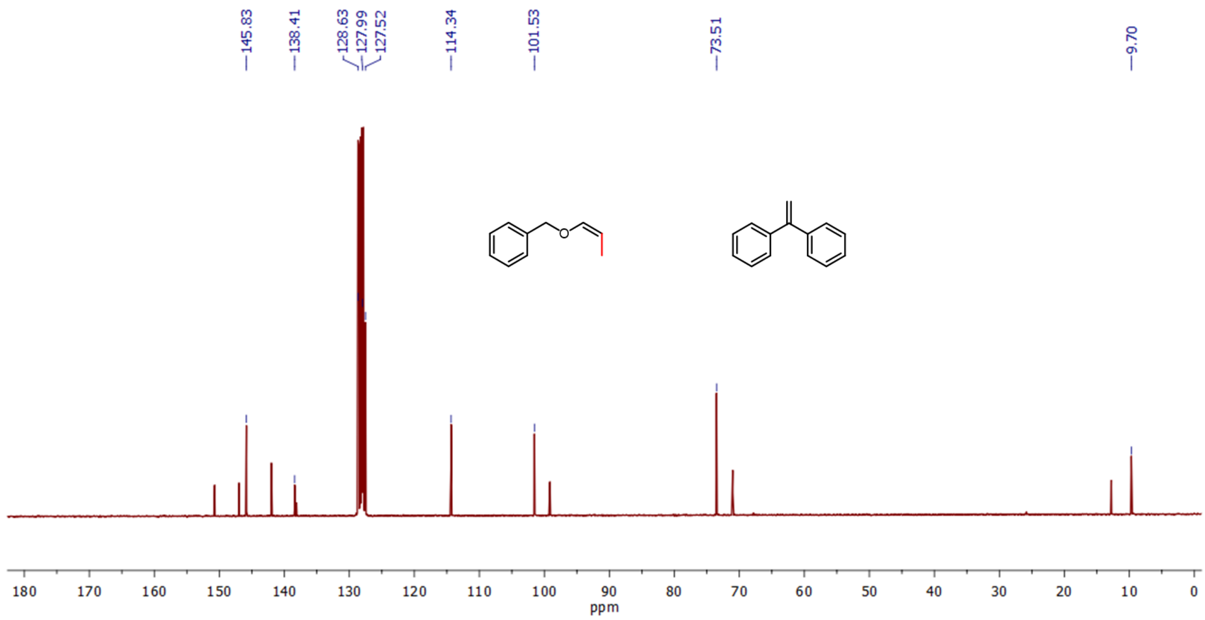

Figure S106. ^{13}C {^{1}H} NMR spectrum (101 MHz) after isomerization of allyl benzyl ether in presence of 1,1-diphenylethylene with catalyst **3** in benzne-d_6.

According to general procedure (B), inside the glovebox, an oven-dried J-Young tube was charged with allyl benzyl ether (26 mg, 0.15 mmol), additive (0.075) and catalyst **4** in toluene-d_8 (125 μL, 0.0075 mmol, 5.0 mol% from a 0.06 mM stock solution prepared in toluene-d_8). To the reaction mixture was added an additional amount of benzene-d_6 to make total volume 400 μL The J-Young tube was sealed, taken out of the glovebox, and heated at 80˚C for 5h. After the reaction was completed, the J-Young tube was exposed to air and the contents filtered through a short plug of neutral alumina to remove the cobalt catalyst. The alumina was washed with an additional toluene-d_8 (400 μL) to collect all the organic products. ^{1}H and ^{13}C NMR spectrum was taken to determine the product conversion.

In the presence of radical trapping reagents 9,10-dihydroanthracene, 1,1-diphenylethylene or xanthene were not hampered the isomerization yield.

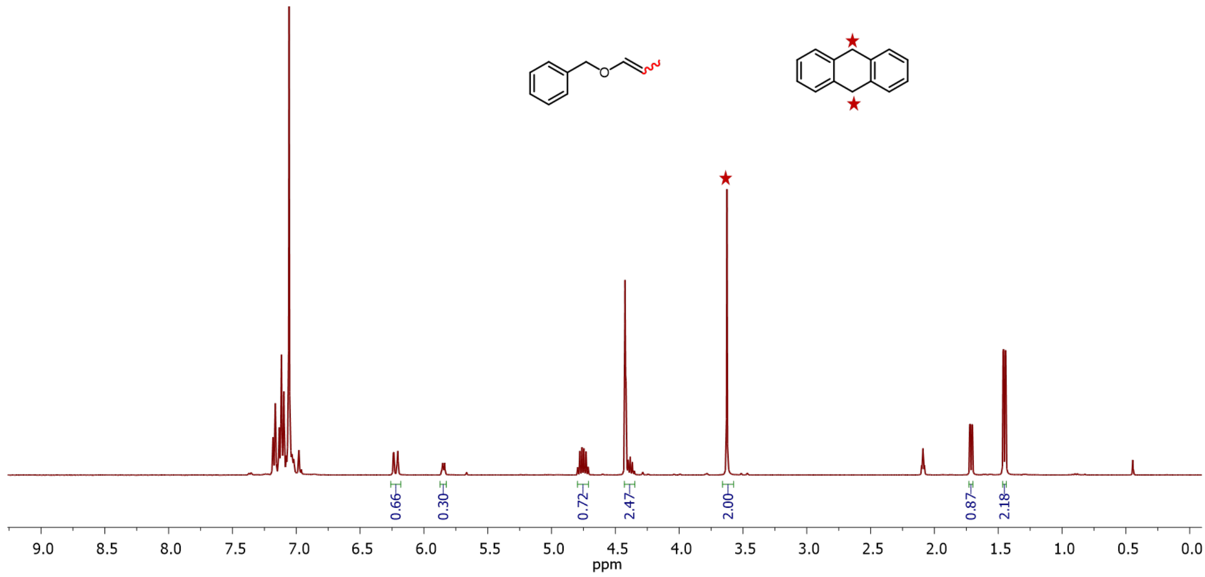

Figure S107. ^{1}H NMR spectrum (400 MHz) after isomerization of allyl benzyl ether in presence of 9,10-dihydroanthracene with catalyst **4** in toluene-d_8.

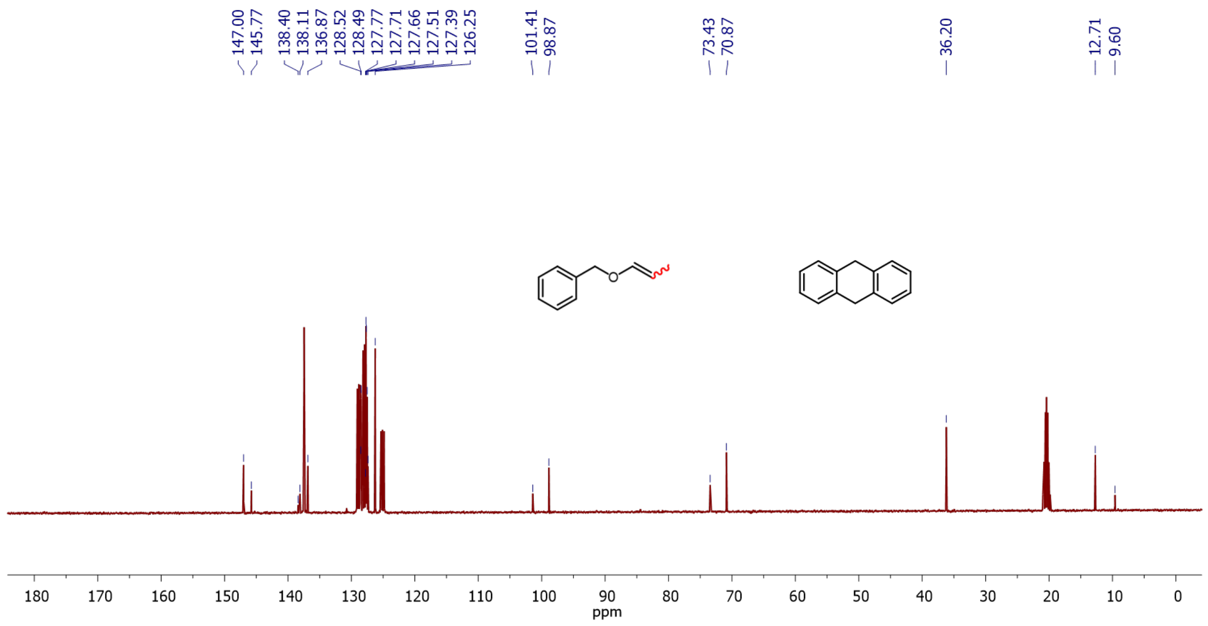

Figure S108. ^{13}C {^{1}H} NMR spectrum (101 MHz) after isomerization of allyl benzyl ether in presence of 9,10-dihydroanthracene with catalyst **4** in toluene-d_8.

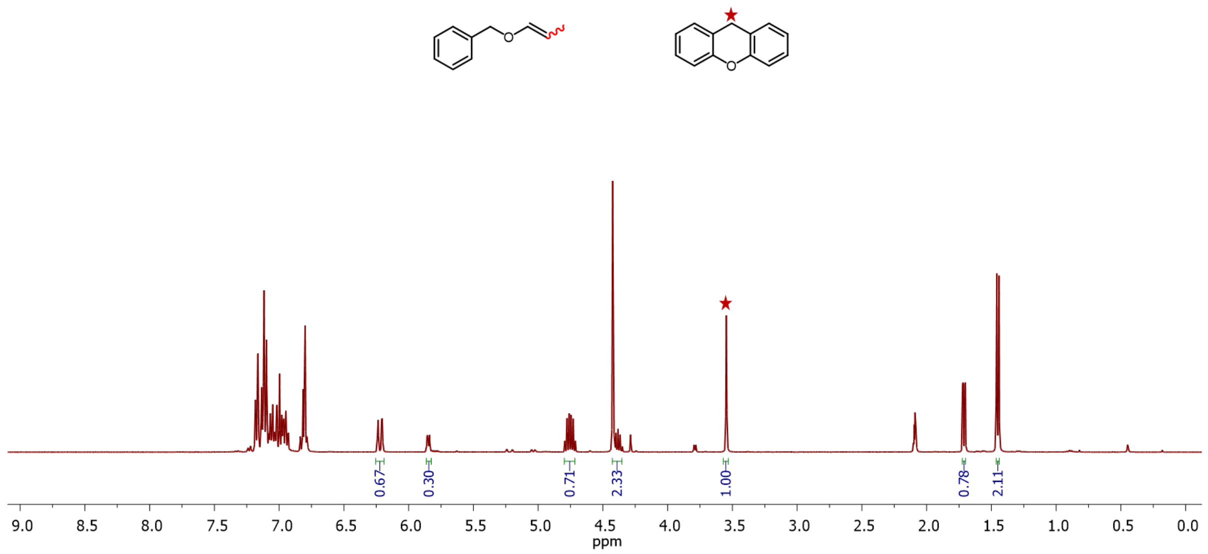

Figure S109. ^{1}H NMR spectrum (400 MHz) after isomerization of allyl benzyl ether in presence of xanthene with catalyst **4** in toluene-d_8.

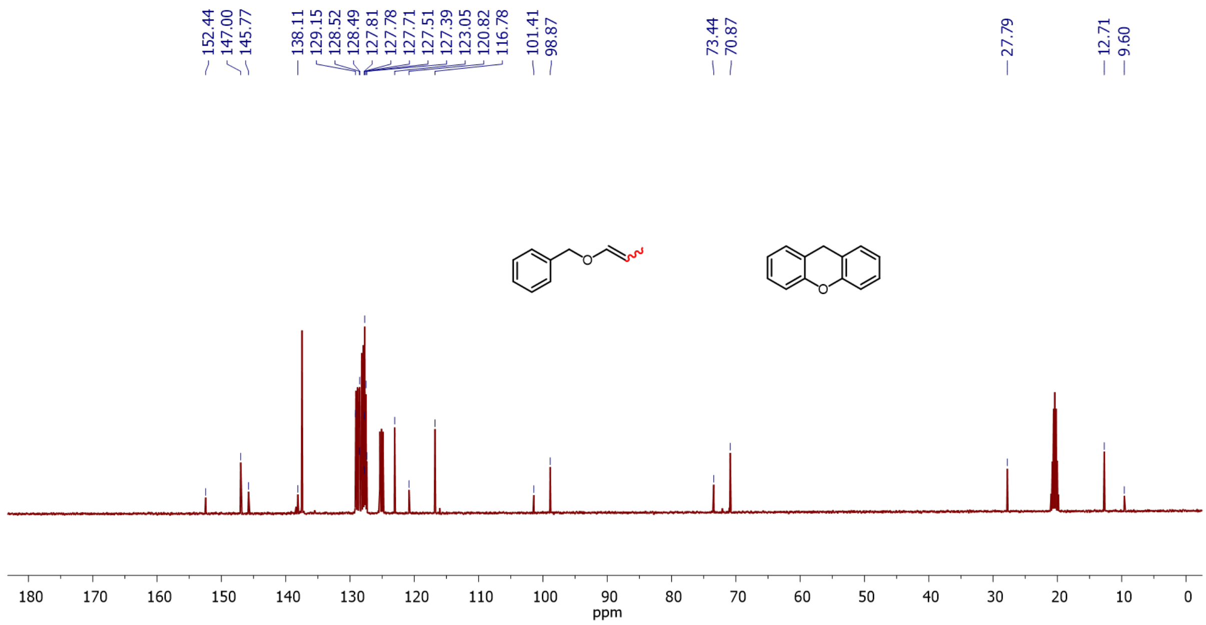

Figure S110. ^{13}C {^{1}H} NMR spectrum (101 MHz) after isomerization of allyl benzyl ether in presence of xanthene with catalyst **4** in toluene-d_8.

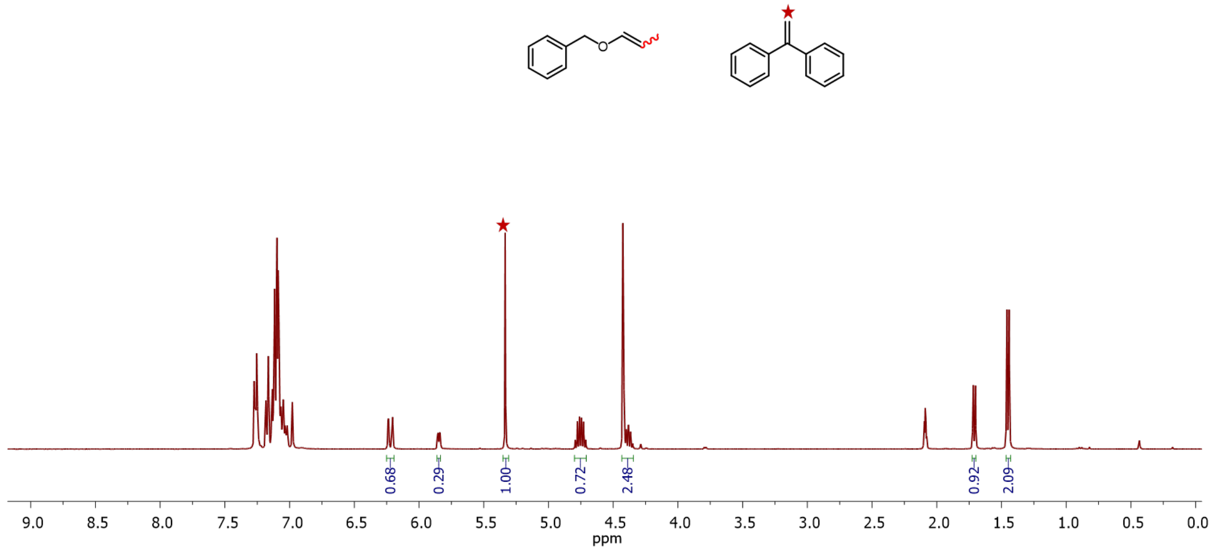

Figure S111. ^{1}H NMR spectrum (400 MHz) after isomerization of allyl benzyl ether in presence of 1,1-diphenylethylene with catalyst **4** in toluene-d_8.

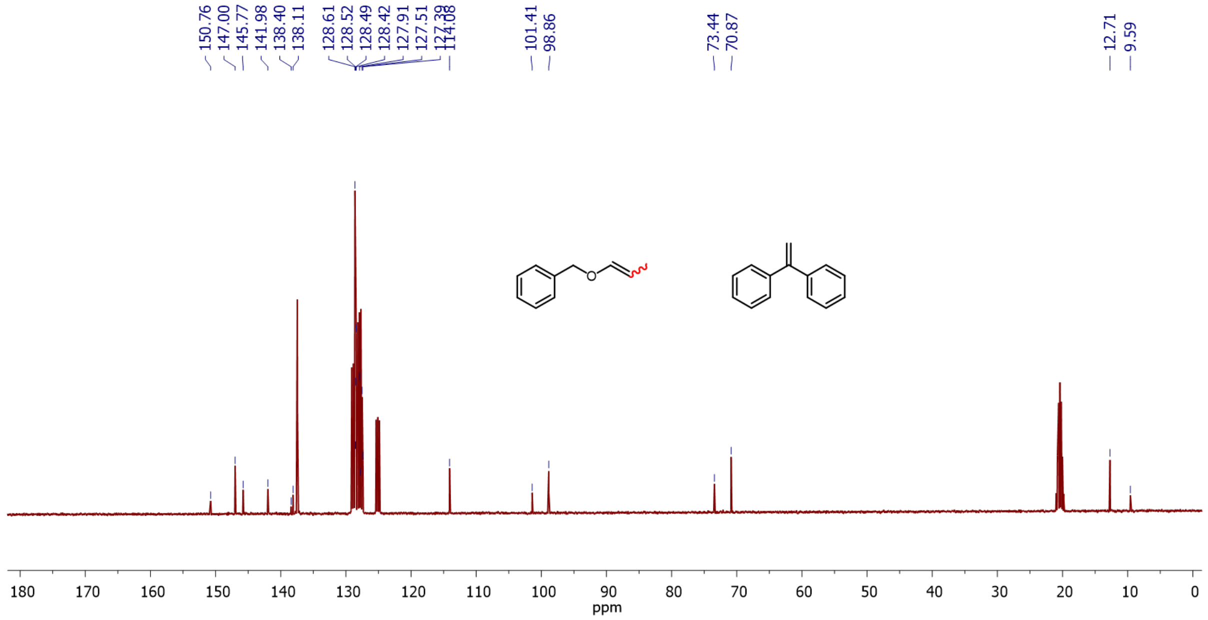

Figure S112. ^{13}C {^{1}H} NMR spectrum (101 MHz) after isomerization of allyl benzyl ether in presence of 1,1-diphenylethylene with catalyst **4** in toluene-d_8.

Experiments performed to decoordinate N_2 ligand from compound 3.

Inside the N_2 filled glovebox, an oven-dried J-Young tube was charged with $[((PC_{NHC}P)Co)_2\text{-}\mu\text{-}N_2](BAr_4^F)_2$ (**3**; 30 mg, 0.01 mmol) dissolved in 450 µL THF-*d*$_8$. Next, three cycles of freeze-pump-thaw was performed then the sample was sealed in vacuum. The changes in the mixture was monitored by NMR spectroscopy. Further, 4-dimethylaminopyridine (DMAP; 3 mg, 0.024 mmol) was added into the sample and the changes were observed by NMR spectroscopy.

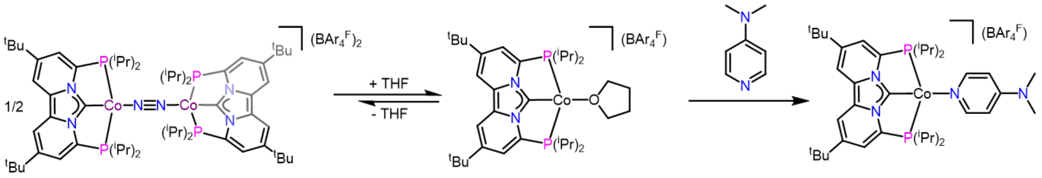

3 **M** **N**

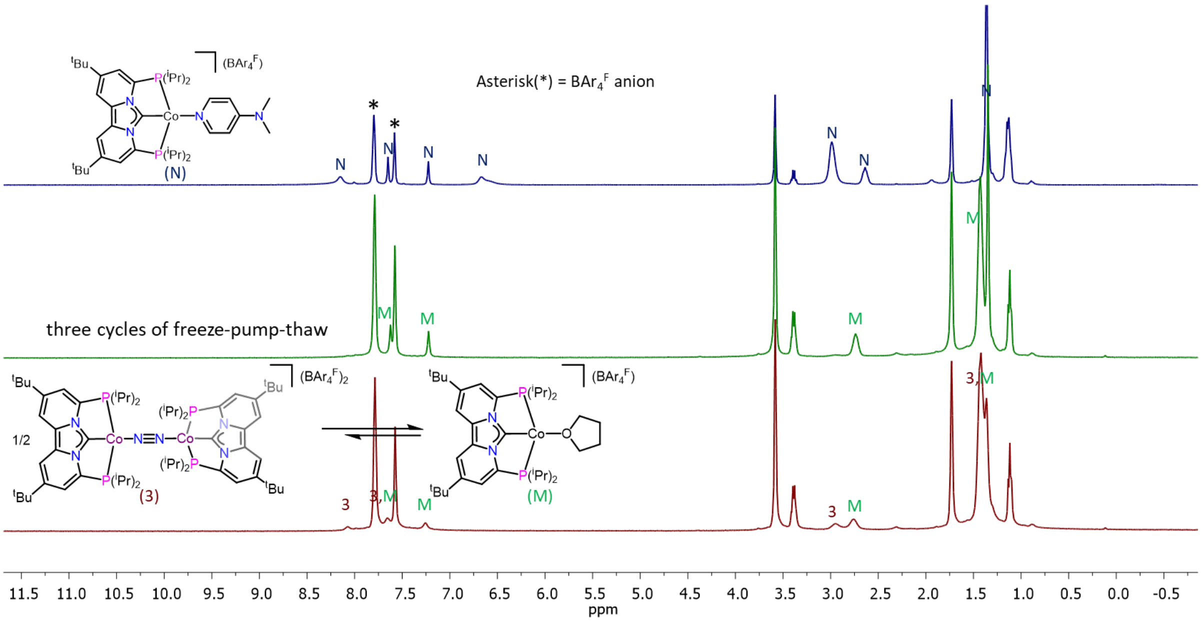

Figure S113. ^{1}H NMR spectra (400 MHz) of N_2 ligand replacement in thf-*d*$_8$.

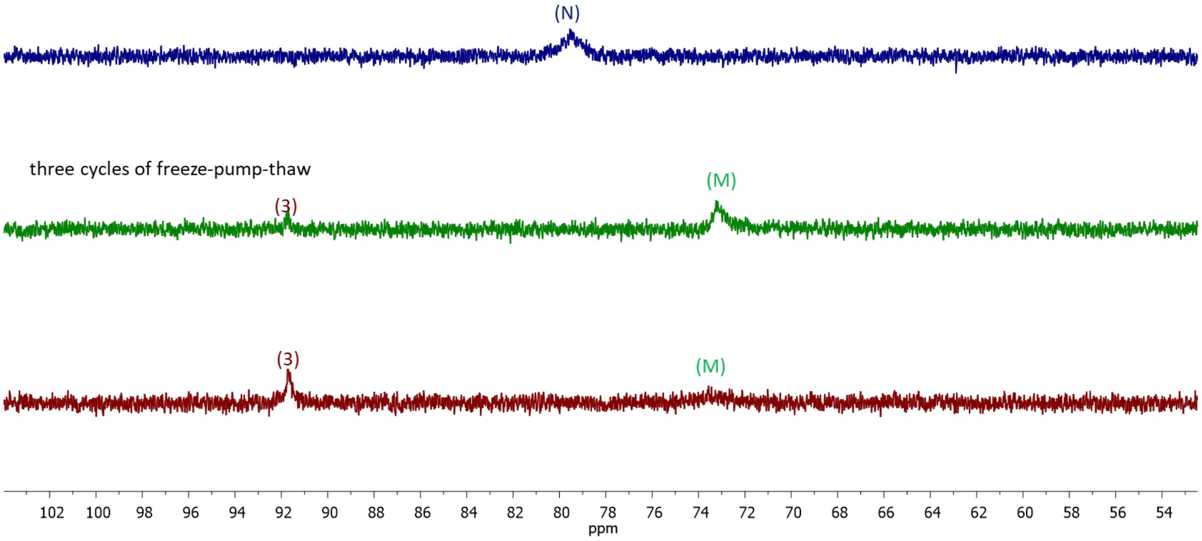

Figure S114. ^{31}P NMR spectrum (162 MHz) of N_2 ligand replacement in thf-*d*$_8$.

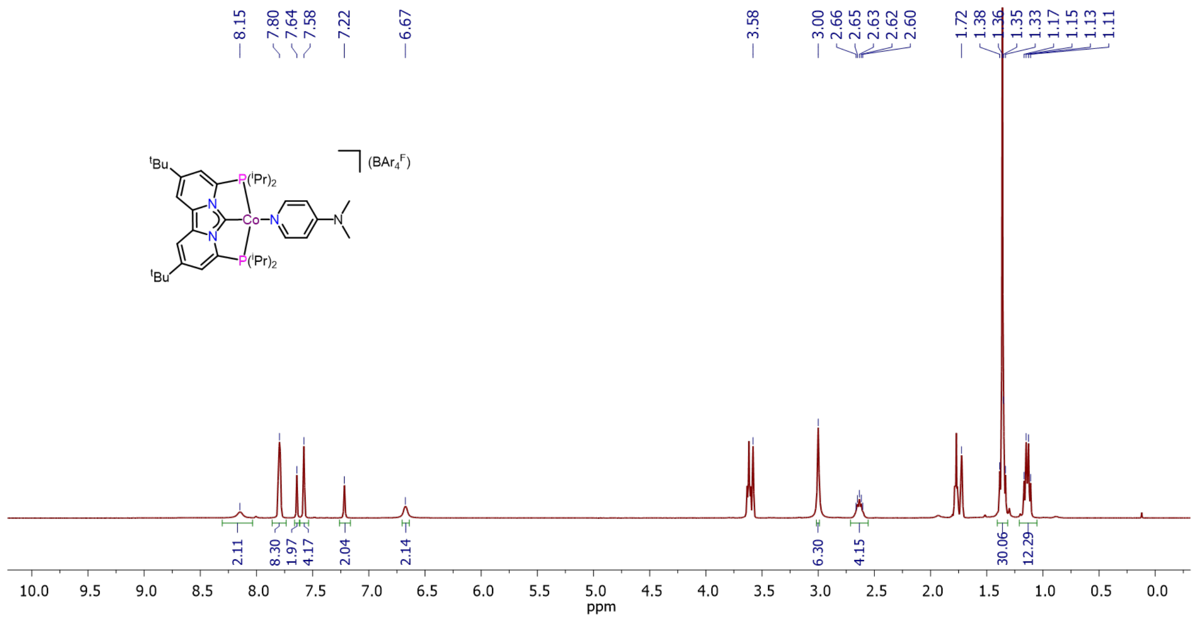

Figure S115. ^{1}H NMR spectrum (400 MHz) of [(PC$_{NHC}$P)Co(DMAP)](BAr$_4$^{F}) in thf-d_8.

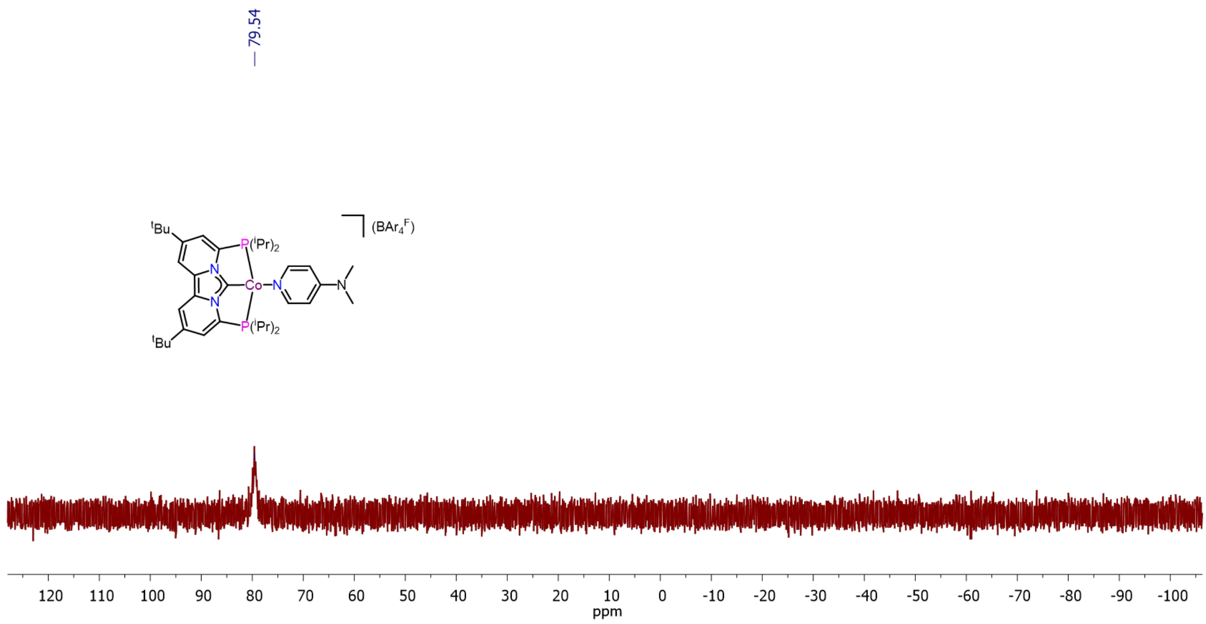

Figure S116. ^{31}P NMR spectrum (162 MHz) of [(PC$_{NHC}$P)Co(DMAP)](BAr$_4$^{F}) in thf-d_8.

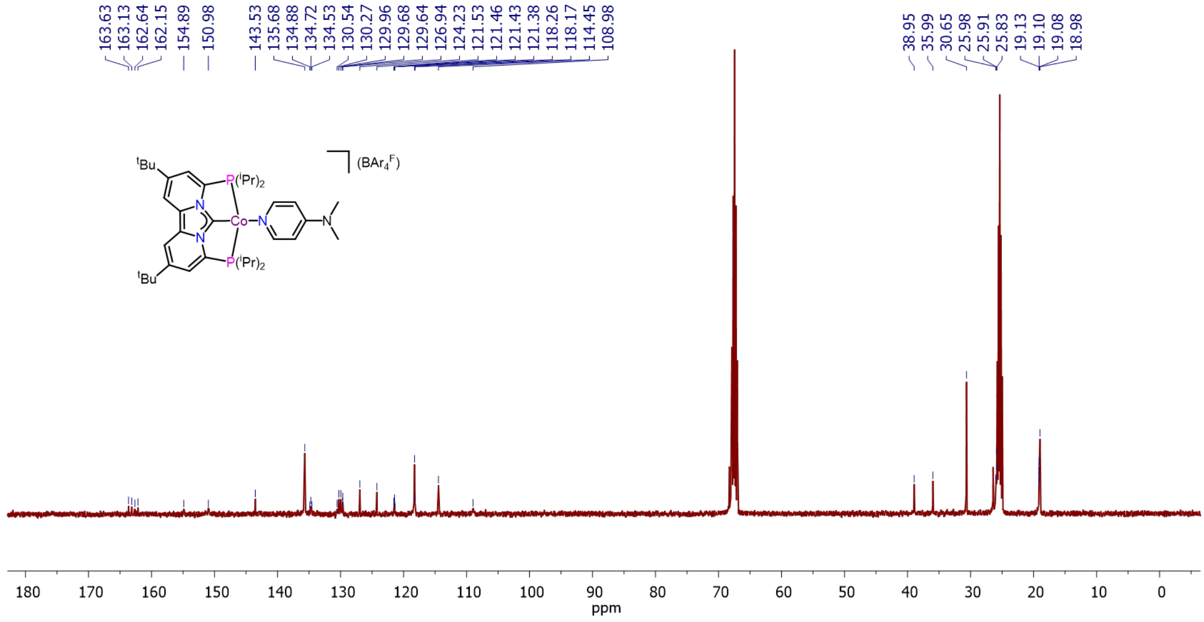

Figure S117. $^{13}C\ \{^{1}H\}$ NMR spectrum (101 MHz) of $[(PC_{NHC}P)Co(DMAP)](BAr_4^F)$ in thf-d_8.

Isomerization of allyl benzyl ether with [(PC$_{NHC}$P)Co(DMAP)](BAr$_4^F$) (5). According to general procedure, inside the glovebox, an oven-dried J-Young tube was charged with [(PC$_{NHC}$P)Co)(DMAP)](BAr$_4^F$) dissolved in THF (200 µL, 2 mol%, 0.03 mM stock solution). Next, the solvent was evaporated under reduced pressure and allyl benzyl ether (51 mg, 0.3 mmol) was added together with 400 µL of benzene-*d$_6$*. The progress of the reaction was monitored by ^{1}H NMR spectroscopy.

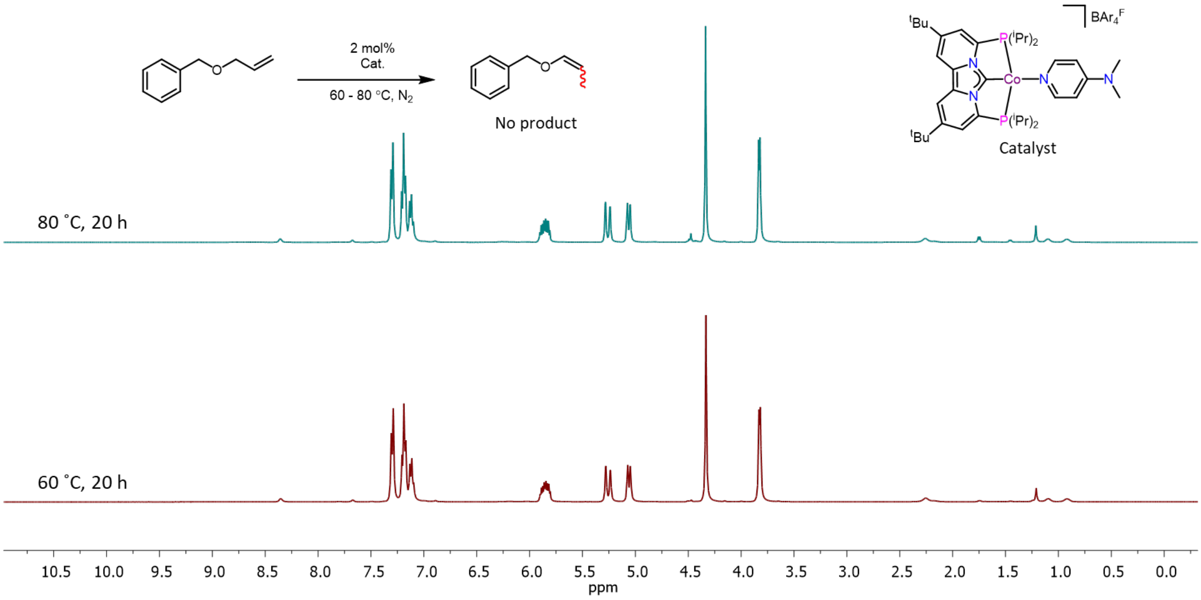

Figure S118. ^{1}H NMR spectrum (400 MHz) of isomerization of allyl benzyl ether with [(PC$_{NHC}$P)Co)(DMAP)](BAr$_4^F$) in benzene-*d$_6$*.

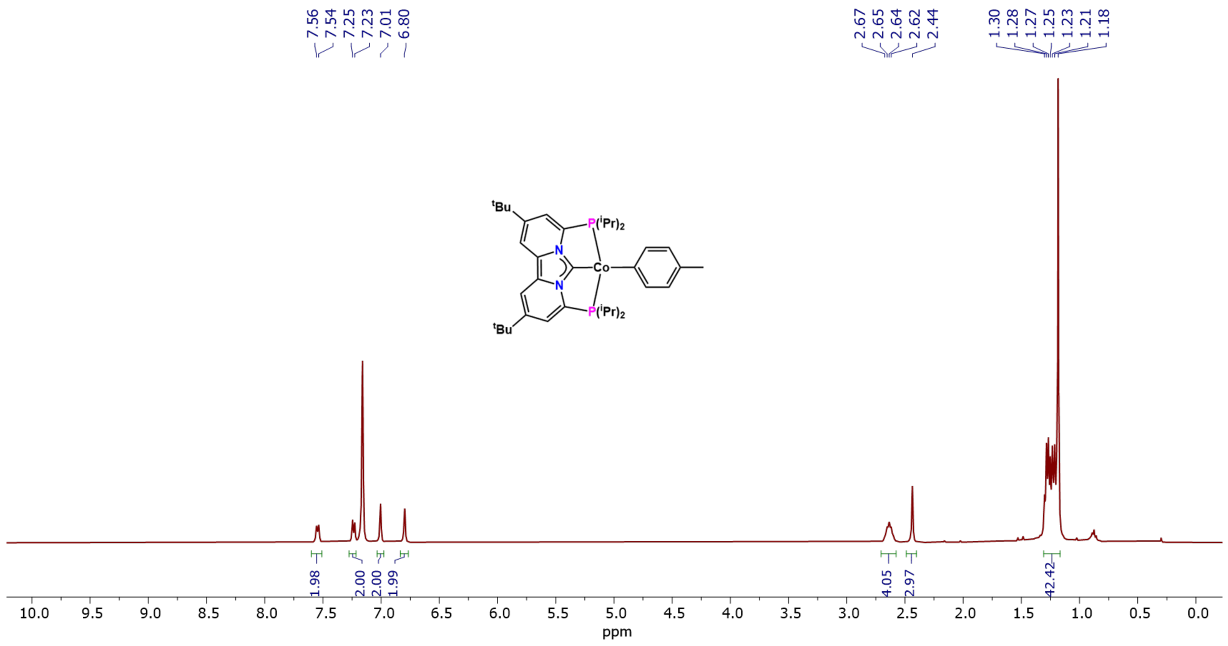

Figure S119. ^{1}H NMR spectrum (400 MHz) of [(PC$_{NHC}$P)Co(p-tol)] in benzene-d_6.

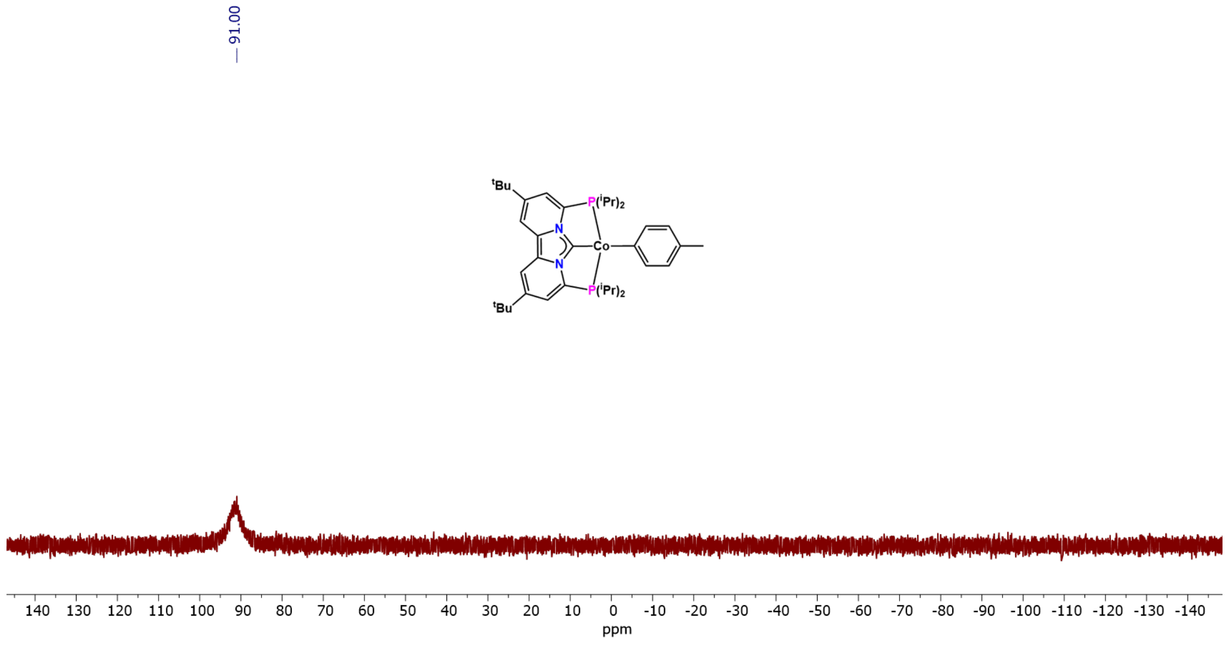

Figure S120. ^{31}P NMR spectrum (162 MHz) of [(PC$_{NHC}$P)Co(p-tol)] in benzene-d_6.

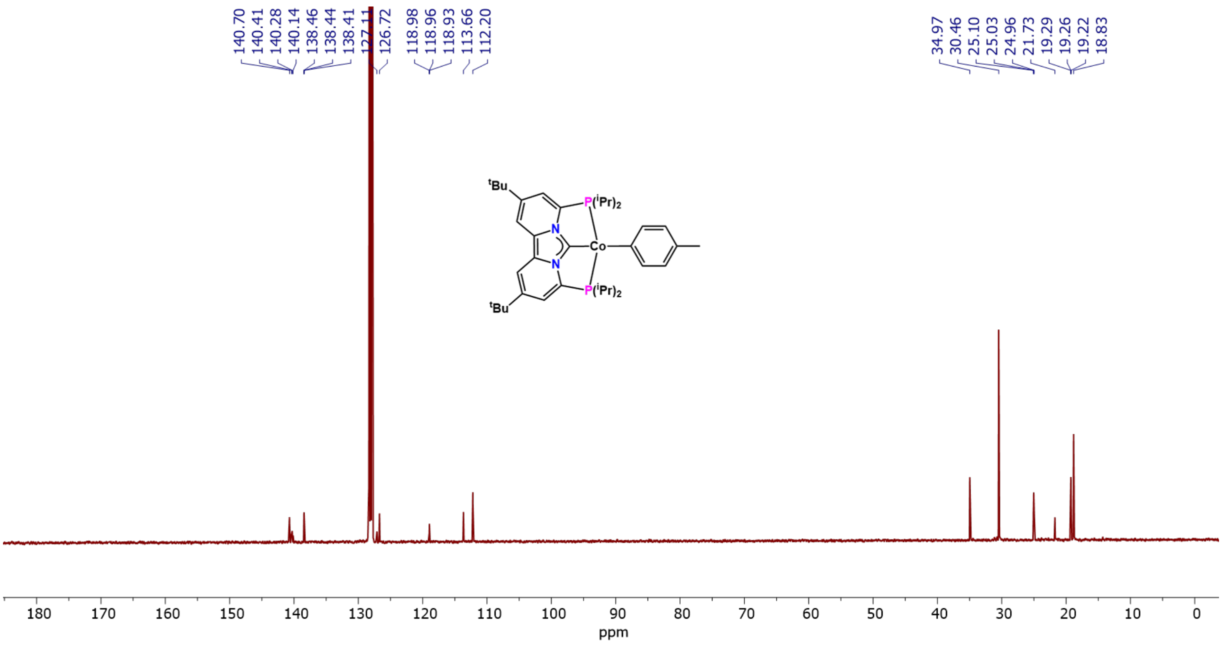

Figure S121. $^{13}C\{^{1}H\}$ NMR spectrum (101 MHz) of [(PC$_{NHC}$P)Co(p-tol)] in benzene-*d$_6$*.

Isomerization of allyl benzyl ether with [(PC$_{NHC}$P)Co(p-tol)] (6). According to general procedure (B), inside the glovebox, an oven-dried J-Young tube was charged with substrate (0.15 mmol) and catalyst **6** in benzene-*d$_6$* (0.0075 mmol (5 mol%), from a 0.06M stock in benzene-*d$_6$*) was added. To the reaction mixture was added an additional amount of benzene-*d$_6$* to make total volume 400 μL. The tube was sealed, taken out of the glovebox, and heated at 90 °C until the reaction was complete. The reaction was monitored by ^{1}H NMR spectroscopy.

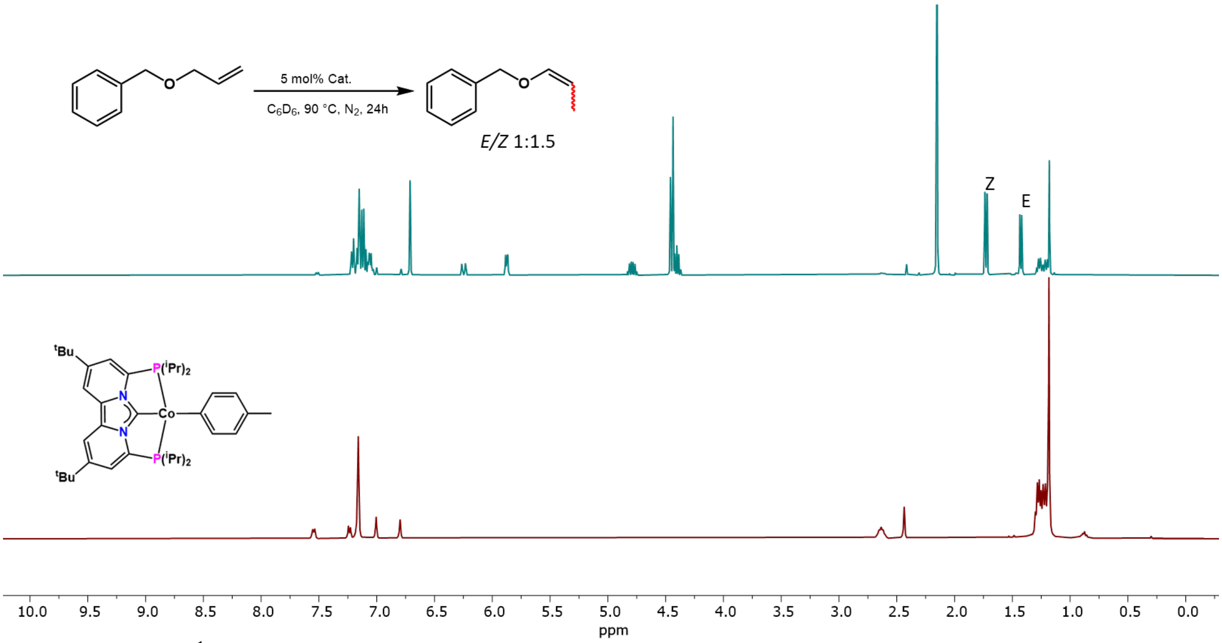

Figure S122. ^{1}H NMR spectrum (400 MHz) of isomerization of allyl benzyl ether with [(PC$_{NHC}$P)Co)(p-tol)] in benzene-*d$_6$*.

Experiments performed to observe methyl migration

Inside the glovebox, an oven-dried J-Young tube was charged with catalyst 4 (15 mg, 0.026 mmol) dissolved in 450 µL benzene-d_6. Next the purple solution was frozen to -110 ˚C then PPh_3 (8 mg, 0.03 mmol) or PMe_3 (2.5 mg, 0.032 mmol from 0.9 M in 2-methyltetrhydrofuran) or IMes Carbene (9 mg, 0.03) was added. The progress of the reaction was monitored by ^{1}H NMR spectroscopy at different time intervals.

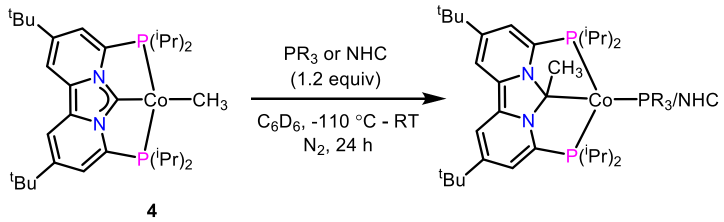

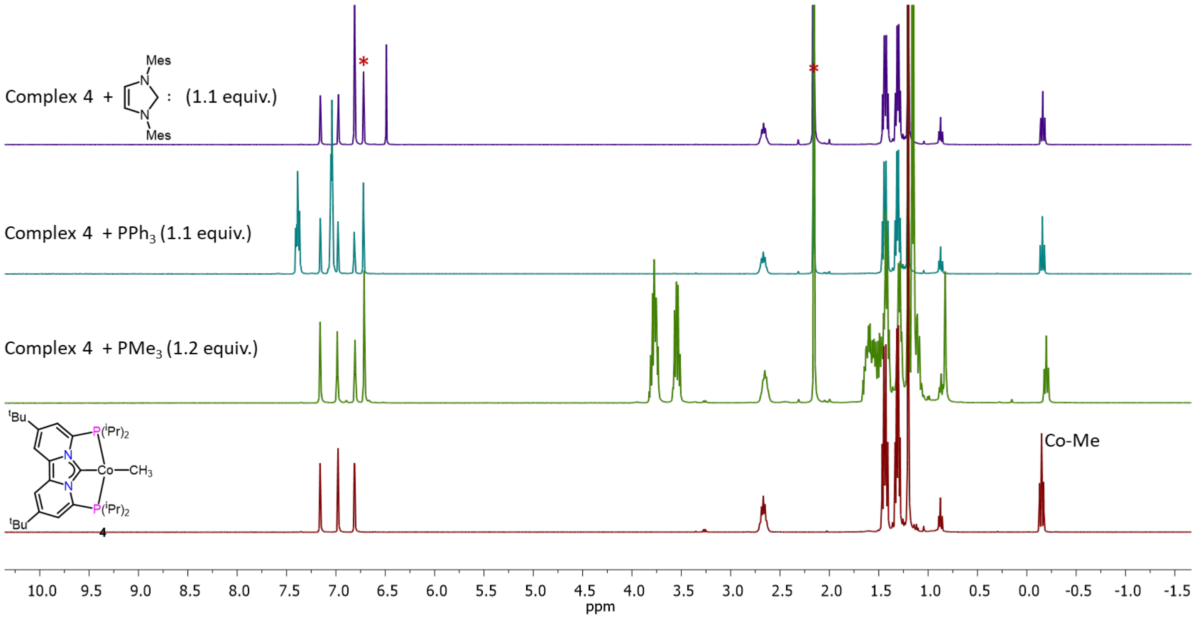

Figure S123. ^{1}H NMR spectrum (400 MHz) of Complex 4 with various monodentate ligand (PPh_3, PMe_3 and IMes carbene) in benzene-d6 after 24h. Asterisk (*) indicates internal standard mesitylene (the reaction mixture was heated at 50 ˚C - 80 ˚C for 12h and the complex 4 remains unchanged).

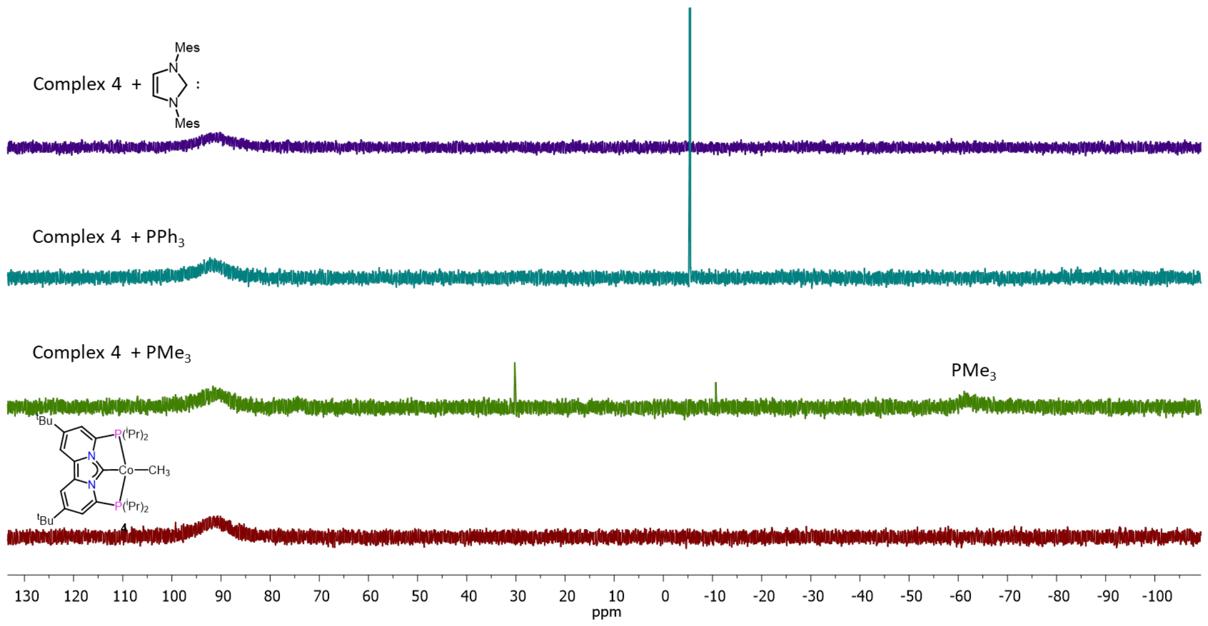

Figure S124. ^{31}P NMR spectrum (162 MHz) of Complex 4 with various monodentate ligand (PPh_3, PMe_3 and IMes carbene) in benzene-d_6 after 24h.

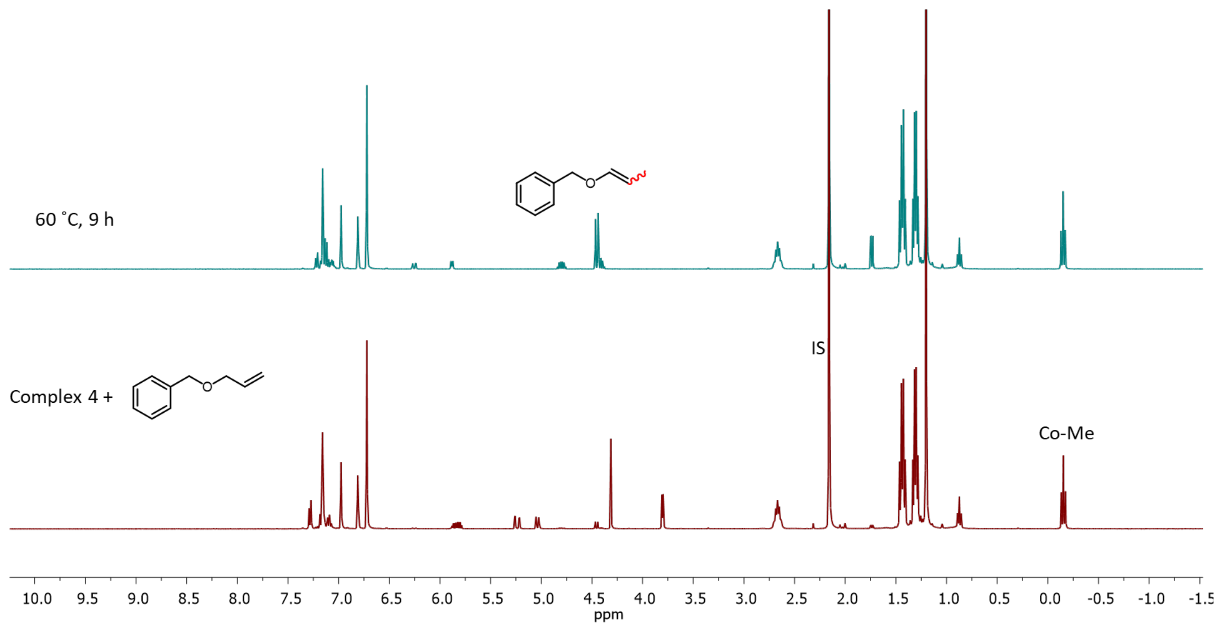

Figure S125. ^{1}H NMR spectrum (400 MHz) of (1:1) mixture of complex 4 and allyl benzyl ether in benzene-d_6 after 9 h at 60 ˚C.

Catalytic cycle for allyl ether isomerization.

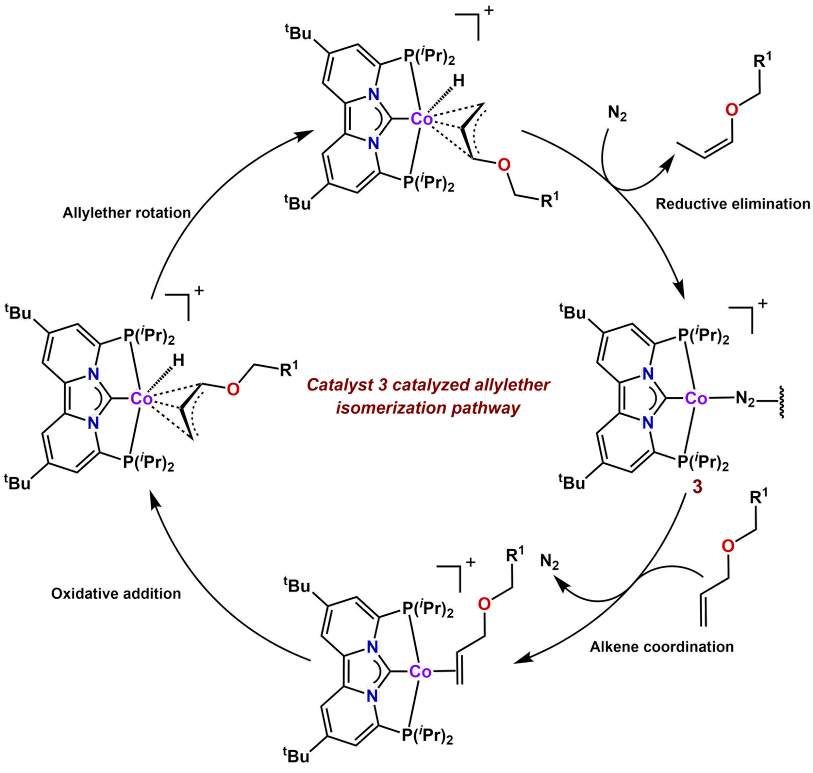

Figure S126. Plausible mechanism for allyl ether isomerization by catalyst 3.

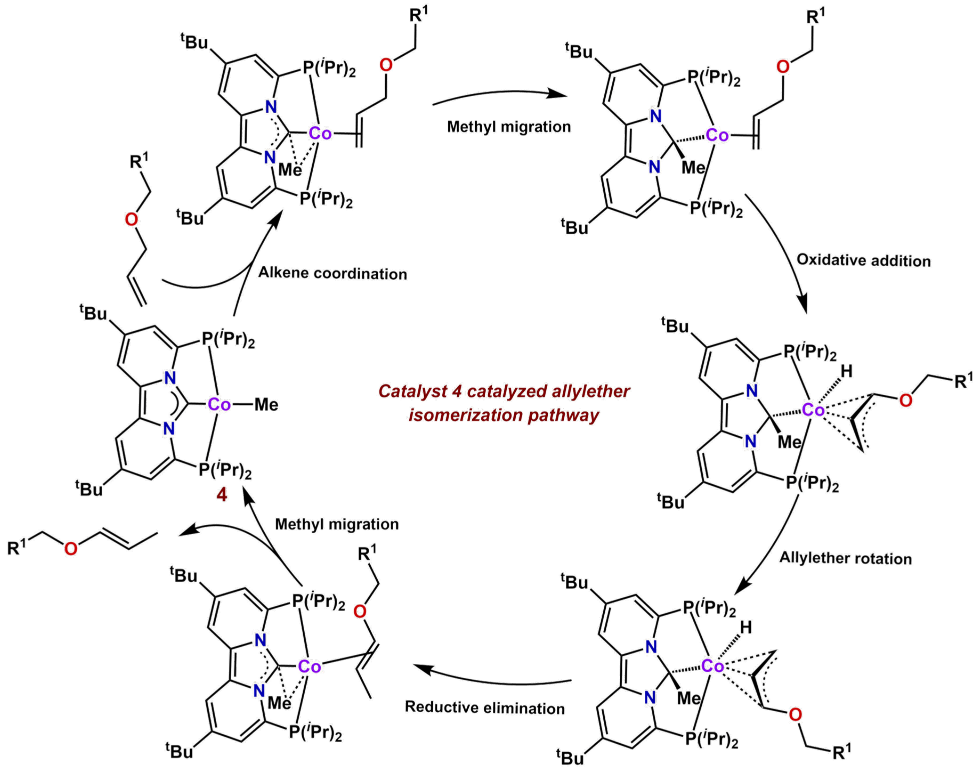

Figure S127. Plausible mechanism for allyl ether isomerization by catalyst 4.

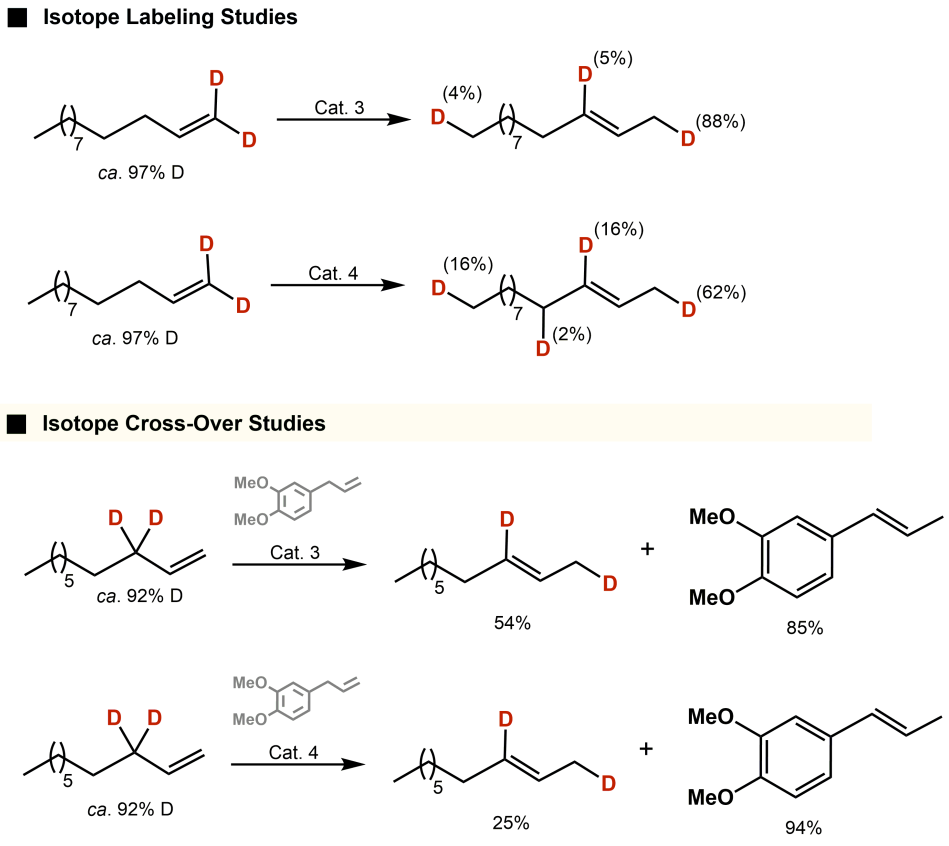

Figure S128. Isotope labelling studies for alkene isomerization by catalyst 3 and 4.

X-Ray Crystallography

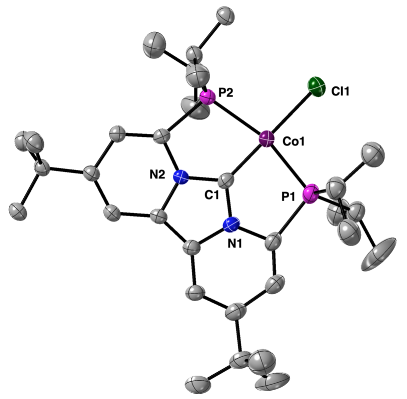

Figure S129. Solid state structure of [((PC$_{NHC}$P)CoCl)] (**2**). Ellipsoids are shown at the 30% probability level. Hydrogen atoms and co-crystallized solvent molecules are not shown for clarity.

Special Refinement Details for 2. Compound **2** crystallizes in the orthorhombic space group p b c a, with one molecule in the asymmetric unit. No further special refinement conditions were necessary.

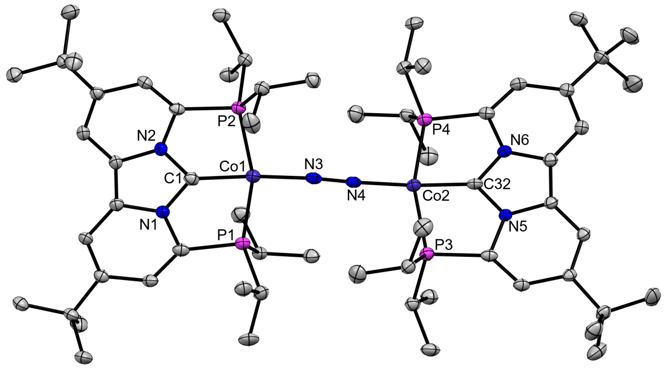

Figure S130. Solid state structure of $[(PC_{NHC}P)Co)_2(\mu\text{-}N_2)](BAr_4^F)_2$ (**3**). Ellipsoids are shown at the 30% probability level. Hydrogen atoms and co-crystallized solvent molecules are not shown for clarity.

Special Refinement Details for 3. Compound **3** crystallizes in the triclinic space group P-1, with one molecule in the asymmetric unit. No further special refinement conditions were necessary.

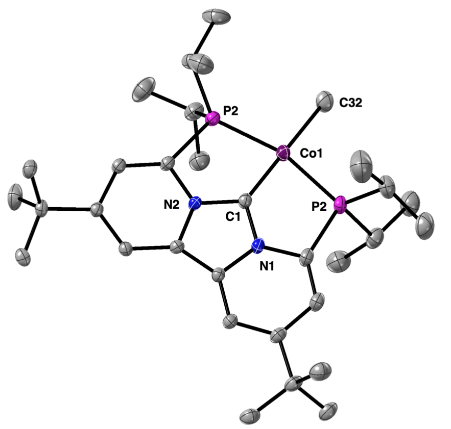

Figure S131. Solid state structure of [(($PC_{NHC}P$)CoMe)] (**4**). Ellipsoids are shown at the 30% probability level. Hydrogen atoms and co-crystallized solvent molecules are not shown for clarity.

Special Refinement Details for 4. Compound [($PC_{NHC}P$)Co(Me)] crystallizes in the tetragonal space group I -4, with one molecule in the asymmetric unit. Solvent molecules were squeezed to improve the refinement parameters. No further special refinement conditions were necessary.

Tables

Table S2. Selected bond angles and distances for complexes **2–4**.

**Bond Distances (Å)**	**Complex (2)**	**Complex (3)**	**Complex (4)**
Co-C1	1.784(4)	1.8365(13)	1.7977(14)
Co-P1	2.2046(11)	2.2426(4)	2.1740(4)
Co-P2	2.2097(12)	2.2252(4)	2.1732(4)
Co-Cl1	2.2327(11)	-	-
Co-N3	-	1.7919(12)	-
Co-C32	-	-	2.0044(19)
**Bond Angles (°)**			
P1 Co1 P2	161.97(5)	160.958(15)	163.143(17)
C1 Co1 P1	81.77(12)	80.30(4)	81.65(5)
C1 Co1 P2	81.76(12)	80.92(4)	81.50(5)
C1 Co1 Cl1	174.95(14)	-	-
Cl1 Co1 P1	97.45(4)	-	-
Cl1 Co1 P2	99.60(5)	-	-
C1 Co1 N3	-	175.10(6)	-
N3 Co1 P1	-	102.75(4)	-
N3 Co1 P2	-	96.19(4)	-
C1 Co1 C32	-	-	175.59(10)
C32 Co1 P1	-	-	97.57(6)
C32 Co1 P2	-	-	99.17(6)

Table S3. Crystal and refinement data for complexes **2**–**4**.

	Complex (**2**)	Complex(**3**)	Complex (**4**)
Identification code (CCDC)	2363040	2363041	2363042
Empirical formula	$C_{31}H_{50}ClCoN_2P_2$	$C_{130}H_{133}B_2Co_2F_{48}N_6OP_4$	$C_{32}H_{53}CoN_2P_2$
Formula weight	607.05	2970.78	586.63
Temperature/K	100.00	102.15	100.15
Crystal system	orthorhombic	triclinic	tetragonal
Space group	Pbca	P-1	I-4
a/Å	*21.6299(3)*	19.72526(14)	*25.3091(3)*
b/Å	*13.35620(10)*	20.48338(15)	*25.3091(3)*
c/Å	22.5850(2)	20.83542(14)	*11.7019(2)*
α/°	90	68.2015(7)	90
β/°	90	74.1982(6)	90
γ/°	90	63.0477(7)	90
Volume/Å^3	6524.66(12)	6912.19(10)	*7495.7(2)*
Z	8	2	8
ρ_{calc}g/cm^3	1.236	1.427	1.040
μ/mm^{-1}	5.955	0.401	0.562
F(000)	2592.0	3038.0	2528.0
Crystal size/mm^3	0.12 × 0.12 × 0.09	0.348 × 0.319 × 0.136	0.336 × 0.214 × 0.151
Radiation	CuKα (λ = 1.54184)	MoKα (λ = 0.71073)	MoKα (λ = 0.71073)
2Θ range for data collection/°	7.83 to 150.304	5.096 to 67.708	5.006 to 66.548
Index ranges	-27 ≤ h ≤ 26, -16 ≤ k ≤ 16, -24 ≤ l ≤ 27	29 ≤ h ≤ 28, -28 ≤ k ≤ 30, -32 ≤ l ≤ 29	-37 ≤ h ≤ 36, -36 ≤ k ≤ 38, -17 ≤ l ≤ 16
Reflections collected	162016	177555	72038
Independent reflections	6701 [R_{int} = 0.0968, R_{sigma} = 0.0275]	47859 [R_{int} = 0.0229, R_{sigma} = 0.0256]	13093 [R_{int} = 0.0289, R_{sigma} = 0.0251]
Data/restraints/parameters	6701/0/358	47859/579/1819	13093/6/349
Goodness-of-fit on F^2	1.073	1.044	1.064
Final R indexes [I>=2σ (I)]	R_1 = 0.0674, wR_2 = 0.1448	R_1 = 0.0479, wR_2 = 0.1245	R_1 = 0.0274, wR_2 = 0.0692
Final R indexes [all data]	R_1 = 0.0735, wR_2 = 0.1475	R_1 = 0.0588, wR_2 = 0.1300	R_1 = 0.0321, wR_2 = 0.0708
Largest diff. peak/hole / e Å^{-3}	0.68/-0.71	1.94/-2.05	0.40/-0.17
Flack parameter	--	--	-0.011(3)

Computational Details

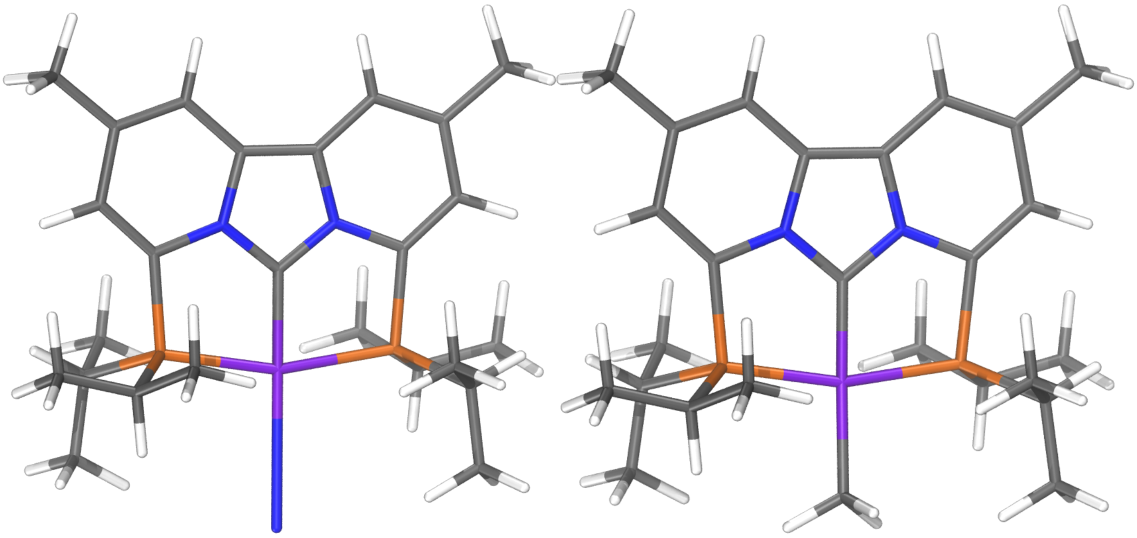

Figure S132. Computationally optimized structures for complexes **3** (left) and **4** (right).

Benchmarking procedure.

To select the computational method that accurately reproduces geometries, we adhered to a benchmarking procedure. The chosen functionals were ωB97xD,[11] M06-D3,[12], B3LYP-D3BJ,[13] and PBE0-D3BJ[14], combined with the def2SVP basis sets for C, H, N, P, O, and def2TZVP for Co. The optimized geometries were compared to the crystal structure of complex **3**. We employed the functional that demonstrated the smallest root mean square deviation of atomic positions (RMSD) between the crystal structure and the optimized geometry of the complex as the primary optimization method. Detailed RMSD results are presented in **Table S4**.

Table S4. Functionals tested during benchmarking procedure and RMSD values in comparison to the crystal structure of complex **3**.

**Tested method**	**RMSD**
ωB97xD/ C, H, N, P, O def2SVP/ Co def2TZVP	0.0513
M06-D3/ C, H, N, P, O def2SVP/ Co def2TZVP	0.0321
B3LYP-D3BJ/ C, H, N, P, O def2SVP/ Co def2TZVP	0.0583
PBE0-D3BJ/ C, H, N, P, O def2SVP/ Co def2TZVP	0.0129

Table S5. Calculated and experimentally determined bond distances for complexes **3** and **4**.

Bond Distances (Å)	Complex 3 (Exp.)	Complex 3 (Calc.)	Complex 4 (Exp.)	Complex 4 (Calc.)
Co-C1	1.805(6)	1.835	1.781(7)	1.803
Co-P1	2.2204(14)	2.233	2.180(3)	2.175
Co-P2	2.2380(14)	2.233	2.168(2)	2.160
Co-Cl1	-	-	-	-
Co-N3	1.775(5)	1.784	-	-
Co-C32	-	-	1.992(9)	1.995

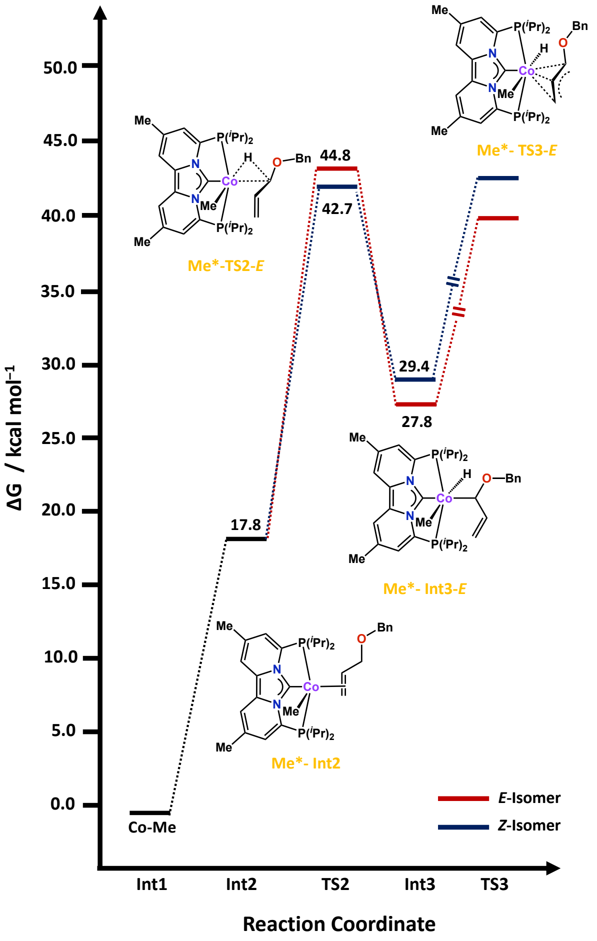

Figure S133. Calculated free energy profiles (ΔG) in kcal/mol at 353.15 K, for the isomerization of allylbenzyl ether with complex **4** without methyl migration.

Table S6. Total energy values (E) and Gibbs free energy values for the isomerization of allylbenzyl ether with complex **3** and **4**

name	PBE0/def2TZVP (gas Phase) (Hartrees)	Thermal correction to Enthalpy at 333.15K/ 1 mol/L (Hartrees)	Solvation correction (SMD model) (Hartrees)	Gibbs Free Energy (Hartrees)
ether	-463.104464	0.154119	-0.012401	-462.962747
N2	-109.446434	-0.011757	0.005491	-109.452699
Co-N2-Int1	-3258.592749	0.515754	-0.051309	-3258.128304
N2-Int2	-3721.709177	0.701796	-0.058489	-3721.065870
N2-TS2-E	-3721.658204	0.695172	-0.057207	-3721.020240
N2-Int3-E	-3721.684938	0.698978	-0.056901	-3721.042861
N2-Int4-E	-3721.696828	0.688121	-0.055037	-3721.063744
N2-TS2-Z	-3721.661814	0.694277	-0.055913	-3721.023449
N2-Int3-Z	-3721.679469	0.701460	-0.058233	-3721.036242
N2-Int4-Z	-3721.698500	0.692017	-0.053442	-3721.059925
Co-□-Int1	-3149.091128	0.509102	-0.058762	-3148.640787
Co-□-Int2	-3612.258977	0.694731	-0.061686	-3611.625933
Co-□-TS2-E	-3612.228594	0.693867	-0.059708	-3611.594434
Co-□-Int3-E	-3612.248846	0.698648	-0.058458	-3611.608656
Co-□-TS3-E	-3612.213402	0.689813	-0.065308	-3611.588897
Co-□-Int4-E	-3612.217781	0.685759	-0.063602	-3611.595623
Co-□-TS4-E	-3612.212576	0.685448	-0.065483	-3611.592611
Co-□-Int5-E	-3612.247746	0.694485	-0.059916	-3611.613178
Co-□-TS5-E	-3612.238754	0.694000	-0.058153	-3611.602906
Co-□-Int6-E	-3612.266160	0.693021	-0.059516	-3611.632656
Co-□-TSRE-E	-3612.215444	0.685045	-0.062172	-3611.592571
Co-□-TS2-Z	-3612.231592	0.694117	-0.058805	-3611.596280
Co-□-Int3-Z	-3612.244008	0.696151	-0.058438	-3611.606295
Co-□-TS3-Z	-3612.215952	0.688846	-0.063008	-3611.590114
Co-□-Int4-Z	-3612.221343	0.689002	-0.061089	-3611.593430
Co-□-TS4-Z	-3612.217383	0.690494	-0.063073	-3611.589962
Co-□-Int5-Z	-3612.248630	0.697514	-0.058059	-3611.603175
Co-□-TS5-Z	-3612.227789	0.685639	-0.061033	-3611.603183
Co-□-Int6-Z	-3612.266163	0.693410	-0.059949	-3611.632702
Co-□-TSRE-Z	-3612.218158	0.685687	-0.060863	-3611.593334

name	PBE0/def2TZVP (gas Phase) (Hartrees)	Thermal correction to Enthalpy at 353.15K/ 1 mol/L (Hartrees)	Solvation correction (SMD model) (Hartrees)	Gibbs Free Energy (Hartrees)
Me-Int1	-3189.134328	0.541495	-0.026655	-3188.619487
Me-Int2	-3652.252182	0.721372	-0.031995	-3651.562805
Me-TS2	-3652.232994	0.719301	-0.030229	-3651.543922
Me-Int3	-3652.243954	0.719579	-0.030959	-3651.555334
Me-TS3-E	-3652.236087	0.717666	-0.031223	-3651.549643
Me-Int4-E	-3652.243285	0.720799	-0.030745	-3651.553231
Me-TS4-E	-3652.214720	0.712002	-0.034855	-3651.537574
Me-Int5-E	-3652.215208	0.706151	-0.034240	-3651.543297
Me-TS5-E	-3652.214720	0.712117	-0.034869	-3651.537472
Me-Int6-E	-3652.243884	0.721552	-0.030623	-3651.552955
Me-TS6-E	-3652.230595	0.717320	-0.031317	-3651.544591
Me-Int7-E	-3652.254400	0.718446	-0.030509	-3651.566462
Me-TS7-E	-3652.230179	0.717504	-0.029425	-3651.542100
Me-Int8-E	-3652.251902	0.719419	-0.031546	-3651.564029

Me-TSRE-E	-3652.212679	0.713884	-0.030839	-3651.529634
Me-TS3-Z	-3652.228367	0.718905	-0.030141	-3651.539602
Me-Int4-Z	-3652.241264	0.719051	-0.030248	-3651.552461
Me-TS4-Z	-3652.216053	0.713970	-0.033374	-3651.535457
Me-Int5-Z	-3652.219911	0.711432	-0.033709	-3651.542188
Me-TS5-Z	-3652.216050	0.713694	-0.033399	-3651.535755
Me-Int6-Z	-3652.234777	0.718907	-0.030598	-3651.546467
Me-TS6-Z	-3652.222650	0.713453	-0.032631	-3651.541828
Me-Int7-Z	-3652.251155	0.718360	-0.030622	-3651.563416
Me-TS7-Z	-3652.228137	0.717351	-0.029642	-3651.540428
Me-Int8-Z	-3652.249285	0.720252	-0.031511	-3651.560544
Me-TSRE-Z	-3652.210747	0.712892	-0.031259	-3651.529115
Me-Int1	-3189.134328	0.541495	-0.026655	-3188.619487
Me-Int2	-3652.252182	0.721372	-0.031995	-3651.562805
Me*-TS2-E	-3652.204160	0.721667	-0.031632	-3651.514126
Me*-Int3-E	-3652.232458	0.723336	-0.032121	-3651.541243
Me*-TS2-Z	-3652.210791	0.724278	-0.030953	-3651.517466
Me*-Int3-Z	-3652.234289	0.727533	-0.031879	-3651.538635

Table S7. Calculated Free energy profiles (ΔG) in kcal/mol for the isomerization of allylbenzyl ether with complex **3** and **4**.

**name**	**$\Delta G0_{(289.15K)}$**[1]	**$\Delta G1_{(289.15K)}$**[2]	**$\Delta G2_{(333.15K)}$**[3]
ether	0	0	0
N2	0	0	0
Co-N2-Int1	0	0	0
N2-Int2	9.9	15.9	15.8
N2-TS2-E	38.8	44.6	44.4
N2-Int3-E	23.3	30.3	30.2
N2-Int4-E	13.7	17.8	17.1
N2-TS2-Z	36.1	42.6	42.4
N2-Int3-Z	27.8	34.2	34.4
N2-Int4-Z	15.2	20.0	19.5
Co-□-Int1	24.2	20.9	21.8
Co-□-Int2	6.0	7.0	7.8
Co-□-TS2-E	23.9	26.5	27.6
Co-□-Int3-E	14.1	17.4	18.6
Co-□-TS3-E	28.6	30.2	31.0
Co-□-Int4-E	24.9	26.3	26.8
Co-□-TS4-E	26.9	28.1	28.7
Co-□-Int5-E	12.5	14.8	15.8
Co-□-TS5-E	17.9	21.2	22.2
Co-□-Int6-E	0.5	2.8	3.6
Co-□-TSRE-E	27.2	28.1	28.7
Co-□-TS2-Z	21.7	25.3	26.4
Co-□-Int3-Z	15.6	19.0	20.1
Co-□-TS3-Z	27.4	29.5	30.3
Co-□-Int4-Z	25.1	27.5	28.2
Co-□-TS4-Z	27.3	29.5	30.4
Co-□-Int5-Z	17.2	17.8	18.3
Co-□-TS5-Z	19.6	21.5	22.1
Co-□-Int6-Z	-0.3	2.8	3.5
Co-□-TSRE-Z	25.8	27.6	28.2
**Name**	**$\Delta G0_{(289.15K)}$**	**$\Delta G1_{(289.15K)}$**	**$\Delta G2_{(353.15K)}$**
Me-Int1	0.0	0.0	0.0
Me-Int2	11.2	17.7	14.3
Me-TS2	21.7	29.6	26.1
Me-Int3	13.0	22.6	18.9
Me-TS3-E	18.1	26.0	22.5
Me-Int4-E	15.2	23.6	20.3
Me-TS4-E	26.6	34.0	30.1
Me-Int5-E	23.5	31.1	26.5
Me-TS5-E	26.7	34.1	30.2
Me-Int6-E	15.4	23.7	20.4
Me-TS6-E	20.6	29.2	25.7
Me-Int7-E	6.4	15.8	12.0
Me-TS7-E	22.8	30.9	27.3
Me-Int8-E	9.5	17.2	13.5
Me-TSRE-E	30.7	38.8	35.1
Me-TS3-Z	23.1	32.2	28.8
Me-Int4-Z	15.3	24.3	20.8
Me-TS4-Z	27.3	35.2	31.4

Me-Int5-Z	23.5	31.3	27.2
Me-TS5-Z	27.1	35.0	31.2
Me-Int6-Z	19.5	28.1	24.5
Me-TS6-Z	23.4	31.3	27.4
Me-Int7-Z	7.6	17.7	13.9
Me-TS7-Z	23.7	32.0	28.3
Me-Int8-Z	12.0	19.3	15.7
Me-TSRE-Z	31.1	39.2	35.4
Me-Int1	0.0	0.0	0.0
Me-Int2	11.17	17.7	14.3
Me*-TS2-E	37.23	42.9	44.8
Me*-Int3-E	20.58	25.9	27.8
Me*-TS2-Z	35.20	40.6	42.7
Me*-Int3-Z	21.14	27.3	29.4

[1]ΔG0(289.15K) refers to the Gibbs free energies defined as the sum of electronic energy (PBE0-D3BJ/def2SVP for C, H, N, O, P and def2TZVP for Co, solvation correction M052X-D3/def2SVP for C,H,N,O,P and def2TZVP for Co and, thermal correction to Gibbs free energy at 298.15K. [2]ΔG1(289.15K) refers to the Gibbs free energies defined as the sum of electronic energy (PBE0-D3BJ/def2TZVP, solvation correction M052X-D3/def2SVP for C,H,N,O,P and def2TZVP for Co and, thermal correction to Gibbs free energy at 298.15K. [3]ΔG2(333.15K) refers to the Gibbs free energies defined as the sum of electronic energy (PBE0-D3BJ/def2TZVP, solvation correction M052X-D3/def2SVP for C,H,N,O,P and def2TZVP for Co and, thermal correction to Gibbs free energy at 333.15K and concentration (from standard state in gas phase, 1 atm, to standard state in solution, 1 mol/l).

Geometries of intermediates and transition states involved in the alkene isomerization

All geometries of the intermediates and transition state structures are provided as xyz-files in a separate supporting information zip file.

Input templates.

Gaussian 09, Revision D.01
Geometry optimization of transition states:

```
#P PBE1PBE/genecp vshift=300
# opt=(TS,noeigen,calcfc,MaxCycle=500) Freq Int=UltraFine
# SCF=(novaracc,Fermi) SCFCyc=180 5d 7f Nosymm  EmpiricalDispersion=GD3BJ
# IOp(1/7=67,1/8=5)
```

Geometry optimization of transition states with qst method:

```
#P PBE1PBE/genecp vshift=300
# opt=(qst2,Redundant) Freq Int=UltraFine
# SCF=(novaracc,fermi) SCFCyc=180 5d 7f Nosymm  EmpiricalDispersion=GD3BJ
# IOp(1/7=67,1/8=5)
```

Geometry optimization of intermediates:

```
#P pbe1pbe/genecp vshift=300
# opt=(calcfc,MaxCycle=500,tight) Freq Int=UltraFine
# SCF=(novaracc,Fermi) SCFCyc=280 5d 7f Nosymm EmpiricalDispersion=GD3BJ
# IOp(1/7=67,1/8=5)
```

Solvent correction:

```
#P M052X/GenECP SP vshift=300  Int=UltraFine
# SCF=(novaracc,Conver=5) SCFCyc=180 5d 7f
# NoSymm SCRF=(smd,solvent=Benzene)  EmpiricalDispersion=GD3
```

References

(1) Motoyama, Y.; Abe, M.; Kamo, K.; Kosako, Y.; Nagashima, H. Encapsulated molecular catalysts in polysiloxane gels: ruthenium cluster-catalyzed isomerization of alkenes. *Chem. Commun.* **2008**, 5321-5323.

(2) Kona, C. N.; Patil, M. N.; Ramana, C. V. Gold(i) catalyzed [1,3] O → C rearrangement of benzylvinyl ethers. *Org. Chem. Front.* **2016**, *3*, 453-456.

(3) Harada, N.-a.; Nishikata, T.; Nagashima, H. Vinyl polymerization versus [1,3] O to C rearrangement in the ruthenium-catalyzed reactions of vinyl ethers with hydrosilanes. *Tetrahedron* **2012**, *68*, 3243-3252.

(4) Krompiec, S.; Kuznik, N.; Urbala, M.; Rzepa, J. Isomerization of alkyl allyl and allyl silyl ethers catalyzed by ruthenium complexes. *J. Mol. Catal. A: Chem.* **2006**, *248*, 198-209.

(5) Wipf, P.; Waller, D. L.; Reeves, J. T. Transition-Metal-Mediated Cascade Reactions: The Water-Accelerated Carboalumination−Claisen Rearrangement−Carbonyl Addition Reaction. *J. Org. Chem.* **2005**, *70*, 8096-8102.

(6) Su, C.-C.; Williard, P. G. Isomerization of Allyl Ethers Initiated by Lithium Diisopropylamide. *Org. Lett.* **2010**, *12*, 5378-5381.

(7) Crivello, J. V.; Kong, S. Efficient Isomerization of Allyl Ethers and Related Compounds Using Pentacarbonyliron. *J. Org. Chem.* **1998**, *63*, 6745-6748.

(8) Yang, X.; Gitter, S. R.; Roessler, A. G.; Zimmerman, P. M.; Boydston, A. J. An Ion-Pairing Approach to Stereoselective Metal-Free Ring-Opening Metathesis Polymerization. *Angew. Chem., Int. Ed.* **2021**, *60*, 13952-13958.

(9) Rullière, P.; Carret, S.; Milet, A.; Poisson, J.-F. Kinetic Resolution in the [2+2] Cycloaddition of Ketenes: An Experimental and Theoretical Study. *Chem. - Eur. J.* **2015**, *21*, 3876-3881.

(10) Gauthier, D.; Lindhardt, A. T.; Olsen, E. P. K.; Overgaard, J.; Skrydstrup, T. In Situ Generated Bulky Palladium Hydride Complexes as Catalysts for the Efficient Isomerization of Olefins. Selective Transformation of Terminal Alkenes to 2-Alkenes. *J. Am. Chem. Soc.* **2010**, *132*, 7998-8009.

(11) Chai, J.-D.; Head-Gordon, M. Long-Range Corrected Hybrid Density Functionals with Damped Atom–Atom Dispersion Correction. *Phys. Chem. Chem. Phys.* **2008**, *10,* 6615-6620

(12) Zhao, Y.; Truhlar, D. G. The M06 suite of density functionals for main group thermochemistry, thermochemical kinetics, noncovalent interactions, excited states, and transition elements: two new functionals and systematic testing of four M06-class functionals and 12 other functionals. *Theor. Chem. Acc.* **2008**, *120*, 215-241.

(13) Becke, A. D. Density-Functional Thermochemistry. III. The Role of Exact Exchange. *J. Chem. Phys.* **1993**, *98,* 5648-5652

(14) Adamo, C.; Barone, V. Toward Reliable Density Functional Methods without Adjustable Parameters: The PBE0 model. *J. Chem. Phys.* **1999**, *110*, 6158-6170
